# The Expansion and Functional Diversification of the Mammalian Ribonuclease A Superfamily Epitomizes the Efficiency of Multigene Families at Generating Biological Novelty

**DOI:** 10.1093/gbe/evt161

**Published:** 2013-10-25

**Authors:** Stephen M. Goo, Soochin Cho

**Affiliations:** Department of Biology, Creighton University

**Keywords:** host defense, angiogenin, gene duplication, positive selection, gene sorting, adaptive evolution

## Abstract

The ribonuclease (RNase) A superfamily is a vertebrate-specific gene family. Because of a massive expansion that occurred during the early mammalian evolution, extant mammals in general have much more RNase genes than nonmammalian vertebrates. Mammalian RNases have been associated with diverse physiological functions including digestion, cytotoxicity, angiogenesis, male reproduction, and host defense. However, it is still uncertain when their expansion occurred and how a wide array of functions arose during their evolution. To answer these questions, we generate a compendium of all RNase genes identified in 20 complete mammalian genomes including the platypus, *Ornithorhynchus anatinus*. Using this, we delineate 13 ancient RNase gene lineages that arose before the divergence between the monotreme and the other mammals (∼220 Ma). These 13 ancient gene lineages are differentially retained in the 20 mammals, and the rate of protein sequence evolution is highly variable among them, which suggest that they have undergone extensive functional diversification. In addition, we identify 22 episodes of recent expansion of RNase genes, many of which have signatures of adaptive functional differentiation. Exemplifying this, bursts of gene duplication occurred for the RNase1, RNase4, and RNase5 genes of the little brown bat (*Myotis lucifugus*), which might have contributed to the species’ effective defense against heavier pathogen loads caused by its communal roosting behavior. Our study illustrates how host-defense systems can generate new functions efficiently by employing a multigene family, which is crucial for a host organism to adapt to its ever-changing pathogen environment.

## Introduction

All living organisms face incessant needs for generating biological innovations to adapt to their ever-changing environment. Gene duplication is one of the major sources for generating such biological novelty (Ohno 1970; Kondrashov 2012). Through various evolutionary mechanisms, duplicated genes often acquire new functions that open new evolutionary trajectories for a species, which might not be possible with only one gene (Innan and Kondrashov 2010). The role of gene duplication in an organism’s adaptation is perhaps most evident in the evolution of the host-defense system (Dyer and Rosenberg 2006; Rosenberg 2008b). To cope with pathogens that constantly change their evolutionary paths, host species need a continuous feed of new genes or modifications on existing genes to come up with more effective defense against them. One of the most efficient ways to achieve this goal is to utilize a multigene family, a group of genes with shared ancestry generated by multiple gene duplication events. Many host-defense systems employ multigene families, such as major histocompatibility complex genes and immunoglobulin genes in animals, reflecting the power of gene duplication to increase the complexity and efficacy of host-defense systems (Nei et al. 1997).

The ribonuclease (RNase) A superfamily has been a good model system for studying gene family evolution in the context of its roles in host defense (Cho et al. 2005; Rosenberg 2008b). It was named after RNase A, the bovine pancreatic RNase, which is one of the first enzymes to be characterized and perhaps the most extensively studied enzyme (Raines 1998). Its structure and ribonucleolytic (or RNA-degrading) mechanism have been extensively studied. Furthermore, the cow RNase A and the RNases identified in many other vertebrates were used for several pioneering studies in the emerging field of molecular evolution in the 1960s and 1970s (Epstein 1967; Beintema et al. 1973; van den Berg and Beintema 1975). A typical RNase gene encodes protein with approximately 130 amino acids in a protein-coding region that is not interrupted by introns. RNase proteins have a N-terminal signal sequence for secretion and six to eight conserved cysteines forming disulfide bridges. They have three distinct catalytic amino acid residues, called as the catalytic triad, and the signature motif “CKXXNTF” (Beintema and Kleineidam 1998). The RNase A superfamily does not share sequence homology with any other RNase families such as RNase H, RNase III, and RNase P. For convenience, we will use the term “RNase” in this article to strictly refer to the members of the RNase A superfamily.

RNase genes are present only in vertebrates. Basal chordates, such as tunicates and amphioxus, and invertebrate metazoans do not have RNase genes (Cho et al. 2005; Cho and Zhang 2006). The human (*Homo sapiens*) has 13 functional RNase genes (Cho et al. 2005). Eight of them were identified first, which includes RNase1, RNase2 (also known as eosinophil-derived neurotoxin or EDN), RNase3 (also known as eosinophil cationic protein or ECP), RNase4, RNase5 (also known as angiogenin), RNase6 (also known as RNase k6), RNase7, and RNase8. They are known as the canonical RNases because they have the key sequence features of RNases such as the catalytic triad and the signature “CKXXNTF” motif. They have a wide range of physiological functions including degrading dietary RNAs, cytotoxicity, apoptosis, angiogenesis, sperm maturation, antibacterial, and antiviral activities, which are summarized in earlier articles (Barnard 1969; Rosenberg et al. 1989; Beintema 1990; Domachowske et al. 1998; Rosenberg 2008a; Sorrentino 2010; Gupta et al. 2013). More recently, five additional RNases were discovered in the human genome, which were named RNase9, RNase10, RNase11, RNase12, and RNase13 (Penttinen et al. 2003; Castella et al. 2004; Devor et al. 2004; Cho et al. 2005). Two additional genes in the human genome (RNAses 14 and 15), identified in this study, are not functional. RNases 13–15 are known as noncanonical RNases because the catalytic triad and/or the signature motif are missing and perhaps do not have ribonucleolytic activities. The physiological functions of these noncanonical RNases have not been extensively studied. RNase9 and RNase10 are expressed in the epididymis of the human, the mouse, and the rat, implying their functions in the male reproductive tract (Zhu et al. 2007; Liu et al. 2008; Cheng et al. 2009; Krutskikh et al. 2012). Recombinant human RNase9 protein has been implicated for antibacterial activities, and the mouse RNase10 is required for sperm maturation (Cheng et al. 2009; Krutskikh et al. 2012). The functions of RNase11, RNase12, and RNase13 are currently unknown. In the human genome, all these 13 RNase genes are located in a just approximately 500-kb region on the long arm of chromosome 14 (14q11.2) (Cho et al. 2005).

Nonmammalian vertebrates, such as fish, reptiles, and birds, have a small number of RNase genes ranging 3–5, whereas mammals in general have much more RNase genes. For example, the mouse (*Mus musculus*) has more than 20 (Rosenberg et al. 2001; Nitto et al. 2005, 2006; Pizzo et al. 2006, 2008, 2011; Cho and Zhang 2007; Tao et al. 2011). Using the RNases identified in the six mammalian genomes, including human, mouse, rat, cow, dog, and opossum, Cho and Zhang (2006) previously determined that the expansion of the RNase A superfamily occurred in the common ancestor of these six mammals before the divergence of the marsupial and the placental mammals (∼190 Ma). At the time of the Cho and Zhang’s study, only those six mammals’ genomes were completely sequenced and publicly available, thus their study had a rather limited scope for drawing a full picture of the evolution of mammalian RNase genes. Since then, we have seen dozens of additional mammalian genome projects being finished, many of which were done by the 29 mammal project led by Broad Institute (Lindblad-Toh et al. 2011). Although many of these recent genomes have low coverage, typically 2×, some have enough depth and coverage (6× or higher) suitable for more thorough comparative genomic studies.

Here in this article, we generate a compendium of the RNase genes in 20 high-coverage mammalian genomes representing 10 different orders of eutherian (placental), metatherian (marsupial), and prototherian (monotreme) mammals (table 1 and supplementary table S1, Supplementary Material online). By analyzing these RNases, we make four key discoveries on how mammalian RNase genes have evolved. First, we identify 13 ancient RNase gene lineages that are present in all extant eutherian mammals, which resulted from the expansion of the RNase A superfamily in the early mammalian evolution. Second, taking advantage of the basal position of the platypus (*Ornithorhynchus anatinus*) in the mammalian phylogeny, we demonstrate that the 13 ancient RNase gene lineages arose before the divergence between the prototherian and the therian lineages (∼220 Ma), which indicates that the mammalian expansion of the gene family is more ancient than previously known. Third, we show that there is a great degree of gene retention variation among the 13 ancient gene lineages, where some gene lineages are retained in more species than others lineages. Furthermore, we find that the rates of protein sequence change, measured as the ratio of nonsynonymous to synonymous substitutions (*d*N/*d*S), vary greatly among different gene lineages. These results suggest that the 13 ancient gene lineages diversified their physiological functions after they arose, thus evolving under different selective regimes. Fourth, we identify 22 cases where one or two recent gene duplication events generated multiple paralogs in a species. We also show that the rate of protein sequence evolution is elevated in many of these paralog groups, which suggest their adaptive roles (Nei et al. 1997). Among those, we further analyze the bursts of gene duplication that occurred for three RNases in the little brown bat to raise a possibility that these expansions have contributed to the species’ host defense.

**Table 1:** The Gene Count for the 13 Ancient RNase Gene Lineages Identified in the 20 Mammals Included in This Study

Common Name	R1	R2/3	R4	R5	R6	R7/8	R9	R10	R11	R12	R13	R14	R15	Others^a^	Total
Human	1	2 (1)	1	1	1	2	1	1	1	1	1	(2)	(1)	0	13 (4)
Chimpanzee	1	2	1	1	1	2	1	1	1	1	1	(1)	(1)	0	13 (2)
Gorilla	1	2 (1)	1	1	1	1 (1)	1	1	1	1	1	(2)	(1)	0	12 (3)
Orangutan	1	2 (1)	1	1	1	2	1	1	1	1	1	(3)	(1)	0	13 (5)
Gibbon	1	1	1	0	1	1 (1)	1	1	1	1	1	0	(1)	0	10 (2)
Rhesus macaque	1	2	1	1	1	2	2	1	1	1	1	(2)	(1)	0	14 (3)
Marmoset	1	1	1	1	1	2	1	1	0	1	(1)	0	(1)	0	10 (2)
Mouse	1	7 (11)	1	5 (3)	1	0	1	1	1	1	1	0	0	0	20 (15)
Rat	3	5 (1)	1	2	1	0	1	1	1	1	1	0	0	0	17 (1)
Naked mole rat	1 (4)	0	1	0	4 (3)	1 (1)	1	1	(1)	1	1	0	0	0	11 (9)
Guinea pig	3 (1)	0	2	0	3 (2)	1 (1)	0	1	1	1	1	0	(1)	0	13 (5)
Rabbit	1	3	1	1 (1)	1	2	1	1	1	1	1	0	0	0	15 (1)
Cow	3	2 (1)	1 (1)	3	1	2 (1)	2 (1)	1	1	1	1	1	1	0	19 (3)
Horse	1	1 (1)	1 (1)	1	1	1 (1)	1	1	1	1	0	(1)	(1)	0	10 (5)
Dog	1	0	1	(1)	1 (1)	0	1	1	(1)	1	1	0	0	0	7 (3)
Giant panda	1	0	1	1	5 (1)	1 (1)	1	1	1	1	1	(1)	0	0	14 (3)
Little brown bat	7	2	11 (3)	7 (9)	1 (7)	1 (1)	1	1	1	1	1	(1)	(1)	0	34 (22)
African elephant	1 (2)	(1)	1	(1)	3	1 (1)	1	1	1	1	1	(1)	0	0	11 (6)
Opossum	1	0	1	1	0	1	0	0	0	1	1 (1)	0	0	15 (1)	21 (2)
Platypus	0	0	1	0	0	0	0	0	0	0	1	0	0	3	5

Note.—“R” stands for “RNase.” For example, “R2/3” indicates the lineage of RNase2/3. Numbers in parentheses indicate the number of pseudogenes.

^a^This group does not represent a monophyletic gene lineage, but it simply includes the species-specific genes identified in the opossum and the platypus that do not belong to any of the 13 ancient gene lineages (RNases 1–15).

Our study, empowered by the 20 high-quality mammalian genomes, provides a full picture of how the mammalian RNase A superfamily arose, expanded, and functionally diversified during evolution. It also provides general insights on how biological systems that require incessant functional changes such as host-defense systems can employ gene families to achieve this goal.

## Materials and Methods

### Nomenclature

In this article, we use “human” for *Homo sapiens*, “chimpanzee” for the common chimpanzee *Pan troglodytes* (Pt), “gorilla” for the western lowland gorilla *Gorilla gorilla* (Gg), “orangutan” for the Sumatran orangutan *Pongo pygmaeus* (Pp), “gibbon” for the northern white-cheeked gibbon *Nomascus leucogenys* (Nl), “rhesus monkey” for the rhesus macaque *Macaca mulatta* (Mmu), “marmoset” for the common marmoset *Callithrix jacchus* (Cj), “mouse” for the house mouse *M. musculus* (Mm), “rat” for the Norway brown rat *Rattus norvegicus* (Rn), “naked mole rat” for *Heterocephalus glaber* (Hg), “guinea pig” for *Cavia porcellus* (Cp), “rabbit” for the European rabbit *Oryctolagus cuniculus* (Oc), “cow” for the domesticated cow *Bos taurus* (Bt), “horse” for *Equus caballus* (Ec), “dog” for *Canis familiaris* (Cf), “giant panda” for *Ailuropoda melanoleuca* (Am), “little brown bat” for *Myotis lucifugus* (Ml), “elephant” for the African elephant *Loxodonta africana* (La), “opossum” for the gray short-tailed opossum *Monodelphis domestica* (Md), and “platypus” for *O. anatinus* (Oa), unless otherwise specified. By “functional gene,” we mean that the gene sequence under investigation is contained in an uninterrupted open reading frame (ORF), and, conversely, a sequence is considered as a “pseudogene” if the ORF is interrupted by one or more premature stop codons anywhere in the ORF or by frame-shifting insertions/deletions. However, the expression status of many “functional” genes still needs experimental verification. Although much is known about the localization, expression, and functional properties of human and murid RNases, much less is known in this respect about the RNases in the other species. Recently, Wheeler et al*.* (2012) made a careful study of the expression of cow RNases. Pseudogenes are distinguished from functional genes by putting “ps” in the name (e.g., *H. Sapiens*-RNase2ps). In this article, we use the term “RNase” to strictly refer to the members of the RNase A superfamily only.

### Identification of RNase Genes

We used the protein sequences of all 13 human RNase genes (RNases1–13) and 19 cow RNase genes, including the cow-specific RNase14 and RNase15, as queries to run TBlastN searches in the 20 completed mammalian genome sequence databases available at the National Center for Biotechnology Information (NCBI) and the Ensembl genome database. The assembly versions and the coverage number of the genomes used in this study are listed in supplementary table S1, Supplementary Material online. We identified pseudogenes by running BlastN searches in the database using the nucleotide sequences of the identified RNase genes as queries. We used 10^−^^10^ as the *E*-value cutoff in all our TBlastN and BlastN searches. We also used the UCSC genome browser (http://genome.ucsc.edu, last accessed November 9, 2012) to run BLAT searches to confirm the presence of the identified genes and pseudogenes and also to obtain their chromosomal coordinates. The chromosomal or scaffold coordinates of all the RNase genes included in this study are listed in supplementary table S2, Supplementary Material online. The nucleotide and amino acid sequences of all the RNase genes are provided in the supplementary dataset, Supplementary Material online.

### Sequence Alignment and Evolutionary Analysis

Protein and nucleotide sequence alignments were made by ClustalX (Thompson et al. 2002) with manual adjustments. MEGA5 was used for evolutionary analyses (Tamura et al. 2011). Phylogenetic trees were reconstructed using the neighbor-joining method (Saitou and Nei 1987) with 2,000 bootstrap replications (Felsenstein 1985). For all the phylogenetic analyses we did, the complete deletion option in MEGA5 was enforced. That is, any site with a gap in one or more sequences was removed from the analysis. For generating evolutionary trees based on nucleotide sequences and protein sequences, we used the Kimura’s two-parameter model (Kimura 1980) and the *p*-distance model (Nei and Kumar 2000), respectively. Numbers of synonymous (*d*S) and nonsynonymous (*d*N) nucleotide substitutions per site between homologous DNA sequences were computed by the modified Nei–Gojobori method (Zhang et al. 1998).

## Results

### The Numbers of RNase A Genes Vary Greatly among 20 Mammalian Genomes

To trace the evolutionary history of the mammalian RNase A superfamily, we searched for the RNase genes and pseudogenes in the genomes of the 20 mammalian species with high coverage (6× or higher). The assembly versions and the coverage numbers of the 20 mammalian genomes are listed in supplementary table S1, Supplementary Material online. We did not include any genomes with coverage less than 6×, because not being able to identify a gene in such low-coverage genomes could be simply due to a genome sequencing omission rather than its true absence in the species. The 20 mammalian species we study here represent a wide range within the extant mammalian phylogeny including 10 different orders: one prototherian (monotreme), one metatherian (marsupial), and eight eutherian (placental) orders (supplementary table S1, Supplementary Material online). The numbers of all the RNase genes identified in these 20 mammalian genomes are listed in table 1. In table 1, RNase2 and RNase3 are grouped together as the RNase2/3 gene lineage because their duplication is a relatively recent event that occurred in the ancestral lineage of all catarrhine primates (old-world monkeys and apes) (Rosenberg et al. 1995). For the same reason, RNase7 and RNase8 are also grouped together as the RNase7/8 gene lineage because their divergence time is also relatively recent (see later). Therefore, there are 13 ancient gene lineages to which all the extant eutherian RNase A genes can be classified: RNase1, RNase2/3, RNase4, RNase5, RNase6, RNase7/8, RNase9, RNase10, RNase11, RNase12, RNase13, RNase14, and RNase15 (table 1).

### The Order and Direction of the RNase Genes Are Well Conserved during Mammalian Evolution

**Fig. 1.—:**
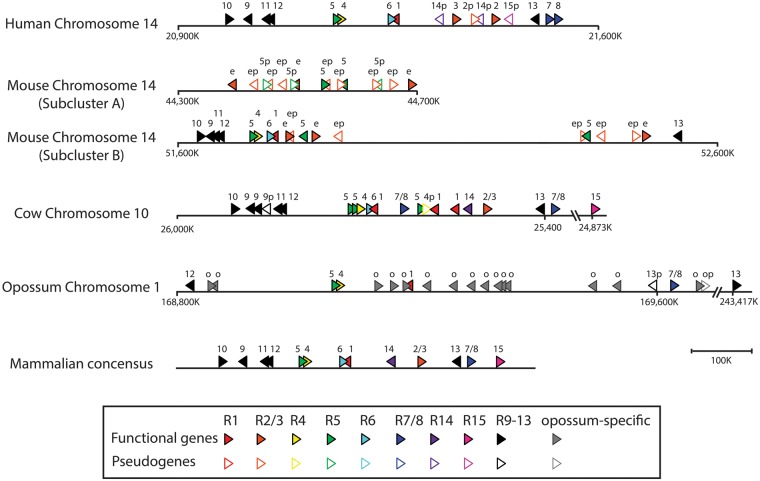
Chromosomal maps of all RNase genes identified in the human, the mouse, the cow, and the opossum. Gene locations are drawn to scale. Arrowheads indicate transcription directions. Filled arrowheads represent functional genes and open arrowheads symbolize pseudogenes. The mammalian consensus map shows the general pattern of gene order and direction of the 13 ancient RNase gene lineages as shown in supplementary table S2, Supplementary Material online. Different colors were used to represent different gene lineages: red for RNase1, orange for RNase2/3, yellow for RNase4, green for RNase5, light blue for RNase6, dark blue for RNase7/8, purple for RNase14, magenta for RNase15, and black for RNases9–13. Numbers above the arrowhead symbols indicate the gene lineage (e.g., “4” for RNase4). Gray arrowheads represent the opossum-specific genes that are not found in any eutherian mammals, and these genes were labeled with “o” above the gray arrowhead symbols. The mouse EARs are labeled with an “e” above the arrowhead. Pseudogenes are denoted by a “p.”

The current assemblies of 15 of the 20 mammalian genomes are relatively in high quality, and the chromosomal locations of their RNase genes are available as numerical coordinates. However, chromosomal coordinates are not available for the other five genomes including the gibbon, the giant panda, the little brown bat, the elephant, and the platypus. These genomes are fragmented with hundreds of scaffolds. For these lower quality genomes, we obtained scaffold coordinates instead. The chromosomal or scaffold coordinates of all RNase genes identified in the 20 mammals are listed in supplementary table S2, Supplementary Material online. Our chromosomal coordinates for the human, the mouse, and the rat, obtained from more recent genome assemblies, correct those reported in two earlier studies (Cho et al. 2005; Cho and Zhang 2006). On the basis of a genomic contig (NT_026437) of a more recent mouse genome assembly (build 38), Wheeler et al*.* (2012) also published the chromosomal coordinates of the mouse RNases, which is consistent with our results. The chromosomal coordinates we obtained from the current cow genome (Baylor4.6.1/bosTau7) are more current than those reported in a previous study (Wheeler et al. 2012), which used an earlier version (Bos_taurus_UMD_3.1/bosTau6), but their gene order is essentially identical to ours. A chromosomal map is drawn to scale for each of the four selected species (human, mouse, cow, and opossum) that have relatively high-quality genome assemblies (fig. 1). For these four genomes and 11 others that have chromosomal coordinates (supplementary table S2, Supplementary Material online), we found that all of their RNase genes are located within a single chromosomal block of 0.5–1 mega base pairs (Mbps). An exception to this rule is the mouse genome, where RNase genes are located in two separate clusters that are just 69 Mbps apart on the same chromosome (fig. 1). Furthermore, the order and transcriptional directions of the RNase genes are well conserved in these mammals. The general consensus pattern of the gene order is as follows: the quartet of RNases9–12, the pair of RNase5–RNase4 (in the same transcriptional direction), the pair of RNase6–RNase1 (in opposite transcriptional directions), RNase14, RNase2/3, RNase13, RNase7/8, and RNase15 (fig. 1 and supplementary table S2, Supplementary Material online). It is remarkable that, although the total RNase gene counts vary greatly among these 15 mammalian species, the clustering, the gene orders, and the transcriptional directions of RNase genes have been conserved well across these species over approximately 190 million years of the therian (marsupial and placental mammals) evolution.

### The 13 Ancient RNase Gene Lineages Arose by Gene Duplication Events in the Common Ancestor of All Extant Mammals

**Fig. 2.—:**
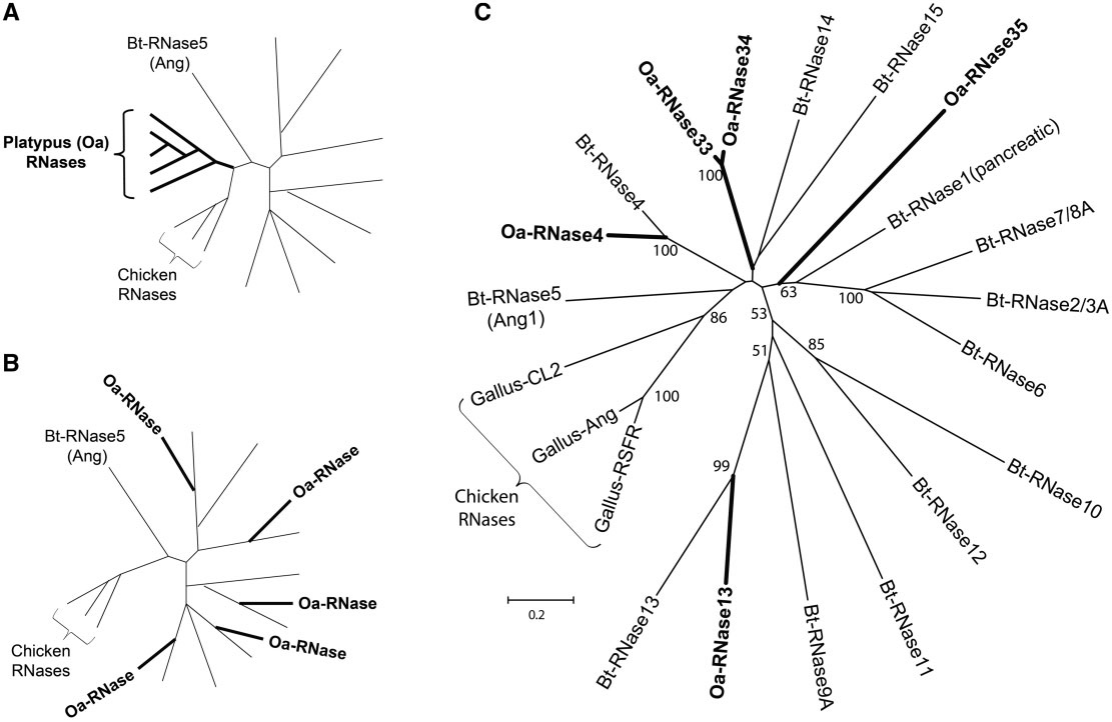
Testing two hypotheses on the origin of the 13 ancient RNase gene lineages using a phylogenetic tree of five platypus (Oa), 13 cow (Bt), and three chicken (Gg) RNase genes. Two hypothetical trees (*A* and *B*) were made arbitrarily just to make a contrast between two hypotheses, and the real-data tree (*C*) was made using real sequences. The tree in panel (*A*) supports the hypothesis that the 13 ancient RNase gene lineages, represented by the 13 cow RNases, arose after the divergence between the platypus, whereas the tree in panel (*B*) supports the hypothesis that they arose before the divergence. The names and branches of the platypus RNases are bolded. In two hypothetical trees (*A* and *B*), branches without labels represent the cow RNases other than Bt-RNase5 (angiogenin), which is most closely related to nonmammalian RNases such as the chicken RNases. For the tree in panel (*C*), a total of 105 amino acid sites are used in tree making. We used the neighbor-joining methods with *p* distance and 1,000 bootstrap replications. The scale bar number indicates the number of amino acid substitutions per site.

Using one metatherian (opossum) and five eutherian (human, mouse, rat, cow, and dog) genomes, Cho and Zhang (2006) previously showed that the expansion of the mammalian RNase A superfamily occurred before the divergence of the marsupials and the placental mammals, which occurred approximately 190 Ma (Meredith et al. 2011). The platypus, a monotreme, takes the most basal position in the mammalian phylogeny, diverging from the therian mammals approximately 220 Ma (Meredith et al. 2011). Thus, the platypus genome, which recently became available (Warren et al. 2008), makes it possible to more precisely narrow down the time when the 13 ancient RNase gene lineages arose. Our search for RNase genes in the platypus genome (WUGSC5.0.1/ornAna1) yielded five functional genes (table 1). We then made a phylogenetic tree with the five functional platypus genes, 13 cow RNase genes, and three chicken RNase genes (Cho and Zhang 2006; Nitto et al. 2006) (fig. 2). The three chicken genes were chosen to represent the nonmammalian RNase A genes, and the 13 cow genes represent the 13 ancient gene lineages of the eutherian RNase A superfamily. We chose the 13 cow genes because the cow is the only species that has at least one functional gene for each of the 13 ancient gene lineages (Cho and Zhang 2006). Two different hypotheses, in regards to the time when the expansion of the ancient gene lineages occurred, would lead to two different phylogenetic patterns of how the platypus genes are related with the cow and chicken genes. First, if the expansion of the 13 ancient gene lineages occurred after the prototherian lineage (including the platypus) diverged from the therian (marsupial and placental mammals) lineage, the five platypus genes would form a species-specific clade and leave the cow genes outside, just like what the chicken genes would do (fig. 2*A*). Second, if the expansion occurred before the prototherian-therian split, the platypus genes would not form a species-specific clade and they would mix with the cow genes in the tree where some platypus genes are sister to some cow genes (fig. 2*B*). Our result, shown in figure 2*C*, supports the second hypothesis and suggests that the expansion of the 13 ancient gene lineages predates the platypus-therian divergence. Two platypus RNase genes, Oa-RNase4 and Oa-RNase13, are sister to Bt-RNase4 and Bt-RNase13, respectively, with strong bootstrap supports. Two other genes, Oa-RNase33 and Oa-RNase34, are recent duplicates that are 93% and 89% identical to each other in their nucleotide and amino acid sequences, respectively. The internal branch that diverge to these two genes (Oa-RNase33 and Oa-RNase34) and the branch of another gene (Oa-RNase35) are connected at the base of the tree, suggesting that they are ancient, platypus-specific genes, which are reminiscent of the ancient opossum-specific RNases reported by Cho and Zhang (2006). None of these three basal platypus genes are closely related with any of the opossum-specific genes or any other mammalian RNase genes (data not shown). Our results suggest that the expansion of the mammalian RNase A superfamily, which established the 13 ancient gene lineages, occurred in the common ancestor of all extant mammals before the divergence between the prototherian and the therian lineages (∼220 Ma). After this split, the platypus retained only two gene lineages, RNase4 and RNase13, and lost the other 11, whereas therian mammals lost the two platypus-specific gene lineages.

### A Wide Range of Number Variation among Eutherian Mammals Results from Differential Retention of the Ancient Gene Lineages

The RNase gene catalog of the 20 mammals summarized in table 1 reveals a wide range of variation in the total number of RNases among these species, ranging from 5 (platypus) to 34 (little brown bat). The variation becomes even greater if pseudogenes are also considered. A great diversity in gene number is still observed among the 18 eutherian mammals, ranging from 7 (dog) to 34 (little brown bat). A major factor for this gene number variation is that the ancient lineages are differentially retained in these species. Only five (RNase1, RNase4, RNase6, RNase10, and RNase12) of the 13 ancient gene lineages are present in all the 18 eutherian mammals. Other gene lineages were deleted or pseudogenized in one or more species. Interestingly, RNase9 is lost in the guinea pig but present in the other 17 eutherian mammals. For two reasons, we believe that the absence of RNase9 in the guinea pig is caused by a genuine gene loss, not by an incompleteness of the guinea pig genome database. First, the guinea pig genome has a relatively high coverage (6.69×, supplementary table S1, Supplementary Material online). Second, no sequencing gaps were found in the region between RNase10 and RNase11 in the guinea pig RNase cluster located in the middle of Scaffold 3 (data not shown). RNase10 and RNase11 are two neighboring genes of RNase9 in the other mammals.

Five other genes (RNase2/3, RNase5, RNase7/8, RNase11, and RNase13) are absent in two to five species. The absence of RNase2/3 in the naked mole rat and the guinea pig can be explained by a single gene loss event in the common ancestor of these Hystricognath rodents. Similarly, the absence of RNase2/3 in the dog and the giant panda is probably due to a single gene loss event in the common ancestor of the two Caniforme carnivores. Thus, at least three independent gene losses occurred for RNase2/3: one in the rodents, one in the carnivores, and one in the African elephant (table 1). Applying the same parsimonious reasoning, the absence of RNase5 in five species (gibbon, naked mole rat, guinea pig, dog, giant panda, and African elephant) and the absence of RNase7/8 in three species (mouse, rat, and dog) can be explained by at least four and two independent gene loss events, respectively. The absence of RNase11 in three species and that of RNase13 in two species are all independent, autaopmorphic events. Two RNase A genes (RNase14 and RNase15) are present only in the cow. They are lost or pseudogenized in all other species including the elephant, which is the most basal species among the 18 eutherians (Meredith et al. 2011). Therefore, massive, independent gene deletions, or pseudogenization events are necessary to explain this unusual pattern of global loss of the genes with their retention in a single, derived species (see Discussion).

### The Rate of Protein Sequence Evolution Varies Greatly among Different Gene Lineages, Suggesting that They Have Evolved under Different Types of Selection

**Fig. 3.—:**
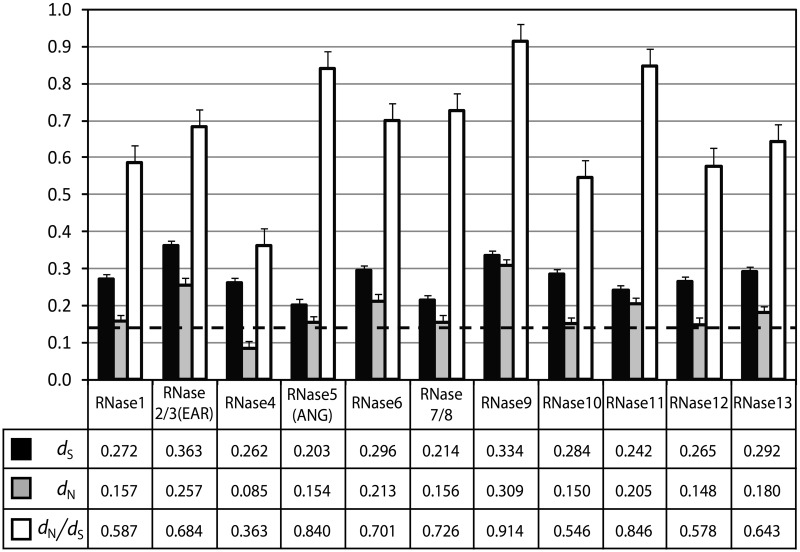
Synonymous (*d*_S_) and nonsynonymous (*d*_N_) nucleotide distances between all possible orthologous gene pairs of the 13 ancient RNase gene lineages. Black bars indicate *d*_S_, gray bars indicate *d*_N_, and white bars indicate *d*_N_/*d*_S_. The genome-wise average *d*_N_/*d*_S_ (0.144) of the six eutherian species measured by Groenen et al*.* (2012) is marked by the dashed horizontal line.

In table 1, we noticed that some genes, such as RNasae4, RNase10, and RNase12, are retained in all eutherian mammals and show no or a very low level of gene number variation among different species. However, some others, such as RNase2/3 and RNase5, are absent in some species and show a greater level of gene number variation. This suggests that different RNase gene lineages have evolved to have different physiological functions putting them under different selective regimes. The functions of RNase10 and RNase12 perhaps do not benefit from gene number increase. However, functional diversification of duplicated genes that have host-defense functions, such as RNase2/3 and RNase5 (Rosenberg 2008b), might be positively selected, reflected by their accelerated protein sequence evolution. To test this, we computed the synonymous (*d*_S_) and nonsynonymous (*d*_N_) nucleotide substitutions per site for 11 of the 13 ancient gene lineages by comparing all possible pairs of orthologous genes. We did not include RNase14 and RNase15 in this analysis because they are present only in the cow. If a species has multiple genes for a certain gene lineage, we randomly chose one of them. For example, we used 18 orthologous RNase1 genes from the 18 eutherian species, and we included only one of the three cow RNase1 genes. Our analysis is not affected by which one of the multiple cow RNase1 genes is chosen. This generates 153 pairs of orthologous RNase1 genes for this analysis. For RNase2/3, only 13 orthologous genes were used to generate 78 ortholog pairs because five eutherian species have lost RNase2/3. The average *d*_S_, *d*_N_, and *d*_N_/*d*_S_ values calculated from all possible ortholog pairs for each gene lineage are presented in figure 3 and supplementary table S3, Supplementary Material online. The average *d*_S_ value does not vary much among the RNase gene lineages, ranging from 0.203 to 0.363. These numbers are within the genome-wise *d*_S_ range (0.138–0.458) of the six eutherian species calculated by Groenen et al*.* (2012). However, the *d*_N_/*d*_S_ average varies greatly among the RNase gene lineages, ranging from 0.363 (RNase4) to 0.914 (RNase9), which suggests that substitutions that change protein sequence were fixed at different paces for the gene lineages during the eutherian evolution. Remarkably, even the lowest *d*_N_/*d*_S_ value (0.363) among the RNase gene lineages is by far greater than the genome-wise *d*_N_/*d*_S_ range (0.116–0.163 with the average of 0.144) of the six eutherian species (Groenen et al. 2012). These results suggest that these ancient RNase gene lineages have evolved under different selective regimes, reflecting diverse physiological functions. In addition, it appears that in general eutherian RNase genes have evolved with accelerated paces of amino acid changing substitutions, which can be caused by positive Darwinian selection, relaxation of function, or a combination of both.

To further characterize the evolution of each of these 11 RNase gene lineages, we plotted all the ortholog pairs with their *d*_S_ and *d*_N_ values (supplementary fig. S1, Supplementary Material online). Interestingly, the patterns of three gene lineages (RNase4, RNase10, and RNase12) that have lowest average *d*_N_/*d*_S_ values are distinct from those of the other gene lineages. More specifically, their *d*_N_ values reach to a plateau at *d*_S_ values of approximately 0.2. Such precocious saturation for nonsynonymous (or amino acid changing) substitutions suggests that the majority of the codons in these genes are under strong purifying selection and that their functions might be more essential than those of the other RNase gene lineages. The observation that these three genes are present in all 18 eutherian mammals supports this idea (table 1). Two other gene lineages (RNase1 and RNase13) that are next lowest in the *d*_N_/*d*_S_ averages (0.587 and 0.643, respectively) show a mild level of *d*_N_ saturation, starting at *d*_S_ = ∼0.3. The other six gene lineages (RNase2/3, RNase5, RNase6, RNase7/8, RNase9, and RNase11) that have relatively high *d*_N_/*d*_S_ averages do not show any significant saturation in their plots. These results corroborate our idea that different selective regimes have governed the evolution of these 11 RNase gene lineages, which suggest that they have undergone extensive functional diversification since they arose during early mammalian evolution. Five gene lineages, including RNase4, RNase10, RNase12, RNase1, and RNase13, appear to have evolved under more functional constraint than the other gene lineages.

### Episodic Bursts of Gene Duplication Further Diversify the RNase Gene Repertoires among the 18 Eutherian Mammals

**Fig. 4.—:**
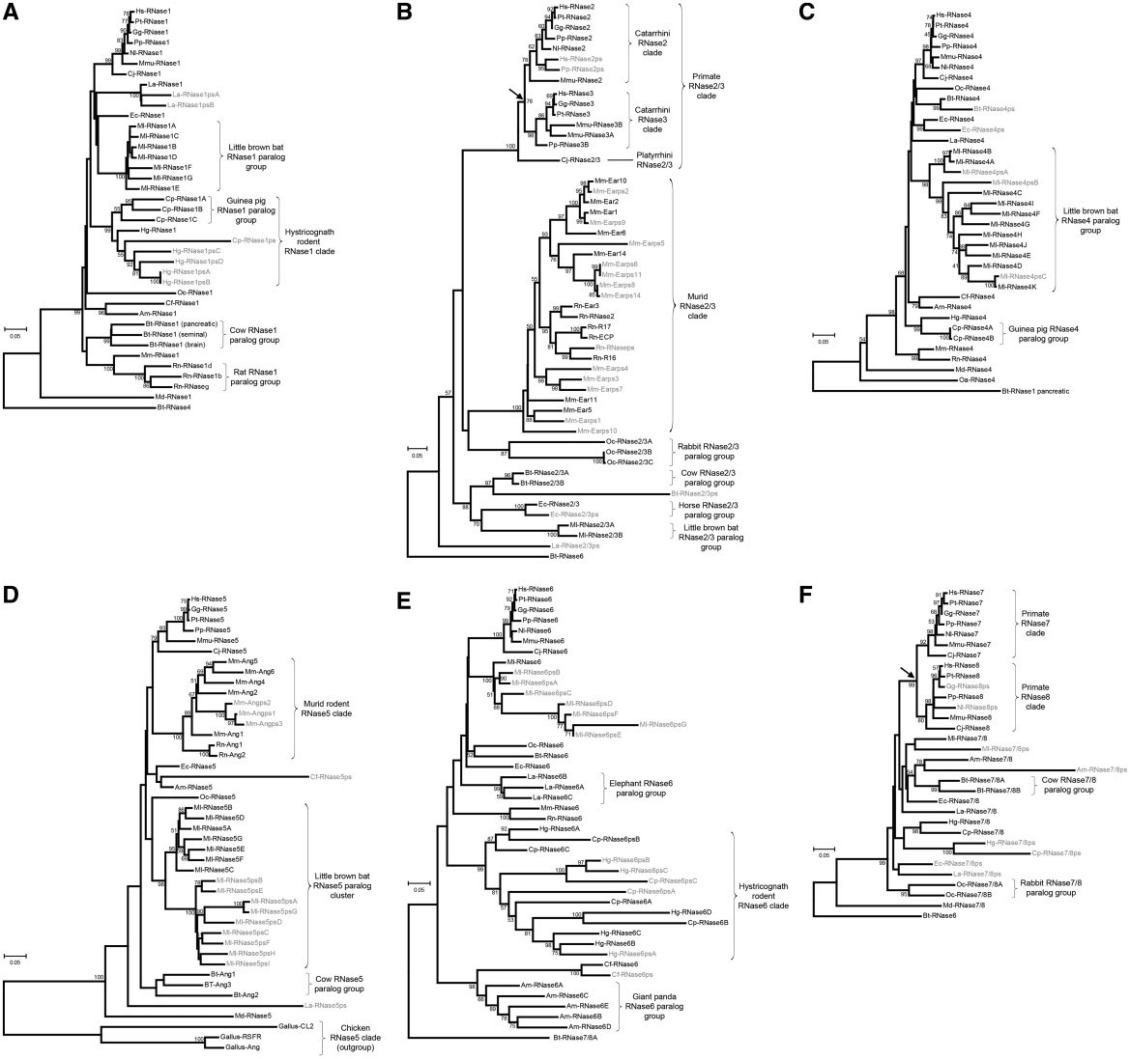
Phylogenetic trees of functional genes and pseudogenes of the six canonical RNase gene lineages. For all trees, the neighbor-joining method with Kimura’s two-parameter, the complete-deletion option, and 1,000 bootstrap replications were used. Trees for RNase1 (*A*), RNase2/3 (*B*), RNase4 (*C*), RNase5 (*D*), RNase6 (*E*), and RNase7/8 (*F*) were made with 454, 475, 471, 439, 501, and 483 nucleotide sites, respectively. Pseudogenes are distinguished with gray color. Paralog groups are marked with brackets. Arrows in (*B*) and (*F*) indicate the time of duplication of RNase2 and RNase3 (*B*) and that of RNase7 and RNase8 (*F*), respectively. The scale bar number indicates the number of nucleotide substitutions per site.

In addition to the differential retention of the 13 ancient lineages, episodic bursts of gene duplication that occurred in some species further increase the gene number variation among different species. To elucidate how these gene duplications occurred, we made gene trees for 6 canonical gene lineages (RNase1, RNase2/3, RNase4, RNase5, RNase6, and RNase7/8) using the nucleotide sequences of the genes and pseudogenes from all mammals (fig. 4). We excluded the five noncanonical RNases, because, except for two RNase9 genes in the rhesus macaque and also in the cow, we did not find any gene duplication events in any of the five noncanonical RNase genes (table 1). The gene trees of these five noncanonical RNases are presented in supplementary figure S2, Supplementary Material online. RNase14 and RNase15 were also excluded because they are present only in the cow as a solo.

Except for the platypus, all mammals have at least one functional RNase1 gene, and four species (rat, guinea pig, cow, and little brown bat) have multiple functional RNase1 genes (table 1 and fig. 4*A*). The species-specific duplication of RNase1 in the rat and the cow are previously studied (Zhao et al. 2001; Dubois et al. 2002; Wheeler et al. 2012). Three functional RNase1 genes and one pseudogene found in the guinea pig do not form a species-specific clade, although three functional genes form a clade. They are mixed with one functional RNase1 and four pseudogenes of the naked mole rat (fig. 4*A*). These two species belong to the same rodent infraorder Hystricognathi. Thus, this pattern suggests that some of the gene duplication events occurred before the divergence of the two species. Two of the three guinea pig RNase1 genes have been previously reported (van den Berg et al. 1977). The African elephant has one functional RNase1 gene and two pseudogenes, just like the Indian elephant (*Elephas maximus*) (Dubois et al. 2003). The RNase1 proteins of the two elephant species differ by only one amino acid, whereas their pseudogenes differ more (data not shown). The little brown bat (Ml) has seven functional RNase1 genes generated by bursts of species-specific gene duplication events, which will be discussed later in Discussion.

The gene tree of all mammalian RNase2/3 genes (fig. 4*B*) and that of only primate RNase2/3 genes (supplementary fig. S3*A*, Supplementary Material online) both confirm two previously reported features of this gene lineage. First, as shown by Rosenberg et al*.* (1995), the duplication of RNase2 and RNase3 occurred in the common ancestor of all Catarrhini (old-world monkeys and apes) primates after it split from the Platyrrhini (new-world monkeys) lineage (between 29 and 43 Ma) (Kumar and Hedges 2011). Second, the mouse (Mm) and the rat (Rn) have numerous RNase2/3 genes and pseudogenes, also known as eosinophil-associated RNases (EARs) (Cho et al. 2005; Cho and Zhang 2006). Four other species, including the rhesus macaque (Mmu), the rabbit (Oc), the cow (Bt), and the little brown bat (Ml), have multiple RNase2/3 genes, all of which were generated by species-specific gene duplications.

In contrast with the dynamic nature of the RNase2/3 evolution, the evolutionary history of RNase4 is more static. All the 20 mammals have at least one functional RNase4, and all of them, except for the guinea pig (Cp) and the little brown bat (Ml), have exactly one functional RNase4 gene (table 1 and fig. 4*C*). The two guinea pig RNase4 genes differ by only one nucleotide, suggesting they were duplicated very recently. Strikingly, the little brown bat has 11 functional RNase4 genes and three pseudogenes, generated by species-specific bursts of gene duplication. The three pseudogenes do not form a clade, suggesting that they were inactivated by three independent pseudogenization events.

Four species, including the mouse, the rat, the cow, and the little brown bat, have multiple RNase5 genes (table 1). The gene tree in figure 4*D* confirms the species-specific clades of the RNase5 genes from the mouse (Mm), the rat (Rn), and the cow (Bt), which have been reported in previous studies (Brown et al. 1995; Cho et al. 2005). We found that the little brown bat (Ml) also underwent a massive expansion that gave rise to seven functional genes and nine pseudogenes. These nine pseudogenes form a clade with a solid bootstrap support (100%), suggesting that they were generated by duplications of a single ancestral pseudogene.

Multiple RNase6 genes are present in four species, including the naked mole rat, the guinea pig, the giant panda, and the African elephant (table 1). As shown in the gene tree (fig. 4*E*), the five RNase6 genes of the giant panda (Am) and the three RNase6 genes of the African elephant (La) form species-specific clades. However, seven functional genes and five pseudogenes of the two Hystricognath rodents, the naked mole rat (Hg) and the guinea pig (Cp), do not form species-specific clades. Gene and pseudogenes from these two species get mixed in this Hystricognath RNase6 clade. Thus, multiple gene duplications occurred before the divergence between the naked mole rat lineage and the guinea pig lineage. Later on, species-specific gene duplications, deletions, and pseudogenizations caused these two species to have distinct RNase6 gene repertoires. This phenomenon is a hallmark of the gene sorting process that has been reported previously in some RNases with host-defense functions such as Murid RNase2/3 (or EAR) and RNase5 (or Ang) (Zhang et al. 2000; Cho et al. 2005).

Previous studies showed that only primates have two distinct genes (RNase7 and RNase8), whereas nonprimate mammals have only one gene that are equally related with the primate RNase7 and RNase8 (Zhang et al. 2002; Cho and Zhang 2006; Zhang 2007). This suggests that the duplication of RNase7 and RNase8 occurred during the early primate evolution, but the exact time has not been dated. Our gene tree in figure 4*F* shows that the gene duplication of RNase7 and RNase8 occurred in the common ancestor of all simian primates, which include Catarrhini (old-world monkeys and apes) and Platyrrhini (new-world monkeys) primates. To further narrow it down, we collected additional RNase7/8-related sequences from the low-coverage genomes of two prosimian primates, the tarsier (*Tarsius syrichta*) and the bush baby (*Otolemur garnettii*). The prosimians (tarsier and lemurs) are more basal than the simians in the primate phylogeny (Kumar and Hedges 2011). The gene tree of both simian and prosimian RNase7/8 sequences shown in supplementary figure S3*B*, Supplementary Material online, clearly indicates that the duplication occurred in the ancestral simian lineage shortly after it diverged from the prosimians (43–65 Ma) (Kumar and Hedges 2011). Independent of this primate duplication, a species-specific duplication of RNase7/8 occurred in the rabbit (Oc) and also in the cow (Bt).

### Episodic Bursts of Gene Duplications Followed by Positive Selection Are a Common Feature of the Eutherian RNase Evolution

After a gene duplication, two duplicated genes can undergo differential amino acid changes and become functionally distinct, which is often driven by positive selection. An elevated level of amino acid changing substitutions can reflect such adaptive functional diversification between two paralogous genes (Innan and Kondrashov 2010; Kondrashov 2012; Magadum et al. 2013). From the entire RNase gene repertoires of the 18 eutherian species that we studied here, we identified 22 paralog groups that have at least two functional paralogs that resulted from one or more species-specific duplication events (table 2). For example, there are four paralog groups identified for RNase1, one each for the rat, the guinea pig, the cow, and the little brown bat (fig. 4*A*). To test whether these paralogs have undergone functional diversification driven by positive selection, we calculated the average *d*_N_/*d*_S_ value of all possible paralog pairs for each of these 22 paralog groups (table 2). These average *d*_N_/*d*_S_ values vary greatly ranging from 0.326 (for the rat RNase5) to 2.105 (for the rhesus monkey RNase9), and 11 paralog groups have average *d*_N_/*d*_S_ values higher than 1. A great variation of *d*_N_/*d*_S_ values among different paralog groups suggests that different selective regimes have governed the evolution of different paralog groups. Furthermore, a great variation exists among different paralog groups within the same gene lineage. For example, the *d*_N_/*d*_S_ values for RNase2/3 range from 0.383 (rat) to 1.310 (mouse). These results indicate that paralogs of the same gene arising in different species can evolve under different types of selection. Three paralog groups, including the mouse (Mm) EAR group, the little brown bat (Ml) RNase4 group, and the little brown bat (Ml) RNase5 group, have *d*_N_/*d*_S_ values greater than 1 with statistical significance, (table 2). The positive selection of the mouse EAR group has been studied previously (Zhang et al. 2000; Cho et al. 2005), and we will discuss on the little brown bat paralog groups later in this article.

**Table 2:** The List of 22 Paralog Groups Identified in This Study and the Numbers of Codon-Based Substitutions per Site Calculated for Each Group

Paralog Group	*N*^a^	Ave. *d*_S_ (SE)	Ave. *d*_N_ (SE)	Ave. *d*_N_/*d*_S_	*P*^b^	*d*_N_/*d*_S_ > 1^c^
Rn-RNase1	3	0.122 (0.023)	0.113 (0.015)	0.939	NS	1/3
Cp-RNase1	3	0.174 (0.027)	0.128 (0.017)	0.750	NS	0/3
Bt-RNase1	3	0.204 (0.028)	0.104 (0.016)	0.514	1.00 × 10^−4^	0/3
Ml-RNase1	7	0.055 (0.012)	0.056 (0.008)	0.997	NS	12/21
**Mm-EAR**	**7**	**0.144 (0.018)**	**0.189 (0.016)**	**1.310**	**0.0151**	**18**/**21**
Rn-EAR	5	0.141 (0.021)	0.125 (0.015)	0.383	NS	2/10
Oc-RNase2/3	3	0.287 (0.030)	0.163 (0.018)	0.568	NS	0/3
Ml-RNase2/3	2	0.093 (0.025)	0.066 (0.015)	0.710	—	0/1
Cp-RNase4^d^	2	0	0.003 (0.003)	∞	—	0/1
**Ml-RNase4**	**11**	**0.125 (0.016)**	**0.160 (0.015)**	**1.343**	**1.45** × **10**^−^**^6^**	**44**/**55**
Mm-RNase5	5	0.136 (0.020)	0.145 (0.015)	1.093	NS	5/10
Rn-RNase5	2	0.043 (0.017)	0.014 (0.007)	0.326	—	0/1
Bt-RNase5	3	0.220 (0.029)	0.210 (0.021)	0.949	NS	0/3
**Ml-RNase5**	**7**	**0.053 (0.012)**	**0.091 (0.013)**	**2.002**	**1.21** × **10**^−^**^7^**	**20**/**21**
Hg-RNase6	4	0.308 (0.028)	0.303 (0.023)	0.979	NS	3/6
Cp-RNase6	3	0.435 (0.035)	0.343 (0.025)	0.783	NS	0/3
Am-RNase6	5	0.220 (0.024)	0.227 (0.017)	1.048	NS	5/10
La-RNase6	3	0.127 (0.025)	0.124 (0.018)	0.985	NS	1/3
Oc-RNase7/8	2	0.271 (0.041)	0.146 (0.022)	0.539	—	0/1
Bt-RNase7/8	2	0.135 (0.029)	0.107 (0.019)	0.796	—	0/1
Mmu-RNase9	2	0.019 (0.011)	0.040 (0.010)	2.105	—	1/1
Bt-RNase9	2	0.242 (0.036)	0.222 (0.023)	0.917	—	0/1

Note.—Average *d*_S_ (numbers of synonymous substitutions per synonymous sites), *d*_N_ (numbers of nonsynonymous substitutions per nonsynonymous sites), and *d*_N_/*d*_S_ values of all possible paralog pairs for each group were measured. Standard errors (SE) are shown in parentheses. NS, not significant. *P* value higher than 0.05. —: Not applicable. We did not perform statistical tests for groups with only two paralogs (i.e*.*, one paralog pair). The paralog groups with average *d*_N_/*d*_S_ value higher than one with statistical significance are bolded.

^a^Number of functional paralogs in each group.

^b^*P* values of the one-tail *t*-test for difference between *d*_N_ and *d*_S_, only applied to groups of three or more paralogs.

^c^Number of paralog pairs with *d*_N_/*d*_S_ > 1 out of the total number of paralog pairs.

^d^The paralog group of Cp-RNase4 has two recently duplicated genes different by only one nucleotide that also cause an amino acid difference.

### Bursts of Recent Gene Duplications Occurred for Four RNase Genes in the Little Brown Bat

**Fig. 5.—:**
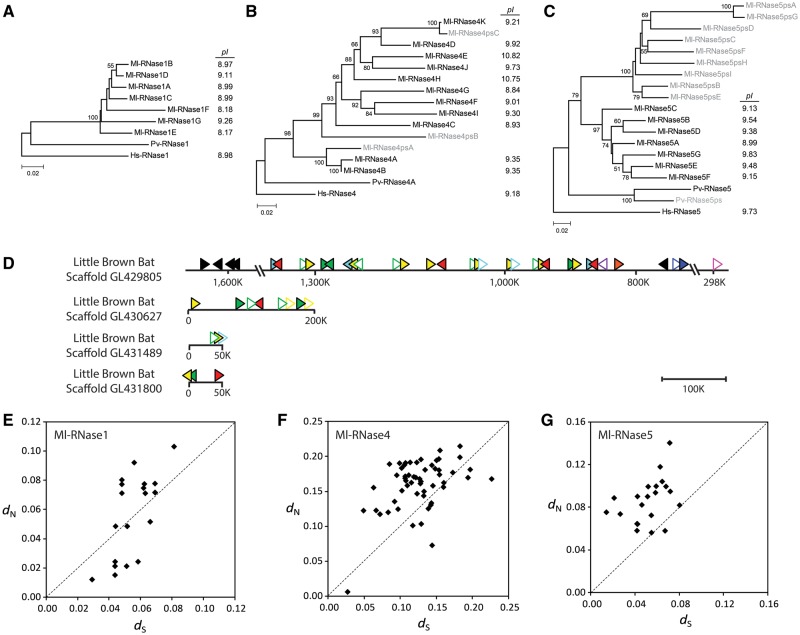
Phylogenetic trees of the RNase1 (*A*), RNase4 (*B*), and RNase5 (*C*) genes of the littler brown bat (Ml) and the flying fox (Pv, *Pteropus vampyrus*). The human (Hs) genes were used to root the tree. The isoelectric points of the human and little brown bat proteins are shown next to the functional genes in three tree. (*D*) Scaffold maps of the little brown bat RNase genes drawn to scale. We followed the same labeling system as figure 1. Pairwise synonymous (*d*_S_) and nonsynonymous (*d*_N_) nucleotide distances between paralogous RNase1 (*E*), RNase4 (*F*), and RNase5 (*G*) genes in the little brown bat. The scale bar number indicates the number of nucleotide substitutions per site.

Our search for RNase A genes in the little brown bat genome yielded 34 genes and 22 pseudogenes, which are surprisingly high numbers considering that the second most gene-abundant species (mouse) has 20 genes and 15 pseudogenes (table 1). We found seven RNase1 genes, 11 RNase4 genes (with three pseudogenes), seven RNase5 genes (with nine pseudogenes), and one RNase6 gene (with seven pseudogenes) in the genome of the little brown bat. To determine when these duplications occurred during the evolution of the order Chiroptera (the bats), we searched for these four RNase genes in the low-coverage (2×) genome of *Pteropus vampyrus*, the large flying fox, which belongs to Pteropodidae, a bat family different from that of the little brown bat (Vespertilionidae). We identified only one functional gene for each of RNase1, RNase4, RNase5, and RNase6 in the large flying fox genome. Furthermore, for all these four RNases, the little brown bat genes form species-specific clades leaving the large flying fox gene outside (fig. 5*A*–*C* and supplementary fig. S4, Supplementary Material online). Therefore, the bursts of duplication for these four RNases occurred in the little brown bat lineage after it diverges from the large flying fox approximately 60 Ma (Kumar and Hedges 2011).

We noted that these four RNase genes (RNase1, RNase4, RNase5, and RNase6) neighbor one another in the mammalian consensus gene order shown in figure 1, raising a possibility that a large block that includes these four genes were duplicated several times leading to simultaneous expansions of these four genes during the evolution of the little brown bat. However, we were not able to test this hypothesis with rigor, because the current assembly of the little brown bat genome is not in high quality, fragmented into scaffolds. A cluster of these four RNase genes, one for each, is present at around coordinate 900,000 of scaffold GL429805, the longest among the three scaffolds, in the consensus gene order (RNase5-RNase4-RNase6-RNase1). Except for this single case, the other genes and pseudogenes of these four RNases are mixed on multiple scaffolds without any repeatable gene order patterns (fig. 5*D* and supplementary table S2, Supplementary Material online). This suggests that repeated duplication of a large block is perhaps not a main mechanism by which the expansion of the four RNase genes occurred.

Next, we tested whether the expansions of the little brown bat RNase1, RNase4, and RNase5 genes were followed by positive selection for amino acid changes, which can reflect their adaptive roles. We did not include RNase6 in this test because its expansion generated eight pseudogenes and only one functional gene. As shown in table 2 and figure 5*E*–*G*, the average *d*_N_/*d*_S_ values of the RNase1, RNase4, and RNase5 paralog groups of the little brown bat are highly elevated (0.997, 1.343, and 2.002, respectively). The *d*_N_/*d*_S_ ratio is higher than one for 12 of the 21 RNase1 paralogous pairs, 44 of the 55 RNase4 paralogous pairs, and 20 of the 21 RNase5 paralogous pairs. These results indicate that the evolution of these paralogs have been driven by positive selection for protein sequence changes.

After the duplication of RNase2/EDN and RNase3/ECP, the isoelectric point (pI) of RNase3/ECP (10.47) was elevated significantly compared with that of RNase2/EDN (9.20), which was caused by accumulation of positively charged amino acids such as arginine and lysine (Rosenberg and Dyer 1995). These positively charged amino acids are important for the antibacterial activities of RNase3/ECP, enabling it to bind to bacterial cell wall (Torrent et al. 2008). Interestingly, similar pI increase occurred for four of the little brown bat RNase4 proteins, including RNase4D, RNase4E, RNase4H, and RNase4J, reminiscent of the primate RNase3/ECP (fig. 5*B*). Much like what RNase3/ECP underwent since it separated from RNase2/EDN, these four bat proteins accumulated numerous substitutions for positively charged amino acids during their evolution as compared with the other bat RNase4 proteins and the human RNase4 (supplementary table S4 and fig. S5, Supplementary Material online).

## Discussion

Our phylogenetic analyses of the five platypus RNases along with the cow and the chicken RNases show that the 13 ancient RNase gene lineages arose during the very early stage of the mammalian evolution predating the divergence of the therian and prototherian lineages (∼220 Ma). Two platypus RNases (RNase4 and RNase13) are sister to the cow RNase4 and RNase13, respectively, with strong bootstrap supports, whereas the other three are platypus-specific (fig. 2*C*). This pattern suggests that most of the ancient lineages, except for RNase4 and RNase13, were lost during the platypus evolution. Or, one could raise another possibility that only RNase4 and RNase13 arose before the therian-prototherian divergence and a later expansion in the therian lineage established the other 11 ancient gene lineages. However, if such a scenario were true, the 11 cow genes, excluding RNase4 and RNase13, would form a clade with the RNase4 and RNase13 genes of the cow and the platypus left outside the clade. We did not see this in figure 2*C*, but instead the divergence between the cow RNase4 and the platypus RNase4 (and also between the cow RNase13 and the platypus RNase13) appears to be a relatively recent event with short external branches, whereas the divergences of the 13 ancient gene lineages are deep in the tree, reflecting their ancient origin. Unfortunately, there are no extant mammals that are more basal than the platypus, thus we are not able to further narrow the time range of this expansion. The RNase sequences of the echidna, another monotreme, would help corroborate our conclusion.

**Fig. 6.—:**
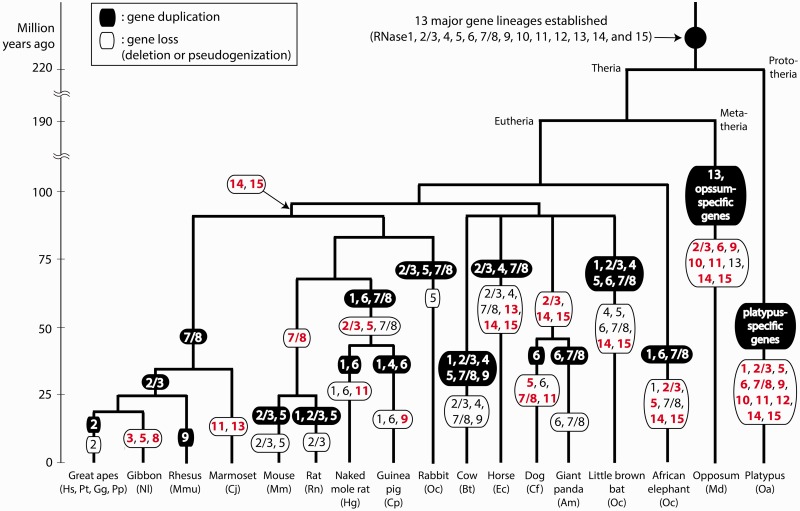
Evolutionary history of the mammalian RNase A superfamily. Gene duplication and gene loss events are mapped on the branches of the phylogeny of the 20 mammals studied here. Permanent losses of gene lineages are labeled red and bold, whereas partial losses are black and nonbold. The phylogeny and divergence times followed Meredith et al*.* (2011).

Two main factors are responsible for a great variation in total RNase counts observed among the 20 mammals. First, some of the 13 ancient gene lineages were lost in some species, whereas some other lineages are kept in all species. Second, recent bursts of gene duplication increased the numbers of genes for certain gene lineages in a species-specific manner. To illustrate these factors more clearly, we generated a diagram that maps the gene duplication and loss events of the RNase gene lineages on the mammalian phylogeny (fig. 6). The locations of those events on the tree were determined by analyzing the gene genealogy of each gene lineage shown in figure 4. This diagram shows the evident role of differential gene duplication and loss events causing a great variation in the RNase gene counts among these species.

In our study, we identified 22 paralog groups that were generated by recent gene duplications (table 2). Surprisingly, the rate of protein sequence evolution, measured by the average *d*_N_/*d*_S_ value of all possible paralog pairs within each group, varies greatly among different paralog groups. A great variation exists even among different paralog groups of the same gene lineage present in different species. This suggests that the same RNase can have different functions thus evolve under different selections in different species. For example, functional requirements for a host-defense gene can be different among different species because they are in different pathogen environments. It also implies that RNases can evolve with great flexibility in terms of the range of functions they can acquire.

Bursts of gene duplications followed by differential gene retentions among different species, also known as gene sorting, is a hallmark of the evolution of host-defense genes (Zhang et al. 2000). It has been observed for the rodent RNase2/3 (also known as EAR) and RNase5 (also known as angiogenin) genes, which have antibacterial and antiviral activities (Gupta et al. 2013). We observed a similar gene sorting process in the RNase6 genes of the naked mole rat and the guinea pig (fig. 4*E*), implying their roles in host defense. Interestingly, all the four species (naked mole rat, guinea pig, giant panda, and African elephant) with multiple RNase6 genes have lost their RNase2/3, and three of them (naked mole rat, guinea pig, and African elephant) have lost their RNase5 too. No other mammals have multiple RNase6 genes except for these four species. From this observation, we propose that RNase6 in these four species acquired host-defense functions similar to what RNase2/3 and RNase5 have in other species. It would be interesting to test experimentally if RNase6 proteins in these four species have such host-defense activities. In human, RNase6 is expressed in monocytes and neutrophils (Rosenberg and Dyer 1996), but otherwise it has not been associated with host-defense functions.

The RNase genes involved in host defense are known to have frequent gene-birth-and-death events and show elevated *d*_N_/*d*_S_ values driven by positive selection for new functions (Zhang et al. 2000; Cho et al. 2005). It is believed that frequent gene turn-over and fast protein sequence changes of host-defense genes give selective advantages to the host species in coping with fast-evolving pathogens (Rosenberg 2008b). In line with this view, RNase2/3, RNase5, RNase6, and RNase7/8, which have been associated with host-defense functions in previous studies (Rosenberg 2008b; Gupta et al. 2013) and here, show most a high gene number variability (table 1 and fig. 6) and have relatively high *d*_N_/*d*_S_ values between orthologs (fig. 3). On the other hand, other genes, such as RNase4, RNase10, and RNase12, show a low gene number variability and relatively low *d*_N_/*d*_S_ values, which probably have more essential functions that do not benefit from gene number increase. The mouse RNase10 is known to be involved in sperm maturation (Krutskikh et al. 2012). The physiological functions of RNase4 and RNase12 are currently unknown. One exception to this correlation between the gene number variability and the rate of protein sequence evolution is RNase9. It has the highest orthologous *d*_N_/*d*_S_ values among the ancient RNase gene lineages (fig. 3 and supplementary table S3, Supplementary Material online), but its number does not vary much among different species (table 1). A recent study showed that recombinant human RNase9 has antibacterial activities, suggesting its host-defense role in the male reproductive tract (Cheng et al. 2009). It is possible that RNase9 also have other functions that are sensitive to gene dose change.

Cho and Zhang (2006) previously identified RNase14 and RNase15 in the cow genome but not in any of the five other mammals they studied. It was puzzling that these two genes appeared to be ancient, reflected by their basal positions in the RNase phylogeny. An alternative possibility that they arose by recent cow-specific gene duplications is not likely (Cho and Zhang 2006 and fig. 2*C*). This study confirms that these two genes are indeed cow specific, not present in any of the 19 other mammals. In addition, we identified 14 RNase14 pseudogenes and 10 RNase15 pseudogenes in a wide range of taxa including seven primates, one rodent, the horse, the giant panda, the little brown bat, and the elephant. This suggests that the common ancestor of all eutherian mammals had functional RNase14 and RNase15 genes but only cow retained their functions, whereas they became lost or pseudogenized in all other mammals. The cow is not a basal mammalian species, thus it requires multiple independent gene loss events to explain their unique retention in this derived species. For RNase14, the NCBI database has one mRNA entry (accession number BC111643) and two EST entries (DT856887 and DR712558) originated from the testis and one EST entry (DT830385) from the liver. For RNase15, two EST entries (CR849599 and CR853111) from the caruncular tissues are present in the database. These entries confirm expression of these genes in the cow, but their physiological functions are currently not known. A previous expression study conducted by Wheeler et al*.* (2012) detected a weak expression of RNase15 in the cow endometrium, and no RNase14 expression was detected in any of the seven cow tissues they tested.

The most dramatic cases of recent species-specific expansion of RNase gene lineages are found in the little brown bat, which experienced bursts of gene duplication for RNase1, RNase4, and RNase5. The evolution of the paralogs generated by these duplications has been driven by positive selection (fig. 5). These observations raise a hypothesis that the paralogs of RNase1, RNase4, and RNase5 have host-defense functions in the little brown bat. It is known that the little brown bats have a communal night roosting behavior (Barclay 1982). They cluster together in a large colony to stay warm at night. In particular, communal night roosting serves a thermoregulatory role for pregnant females, which must maintain a high body temperature to promote rapid embryo development. Furthermore, it is also a vital factor in the survival of young bats over their first winter. As a trade-off, their crowded lifestyle made them more vulnerable to contagious pathogens (Scott and Duszynski 1997). A recent outbreak of white-nose syndrome, a fungal disease, which caused local extinctions of the little brown bat populations in North America, was facilitated by their communal roosting behavior (Frick et al. 2010). Therefore, we hypothesize that RNase1, RNase4, and RNase5 have host-defense functions in the little brown bat, and their expansion and functional diversification contributed to the species’ adaptation to communal roosting. In line with this hypothesis, the large flying fox (*P. vampyrus*), which does not have such communal roosting behavior, has just one gene for RNase1, RNase4, and RNase5 (supplementary fig. S4, Supplementary Material online). It would be interesting to study RNase genes of other microbat species to test whether the bursts of duplication of these three RNase genes are associated with the origin of the communal roosting behavior.

As discussed earlier, RNase4 is among the least variable in gene number (table 1), and it is also most conserved gene in terms of the rate of protein sequence evolution (fig. 3). Thus, it is striking that the little brown bat has 11 functional RNase4 genes that evolved under strong positive selection (table 2 and fig. 5*C*). Furthermore, four of the 11 RNase4 genes encode proteins with an elevated isoelectric point and numerous substitutions for positive amino acids (supplementary table S4 and fig. S5, Supplementary Material online), which are the hallmarks of antibacterial activities. These observations raise an intriguing hypothesis that at least some of the little brown bat RNase4 genes switched to host-defense functions after gene duplication. Dubbed as neofunctionalization, one of the two duplicated genes can be released from functional constraint and acquire new functions, whereas the other gene performs the original functions (Innan and Kondrashov 2010). It would be interesting to further investigate whether this “functional switch” occurred in duplicated RNase4 genes, which would require studying the physiological functions of the RNase4 genes in the little brown bat and other related bat species.

While this article was being prepared, two studies reported the expansion of the microbat RNase genes. First, Zhang et al. (2013) reported their identification of seven full-length sequences and two partial sequences of RNase4 in David’s Myotis (*Myo. davidii*), an Old-World *Myotis* species, suggesting that expansion of RNase4 started in the common ancestor of the New-World and the Old-World *Myotis* species. To test this, we made a phylogenetic tree of our 11 functional RNase4 proteins in *Myo. lucifugus* with the seven full-length *Myo. davidii* RNase4 proteins identified by Zhang et al*.* (supplementary fig. S6*B*, Supplementary Material online). In the tree, the RNase4 proteins from the new-world species (*Myo. lucifugus*) do not form a species-specific cluster, but they are mixed with the RNase4 proteins from the old-world species (*Myo. davidii*), which supports the idea that the expansion of RNase4 started before the divergence of the two species. More recently, Xu et al*.* (2013) reported their identification of nine RNase1 genes in the little brown bat using PCR amplification. Surprisingly, only two of their nine RNase1 genes are identical to two of the seven that we report in this article at the nucleotide sequence level. Their RNase1alpha2 is identical to our Ml-RNase1D, and their RNase1gamma4 to our Ml-RNase1F. This partial overlap suggests that the actual total number of the RNase1 genes in the little brown bat can be as many as 14. However, at the protein sequence level, their RNase1alpha1 is identical to our Ml-RNase1B, and their RNase1gamma5 is identical to our Ml-RNase1G. The phylogenetic tree that includes the *Myo. lucifugus* RNase4 proteins identified in both our study and in the work of Xu et al. is presented in supplementary figure S6*A*, Supplementary Material online. It would require more rigorous gene sequencing of multiple individuals of both species to determine whether these synonymous differences are due to polymorphisms or very recent gene duplication.

## Supplementary Material

Supplementary data set, figures S1–S6, and tables S1–S4 are available at *Genome Biology and Evolution* online (http://www.gbe.oxfordjournals.org/).

Supplementary Data
