# Supplementary material for: The Expansion and Functional Diversification of the Mammalian Ribonuclease A Superfamily Epitomizes the Efficiency of Multigene Families at Generating Biological Novelty
Source: Genome Biol Evol. 2013 Oct 25;5(11):2124–40. doi: 10.1093/gbe/evt161 (PMC3845642; doi:10.1093/gbe/evt161)
Supplement: Supplementary Data [file supp_5_11_2124__index.html]

The expansion and functional diversification of the mammalian ribonuclease A superfamily epitomizes the efficiency of multi-gene families at generating biological novelty — The Expansion and Functional Diversification of the Mammalian Ribonuclease A Superfamily Epitomizes the Efficiency of Multigene Families at Generating Biological Novelty — Supplementary Data 

# The Expansion and Functional Diversification of the Mammalian Ribonuclease A Superfamily Epitomizes the Efficiency of Multigene Families at Generating Biological Novelty

## Supplementary Data

files

**Files in this Data Supplement:**

- Supplementary Data - pdf file
- Supplementary Data - pdf file
