# Supplementary material for: The Expansion and Functional Diversification of the Mammalian Ribonuclease A Superfamily Epitomizes the Efficiency of Multigene Families at Generating Biological Novelty
Source: Genome Biol Evol. 2013 Oct 25;5(11):2124–40. doi: 10.1093/gbe/evt161 (PMC3845642; doi:10.1093/gbe/evt161)
Supplement: Supplementary Data [file supp_evt161_Supplementary_Dataset.pdf]

Supplementary Dataset. The nucleotide and protein sequences of all RNases used in this study. Nucleotide sequences are presented first, followed by protein sequences.

NUCLEOTIDE SEQUENCES

Human (*Homo sapiens*)

```
>Hs-RNase1
ATGGCTCTGGAGAAGTCTCTTGTCCGGCTCCTTCTGCTTGTCCTGATACTGCTGGTGCTGGGCTGGGTCCAGCCTTCCCTGGGCAAGGA
ATCCCGGGCCAAGAAATTCCAGCGGCAGCATATGGACTCAGACAGTTCCCCCAGCAGCAGCTCCACCTACTGTAACCAAATGATGAGGC
GCCGGAATATGACACAGGGGCGGTGCAAACCAGTGAACACCTTTGTGCACGAGCCCCTGGTAGATGTCCAGAATGTCTGTTTCCAGGAA
AAGGTCACCTGCAAGAACGGGCAGGGCAACTGCTACAAGAGCAACTCCAGCATGCACATCACAGACTGCCGCCTGACAAACGGCTCCAG
GTACCCCAACTGTGCATACCGGACCAGCCCGAAGGAGAGACACATCATTGTGGCCTGTGAAGGGAGCCCATATGTGCCAGTCCACTTTG
ATGCTTCTGTGGAGGACTCTACCTAA

>Hs-RNase2
ATGGTTCCAAAACTGTTCACTTCCCAAATTTGTCTGCTTCTTCTGTTGGGGCTTCTGGCTGTGGAGGGCTCACTCCATGTCAAACCTCC
ACAGTTTACCTGGGCTCAATGGTTTGAAACCCAGCACATCAATATGACCTCCCAGCAATGCACCAATGCAATGCAGGTCATTAACAATT
ATCAACGGCGATGCAAAAACCAAAATACTTTCCTTCTTACAACTTTTGCTAACGTAGTTAATGTTTGTGGTAACCCAAATATGACCTGT
CCTAGTAACAAAACTCGCAAAAATTGTCACCACAGTGGAAGCCAGGTGCCTTTAATCCACTGTAACCTCACAACTCCAAGTCCACAGAA
TATTTCAAACTGCAGGTATGCGCAGACACCAGCAAACATGTTCTATATAGTTGCATGTGACAACAGAGATCAACGACGAGACCCTCCAC
AGTATCCGGTGGTTCCAGTTCACCTGGATAGAATCATCTAA

>Hs-RNase2ps
ATGGTTCCAAAACTGTTCACTTCCCAAATTTGTCTGCTTCTTCTGTTGGGGCTTCTGGCTGTGGAGGGCTCACTCCATGTCAAACCTCC
ACAGTTTACCTGGGCTCAGGGGTTTGACGTCCATCAGTAGGAACCAGCTCCAATGCACCAATGCAATGCGGGTAATTAACAATTATCAA
CGGCGATGGAAAAACCAAAATACTTTTCTTCTTGCAACTTTTGCTAATGTAGTTAATGTTTGTGGTAACCCAACTATAACCTGCCCTCA
TAACAGAACTCTCAACAATTGTCATCATAGTGGAGTCCAGGTGCCTTTAATGTACTGTAACCTCACAACTCCAAGTCCACAGAATATTT
CAAACTGCAGGTATGCGCAGACACCAGCAAACATGTTCTATATAGTTGCATGTGACAACAGGGATCAACGACGGGACCCTCCACAGTAT
CCAGTGGTTCCAGTTCACCTGGATACCATCATCTAA

>Hs-RNase3
ATGGTTCCAAAACTGTTCACTTCCCAAATTTGTCTGCTTCTTCTGTTGGGGCTTATGGGTGTGGAGGGCTCACTCCATGCCAGACCCCC
ACAGTTTACGAGGGCTCAGTGGTTTGCCATCCAGCACATCAGTCTGAACCCCCCTCGATGCACCATTGCAATGCGGGCAATTAACAATT
ATCGATGGCGTTGCAAAAACCAAAATACTTTTCTTCGTACAACTTTTGCTAATGTAGTTAATGTTTGTGGTAACCAAAGTATACGCTGC
CCTCATAACAGAACTCTCAACAATTGTCATCGGAGTAGATTCCGGGTGCCTTTACTCCACTGTGACCTCATAAATCCAGGTGCACAGAA
TATTTCAAACTGCACGTATGCAGACAGACCAGGAAGGAGGTTCTATGTAGTTGCATGTGACAACAGAGATCCACGGGATTCTCCACGGT
ATCCTGTGGTTCCAGTTCACCTGGATACCACCATCTAA

>Hs-RNase4
ATGGCTCTGCAGAGGACCCATTCATTGCTTCTGCTTTTGCTGCTGACCCTGCTGGGGCTGGGGCTGGTCCAGCCCTCCTATGGCCAGGA
TGGCATGTACCAGCGATTCCTGCGGCAACACGTGCACCCTGAGGAGACAGGTGGCAGTGATCGCTACTGCAACTTGATGATGCAAAGAC
GGAAGATGACTTTGTATCACTGCAAGCGCTTCAACACCTTCATCCATGAAGATATCTGGAACATTCGTAGTATCTGCAGCACCACCAAT
ATCCAATGCAAGAACGGCAAGATGAACTGCCATGAGGGTGTAGTGAAGGTCACAGATTGCAGGGACACAGGAAGTTCCAGGGCACCCAA
CTGCAGATATCGGGCCATAGCGAGCACTAGACGTGTTGTCATTGCCTGTGAGGGTAACCCACAGGTGCCTGTGCACTTTGACGGTTAG

>Hs-RNase5
ATGGTGATGGGCCTGGGCGTTTTGTTGTTGGTCTTCGTGCTGGGTCTGGGTCTGACCCCACCGACCCTGGCTCAGGATAACTCCAGGTA
CACACACTTCCTGACCCAGCACTATGATGCCAAACCACAGGGCCGGGATGACAGATACTGTGAAAGCATCATGAGGAGACGGGGCCTGA
CCTCACCCTGCAAAGACATCAACACATTTATTCATGGCAACAAGCGCAGCATCAAGGCCATCTGTGAAAACAAGAATGGAAACCCTCAC
AGAGAAAACCTAAGAATAAGCAAGTCTTCTTTCCAGGTCACCACTTGCAAGCTACATGGAGGTTCCCCCTGGCCTCCATGCCAGTACCG
AGCCACAGCGGGGTTCAGAAACGTTGTTGTTGCTTGTGAAAATGGCTTACCTGTCCACTTGGATCAGTCAATTTTCCGTCGTCCGTAA

>Hs-RNase6
ATGGTGCTATGCTTTCCTCTTCTTTTACTGCTGCTGGTTCTATGGGGACCAGTGTGTCCACTTCATGCTTGGCCTAAGCGTCTCACCAA
GGCTCACTGGTTTGAAATTCAGCATATACAGCCAAGTCCTCTCCAATGCAACAGGGCAATGAGTGGCATCAACAATTATACCCAGCACT
GTAAGCATCAAAATACCTTTCTGCATGACTCTTTCCAGAATGTGGCTGCTGTCTGTGATTTGCTCAGCATTGTCTGCAAAAATCGTCGG
CACAACTGCCACCAGAGCTCAAAGCCTGTCAACATGACTGACTGCAGACTCACTTCAGGAAAGTATCCCCAGTGCCGCTATAGTGCTGC
TGCCCAGTACAAATTCTTCATTGTTGCCTGTGACCCCCCTCAGAAGAGCGATCCCCCCTACAAGTTGGTTCCTGTACACTTAGATAGTA
TTCTCTAA

>Hs-RNase7
ATGGCACCGGCCAGAGCAGGATTCTGCCCCCTTCTGCTGCTTCTGCTGCTGGGGCTGTGGGTGGCAGAGATCCCAGTCAGTGCCAAGCC
CAAGGGCATGACCTCATCACAGTGGTTTAAAATTCAGCACATGCAGCCCAGCCCTCAAGCATGCAACTCAGCCATGAAAAACATTAACA
AGCACACAAAACGGTGCAAAGACCTCAACACCTTCCTGCACGAGCCTTTCTCCAGTGTGGCCGCCACCTGCCAGACCCCCAAAATAGCC
TGCAAGAATGGCGATAAAAACTGCCACCAGAGCCACGGGGCCGTGTCCCTGACCATGTGTAAGCTCACCTCAGGGAAGCATCCGAACTG
CAGGTACAAAGAGAAGCGACAGAACAAGTCTTACGTAGTGGCCTGTAAGCCTCCCCAGAAAAAGGACTCTCAGCAATTCCACCTGGTTC
CTGTACACTTGGACAGAGTCCTTTAG

>Hs-RNase8
```

ATGGCACCGGCCAGAGCAGGATGCTGCCCCCTGCTGCTGCTGCTTCTGGGGCTGTGGGTGGCAGAGGTCCTAGTCAGAGCCAAGCCCAA
GGACATGACATCATCTCAGTGGTTTAAAACTCAGCATGTGCAGCCCAGCCCTCAAGCATGCAACTCAGCCATGAGCATCATCAATAAGT
ACACAGAACGGTGCAAAGACCTCAACACCTTCCTGCACGAGCCCTTCTCCAGTGTGGCCATCACCTGCCAGACCCCCAACATAGCCTGC
AAGAATAGCTGTAAAAACTGCCACCAGAGCCACGGGCCCATGTCCCTGACCATGGGTGAGCTCACCTCAGGGAAGTACCCAAACTGCAG
GTACAAAGAGAAGCACCTGAACACACCTTACATAGTGGCCTGTGACCCTCCACAACAGGGTGACCCAGGGTACCCACTTGTTCCTGTGC
ACTTGGATAAAGTTGTCTAA

>Hs-RNase9
ATGATGAGAACTCTCATCACCACACACCCACTGCCCCTGCTTCTATTGCCGCAGCAGCTGCTGCAGCTGGTGCAGTTTCAAGAGGTGGA
TACAGATTTTGATTTCCCAGAAGAAGATAAAAAAGAAGAATTTGAAGAGTGTTTGGAAAAATTTTTTAGTACAGGGCCCGCCAGACCAC
CTACCAAAGAAAAAGTCAAAAGACGTGTCCTTATTGAACCTGGAATGCCACTAAATCATATAGAGTACTGTAACCATGAAATCATGGGA
AAAAATGTTTACTACAAACACCGTTGGGTGGCAGAACATTACTTCCTTCTTATGCAATATGACGAGCTCCAAAAAATCTGTTACAACAG
ATTTGTGCCATGTAAGAATGGAATTAGGAAATGTAACAGGAGCAAAGGTCTTGTAGAAGGAGTGTATTGTAATTTAACAGAAGCATTTG
AAATACCAGCGTGTAAATACGAATCACTTTATAGGAAGGGCTACGTCCTTATCACTTGTTCATGGCAAAATGAAATGCAAAAACGTATT
CCTCATACTATAAATGATCTCGTGGAGCCACCTGAACACAGAAGTTTCCTCAGTGAGGATGGTGTCTTTGTCATATCGCCCTAG

>Hs-RNase10
ATGAAGCTGAATCTGGTGCAGATCTTTTTCATGTTGCTGATGCTGCTGCTGGGCCTGGGGATGGGCCTGGGGTTGGGACTTCATATGGC
TACAGCAGTCTTGGAGGAGAGTGATCAACCGCTCAATGAATTTTGGTCCAGTGACTCACAGGACAAAGCTGAGGCCACTGAGGAGGGAG
ACGGCACCCAAACCACAGAAACGCTGGTGCTTAGCAACAAAGAAGTGGTGCAACCTGGCTGGCCAGAAGATCCCATCCTCGGTGAAGAT
GAGGTTGGGGGTAACAAGATGCTCAGAGCCTCAGCTCTCTTTCAGAGCAACAAAGACTATCTTAGGCTTGACCAGACAGATAGAGAATG
CAATGATATGATGGCACACAAGATGAAGGAGCCCAGTCAGAGTTGCATAGCCCAGTATGCATTCATCCATGAGGATCTAAACACAGTCA
AAGCTGTCTGTAACAGTCCTGTCATTGCCTGTGAGCTCAAGGGGGGAAAATGTCACAAAAGCTCCCGACCTTTTGATTTGACATTGTGC
GAGCTGTCCCAACCAGACCAGGTCACTCCTAACTGCAATTACCTAACTTCTGTTATAAAAAAGCACATTATTATAACCTGTAATGACAT
GAAGCGCCAGTTACCAACTGGACAA

>Hs-RNase11
ATGGAGACCTTTCCTCTGCTGCTGCTCAGCCTGGGCCTGGTTCTTGCAGAAGCATCAGAAAGCACAATGAAGATAATTAAAGAAGAATT
TACAGACGAAGAGATGCAATATGACATGGCAAAAAGTGGCCAAGAAAAACAGACCATTGAGATATTAATGAACCCGATCCTGTTAGTTA
AAAATACCAGCCTCAGCATGTCCAAGGATGATATGTCTTCCACATTACTGACATTCAGAAGTTTACATTATAATGACCCCAAGGGAAAC
AGTTCGGGTAATGACAAAGAGTGTTGCAATGACATGACAGTCTGGAGAAAAGTTTCAGAAGCAAACGGATCGTGCAAGTGGAGCAATAA
CTTCATCCGCAGCTCCACAGAAGTGATGCGCAGGGTCCACAGGGCCCCCAGCTGCAAGTTTGTACAGAATCCTGGCATAAGCTGCTGTG
AGAGCCTAGAACTGGAAAATACAGTGTGCCAGTTCACTACAGGCAAACAATTCCCCAGGTGCCAATACCATAGTGTTACCTCATTAGAG
AAGATATTGACAGTGCTGACAGGTCATTCTCTGATGAGCTGGTTAGTTTGTGGCTCTAAGTTGTAA

>Hs-RNase12
ATGATAATAATGGTGATAATTTTCTTGGTGCTTCTGTTCTGGGAAAATGAGGTGAATGATGAAGCAGTGATGTCAACTTTAGAACACTT
GCATGTGGACTACCCTCAGAATGACGTTCCCGTTCCTGCAAGGTACTGCAACCACATGATCATACAAAGAGTTATCAGGGAACCTGACC
ACACTTGTAAAAAGGAGCATGTCTTCATCCATGAGAGGCCTCGAAAAATCAATGGTATTTGCATTTCTCCCAAGAAGGTTGCTTGCCAA
AACCTTTCGGCCATTTTCTGCTTTCAGAGTGAGACAAAGTTCAAAATGACAGTCTGTCAGCTCATTGAAGGCACAAGATACCCTGCCTG
CAGGTACCACTATTCCCCCACAGAGGGGTTTGTTCTTGTCACTTGTGATGACTTGAGGCCAGATAGTTTCCTTGGCTATGTTAAATAA

>Hs-RNase13
ATGGCACCAGCTGTGACCCGGCTCCTTTTCCTCCAGCTTGTTCTGGGGCCAACTCTGGTCATGGACATCAAGATGCAGATTGGCAGCAG
GAACTTCTATACCTTAAGCATTGACTATCCCAGGGTTAACTACCCAAAGGGTTTCCGGGGCTATTGTAATGGTCTGATGTCCTATATGC
GAGGCAAGATGCAAAATTCAGATTGCCCAAAGATCCATTATGTGATACATGCCCCTTGGAAGGCCATCCAGAAGTTCTGCAAGTATAGT
GACAGCTTCTGTGAGAATTACAATGAATACTGCACACTCACCCAGGATTCCCTCCCCATCACGGTCTGCTCCCTGAGCCACCAACAGCC
ACCCACTAGCTGCTACTACAATAGCACCCTAACCAACCAGAAGCTCTACCTACTCTGCTCCCGCAAGTATGAAGCTGATCCAATAGGTA
TCGCTGGTCTCTATTCGGGAATTTAA

>Hs-RNase14psA
ATGAATCTGATGTGGACCCTCCTCCTTTTCCTCCTTTTGGACGTAACTGTCTTCATTCCAGCCCTGCCCTTCTCAACACGACATTTAGA
CAACCCCAGGTCGTGGGTCCCTAGAGGACACCACCGATACTGTGATGTGATGATGAGGTGCCGTTGGCTGATCTATAGGGGTAAATGCG
AGCAGATCCACACATTCATTCATAGAATCTGACCACCATAGCAGATTTCTGCAGAACTCCACCACTGCCCTGTACCAACAGCCCCTCCA
TGTGCAGCTGCCACAACAGTACTCATGATGTCAATGTCACTGACTGCTTTGCCAGCACAGGGACCCGACCTCTTCACTGCCACTACCAA
AAATAAGAAGGAGTCCACCAGGCCCATGCGAGTGGGCTGCAAGAAGGGGGCATCTGTTCACCTGGATGGCTAG

>Hs-RNase14psB
ATGAATCTGATTTGGACCCTCCTCCTCTTCCTCCTTTTGGACCTAACTGTCTTCACTCCAGCCCTGCCCTTCTTAACACGACATATAGA
CAACCCCAGGTCGTGGGTCCCTAGAGGACACCACCGATACTGTGATGTGATGATGAGGcGCAGTTGGCTGATCTATAGGGGTAAATGCG
AGCAGATCCACACATTCATTCATAGAATCTGACCACCATAGCAGATTTCTGCAGAACTCCACCACTGCCCTGTACCAACAGCCCCTCCA
TGTGCAGCTGCCGCAACAGTACTCATGATGTCAATGTCACTGACTGCTTTGCCAGCGCAGGGACCCGACCTCTTCACTGCCACTACCAA
AAATAAGAAGGAGTCCACCAGGCCCATGCGAGTGGGCTGCAAGAAGGGGGCATCTGTTCACCTGGATGGCTAG

>Hs-RNase15ps
ATGGTTCTGAcGGGGACCCTCCTGTGTCTGCTGCTACGTCAGAGTGTTTCCCCCAGGCCCAGGACTTAGACAGTAGCCGTCTGATTACC
TGAGGTGCAGAATCTCAGGTGGGTCCAGCCACTATTGTGACGTGATAATATAGCATAGGGGAATGGTCTCCAGAGTCTGCAAGCTCATC
AACACCTTCATTCACTAGAGCATCAGAAGGATAAATGGCATATGCAAGGCGATATCTACTTTCGGGAcCTGACAGTGGACAACTGCTAC
CTCAGTCTCCgAACACTTGAgCTCACCCAGTGTGTGATTTCTGTGGGCTCCTGGCCCCCTAATTTCCGTTACAGGTTTAGGATGTTCTC
CCAGCACATCCACTTGACCTGTGTGTGGAACCAGCCAGTTCATCTGGAGGGCTAG

Chimpanzee (*Pan troglodytes*)

>Pt-RNase1
ATGGCTCTGGAGAAGTCTCTTGTCCTGCTCCCTCTGCTTGTCCTGATACTGCTGGTGCTGGGCTGGGTCCAGCCTTCCCTGGGCAAGGA
ATCCCGGGCCAAGAAATTCCAGCGGCAGCATGTGGACTCAGACAGTTCCCCCAGCAGCAGCTCCACCTACTGTAACCAAATGATGAGGC
GCCGGAATATGACACAGGGGCGGTGCAAACCAGTGAACACCTTTGTGCACGAGCCCCTGGTAGATGTCCAGAATGTCTGTTTCCAGGAA
AAGGTCACCTGCAAGAACGGGCAGGGCAACTGCTACAAGAGCAACTCCAGCATGCACATCACAGACTGCCGCCTGACAAACGGCTCCAG
GTACCCCAACTGTGCATACCGGACCAGCCCGAAGGAGAGACACATCATTGTGGCCTGTGAAGGGAGCCCATATGTGCCGGTCCACTTCG
ATGCTTCTGTGGAGGACTCCACCTAA

>Pt-RNase2
ATGGTTCCAAAACTGTTCACTTCCCAAATTTGTCTGCTTCTTCTGTTGGGGCTTCTGGCTGTGGAGGGCTCACTCCATGTCAAACCTCC
ACAGTTTACCTGGGCTCAATGGTTTGAAACCCAGCACATCAATATGACATCCCAGCAATGCACCAATGCAATGCAGGTCATTAACAATT
ATCAACGGCGATGCAAAAACCAAAATACTTTCCTTCTTACAACTTTTGCTAACGTAGTTAATGTTTGTGGTAACCCAAATATGACCTGT
CCTAGTAACAAAACTCGCAAAAATTGTCATCAAAGTGGAAGCCAGGTGCCTTTAATCCACTGTAACCTCACAACTCCAAGTCCACAGAA
TATTTCAAACTGCAGGTATGCGCAGACACCAGCAAACATGTTCTATATAGTTGCATGTGACAACAGAGATCAACGACGGGACCCTCCAC
AGTATCCGGTGGTTCCAGTTCACCTGGATAGAATCATCTAA

>Pt-RNase3
ATGGTTCCAAAACTGTTCACTTCCCAAATTTGTCTGCTTCTTCTGTTGGGGCTTATGGGTGTGGAGGGCTCACTCCATGCCAGACCCCC
ACAGTTTACGAGGGCTCAGTGGTTTGCCATCCAGCACATCAGTCTGAACCCCCCTCGATGCACCATTGCAATGCGGGTAATTAACAATT
ATCGATGGCGTTGCAAAAACCAAAATACTTTTCTTCGTACAACTTTTGCTAATGTAGTTAATGTTTGTGGTAACCAAAGTATACGCTGC
CCTCATAACAGAACCCTCAACAATTGTCATCAGAGTAGATTCCGGGTGCCTTTACTCCACTGTGACCTCATAAATCCAGGTGCACAGAA
TATTTCAAACTGCGGGTATGCAGACAGACCAGGAAGGAGGTTCTATGTAGTTGCATGTGACAACAGAGATCCACGGGATTCTCCACGGT
ATCCTGTGGTTCCAGTTCACCTGGATACCACCATCTAA

>Pt-RNase4
ATGGCTCTGCAGAGGACCCATTCATTGCTTCTGCTTTTGCTGCTGACCCTGCTGGGGCTGGGGCTGGTCCAGCCCTCCTATGGCCAGGA
TGGCATGTACCAGCGATTCCTGCGGCAACACGTGCACCCTGAGGAGACAGGTGGCAGTGATCGCTACTGCAACTTGATGATGCAAAGAC
GGAAGATGACTTTGTATCACTGCAAGCGCTTCAACACCTTCATCCATGAAGATATCTGGAACATTCGTAGTATCTGCAGCACCACCAAT
ATCCAATGCAAGAACGGCAAGATGAACTGCCATGAGGGTGTAGTGAAGGTCACAGATTGCAGGGACACAGGAAGTTCCAGGGCACCCAA
CTGCAGATATCGGGCCATGGCGAGCACTAGACGTGTTGTCATTGCCTGTGAGGGTAACCCACAGGTGCCTGTGCACTTTGACGGTTAG

>Pt-RNase5
ATGGTGATGGGCCTGGGCGTTTTGTTGTTGGTCTTCGTGCTGGGTCTGGGTCTGACCCCACCGACCCTGGCTCAGGATAACTCCAGGTA
CACACACTTCCTGACCCAGCACTATGATGCCAAACCACAGGGCCGGGATCACAGATACTGTGAAAGCATCATGAGGAGACGGGGCCTGA
CCTCACCCTGCAAAGACATCAACACATTTATTCATGGCAACAAGCGCAGCATCAAGGCCATCTGTGAAAACAAGAATGGAAACCCTCAC
AGAGAAAACCTAAGAATAAGCAAGTCTTCTTTCCAGGTCACCACTTGCAAGCTACATGGAGGGTCCCCCTGGCCTCCATGCCAGTACCG
AGCCACAGCGGGGTTCAGAAACGTTGTTGTTGCTTGTGAAAATGGCTTACCTGTCCACTTGGATCAGTCAATTTTCCGTCGTCCGTAA

>Pt-RNase6
ATGGTGCTATGCTTTCCTCTTCTTTTACTGCTGCTGGTTCTATGGGGACCAGTGTGTCCACTTCATGCTTGGCCTAAGCGTCTCACCAA
GGCTCACTGGTTTGAAATTCAGCATATACAGCCAAGTCCTCTCCAATGCAACAGGGCAATGAGTGGCATCAACAATTATGCCCAGCACT
GTAAGCATCAAAATACCTTTCTGCATGACTCTTTCCAGAATGTGGCTGCTGTCTGTGATTTGCTCAGCATTGTCTGCAAAAATCGTCGG
CACAACTGCCACCAGAGCTCAAAGCCTGTCAACATGACTGACTGCAGACTCACTTCAGGAAAGTATCCCCAGTGCCGCTATAGTGCTGC
TGCCCAGTACAAATTCTTCATTGTTGCCTGTGACCCCCCTCAGAAGAGCGACCCCCCCTACAAGTTGGTTCCTGTACACTTAGATAGTA
TTCTCTAA

>Pt-RNase7
ATGGCACCGGCCAGAGCAGGATTCTGCCCCCTTCTGCTGCTTCTCTTGCTGGGGCTGTGGGTGGCAGAGATCCCAGTCAGTGCCAAGCC
CAAGGGCATGACCTCATCACAGTGGTTTAAAATTCAGCACGTGCAGCCCAGCCCTCAAGCATGCAACTCAGCCATGAAAAACATTAACA
AGCACACAAAACGGTGCAAAGACCTCAACACCTTCCTGCACGAGCCTTTCTCCAGTGTGGCTGCCACCTGCCAGACCCCCAAAATAGCC
TGCAAGAATGGCGATAAAAACTGCCACCAGAGCCACGGGCCCGTGTCCCTGACCATGTGTAAGCTAACCTCAGGGAAGTATCCGAACTG
CAGGTACAAAGAGAAGCGACAGAACAAGTCTTACGTAGTGGCCTGTAAGCCTCCCCAGAAAAAGGACTCTCAGCAATTCCACCTGGTTC
CTGTACACTTGGACAGAGTCCTTTAG

>Pt-RNase8
ATGGCACCGGCCAGAGCAGGATGCTGCCCCCTGCTGCTGCTGCTGCTGCTGCTTCTGGGACTGTGGGTGGCAGAGGTCCTAGTCAGTGC
CAAGCCCAAGGACATGACATCATCTCAGTGGTTTAAAACTCAGCATGTGCAGCCCGGCCCTCAAGCATGCAACTCAGCCATAAGCAACA
TCAATAAGTACACAGAACGGTGCAAAGACCTCAACACCTTCCTGCACGAGCCCTTCTCCAGTGTGGCCATCACCTGCCAGACCCCCAAC
ATAGCCTGCAAGAATAGCTGTAAAAACTGCCATCAGAGCCACGGGCCCATGTCCCTGACCATGGGTGAGCTCACCTCAGGGAAGTACCC
AAACTGCAGGTACAAAGAGAAGCACCTGAACACACCTTACATGGTGGCCTGTGACCCTCCACAACAGGGTGACCCAGGGTACCCACTTG
TTCCTGTGCACTTGGATAAAGTTGTCTAA

>Pt-RNase9
ATGATGAGAACTCTGATCACCACACACCCACTGCCCCTGCTTCTATTGCCGCAGCAGCTGCTGCAGCCAGTGCAGTTTCAAGAGGTGGA
CACAGATTTTGATTTCCCAGAAGAAGATAAAAAAGAAGACTTTGAGGAGTATTTGGAACAATTTTTTAGTACAGGGCCTACCAGACCAC
CTACCAAAGAAAAAGTCAAAAGACGTGTCCTTATTGAATCTGGAATGCCACTAAATCATATAGAGTACTGTACCCATGAAATCATGGGA
AAAAATGTTTACTACAAACACCGTTGTGTGGCAGAACGTTACTTCCTTCTTATGCAATATGACGAGCTCCAAAAAATCTGTTACAACAG
ATTTGTGCCATGTAAGAATGGAATTAGGAAATGTAACAGGAGCAAAGGTCTTGTAGAAGGAGTGTATTGTAATTTAACAGAAGCATTTG

AAATACCAGCGTGTAAATACGAATCACTTTATAGGAAGGGCTATGTCCTTATCACTTGTTCATGGCAAAATGAAATGCAAAAACGTATT
CCTCATACTATAAATGATCTCGTGGAGCCACCTGAACACAGAAGTTTCCTCAGTGAGGATGGTGTCTTTGTCATACCGCCCTAG

>Pt-RNase10
ATGAAGCTGAATCTGGTGCAGATCTTTTTCATGTTGCTGATGCTGCTGCTGGGCCTGGGGATGGGCCTGGGGTTGGGACTTCATATGGC
TGCAGCAGTCTTGGAGGAGAGTGATCAACCGCTCAATGAATTTTGGTCCAGTGACTCACAGGACAAAGCTGAGGCCACTGAGGAGGGAG
ACGACATCCAAACCACAGAAACGCTGGTGCTTAGCAACAAAGAAGTGGTGCAACCTGGCTGGCCAGAAGATCCCATCCTCGGTGAAGAT
GAGGTTGGGGGTAACAAGATGCTCAGAGCCTCAGCGCTCTTTCAGAGCAACAAAGACTATCTTAGGCTTGACCAGACAGATAGAGAATG
CAATGATATGATGGCACACAAGATGAAGGAGCCCAATCAGAGTTGCATAGCCCAGTATGCATTCATCCATGAAGATCTAAACACAGTCA
AAGCTGTCTGTAACAGTCCTGTCATTGCCTGTGACCTCAAGGGGGGAAAATGTCACAAAAGCTCCCGACCTTTTGATTTGACATTGTGC
GAGCTGTCCAAACCAGACCAGGTCACTCCTAACTGCAATTACCTAACTTCTGTTATAAAAAAGCACATTATTATAACCTGTAATGACAT
GAAGCGCCAGTTACCAACTGGACAATGA

>Pt-RNase11
ATGGAGACCTTTCCTCTGCTGCTGCTCAGCCTGGGCCTGGTTCTTGCAGAAGCATCAGAAAGCACAATGAAGATAATTAAAGAAGAATT
TACAGACGAAGAGATGCAATATGACATGGCAAAAAGTGGCCAAGAAAAACAGACCATTGAGATATTAATGAACCCGATCCTGTTAGTTA
AAAATACCAGCCTCAGCATGTCCAAGGATGATATGTCTTCCTCATTACTGACATTCAGAAGTTTACATTATAATGACCCCAAGGGAAAC
AGTTCGGGTAATGACAAAGAGTGTTGCAATGACATGACAGTCTGGAGAAAAGTTTCAGAAGCAAACGGATGGTGCAAGTGGAGCAATAA
CTTCATCCACAGCTCCACGGAAGTGATGCGCAGGGTCCACAGGGCCCCCAGCTGCAAGTTTGTACAGAATCCTGGCATAAGCTGCTGTG
AGAGCCCAGAACTGGAAAATACAGTGTGCCAGTTCACTACAGGCAAACAATTCCCCAGGTGCCAATACCATAGTGTTACCTCATTAGAG
AAGATATTGACAGTGCTGACAGGTCATTCTCTGATGAGCTGGTTAGTTTGTGGCTCTAAGTTGTAA

>Pt-RNase12
ATGATAATAATGGTGATAATTTTCTTGGTGCTTCTGTTCTGGGAAAATGAGGTGAATGATGAAGCAGTGATGTCAACCTTAGAACACTT
GCATGTGGACTACCCTCAGAATGACGTTCCCGTTCCTGCAAGGTACTGCAACCACATGATCATACAAAGAGTTATCAGGGAACCTGACC
ACACTTGTAAAAAGGAGCATGTCTTCATCCATGAGAGGCCTCGAAAAATCAATGGTATTTGCATTTCTCCCAAGAAGGTTGCTTGCCAA
AACCTTTCGGCCATTTTCTGCTTTCAGAGTAAGACAAAGTTCAAAATGACAGTCTGTCAGCTCATTGAAGGCACAAGATACCCTGCCTG
CAGGTACCACTATTCCCCCACAGAGGGGTTTGTTCTTGTCACTTGTGATGACTTGAGGCCAGATAGTTTCCTTGGCTATGTTAAATAA

>Pt-RNase13
ATGGCACCAGCTGTGACCCGGCTCCTTTTCCTCCAGCTTGTTCTAGGGCCAACTCTGGTCATGGACATCAAGATGCAGATTGGCAGCAG
GAACTTCTATACCTTAAGCATTGACTATCCCAGGGTTAACTACCCAAAGGGTTTCCGGGGCTATTGTAATGGTCTGATGTCCTATATGC
GGGGCAAGATGCAAAATTCAGATTGCCCAAAGATCCATTATGTGATACATGCCCCTTGGAAGGCCATCCAGAAGTTCTGCAAGTATAGT
GACAGCTTCTGTGAGAATTACAATGAATACTGCACACTCACCCAGGATTCCCTCCCCATCACGGTCTGCTCCCTGAGCCACCAACAGCC
ACCCACTAGCTGCTACTATAATAGCACCCTAACCAACCAGAAGCTCTACCTACTCTGCTCCCGCAAGTATGAAGCTGATCCAATAGGTA
TCGCTGGTCTCTATTCGGGAATTTAA

>PtRNase14ps
ATGAATCTGATGTGGACCCTCCTCCTTTTCCTCCTTTTGGACCTAACTGTCTTCACTCCAGCCCTGCCCTTCTCAACACGACATATAGA
CAACCCCAGGTCTTGGGTCCCTAGAGGACACCACTGATACTGTGATGTGATGATGAGGTGCTGTTGGCTGTATCCATAGGGGTAAATGC
AAGCCGATCCACACATTCATTCATAGAATCTGACCACCATAGCAGATTTCTGCAGAACTCCACCACTGCCCTGTACCAACAGCCCCCTC
CATGTGCAGCTGCCACAACAGTACTCATGATGTCAATGTCACTGACTGCTTTGCCAGCACAGGGACCCGACCTCTTCGCTGCCACTACC
AAAAATAAGAAGGAGTCCACCAGGCCCATGCGAGTGGGCTGCAAGAAGGGGGCATCTGTTCACCTGGATGGCTAG

>Pt-RNase15ps
ATGGTTCTGATGGGGACCCTCCTGTGTCTGCTGCTACGTCAGAGTGTTTCCCCCAGGCCCAGGACTTAGACAGTAGCCGTCTGATTACC
TGAGGTGCAGAATCTCAGGTGGGTCCAGCCACTATTGTGACGTGATAATATAGCATAGGGGAATGGTCTCCAGAGTCTGCAAGCTCATC
AACACCTTCATTCACTAGAGCATCAGAAGGATAAATGGCATATGCAAGGCGATATCTACTTTCGGGATCTGACAGTGGACAACTGCTAC
CTCAGTCTCCAAACACTTGATCTCACCCAGTGTGTGATTTCTGTGGGCTCCTGGCCCCCTAATTTCCGTTACAGGTTTAGGATGTTCTC
CCAGCACATCCACTTGACCTGTGTGTGGAACCAGCCAGTTCATCTGGAGGGCTAG

Gorilla (*Gorilla Gorilla*)

>Gg-RNase1
ATGGCTCTGGAGAAGTCTCTTGTCCTGCTCCCTCTGCTTGTCCTGATACTGCTGGTGCTGGGCTGGGTCCAGCCTTCCCTGGGCAAGGA
ATCCCGGGCCAAGAAATTCCAGCGGCAGCATATGGACTCAGACAGTTCCCCCAGCAGCAACTCCACCTACTGTAACCAAATGATGAGGC
GCCGGAATATGACACAGGGGCGATGCAAACCAGTGAACACCTTTGTGCACGAGCCCCTGGTAGATGTCCAGAATGTCTGTTTCCAGGAA
AAGGTCACCTGCAAGAACGGGCAGGGCAACTGCTACAAGAGCAACTCCAGCATGCACATCACAGACTGCCGCCTGACAAACGGCTCCAG
GTACCCCAACTGTGCATACCGGACCAGCCCGAAGGAGAGACACATCATTGTGGCCTGTGAAGGGAACCCATATGTGCCAGTCCACTTCG
ATGCTTCTGTGGAGGACTCCACCTAA

>Gg-RNase2
ATGGTTCCAAAACTGTTCACTTCCCAAATTTGTCTGCTTCTTCTGTTGGGGCTTCTGGCAGTGGAGGGCTCACTCCATGTCAAACCTCC
ACAGTTTACCTGGGCTCAATGGTTTGAAACCCAGCACATCAATATGACCTCCCAGCAATGCACCAATGCAATGCGGGTCATTAACAATT
ATCAACGGCGATGCAAAAACCAAAATACTTTCCTTCTTACAACTTTTGCTAACGTAGTTAATGTTTGTGGTAACCCAAATATGACCTGT
CCTAGTAACAAAACTCGCAAAAATTGTCATCACAGTGGAAGCCAGGTGCCTTTAATCCACTGTAACCTCACAACTCCAAGTCCACAGAA
TATTTCAAACTGCAGGTATGCGCAGACACCAGCAAACATGTTCTATATAGTTGCATGTGACAACAGAGATCAACGACGGGACCCTCCAC
AGTATCCAGTGGTTCCAGTTCACCTGGATAGAATCATCTAA

>Gg-RNase2ps

ATGGTTCCAAAACTGTTCACTTCCCAAATTTGTCTGCTTCTTCTGTTGGGGCTTCTGGCTGTGGAGGGCTCACTCCATGTCAAACCTCC
ACAGTTTACCTGGGCTCANNNNNNNNNNNNNNNNNNNNNNNNNNNNNNNNNNNNNNNNNNNNNNNNNNNNNNNNNNNNNNNNNNNNNNNN
NNNNNNNNNNNNNNNNNNNNNNNNNNNNNNNNNNNNNNNNNNNNNNNNNNNNNNNNNNNNNNNNNNNNNNNNNNNNNNNNNNNNNNNNNN
NNNNNNNNNNNNNNNNNNNNNNNNNNNNNNNNNNNNNNNNNNNNNNNNNNNNNNNNNNNNNNNNNNNNNNNNNNNNNNNNNNNNNNNNNN
NNNAACTGCAGGTATGCGCAGACACCAGCAAACATGTTCTATATAGTTGCATGTGACAACAGGGATCAACGACGGGACCCTCCACAGTA
TCCAGTGGTTCCAGTTCACCTGGATACCATCATCTAA

>Gg-RNase3
ATGGTTCCAAAACTGTTCACTTCCCAAATTTGTCTGCTTCTTCTGTTGGGGCTTATGGGTGTGGAGGGCTCACTCCATGCCAGACCCCC
ACAGTTTACGAGGGCTCAGTGGTTTGCCATCCAGCACATCAGTCTGAACCCCCCTCGATGCACCATTGCAATGCGGGTAATTAACAATT
ATCGATGGCGTTGCAAAAACCAAAATACTTTTCTTCGTACAACTTTTGCTAATGTAGTTAATGTTTGTGGTAACCAAAGTATACGCTGC
CTTCATAACAGAACTCTCAACAATTGTCATCGGAGTAGATTCCGGGTGCCTTTACTCCACTGTGACCTCATAAATCCAGGTGCACAGAA
TATTTCAAACTGCAGGTATGCAGACAGACCAGGAAGGAGGTTCTATGTAGTTGCATGTGACAACAGAGATCCACAGGATTCTCCACGGT
ATCCTGTGGTTCCTGTTCACCTGGATACCACCATCTAA

>Gg-RNase4
ATGGCTCTGCAGAGGACCCGTTCATTGCTTCTGCTTTTGCTGCTGACCTTGCTGGGGCTGGGGCTGGTCCAGCCCTCCTATGGCCAGGA
TGGCATGTACCAGCGATTCCTGCGGCAACACGTGCACCCTGAGGAGACAGGTGGCAGTGATCGCTACTGCAACTTGATGATGCAAAGAC
GGAAGATGACTTTGTATCACTGCAAGCGCTTCAACACCTTCATCCATGAAGATATCTGGAACATTCGTAGTATCTGCAGCACCACCAAT
ATCCAATGCAAGAACGGCAAGATGAACTGCCATGAGGGTGTAGTGAAGGTCACAGATTGCAGGGACACAGGAAGTTCCAGGGCACCCAA
CTGCAGATATCGGGCCATGGCGAGCACTAGACGTGTTGTCATTGCCTGTGAGGGTAACCCACAGGTGCCTGTGCACTTTGACAGTTAG

>Gg-RNase5
ATGGTGATGGGCCTGGGCGTTTTGTTGTTGATCTTCGTGCTGGGTCTGGGTCTGACCCCACCGACCCTGGCTCAGGATAACTCCAGGTA
CACACACTTCCTGACCCAGCACTATGATGCCAAACCACAGGGCCGGGATGACAGATACTGTGAAAGCATCATGAGGAGACGGGGCCTGA
CCTCACCCTGCAAAGACATCAACACATTTATTCATGGCAACAAGCGCAGCATCAAGGCCATCTGTGAAAACAAGAATGGAAACCCTCAC
AGAGAAAACCTAAGAATAAGCAAGTCTTCTTTCCAGGTCACCACTTGCAAGCTACATGGAGGGTCCCCCTGGCCTCCATGCCAGTACCG
AGCCACAGCGGGGTTCAGAAACGTTGTTGTTGCTTGTGAAAATGGCTTACCTGTCCACTTGGATCAGTCAATTTTCCGTCGTCCGTAA

>Gg-RNase6
ATGGTGCTACGCTTTCCTCTTCTTTTACTGCTGCTGGTTCTATGGGGACCAGTGTGTCCACTTCATGCTTGGCCTAAGCGTCTCACCAA
GGCTCACTGGTTTGAAATTCAGCATATACAGCCAAGTCCTCTCCAATGCAACAGGGCAATGATTGGCATCAACAATTATACCCAGCACT
GTAAGCATCAAAATACCTTTCTGCATGACTCTTTCCAGAATGTGGCTGCTGTCTGTGATTTGCTCAGCATTGTCTGCAAAAATCGTCGG
CACAACTGCCACCAGAGCTCAAAGCCTGTCAACATGACTGACTGCAGACTCACTTCAGGAAAGTATCCCCAGTGCCGCTATAGTGCTGC
TGCCCAGTACAAATTCTTCATTGTTGCCTGTGACCCCCCTCAGAAGAGCGACCCCCCCTACAAGTTGGTTCCTGTACACTTAGATAGTA
TTCTCTAA

>Gg-RNase7
ATGGCACCGGCCAGAGCAGGATTCTGCCCCCTTCTGCTGCTTCTGCTGCTGGGGCTGTGGGTGGCAGAGATCCCAGTCAGTGCCAAGCC
CAAGGGCATGACCTCATCTCAGTGGTTTAAAATTCAGCACGTGCAGCCCAGCCCTCAAGCATGCAACTCAGCCATGAAAAACATCAACA
AGCACACAAAACGGTGCAAAGACCTCAACACCTTCCTGCACGAGCCTTTCTCCAGTGTGGCCGCCACCTGCCAGACCCCCAAAATAGCC
TGCAAGAATGGCGATAAAAACTGCCACCAGAGCCACGGGCCCGTGTCCCTGACCATGTGTAAGCTCACCTCAGGGAAGTATCCGAACTG
CAGGTACAAAGAGAAGCGACAGAACAAGTCTTACGTAGTGGCCTGTAAGCCTCCCCAGAAAAAGGACTCTCAGCAATTCCACCTGGTTC
CTGTACACTTGGACAGAGTCCTTTAG

>Gg-RNase8ps
ATGGCACCGGCCAGAGCAGGACGCTGCCCCCTGCTGCTGCTGCTTCTGGGGCTGTGGGTGGCAGAGGTCCTAGTCAGAGCCAAGCCCAA
GGACATGACATCATCTCAGTGGTTTAAAACTCAGCATGTGCAGCCCAGCCCTCAAGCATGCAACTCAGCCATGAGCAaCATCAATAAGT
ACACAGAACGGTGCAAAGACCTCAACACCTTCCTGCACGAGCCCTTCTCCAGTGTGGCCATCACCTGCCAGACCCCCAACATAGCCTGC
AAGAATAGCTGCAAAAACTGCCCTGACCATGAGTGAGCTCACCTCAGGGAAGTACCCAAACTGCAGGTACAAAGAGAAGCACCTGAACG
CGCCTTACATAGTGGCCTGTGGCCCTCCACAACAGGGTGACCCAGGGTACCCACTTGTTCCTGTGCACTTGGATAAAGTTGTCTAA

>Gg-RNase9
ATGATGAGAACTCTGATCACCATACACCCACTGCCCCTGCTTCTATTGCTGCAGCAGCTGCTGCAGCCAGTGCAGTTTCAAGAGGTGGA
TACAGATTTTGATTTCCCAGAAGAAGATAAAAAAGAAGAATTTGAGGAGTATTCGGAACAATTTTTTAGTATAGGGCCCACCAGACCAC
CTACCAAAGAAAAAGTCAAAAGACGTGTCCTTATTGAACCTGGAATGCCACTAAATCATATAGAGTACTGTAATCATGAAATCATGGGA
AAAAATGTTTACTACAAACACCGTTGTGTGGCAGAACATTACTTCCTTCTTATGCAATATGACGAGCTCCAAAAAATCTGTTACAACAG
ATTTGTGCCATGTAAGAATGGAATTAGGAAATGTAACAGGAGCAAAGGTCTTGTAGAAGGAGTGTATTGTAATTTAACAGAAGCATTTG
AAATACCAGCGTGTAAATACGAATCACTTTATAGGAAGGGCTATGTCCTTATCACTTGTTCATGGCAAAATGAAATGCAAAAACTTATT
CCTCATACTATAAATGATCTCGTGGAGCCACCTGAACACAGAAGTTTCCTCAGTGAGGATGGTGTCTTTGTCATACCGCCCTAG

>Gg-RNase10
ATGAAACTGAATCTGGTGCAGATCTTTTTCATGTTGCTGATGCTGCTGCTGGGCCTGGGGATGGGCCTGGGGTTGGGACTTCATATGGC
TGCAGCAGTCTTGGAGGAGAGTGATCAACCGCTCAATGAATTTTGGTCCAGTGACTCACAGGACAAAGCTGAGGCCACTGGGGAGGGAG
ACGGCACCCAAACCACAGAAACGCTGGTGCTTACCAACAAAGAAGTGGTGCAACCTGGCTGGCCAGAAGATCCCATCCTCGGTGAAGAT
GAGGTTGGGGGTAACAAGATGCTCAGAGCCTCAGCTCTCTTTCAGAGCGACAAAGACTATCTTAGGCTTGACCAGACAGATAGAGAATG
CAATGATATGATGGCACACAAGATGAAGGAGCCCAATCAGAGTTGCATAGCCCAGTATGCATTCATCCATGAGGATCTAAACACAGTCA
AAGCTGTCTGTAACAGTCCTGTCATTGCCTGTGAGCTCAAGGGGGGAAAATGTCACAAAAGCTCCCGACCTTTTGATTTGACATTGTGC
GAGCTGTCCAAACCAGACCAGGTCACTCCTAACTGCAATTACCTAACTTCTGTTATAAAAAAGCACATTATTATAACCTGTAATGACAT
GAAGCGTCAGTTACCAACTGGACAATGA

>Gg-RNase11
ATGGAGACCTTTCCTCTGCTGCTGCTCAGCCTGGGCCTGGTTCTTGTAGAAGCATCAGAAAGCACAATGAAGATAATTAAAGAAGAATT
TACAGATGAAGAGATGCAATATGACATGGCAAAAAGTGGCCAAGAAAAACAGACCATTGAGATATTAATGAACCCGATCCTGTTAGTTA
AAAATACCAGCCTCAGCATGTCCAAGGATGATATGTCTTCCTCATTACTGACACTCAGAAGTTTACATTATAATGACCCCAAGGGAAAC
AGTTCGGGTAATGACAAAGAGTGTTGCAATGACATGACAGTCTGGAGAAAAGGTTCAGAAGCAAGCGGATCGTGCAAGTGGAGCAATAA
CTTCATCCACGGCTCCACGGAAGTGATGCGCAGGGTCCACAGGGCCCCCAGCTGCAAGTTTGTACAGAATCCTGGCATAAGCTGCTGTG
AGAGCCCAGAATTGGAAAATACAGTGTGCCAGCTCACTACAGGCAAACAATTCCCCAGGTGCCAATACCATAGTGTTACCTCATTAGAG
AAGATATTGACAGTGCTGACAGGTCATTCTCTGATGAGCTGGTTAGTTTGTGGCTCTAAGTTGTAA

>Gg-RNase12
ATGATAATAATGGTGATAATTTTCTTGGTGCTTCTGTTCTGGGAAAATGAGGTGAATGATGAAGCAGTGATGTCAACCTTAGAACACTT
GCATGTGGACTACCCTCGGAATGACGTTCCCGTTCCTGCAAGGTACTGCAACCACATGATCATACAAAGAGTTATCAGGGAACCTGACC
ACACTTGTAAAAGGAGCATGTCTTCATCCATGAGAGGCCTCGAAAAATCAATGGTATTTGCATTTCTCCCAAGAAGGTTGCTTGCCAA
AACCTTTCGGCCATTTTCTGCTTTCAGAGTGAGACAAAGTTCAAAATGACAGTCTGTCAGCTCATTGAAGGCACAAGATACCCTGCCTG
CAGGTACCACTATTCCCCCACAGAGGGGTTTGTTCTTGTCACTTGTGATGACTTGAGGCCAGATAGTTTCCTTGGCTATGTTAAATAA

>Gg-RNase13
ATGGCACCAGCTGTGACCCGGCTCCTTTTCCTCCAGCTTGTTCTAGGGCCAACTCTGGTCATGGACATCAAGATGCAGATTGGCAGCAG
GAACTTCTATACCTTAAGCATTGACTATCCCAGGGTTAACTACCCAAAGGGTTTCCGGGGCTATTGTAATGGTCTGATGTCCTATATGC
GGGGCAAGATGCAAAATTCAGATTGCCCAAAGATCCATTACGTGATACATGCCCCTTGGAAGGCCATCCAGAAGTTCTGCAAGTATAGT
GACAGCTTCTGTGAGAATTACAATGAATACTGCATACTCACCCAGGATTCCtTCCCCATCACGGTCTGCTCCCTGAGCCACCAACAGCC
ACCCACTAGCTGCTACTACAATAGCACCCTAACCAACCAGAAGCTCTACCTACTCTGCTCCCGCAAGTATGAAGCTGATCCAATAGGTA
TCGCTGGTCTCTATTCCGGAATTTAA

>Gg-RNase14psA
ATGAATCTGATGTGGACCCTCCTCCTTTTCCTCCTTTTGGACGTAACTGTCTTCACTCCAGCCCTACCCTTCTCAACACGACATATAGA
CAACCCCAGGTCGTGGGTCCCTAGAGGACACCACCGATACTGTGATGTGATGATGAGGTGCTGTTGGCTGATCTATAGGGGTAAATGCG
AGCCGATCCACACATTCATTCATAGAATCTGACCACCATAGCAGATTTCTGCAGAACTCCACCACTGCCCTGTACCAACATCCCCTCCA
TGTGCAGCTGCCACAACAGTACTCATGATGTCAATGTCACTGACTGCTTTGCCAGCACAGGGACCCGACCTCTTCACTGCCACTACCAA
AAATAAGAAGGAGTCCACCAGGCCCATGCGAGTGGGCTGCAAGAAGGGGGCATCTGTTCACCTGGATGGCTAG

>Gg-RNase14psB
ATGAATCTGATATGGACCCTCCTCCTCTTCCTCCTTTTGGACGTAACTGTCTTCACTCCAGCCCTGCCCTTCTCAACACGACATATAGA
CAACCCCAGGTCGTGGGTCCCTAGAGGACACCACCGATACTGTGATGTGATGATGAGGTGCTGTTGGCTGATCTATAGGAGTAAATGCG
AGCCGATCCACACATTCATTCATAGAATCTGACCACCATAGCAGATTTCTGCAGAACTCCACCACTGCCCTGTACCAACAGCCCCTTCA
TGTGCAGCTGCCACAACAGTACTCATGATGTCAATGTCACTGACTGCTTTGCCAGCACAGGGACCCGACCTCTTCACTGCCACTACCAA
AAATAAGAAGGAGTCCACCAGGCCCATGCGAGTGGGCTGCAAGAAGGGGGCATCTGTTCACCTGGATGGCTAG

>Gg-RNase15ps
ATGGTTCTGACGGGGACCCTCCTGTGTCTGTTGCTACGTCAGACTGTTTCCCCCAGGCCCAGGACTTAGATAGTAGCCGTCTGATTACC
TGAGGTGCAGAATCTCAGGTGGGTCCAGCCACTATTGTGACGTGATAATATAGCATAGGGGAATGGTCTCCAGAGTCTGCAAGCTCATC
AACACCTTCATTCACTGGAGCATCAGAAGGATAAATGGCATATGCAAGGCGATATCTACTTTCGGGACCTGACAGTGGGCAACAGCTAC
CTCAGTCTCCGAACACTTGAGCTCACCCAGTGTGTGATTTCTGTGGGCTCCTGGCCCCCTAATTTCCGTTACAGGTTTAGGATGTTCTC
CCAGCACATCCACTTGACCTGTGTGTGGAACCAGCCAGTTCATCTGGAGGGCTAG

Orangutan (*Pongo pygmaeus*)

>Pp-RNase1
ATGGCTCTGGAGAAATCTCTTGTCCTGCTCCCTCTGCTTGTCCTGATACTGCTGGTGCTGGGCTGGGTCCAGCCTTCCCTGGGCAAGGA
ATCCCGGGCCAAGAAATTCCAGCGGCAGCACATGGACTCAGGCAGTTCCCCCAACAGCAACTCCACCTACTGCAACCAAATGATGAGGC
GCCGGAATATGACACAGGGGCGGTGCAAACCAGTGAACACCTTTGTGCACGAGCCCCTGGTAGATGTCCAGAATGTCTGTTTCCAGGAA
AAGGTCACCTGCAAGAACGGGCAGGGCAACTGCTACAAGAGCAACTCCAGCATGCACATCACAGACTGCCGCCTGACACACGGCTCCAG
GTACCCCAACTGTGCATACCGGACCAGCCCGAAGGAGAGACACATCATTGTGGCCTGTGAAGGGAGCCCATATGTGCCAGTCCACTTCG
ATGCTTCTGTGGAGGACTCCACCTAA

>Pp-RNase2
ATGGTTTCAAAACTGTTCACTTCTCAAATTTGTCTGCTTCTTCTGTTGGGGCTTCTGGCTGTGGACGGCTCACTCCATGTCAAACCTCC
ACAGTTTACCTGGGCTCAATGGTTTGAAACCCAGCACATCAATATGACCTCCCAGCAATGCAACAATGCAATGCAGGTCATTAACAATT
TTCAACGGCGTTGCAAAAACCAAAATACTTTTCTTCGTACAACTTTTGCTAATGTAGTTAATGTTTGTGGTAACCCAAATATAACCTGT
CCTAGTAACAGAAGTCGCAACAATTGTCATCATAGTGGAGTCCAGGTGCCTTTAATCCACTGTAACCTCACAACTCCAAGTCCACAGAA
TATTTCAAACTGCAGGTATGCGCAGACACCAGCAAACATGTTCTATATAGTTGCATGTGACAACAGGGATCCACGACGGGACCCTCCAC
AGTATCCGGTGGTTCCAGTTCACCTGGATAGAATCATCTAA

>Pp-RNase2ps
ATGGTTCCAAAACTGTTCACTTCTCATATTTGTCTGCTTCTTCTGTTGGGGCTTCTGGCTGTGGACGGCTCACTCCATGTCAAACCTCC
ACAGTTTACCTGGGCTCAGTGGTTTGACGTCCATCAGTAGGAACCCCCTCCAATGCACCAATGCAATGAGGGTAATTAACAATTATCAA
CGGCAATGCAAAAACCAAAATACTTTTCTTCTTATAAGTTTTGCTAATGTAGTTAATGTTTGTGGTAACCCAAATATAACCTGTCTTCA
TAACAGAACTCTCAACAATTGTCATCATAATGGAGTCTGGGTGCCTTTAATCCACTGTAACCTCACGACTCCAAGTCCACAGAATATTT
CAAACTGCAGGTATGCACAAACACCAGCAAACATGTTCTACATAGTTGCATGTGACAACAGAGATCCATGACAGGACTCTCCACAGTAT
GCAGTGGTTCCAGTTCACCTGGATACCATCATCTAA

>Pp-RNase3
ATGGTTCCAAAACTGTTCACTTCCCAAATTTGTCTGCTTCTTCTGTTGGGGCTTAGTGGTGTGGGGGGCTCACTCCATGCCAAACCCCG
ACAGTTTACGAGGGCTCAGTGGTTTGCCATCCAGCACGTCAGTCTGAACCCTCCTCAATGCGCCACTGCAATGCGGGTAATTAACAATT
ATCAACGGCGTTGCAAAGACCAAAATACTTTTCTTCGTACAACTTTTGCTAATGTAGTTAATGTTTGTGGTAACCCAAATATAACCTGT
CCTAGTAACAGAAGTCGCAACAATTGTCATCATAGTGGAGTCCAGGTGCCTTTCATCTACTGTAACCTCACAACTCCAAGTCCACAGAA
TATTTCAAACTGCAGCTATGCAAACATAACAGGAAGGAGGTTCTATATAGTTGCATGTGACAACAGAGATCCACGGGATTCTCCACAGT
ATCCTGTGGTTCCAGTTCACCTGGATACCATCATCTAA

>Pp-RNase4
ATGGCTCTGCAGAGGACCCATTCATTGCTTCTGCTTTTGCTGCTCACCCTGCTGGGGCTGGGGCTGGTCCAGCCCTCCTATGGCCAGGA
TGGCATGTACCAGCGATTCCTGCGGCAACACGTGCACCCTGAGGAGACAGGTGGCAATGATCGCTACTGCAACTTGATGATGCAAAGAC
GGAAGATGACTTTGTATCACTGCAAGCGCTTCAACACCTTCATCCATGAAGATATCTGGAACATTCGTAGTATCTGCAGCACCACCAAT
ATCCAGTGCAAGAACGGCAAGACGAACTGCCACGAGGGTGTTGTGAAAGTCACAGACTGCCGGGACACAGGAAGTTCCAGGGCACCCAA
CTGCAGATATCGGGCCATGGCGAGCACTAGACGTGTTGTCATTGCCTGTGAGGGGAACCCACAGGTGCCTGTGCACTTTGACGGTTAG

>Pp-RNase5
ATGGTGATGGGCCTGGGCGTTTTGTTGTTGGTCTTCATGCTGGGTCTGGGTCTGACCCCACCAACCCTGGCTCAGGATAACTCCAGGTA
CACAGACTTCCTGGCCCAGCACTATGATCCCAAACCACAGGGCCGGGATGACAGATACTGTGAAAGCATCATGAGGAGACGGGGCCTGA
CCTCACCCTGCAAAGGCATCAACACATTTATTCATGGCAGCAAGCGCAGCATCAAGGCCATCTGTGAAAACAAGAATGGAAACCCTCAC
AGAGAAAACCTAAGAATAAGCAAGTCTTCTTTCCAGGTCACCACTTGCAAGCTACATGGAGGGTCCCCCTGGCCTCCATGCCACTACCG
AGCCACAGCAGACTTCAGAAACATTGTTGTTGCTTGTGAAAATGGCTTACCTGTCCACTTGGATCAGTCAATTTTCCGTCGTCTGTAA

>Pp-RNase6
ATGATGCTACGCTTTCCTCTTCTTTTATTGCTGCTCGTTCTATGGGGACCAGTGTGTCCACTTCATGCTTGGCCTAAGCGTCTCACCAA
GGCTCACTGGTTTGAAATTCAGCATATACAGCCAAGTCCTCTCCAATGCAACAGGGCAATGAGTGGCATCAACAATTATACCCAGCACT
GTAAGCATCAAAATACCTTTCTGCATGACTCTTTCCAGAATGTGGCTGCTGTCTGTGATTTGCTCAGCATTGTCTGCAAAAATCATAGG
CACAACTGCCACCAGAGCTCAAAGCCTGTCAACATGACTGACTGCAGACTCATTTCAGGAAAGTATCCCCAGTGCCGCTATAGTGCTGC
TGCCCAGTACAAATTCTTCATTGTTGCCTGTGATCCACCTCAGAAGAGCGACCCCCGCTACAAGTTGGTTCCTGTACACTTAGATAGTA
TTGTCTAA

>Pp-RNase7
ATGGCACCGGCCAGAGCAGGATTCTGCCCCCTTCTGCTGCTTCTGCTGCTGGGGCTGTGGGTGGCAGAGATCCCAGTCAGTGCCAAGCC
CAAGGGCTTGACCTCATCTCAGTGGTTTAAAATTCAGCACGTGCAGCCCAGCCCTCAAGCATGCAACTCAGCCATGAAAAACATCAACA
AGCACACAAAACGGTGCAAAGACCTCAACACCTTCCTACACGAGCCTTTCTCCAGTGTGGCCGCCACCTGCCAGACCCCCAAAAAAGCC
TGCAAGAATGGCGAGAAAAACTGCCACCAGAGCCACGGGCCCGTGTCCCTGACCATGTGTAAGCTCACCTCAGGGAAGTATCCGAACTG
CAGGTACAAAGAGAAGCGACAGAACAAGTCTTACATAGTGGCCTGTAAGCCTCCCCAGAAAAACGACTCTCAGAAATTCCACCTGGTTC
CTGTGCACTTGGACAGAGTCCTTTAG

>Pp-RNase8
ATGGCACCAGCCAGAGCAGGATGGTGCGCCGTGGTGATGCTGCTTCTGGGGCTGTGGGTGGCAGAGATCCCAGTCAGTGCCAAGCCCAA
GGACATGACATCATCTCAGTGGTTTAAAACTCAGCATATGCAGCCCAGCCCTCAAGCATGCAACTCAGCCATGAACAACATCAATAAGT
ACACAGAACAGTGCAAAGACCTCAACACCTTCCTGCATGAGCTCTTCTCCAGTGTGGCCACCACCTGCCAGACCCCCAACATAGCCTGC
AAGAATAGCCGTAAAAACTGCCACCAGAGCCACGGGCCCATGTCCCTGACCATGTGTGAGCTCACCTCAGGGAAGTACCCAAACTTCAG
GTACAAAGAGAAGCACCTGAACGCACCTTACATAGCAGCCTGTGACCCTCCACAACAGGGTGACCCAGGGTACCCACTTGTTCCTGTGC
ACTTGGATAAAGTTGTCTAA

>Pp-RNase9
ATGATGAGAACTCTGATCACCACACACCCACTGCCCCTGCTTCTATTGCTGCAGCAGCTGCTGCAGCCAGTGCAGTTTCAAGAGGTGGA
TACAGATTATGATCTCCCAGAAGATAAAAGAGAAGAATTTGAAGAGTATGTGGAACAATTTTTTAGTACAGGGCCCACCAGACCACCTA
CCAAAGAAAAAGTCAAAAGACTTCTCCTTATTGAACCTGGAATGCCGTTATATCATGTAGACTACTGTAATAGTGAAATCATGAGAAAA
AATGTTTACTATAAACACCGTTGTGTGGCAGAACATTACTTCCTTCTTATGCAGTACGATGAGCTCCAAAAAATCTGTTACAACAGATT
CGTGCCATGCAAGAATGGAGTTAGGAAATGTAACAGGAGCAAAGGTCTTGTAGAAGGAGTGTATTGTAATTTAACAGAAGCATTTCGAA
TACCAGCATGTAAATACGAATCATTTTATAGGAAGGGCTATGTCCTTATCACTTGTGCATGGCAAAATGAAATGCAAAAACTTATTCCT
CATACCATAAATGATCTCGTGGAGCCACCTGAACACAGAAGTTTCCTCAGTGAGGATGGTGTCTTTGTCATACTGCCCTAG

>Pp-RNase10
ATGAAGCTGAATCTGGTGCAGATCTTTTTCATGTTGCTGATGCTGCTGCTGGGCCTGGGGATGGGCCTGGGGTTGGGGCTTCATATGGC
TGCAGCAGTCTTGGAGGAGAGTAATCAACCGCTCAATGAATTTTGGTCCAGTGACTCACAGGACAAAGCTGAGGCCACTGAGGAGGGAG
AGGGCACCCAAACCACAGAAACGCTGGTGCTTAGCAACAAAGAAGTGGTGCAACCTGGCTGGCCGGAAGATCCCATCCTTGGTGAAGAT
GAGGTTGGAGGAAACAAGATGCCCAGAGCCTCAGCTCTCTTTCAGAGCAACAAAGACTATCTTAGGCTTGACCAGACAGATAGAGAATG
CAATGATATGATGGCACACAAGATGAAGGAGCCCAATCAGAGTTGCATAGCCCAGTACGCATTCATCCATGAGGATCTAAACACAGTCA
AAGCTGTCTGTAACAGTCCTGTCATTGCCTGTGAGCTCAAGGGCGGAAAATGTCACAAAAGCTCCAGACCTTTTGATTTGACATTGTGC
GAGCTGTCCAAACCAGACCAGGTCACTCCTAACTGCAATTACCTAACTTCTGTTATAAAAAAGCACATTATTATAACCTGTAATGACGT
GAAGCGCCAGTTACCAACTGGACAATGA

>Pp-RNase11
ATGGAGACCGTTCCTCTGCTGCTGCTCAGCCTGGGCCTGGTTCTTGCAGAAGCATCAGAAAGCACAATGAAGATAATTAAAGAAGAATT
TATAGAGGAAGAGATGCAATATGACATGGCAAAAAGTGGCCAAGAAAAACAGACCATTGAGATATTGATGAACCCGATCCAGTTAGTTA
AAAATACCAGCCTCAGCATGTCCAAGGATGATATGTCTTCCTCATTACTGACATTCAGGAGGTTACATTATAGTGGCCCCAAAGGAGAT
AGTTCAGGTAATGACAAAGAGTGTTGCAATGACATGACAGTCTGGAGAAAAGTTTCAGAAGCAAACAGATCGTGCAAATGGAACAATAA
CTTCATCCATGGCTCCACGGAAGTGATGCGCAGGGTCCACAAGGCCCCCAGCTGCAAGTTTGTACAGAATCCTGGCATAAGCTGCTGTG

AGAGCCCAGAACTGGAAAATACAGTGTGCCAGCTCACTACAGGCAAACAATTCCCCAGGTGCCAATACCATAGTGTTACCTCATTAGAG
AAGATACTGACAGTGCTGACAGGTCATTCTCTGATGAGCTGGTTAGTTTGTGGCTCTAAGTTGTAA

>Pp-RNase12
ATGATAATAATGGTGATAATTTTCTTGGTGCTTCTGTTCTGGGAAAATGAGGTGAATGATGAAGTAGTGATGTCAACCTTAGAACACTT
GCATGTGGACTATCCTCAGAATGACGTTCCCGTTCCTGCAAGGTACTGCAACCACATGATCATACAAAGAGTTATCAGGGAACCTGACC
ACACTTGTAAAAAGGAGCATGTCTTCATCCATGAGAGGCCTCGAAAAATCAATAGTATTTGCATTTCTCCCAAGAAGGTTGCTTGCCAA
AACGTTTCAGCCATTTTCTGCTTTCAGAGTGAGACAAAGTTCAAAATGACAGTCTGTCAACTCATTGAAGGCACAAGATACCCTGCCTG
CAGGTACCACTATTCCCCCACAGAGGGGTTTGTTCTTGTCACTTGTGATGACTTGAGGCCAGATAGTTTCCTCGGCTATGTTAAATAA

>Pp-RNase13
ATGGCACCAGCTGTGACCCGGCTCCTTTTCCTCCAGCTTGTTCTAGGGCCAACTCTGGTCGTGGACATCAAGATGCAGATTGGCAGCAG
GAACTTCTATACCTTAAGCATTGACTATCCCAGGGTTACCTACCCAAAGGGTTTCCGGGGCTATTGTAATGGTCTGATGTCCTATATGC
GGGGCACGATGCAAAATTCAGATTGCCCAGAGATCCATTATGTGATACATGCCCCTTGGAAGGCCATCCAGAAGTTCTGCAAGTATAGT
GACAGCTTCTGTGAGAATTACAATGAATACTGCACACTCACCCAGGATTCCTTCCCCATCACGGTCTGCTCCCTGAGCCACCAACAGCC
ACCCACTAGCTGCTACTACAATAGCACCCTAACCAACCAGAAGCTCTACCTACTTTGCTCCCGCAGGTATGAAGCTGATCCAATAGGTA
TCGCTGGTCTGTATTCAGGAATTTAA

>Pp-RNase14psA
ATGAATCTGATGTGGACCCTCCTCCTTTTCCTCCTTTTGGACCTAACTGTCTTGACTCCAGCCCTGTCCTTCTCAACACGACATATAGA
CAACCCCAGTTCTTGGGTCCCTAGAGGACACCACCGATACTGTGATGTGATGATGAGGCGCCGTTGGCTGATCTATAGGGGTAAATGCA
AGCAGATCCACACATTCATTCATAGAATCTGACCACCATAGCAGATTTCTGCAGAACTCCACCACTGCCCTGTACCAACAGCCCCTCCA
TGTGCAGCTGCCACAACAGTACTCATGATGTCAATATCACTGACTGCTTTGCCAGCACAGGGATCTGACCTCTTCACTGCCACTACCAA
AAATAAGGAGTCCACGAGGCCCATGCGAGTGGGCTGCAAGAAGGGGGCACCTGTTCACCTGGATGGCTAG

>Pp-RNase14psB
ATGAATCTGATGTGGACCCTCCTCCTTTTCCTGCTTTTGGACCTAACTGTCTTGACTCCAGCCCTGCCCTTCTCGACACGACATATAGA
CAACCCCAGTTCTTGGGTCCCTAGAGGACACCACCGATACTGTGATGTGATGATGAGGCGCCGTTGGCTGATCTATAGGGGTAAATGCA
AGCAGATCCACACATTCATTCATAGAATCTGACCACCATAGCAGATTTCTGCAGAACTCCACCACTGCCCTGTACCAACAGCCCCTCCA
TGTGCAGCTGCCACAACAGTACTCATGATGTCAACGTCACTGACTGCTTTGCCAGCACAGGGACCTGACCTCTTCACTGCCACTACCAA
AAATAAGAAGGAGTCCACCAGGCCCATGCGAGTGGGCTGCAAGAAGGGGGCACCTGTTCACCTGGATGGCTAG

>PpRNase14psC
ACGAATCTGATGTGGACCCTCCTCCTCTTCCTCCTTTTGGATCTAACTGTCTTCACTCCAGCCCTTTGGTCCCTAGAGGACACCACCGA
TACTGTGATGTGATGATGAGGCGCCGTTGGCTGATCTATAGGGGTAAATGCAAGCCGATCCACGCATTCATTCATGAGAATCTGACCAC
CATAGCAGATTTCTGCAGAACTCCACCACTGCCCTGTACCAACAGCCCCTCCATGTGCAGCTGCCACAACAGTACTCATGATGTCAATA
TCACTGACTGCTTTGCCAGCACAGGGACCCGACCTCTTCACTGCCACTACCAAAAATAAGAAGGAGTCCAACAGGCCCATGTGAGTGGG
CTGCAAGAAGGGGGCATCTGTTCACCCGGATGGCTAG

>Pp-RNase15ps
ATGGTTCTGACGGGGACCCTCCTGCGTCTGCTGTTACATCAAACTGTTTCCCCCAGGCCCAGGACTTATACAGAAGCCGTCTGATTACC
TGAGGTGCAGAATCTCAGGTGGGTCCAGCCACTATTGTGATGTGGTAATGTAGCATAGGGGAATGGTCTCCAGAGTCTACAAGCTCATC
AACACCTTCATTCACCAGAGCATCAGAAGGATAAATGGCATATGCAAGGCCATATCTACTTTCGGAACCTGACAGTGGACAACTGCTAC
CTCAGTCTCCGAACACTTGAGCTCACTCACTGTGTGATTTCTGTGGGCTCCTGACCCCCTAATTTCCGTTACAGGTTTAGGATGTTCTC
CCAGCACATCCACTTGACCTGTGTGTGGAACCAGCTAGTTCATCTGGAGGGCTAG

Gibbon (*Nomascus leucogenys*)

>Nl-RNase1
ATGGCTCTGGAGAAGTCTCTTGTCCTGCTCCCCCTGTTTGTCCTGATGCTGCTGGTGCTGGGCTGGGTCCAGCCTTCCCTGGGCAAGGA
ATCCCGGGCCAAGAAATTCCAGCGGCAGCACATGGACTCAGACAGTTCCCCCAGCAGCAACTCCACCTACTGCAACCAAATGATGAGGC
GCCGGAATATGACACAGGGGCGGTGCAAACCAGTGAACACCTTTGTGCACGAGCCCCTGGTAGATGTCCAGAATGTCTGCTTCCAGGAA
AAGGTCACCTGCAAGAACGGGCAGGCCAACTGCTACAAGAGCAACTCCAGCATGCACATCACAGACTGCCGCCTGACAAACGGCTCCAG
GTACCCCAACTGTGCATACCGGACCAGCCCGAAGGAGAGACACATCATTGTGGCCTGTGAAGGGAGCCCATATGTGCCAGTCCACTTCG
ACGCTTCTGTGGAAGACTCCACCTAA

>Nl-RNase2
ATGGTTCCAAAACCGTTCACTTCCCAAATTTGTCTGCTTCTTCTGTTGGGGCTTATGGGTGTGGAGGGCTCACTCCATGCCAAACCCCG
ACAGTTTACCTGGGCTCAGTGGTTTGAAATCCAGCACATCAATATGACCTCCCAGCAATGCACCAATGCAATGCGGGTCATTAACAATT
ATCAACGGCGATGCAAAAACCAAAATACTTTTCTTCGTACCACTTTTGCTAATGTAGTTAATGTTTGTGGTAACCCAAATATGACATGT
CCTAGTAACAAAACTCGTAAAAATTGTCATCAAAGTGGAAGCCAGGTGCCTTTAATCCACTGTAACCTCACAACTCCAAGTCCACAGAA
TATTTCAAACTGCGGGTATGCGCAGACACCAGCAAACATGTTCTATATAGTTGCATGTGACAACAGAGATCAACGACGGGACCCTCCAC
AGTATCCAGTAGTTCCAGTTCACCTGGATAGAATCATCTAA

>Nl-RNase4
ATGGCTCTGGCGAGGACCCATTCATTCCTTCTGCTTTTGCTGCTGACCCTGCTGGGGCTGGGGCTGGTCCAGCCCTCCTATGGCCAGGA
TGGCATGTACCAGCGATTCCTGCGGCAACATGTGCACCCTGAGGAGACAGGTGGCAATGATCGCTACTGCAACTTGATGATGCAAAGAC
GGAAGATGACTTTGTATCACTGCAAGCGCTTCAACACCTTCATCCATGAAGATATCTGGAACATTCGTAGTATCTGCAGCACCACCAAT
ATCCAATGCAAGAATGGCAAGATGAACTGCCATGAGGGTGTAGTGAAGGTCACAGACTGCAGGGACACAGGAAGTTCCAAGGCACCCAA
CTGCAGATATCGGGCCATGGCGAGCATTAGACCTGTTGTCATTGCCTGTGAGGGTAACCCACAGGTGCCTGTGCACTTTGACGGTTAG

>Nl-RNase6
ATGGTGCTACGCTTTCCTCTTCTTTTATTGCTGCTGGTTCCATGGGGACCAGTGTGTCTGCTTCATGCTTGGCCTAAGCATCTCACCAA
GGCTCACTGGTTTGAAATTCAGCATATACAGCCAAGTCCTCTCCAATGCAACAGGGCAATGAGTGGCATCAACAATTACACCCAGCACT
GTAAGCATCAAAATACCTTTCTGCATGACTCTTTCCAGAATGTGGCTGCTGTCTGTGATTTGCTCAGCATTGTCTGCAAAAATCGTTGG
CACAACTGCCACCAGAGCTCAAAGCCTGTCAACATGACTGACTGCAGACTCACTTCAGGAAAGTATCCCCAGTGCCGCTATAGTGCTGC
TGCCCAGCACAAATTCTTCATTGTTGCCTGTGACCCCCCTCAGAAGAGCGACCCCCCCTACAAGTTGGTTCCTGTACACTTAGATAGTA
TTGTCTAA

>Nl-RNase7
ATGGCACCAGCCAGAGCAGGATTCTGTCCCCTTCTGCTGCTTCTGCTGCTGGGGCTGTGGGTGGCAGAGATCCCAGTCAATGCCAAGCC
CAAGGGCATGACCTCATCTCAGTGGTTTAAAATTCAGCACGTGCAGCCCAGCCCTCAAGCATGCAACTCAGCCATGAAAAACATCAACA
AGCACACAAAACGGTGCAAAAACGTCAACACCTTCCTGCAAGAGCCTTTCTCCAGTGTGGCCACCACCTGCCAGACCCCCAAAATAGCC
TGCAAGAATGGCGATAAAAACTGCCACCAGAGCCGCGGGCCAGTGTCCCTGACCATGTGTGAGCTCACCTCAGGGAAGTATCCGAACTG
CAGGTACAAAGAGAAGCGACAGAACAAGTCTTACATAGTGGCCTGTAAGCCTCCCCAGAAAAAGGACTCTCAGCAATTCCACCTGGTTC
CTGTGCACTTGGACAGAGTCCTTTAG

>Nl-RNase8ps
CTGTCTCCTTTAAGAGAGCAGGATGCTGCCCCCTGCTGCTGCTGCTTCTGGGGCTGTGGGTGGCAGAGATCCCAGTCAGTGCCAAGCCC
AAGGACATGACATCATCTCAGTGGTTTAAAACTCAGCATGTGCAGCCCAGCCCTCAAGCATGCAACTCAATCATGAGCAACATCAATAA
GTACACAGAACAGTGCAAAGACCTCAACACCTTCCTGCACGAGCCCTTCTCCAGTGTGGCCACCACCTGCCAGACCCCCAACATAGCCT
GCAAGAATAGCCATAAAAATTGCCACCAGAGCCACGGGCCCGTGTCCCTGACCATGTGCGAGCTCACCTCAGGGAAGTACCCAAACTGC
AGGTACAAAGAGAAGCACCTGAACCCACCTTACATATTGGCCTGTGACTTTCCACAACAGGGTGACCCAGGGTACCCGCTTGTTCCTGT
TCACTTGGATAAAGTTGTCTAA

>NlRNase9
ATGATGAGAACTCTGATCACCACACACCCACTGCCCCTGCTTCTATTGCTGCAGCAGCTGCTGCAGCCAGTGCAGTTTCAAGAGGTGGA
TACAGATTTTGATTTCCCAGAAGATAAAAAAGAAGAATTTGAAGAGTATTTGGAACAATTTTTTAGTACAGGGCCCACCAGACCACCTA
CCAAAGAAAAAGTCAAAAGACGTGTCCTTATTGAACCTGGAATGCCATTAGATCATATAGACTACTGTAATAGTGAAATCATGAGAAAA
AATGTTTACTACAAACACCGTTGTGTGGCGGAACATTACTTCCTTCTTATGCAATATGACGAGCTCCAAAACATCTGTTACAACAGATT
TGTGCCATGTAAGAATGGAATTAGGAAATGTAACAGGAGCAAAGGTCTTGTAGAAGGAGTGTATTGTAATTTAACAGAAGCATTTGAAA
TTCCAGCGTGTAAATACGAATCATTTTATAGGAAGGGCTATGTCCTTATCACTTGTGCATGGCAAAATGAACTACAAAAACTGATTCCT
CATACTATAAATGACCTCGTGGAGCCAACTGAACACAGAAGTTTCCTCGGTGAGGATGGTGTCTTTGTCATACCGCCCTAG

>Nl-RNase10
ATGAAGCTGAATCTGGTGCAGATCTTTTTCATGTTGCTGATGCTGCTGCTGGGCCTGGGGATGGGCCTGGGGTTGGGGCTTCATATGGC
TGCAGCAGTCTTGGAGGAGAGTGATCAACCGCTCAATGAATTTTGGTCCAGTGACTCACAGGACAAAGCTGAGGCCACTGAGGAGGGAG
AGGGCACCCAAACCACAGAAACGCTGGTGCTTGGCAACAAAGAAGTGGTGCAACCTGGCTGGCCAGAAGATCCCATCCTCAGTGAAGAT
GAGGTTGGAGGAAACAAGATGCTCAGAGCCTCAGCTCTCTTTCAGAGCAACAAAGACTATCTTAGGCTTGACCCGACAGACAGAGAATG
CAATGATATGATGGCACACAAGATGAAGGAGCCCAATCAGAGTTGCATAGCCCAGTATGCATTCATCCATGAGGATCTAAACACAGTCA
AAGCTGTCTGTAACAGTCCTGTCATTGCCTGTGAGCTCAAGGGGGGAAAATGTCACAAAAGCTCCCGACCTTTTGATTTGACATTGTGC
GAGCTGTCCAAACCAGACCAGGTCACTCCTAACTGCAATTACCTAACTTCTGTTATAAAAAAGCACATTATTATATCCTGTAATGACAT
GAAGCACCAGTTACCAACTGGACAATGA

>Nl-RNase11
ATGGAGACCTTTCCTCTGCTGCTGCTCAGCCTGGGGCTGGTTCTTGCAGAAGCATCAGAAAGCACAATGAAGATAATTAAAGAAGAATT
TACAGAGAAAGAGATGCAATATGACATGGCAAAAAGTGGCCAAGAGAAACAGACCATTGAGATATTAATGAACCCAGTCCCGTTAGTTA
AAAATACTAGCCTCAGCATGTCCAAGGATGATATGTCTTCCTCATTACTGACATTCAGAAGGTTACATTATAATGACCCCAAGGGAAAC
AGTTCAGGTAATGACAAAGAGTGTTGCACAGTCTGGAGAAAAGTTTCAGAAGCAAATGGATCATGCAAGTGGAGCAATAACTTCATCCA
TGGCTCCACGGAAGTGATGCGCAGGGTCCACAAGGCCCCCAGCTGCAAGTTTGTACAGAATCCTGGCATAAGCTGCTGTGAGAGCCCAA
AACTGGAAAATACAGTGTGCCAGCTCACCACAGGCAAACAATTCCCCAGGTGCCAATACCATAGTGTTACCTCATTAGAGAAGATATTG
ACAGTGCTGACAGGTCATTCTCTGATGAGCTGGTTAGTTTGTGGCTCTAAGCTGTAA

>Nl-RNase12
ATGATAATAATGGTGATAATTTTCTTGGTGCTTCTGTTCTGGGAAAATGAGGTGAATGATGAAGTAGTGATGTCAACCTTAGAACACTT
GCATGTGGACTACCCTCAGAATGACGTTCCCGTTCCTGCAAGGTACTGCAACCACATGATCATACAAAGAGTTATCAGGGAACCTGACC
ACACTTGTAAAAAGGAGCATGTCTTCATCCATGAGAGGCCTCGAAAAATCAATGGTATTTGCATTTCTCCCAAGAAGGTTGCTTGCCAA
AACCTTTCAGCCATTTTCTGCTTTCAGAGTGAGACAAAGTTCAAAATGACAGTCTGTCAGCTCATTGAAGGCACAAGATACCCTGCCTG
CAGGTACCACTATTCCCCCACAGACGGGTTTGTTCTTGTCACTTGTGATGACTTGAGGCCAGATAGTTTCCTTGGCTATGTTAAGTGA

>Nl-RNase13
ATGGCACCAGCTGTGACCCGGCTCCTTTTCCTCCAGCTTGTTCTAGGGCCAACTCTGGTCATGGACATCAAGATGCAGATTGGCAGCAG
GAACTTCTATACCTTAAGCATTGACTATCCCAGGGCTAACTACCCAAAGGGTTTCCGGGGCTATTGTAATGGTCTGATGTCCTATATGC
GGGGCAAGATGCAAAATTCAGATTGCCCAAAGATCCATTATATGATACATGCCCCTTGGAAGGCCATCCAGAAGTTCTGCAAGTATAGT
GACAGCTTCTGTGAGAATTATAATGAATACTGCACACTCACCGAGGATTCCTTCCCCATCACGGTCTGCTCCCTGAGCCACCAACAGCC
ACCCACTAGCTGCTACTACAATAGCACCCTAACCAACCAGAAGCTCTACCTACTCTGCTCCCGCAAGTATGAAGCTGATCCAATAGGTA
TCGCTGGTCTCTATTCGGGAATTTAA

>Nl-RNase15ps
ATGGTTCTGATGGCGACCCTCCTGTGTCTGCTGCTACATCAGACTGTTTCCCCCAGGCCCAGGACTTATACAGAAGCCGTCTGATTACC
TAAGGTGCAGAATCTCAGGTGGGTCCAGCCACTATTGTGACGTGATAATGTAGCATAGGGGAATGGTCTCCAGAGTCTGCAAGCTCATC
AACACCTTCATTCACCAGAGCATCAGAAGGATAAATGACATATGCAAGGCCATGTCTATTTTCGGAACCTGACAGTGGACAACTGCTAC

CTCTCAGTCTCCGAACACTTGAGCTCACCCAGTGTGTGATTTCTGTGGGCTCCTGGCCCCCTAATTTCCGTTACAGGTTTAGGGTGTTC
TCCCAGCACATCCACTTGACCTGTGTGTGGAACCAGCCAGTTCATCTGGAGGGCTAG

Rhesus monkey (*Macaca mulatta*)

>Mmu-RNase1
ATGGCTCTGGATAAGTCTGTCATCCTGCTCCCTCTGCTTGTCCTGGTGCTGCTGGTGCTGGGCTGCCTGGGCAGGGAATCCCGGGCCAA
GAAATTCCAGCGGCAGCACATGGACTCAGGCAGTTCCCCCAGCAGCAACTCCACCTACTGCAACCAAATGATGAAGCGCCGGAGTATGA
CGCACGGGCGGTGCAAACCAGTGAACACCTTTGTGCATGAGCCCCTGGTAGATGTCCAGAATGTCTGCTTCCAGGAAAAGGTCACCTGC
AAGAACGGGCAGACCAACTGCTTCAAGAGCAAGTCCAGCATGCACATCACAGACTGCCGCCTGACAAACGGCTCCAGGTACCCCAACTG
TGCATACCGGACCAGCCCGAAGGAGAGACACATCATTGTGGCCTGTGAAGGGAGCCCACATGTGCCAGTCCACTTCGATGCTTCTGTGG
AGGACTCAACCTAA

>Mmu-RNase2
ATGGTTCCAAAACTGTTCACTTCCCCAATTTGTCTGCTTCTTCTGTTGGGGCTTATGGGTGTGGAAGGCTCACTTCATGCCAAACCCGG
ACAATTTACCTGGGCTCAGTGGTTTGAAATCCAGCATATAAATATGACCTCTGGCCAATGCACCAATGCAATGCTGGTAATTAACAATT
ATCAACGGCGATGCAAAAATCAAAATACTTTTCTTCTTACAACTTTTGCTGATGTAGTTCATGTCTGTGGTAACCCAAGCATGCCCTGC
CCTAGCAACACAAGTCTCAACAATTGTCATCATAGTGGAGTCCAGGTGCCTTTAATCCACTGTAACCTCACAACTCCAAGTCGAAGGAT
TTCAAATTGCAGGTATACACAGACAACAGCAAACAAGTACTACATAGTTGCATGTAACAACAGCGATCCAGTACGGGACCCTCCACAGT
ATCCAGTGGTTCCAGTTCACTTGGATAGAGTCATCTAA

>Mmu-RNase3A
ATGGTTCCAAAACTGTTCACTTCCCCAATTTGTCTGCTTCTTCTGTTGGGGCTTATGGGTGTGGAGGGCTCACTCCATGCCAGACCCCC
ACAGTTTACGAAGGCTCAGTGGTTTGCCATCCAGCACATCAATGTGAACCCCCCTCGATGCACCATTGCAATGCGGGTAATAAATAATT
ATCAACGGCGTTGCAAAAACCAAAATACTTTTCTTCGTACAACTTTTGCAAATACAGTTAATGTTTGTCGTAACCGAAGTATACGCTGC
CCTCATAACAGAACTCTCCACAATTGTCATCGTAGTAGCTACCGGGTGCCTTTACTCCACTGTGACCTCATAAATCCAGGTGCACAGAA
TATTTCAACCTGCAGGTATGCAGACAGACCAGGACGGAGGTTCTATGTAGTTGCATGTGAAAGCAGAGATCCACGGGATTCTCCACGGT
ATCCAGTGGTTCCAGTTCACCTGGATACCATCATCTAA

>Mmu-RNase3B
ATGGTTCCAAAATTGTTCACTTCCCAAATTTGTCTGCTTCTTCTGTTGGGGCTTATGGGTGTGGAGGGCTCACTCCATGCCAGACCCCC
AGAGTTTACAAGGGCTCAGTGGTTTGCCATCCAGCACATCAATATGAACCCCCCTTCATGCAACATTGCAATGCGGGTAATTAACAAGT
ATCAACGGTGTTGCGAAAAGCAAAATACTTTTCTTCGTACAACTTTTGCAAATGTAGTTAATGTTTGTCGTAACCCAAGTATACCCTGC
GTTCATAACAGAACTCTCCAAAATTGTCATCATAGTAGCGTCCAGGTGCCTTTACTCCAGTGTGAACACATAAATAGAGGTGCACAGAA
TGTTTCAACCTGCGAGTATGCAGACAGACAAGGACAGAGTTTCTATGTAGTTGCATGTGAAAACAGAGATCCACGGGATCCTCCACAGT
ATCCAGTGGTTCCAGTACTCCTGGACAGAATCATCTAA

>Mmu-RNase4
ATGGCTCTGCAGAGGACCCATTCATTGCTTCTGCTTTTGCTGCTGACCCTGCTGGGGCTGGGGCTGGTCCAGCCCTCCTATGGCCAGAA
TGGCATGTACCAGCGATTCCTGAGGCAACATGTGCACCCTGAGGAGACAGGTGGCAATGATCGCTACTGCAACATGATGATGCAAAGAC
GGAAGATGACTTTGTATCATTGCAAGCGCTTCAACACCTTCATCCATGAAGATATCTGGAACATTCGTAGTATCTGCAGCACCACCAAT
ATCCAATGCAAGAACGGCAAGATGAACTGCCATGAGGGTGTAGTGAAGGTCACAGACTGCAGGGATACAGGAAGTTCCAAGGCACCCAA
CTGCAGATATCGGGCCATGGCGAGCACTAGACGTGTTGTCATTGCCTGCGAGGGTAACCCACAGGTGCCTGTGCACTTTGACGGTTAG

>Mmu-RNase5
ATGGTGATGGGCCTGGGCCTTTTCTTGTTGGTCTTCATGCTGGGTCTGGGTCTGACCCCACCCACCCTGGCTCAGGATAACCCCAGGTA
CAGAGACTTCCTGGCCAAGCACTATGATGCCACACCACAGGGCCGGAATGACAGATACTGTGAAAGCACAATGAGGAGACGGCATCTGA
CCTCACCCTGCAAAGACATCAACACCTTTGTTCATGGCAACAGGCACCACATCACGGCCATCTGTGGAGATGAGAATGGAAGCCCTTAT
GGAGGAAACCTAAGAATAAGCACGTCTCCTTTCCAGGTCACCACTTGCAAACTACGTGGAGGATCCCCCCGGCCTCCATGCCAGTACCG
AGCCACACGGGGGTCCAGAAACATTGTTGTTGGTTGTGAAAATGGCCTACCTGTCCACTTGGATGAGTCCATTTTCCGTCCGTAA

>Mmu-RNase6
ATGGTGCTACGCTTTCCTCTTCTTTTATTGCTGCTGGTTCTATGGGGACCAGTGTGCCTACTTCATGCTTGGCCTAAGCATCTCACCAG
GGCTCATTGGTTTGAAATTCAGCATATACAGCCAAGTCCTCTCCAATGCAACAGGGCAATGAGTGGCATCAACAATTACACTCAGCACT
GTAAGCATCAAAATACCTTTCTGCATGACTCTTTCCAGAATGTGGCTGCTGTCTGTGATTTGCTCAGCATCATCTGCAAAAATCGTCAG
CACAACTGCCACCAGAGCTCAAAGCCTGTCAACATGACTGACTGCAGACTCACTTCAGGAAAGTACCCCCAGTGCCGCTATAGTGCTGC
CGCCCAGTACAAATTCTTCATTGTTGCCTGTGACCCCCCTCAGAAGAGCGACCCCCCCTACAAGTTGGTTCCTGTACACTTAGATAGTA
TTGTCTAA

>Mmu-RNase7
ATGGCACCGGCCAGAGCAGGATTCTGCCCCCTTCTGCTGCTTCTGCTGCTCGGGCTGTGGGTGGCAGAGATCCCAGTCAGTGCCAAACC
CAAGGGCATGACCCCATCTCAGTGGTTTCAAATTCAGCACGTGCAGCCCAGCCCTCAAGCATGCAACTCAGCCATGAAAAACATCAACA
AGCACACAAAACGGTGCAAAGACCTCAACACCTTCCTACACGAGCCTTTCTCCCGTGTGGCCACCACCTGCCAGACCTCCAACAAAGCC
TGCAAGAATGGCGATAAAAACTGCCACCAGAGCCATGGAGCCGTGTCCCTGACCATGTGTCAGCTCACCTCAGGGAAGTACCCGAACTG
CAGGTACAAAGAGAAGCGCCAGAACAAGTCTTACATAGTGGCCTGTAAGCCTCCCCAGAAAAAAGACTCTCAGCAATTCCACCTGGTTC
CTGTGCACTTGGACAGAGTCCTTTAG

>Mmu-RNase8
ATGGCACCGGCCAGAGCAGGATGCTGCCCCTTGCTGCTGCTGCTTCTGGGGCTGTGGGTGGCAGAGATCCCAGTCAGTGCCAAGTCCAA
GGACGTGACCTCATCTCAGTGGTTTAAAACTCAGCATGTGCAGCCCAGCCCTGAAGCATGCTACTCAGCCATGAGCAACATCAGTAAGT
ACACAGAATGGTGCAAAGACCTCAACACCTTCCTGCATGAGCCCTTCTCCGGTGTGGCCACCACCTGCCAGACCCCCAACATAGCCTGC

AAGAATCGCCATAAAAACTGCCCCCAGAGCAGCGGGCCCGTGTCCCTGACCATGTGTGAGCTCACTTCAGGGAAGTACCCAAACTGCAG
GTACAAAAAGAAGCACCTGAACGCACCTTACATAGTGGCCTGTGACCCTCCACAACAGGGTGACCCAGGGTACCCACTTGTTCTGTGCA
CTTGGATAAAGTTGTCTAA

>Mmu-RNase9A
ATGATGAGAACTCTGATCACCACACACCCACTGCTCCTGCTTCTATTGCTGCAGCAGCTGCTGCAGCCAGTGCAGTTTCAAGAGGTGGA
TACAGATTTCGATTCCCCAGAAGATAAAATGGAAGAATTTCGAGAGTATTTGGAAGAATTTCGTAGGACAGGGCCCACCAGACCACCTA
CCAAAGAAAAAGTCGAAAGACGTGTCATTATTGAACCTGGAATGCCATTATATCATAGGGACTACTGTAATGAAGAAATCATGAGAAAA
AATGTTTACCACAAACAGCGTTGTGTGACAGAACATTACTTTCTCCTTATGCAATATGACGAGCTGGAAAAAATCTGTTACAACAGATT
TGTGCCATGTAAGAATGGAGTTAGGAAATGCAACAGGAGCAAGGGTCTTGTAGAAGGAGTGTATTGTAATTTAACAGAAGCATTTAAAA
TACCGAGGTGTAAATATAAGTCATTTTATAGGAGGGGCTATGTCCTTATCACCTGTGCATGGCAAAATGAAATACACAAACTTATTCCT
CATACTATAAATGATCTCGTGGAGCCACCTAAACACAGAAGTTTCCTCAATGAGGATGGTGTCTTTGTCATACTGCCCTAG

>Mmu-RNase9B
ATGATGAGAACTCCGATCACCACATACCCACTGCTCCTGCTTCTATTGCTGCAGCAGCTGCTGCAGCCAGTGCAGTTTCAAGAGGTGGA
TACAGATTTTGATTCCCCAGATGATGATATGGAAGAATTTGAAGAGTATTTGGAAGAATTTCATAGGACAGGGCCCACCAGACCACCTA
CCAAAGAAAACGTCAAAAGACGTGTCATTATTGAACCTGGAATGCCATTATATGATAGGGAGTACTGTAATGCAGAAATCATGAGAAAA
AATGTTTACCACAAACAGCGTTGTGTGACAGAACATTACTTCCTCCTTATGCAATATGACGAGCTGGAAAAAATCTGTTACAACAGATT
TGTGCCATGTAAGAATGGAGTTAGGAAATGCAACAGGAGCAAGGGTCTTGTAGAAGGAGTGTATTGTAATTTAACAGAAGCATATGAAA
TACCGTGGTGTAGATATAAATCATTTTATAGGAGGGGCTATGTCCTTATCACCTGTGCATGGCAAAATGAAATACACAAACTTATTCCT
CATACTATAAATGATCTCGTGGAGCCACCTAAACACAGAAGTTTCCTCAATGAGGATGGTGTCTTTGTCATACCGCCCtag

>Mmu-RNase10
ATGAAGCTGAATCTGGTGCAGATCTTGTTCATGTTGCTGATGCTGATGCTGGGCCTGGGGATGGGCCTGGGGCTGGGGCTTCAAATGGC
TGCAGCAGTCTTGGAGGAGAGTGATCAACCGCTCAATGAATTTTGGTCCAGTGACTCACAGGACGAAACTGAGGCCACTGAGGAGGGAG
ATGGCACCCAAACCACAGAAACGCTGGTACTTGGCAACAAAGAAGTGGTGCAACCTGGCTGGCCGGAAGATCCCATCCTCAATGAAGAT
GAGGTTGGAGGAAACAAGATGCTCAGAGCCTCAGCTCTCTTTCAGAGCAACAAAGACTATCTTAGGCTTGACCAGACAGATAGAGAATG
CAATGAAATGATGGCACACAAGATGAAGGAGCCCAATCAGAGTTGCATAGCCCAGTATGCATTCATCCATGAGGATCTAAAGACAGTCA
AAGCTGTCTGTAACAGTCCTGTCATTGCCTGTGAGCTCAAGGGGGGAAAATGTCACAAAAGCTCCCGACCTTTTGATTTGACATTGTGC
GAGCTGTCCAAACCAGACCAGGTCACTCCTAACTGCAATTACCTAACTTCTGTTATAAAAAAGCACATTATTATAACCTGTAATGACGT
GAAACTCCAGTTACCCACTGGATAA

>Mmu-RNase11
ATGGAGACCTTTCCTCTGCTGCTGCTCAGCCTGGGGCTGGTTCTTGCAGAAGCATCAGAAAGCACAATGAAGATAATTAAAGAAGAATT
TACAGAGGAAGAGATGCAATATGACATGGCAAAAAGTGGCCAAGGAATACAGACCATTGAGATATTAATGAACTCGATCCTGTTAGTTA
AAAATACCAGCCTCAGCATGTCCAAGGATGATATGTCTTCCTCATTACTGACATTCAGAAGGTTACATTATAATGACCCCAAGGGAAAC
AGTTCAGGTAATGACAAAGAGTGTTGCAATGACATGATAGTCTGGAGAAAAGTTTCAGAAGCAAACAGATCGTGCAAGTGGAGCAATAA
CTTCATCCATGACTCCACAGAAGTGATGCACGGGGTCCACAAGGCCCCCAGCTGCAAGTTTGTACAGAATCCTGGCATAAGCTGCCGTG
AGAGCCCAGAACTGGAAAATACAGTGTGTCAGCTCACTGCAGACAAACAATTGCCCAGGTGCCAATACCATAGTGTTACCTCATTAGAG
AAGATATTGACAGTATTGACAGGTCATTCTCTGATGAGCTGGTTAGTTTGTGGCTCTAAGTTGTAA

>Mmu-RNase12
ATGATAATAATGGTGATAATTTTCTTGGTGCTTCTGTTCTGGGAAAATGAGGTGAACGATGAAGTAGTGACGTCAACCTTAGAACACTT
GCATGTGGACTACCCTCAGAATGATGTTCCCGTTCCTGCAAGGTACTGCACCCACATGATCATACAAAGAGTCATCAGGGAACCTGACC
ACACTTGTAAAAAGGAGCACGTCTTCATCCATGAGAGGCCTCGAAAAATCAATGGTATTTGCACTTCTCCCAAGAAGGTAGCTTGCCAA
AACCTTTCGGCCATTTTCTGCTTTCAGAGTGAGACAAAGTTCAAAATGACAGTCTGTCAGCTCACTGAGGGCACAAGATACCCTGCCTG
CAGGTACCACTATTTCACCACAGAGGGGTTTGTTCTTGTCACTTGTGATGACTTGAGGCCAGATACTTTCCTGGGCTATGTTAAATAA

>Mmu-RNase13
ATGGCACCAGCTGTGACCCGGCTCCTTTTCCTTCAGCTTGTTCTAGGGCCAACTCTGGTCATGGACATCAAGACACAGATTGGCAAAAA
GAAATTCTATGCTTTAAACGTTGACTATCCCAGGGTTATCTTCCCAAAGAGTTTCAAGGGCTATTGTAATGGTCTGATGTCCTATATGC
GGGGCAAGGTGCAAAATTCGGATTGCCCAAAGATCCATTATGTGATACATGCCCCTTGGAAGGTCATCAAGAGGTTCTGCAAGTATAGT
GACAACTTCTGTGATAATTACAATGAATACTGCACGCTCACTGAGGATTCCTTCCCCATCACGGTCTGCTCCCTGAACTACCAACAGCC
ACCCACCAGTTGCTACTATGATAGCACCCTGACCAACCAGAAGCTCTACCTACTCTGCTCCCGCAAGTATGAAGCTGATCCAATAGGTA
TCATCGGTCTCTATTCGGGAGTTTAA

>Mmu-RNase14psA
GTGAATCTGATGTGGACCCTCCTCATTTTCCTCCTTTTGGACCTAACCGTCTTCACTCCAGCCTTCACCTTCTTAACACGACATATAGA
CAACTCCAGGTCTTGGGTCCCTAGAGGACACCACTGACACTGTGATGTGACGATGAGGTGCCGTTGGCTGATCCATAGAGGTAAATGCA
AGCAGATCCACACATTCATTCATGAGAATCTGACAGCCATAGCAGATTTCTGCAGAACTCCACCAGTGCCCTGTACCAACAGCCCCTCC
ATGTGCAGCTACCACAACAGGACTCATGATGTCAATGACACTGACTGTTTTGCCAGCACAGGGACCCGACCTCTTCACTGCCACTACCA
AAAATAAGAAGGAGTCCACCAGGCCCATGCGAGTGGGCTGTAAGAAGGGGGCACCTGTTCACCTGGATGGCTAG

>Mmu-RNase14psB
ATGAATCTGATGTGGACCCTTTTCCTCTTCCTCCTTTTGGACCTAACTGTCTTCACTCCAGCTCTGCCCTTCTCAACACGACATATAGA
TAACCCCAGGTCTTGGGTCCCTAGAGGACACCACTGACACTGTGATGTGACGATGAGGTGCCGCTGGCTGATCCATAGAGGTAAATGCA
AGCAGATCCACACATTCATTCATAAGAATCTGACCACCATAGCAGATTTCTGCAGAACTTCACCAGTGCCCTGTACCAACAGCCCCTCC
ATGTGCAGCTACCACAACAGGACTCATGATGTCAATGACACTGACTGTTTTGCCAGCACAGGGACCCGACCTCTTCACTGCCACTACCA
AAAATAAGGAGTCCACCAGGCCCATGCGAGTGGGCTGTAAGAAGGGGGCACCTGTTCACCTGGATGGCTAG

>Mmu-RNase15ps

TTGGTTCTGACGGGGACCCTTCTGAGTCTGCTGCTAGATCACACTGTTTCCCCCAGGCCTAGGACTTCTACAGATGCTGTCGTGACTAC
CTGAGGTGCAGAATCTCAGGTGGGTTCAGCCACTCTTGTGACGTGATAATGTAGCACAGGGTAATGTCTCCAGAGTCTGCAGGCCATCA
ACTCCTTCATTCACCAGAGCATCAGAAGGATAAATGGCATATGCAAGGCCATATCTACTTTCGGAATCTGACAGTGCACAACTGCTACC
TCAGTCTCCAAACACTTGAGCTCACCCAGTGTGTGATTTCTGTGGGTTCCTGCCCCCCTAATTTCTGTTACAGGTTTAGGATGTTCTCC
CAGCATATCCACTTGACTTGTGTGTGGAACCAGCCAGTTCATCTGGAGGGCTAG

Marmoset (*Callithrix jacchus*)

>Cj-RNase1
ATGGCTCTGGAGAAGTCCCTCGCCCTGCTCCCTCTGTTAGTCCTGGTGCTGCTGGTGCTGGGCTGGGCCAAGCCTTCCCTGGGCAAGGA
ATCCCGGGCCGAGAAGTTCCAGCGGCAGCACATGGACTCAGATGGTTCCCCCAGCAGCAACCCCACCTACTGCAACAACATGATGAGGC
GCCGGAATATGACACAGGGACGGTGCAAACCAGTGAACACCTTTGTGCACGAGCCCCTGGTAGATGTCCAGAATGTCTGCTTCCAGGAA
AAGGTCACCTGCAAGAACGGGCAGCCCAACTGCTACAAGAGCAGCTCCAGCATGCGTATCACCGACTGCCGCCTGACAAACGGCTCCAG
GTACCCCAACTGTGCATACCGGACCAGCCAGAAGGAGAGACACATCATTGTGGCCTGTGAAGGGAACCCGTATGTGCCCGTCCACTTCG
ATGCTTCAGTGGAGGGCTCCACCTAA

>Cj-RNase2/3
ATGGTTCCAAAACTGTTCACCTCTCAAATTTGTCTGCTTCTTCTGTTGGGGCTTTTGAGTGCAGAGGGCTCACCCCATGCCAGACCCCA
GCAGTATTCCAGGGCTCAGTGGTTTAGCATCCAGCACATCCAAACGGCTCCCCTCCACTGCACCTCTGCAATGCGGGCAATTAACAAGT
ATCAATCTCGATGCAAAAACAAAAATACTTTTCTTCATACAACTTTTGCTGATGTAGTTAATGTTTGCGGTAACACAAATATGACCTGC
CCTCATAATGCATCTCTCAACAATTGTCATCACAGTGGAGTCCAGGTGCCTTTAACCTACTGTAACCTCACAGGTCCACAGACTATTTC
AAACTGCGTGTATTCCTCGACTCAAGCAAACAAGTTCTATGTAGTTGCATGTGAAAACAGAGATCCACGGGACCCTCCACAGTATCCAG
TGGTCCCGGTTCACCTGGATACCATCATCTAA

>Cj-RNase4
ATGGCTCTGCAGAGGACCCATTCATTGCTTCTGCTTTTGCTGCTGACCCTAGTGGGGCTAGGGCTGGTCCAGCCCTCCTATGGCCAGGA
TCGCATGTACCAGCGATTCCTGCGGCAACACGTGCACCCTGAGGAGATAGGTGGCAATGATGGCTACTGCAACTTGATGATGCAAAGAC
GGAAGATGACTTCGAATCATTGCAAGCGCTTCAACACTTTCATCCATGAAGATATCTGGAACATTCATAGTATCTGCAGCACCGCCAAT
ATCCAATGCAAGAATGGCAAGATGAACTGCCATGAGGGTGTAGTGAAGGTCACAGATTGCAGGGAGACAGGAAGTTCCAGGGTACCCAA
CTGCAGATATCGAGCCACGGCCAGCACTAGACGTGTTGTCATTGCCTGTGAGGGTAACCCAGGGGTGCCTGTGCATTTTGACAGTTAG

>Cj-RNase5
ATGGTGATGGGCCTGCACCTTTTGTTGTTGGTCTTCATTCTGGGTCTGGGTCTGACCCCACCAACTCTGGCTCAAAATGACATCAGGTA
CATCAGGTTCCTGGAAGAGCACTATGATCCTAAAACAAGGAATGGGAATGACAGATACTGTGAAAAAATGATGAGGTTAAGGAACATGA
TCTCACCCTGCAAAGGAACCAACACCTTTATTCATGGCAACAAGGAGAGCATCAAGGCCATCTGTGGAACTGAGAATGGAGAGTCTTAC
AATGGAAACAAAAGAATAAGCAAGTCTGCTTTCCAGGTCACCATTTGCAAGCATAGAGGAGGGTCCCCCCGGCCTCCATGCCAGTACCG
AGCCACAGCGGGGTTCAGAAATGTTGTTGTTGCCTGTGAAAATGGCTTACCTGTCCACTTAGATGAGTCCATTTTCCGTCCGTAA

>Cj-RNase6
ATGGTGCAACACTTTCCCCTTCTTTTATTGCTGCTGGTTCTATGGGGACAAGTGTGTCCACTTCATGCTATACCTAAGAATCTCACCAA
GGCTCAATGGTTTGAAATTCAGCATATAAGGCCAAGTCCTCTCCAATGCAACAGGGCAATGAGTGGCATCAACAATTATACCCAGCACT
GTAAGCCTCAAAATACCTTTCTGCATGACTCTTTCCAGAATGTGACTGCTGTCTGTGAGTTGCTCAGCATCACCTGCAAAAATGGTTTG
CACAACTGCCACCAGAGCTTAAAGCCTGTCAACATGACTGACTGCAGACTCACTTCAGGAAATTATCCCCAGTGCCGCTACAGTACTGC
TGCCAAATACAAATTCTTCATCATTGCCTGTGACCCCCCTCAGAAAGACGATCCCCCCTACAAGTTGGTTCCTGTACACTTAGATCGTA
TTTTTTAG

>Cj-RNase7
ATGGTACCGGCCAGAGCAGGATACTTCCCGCTTCTGCTGCTTCTGCTGCTGGGGCTGTGGGTGGCTGAGATCCCAGTCAGTGTCAAGCC
CAAGGGCATGACCCCATCTCAGTGGTTTAACACTCAGCACGTGCAGCCCAGCCCTCAAGCGTGTGCCTCAGCCATGAGAAACATCAACA
AGCACACAAAACGGTGCAAAGACCTCAACACCTTCCTGCACAAGCCCTTCTCCAGTGTGGCCGCCACCTGCCAGACCCCCAACATAACC
TGCAAGAATGGCCATAAAAACTGCCACCAGAGCCATGGGCCCGTGTCCCTGACCATGTGTCGGCTCACCTCAGGGAAGTACCCGAACTG
CAGGTACAAAGAGAAGCACCTGAACAAGTCTTACATAGTGGCCTGTGACCCTCGCCAGAAAAAGGACCCTCAGCAATTCCACCTGGTTC
CTGTGCACTTGGACAGAGTCCTTTAG

>Cj-RNase8
ATGGCACTGGCCAGAGAAGGATGCTGCCCCCTGCTGCTGCTCCTGCTGCTGGAGCTGTGGGTGACTGAGATCCCAGTCAGTGCCAAGCA
GAAGGGCATGACCCCATCTCAGTGGTTTAAAACTCAGCACGTGCAGCCCAGCCCTCAAGCATGCGACTTAGCCATGAGAAACATCAATA
ACTACAAAAAAATGGTGCAAAGACCTCAACACCTTCCTGCACGAGCCCTTCTCCAGCGTGGCCGCCACCTGCCAGACCCCCAACATAACC
TGCAAGAATGGCCATAAAAACTGCCACCAGAGCCACGGGCCCGTGTCCCTGACCATGTGTGGGCTCACCTCAGGGAAGTACTTGAACTG
CAGGTACAAAGAGGAGCACCAGAACAAGTCTTACATAGTGGCCTGTGACCCTTTACAACAGGGTGACCCAGAGTACCCACTTGTTCCTG
TGCACTTGGATAAAGTTGTGTAA

>Cj-RNase9
ATGAGAATGCTGATCACCACATACTCACTGCCTCTGCTTCTATTGCTGCTGCAACCACTGCAGTTTCAAGAGGTGTATTATGAAGATTA
TTATTTGCCAGCATATAGAAGTACAGAAGGCTTTGAAGACTTTTGGGTAGAATTTCACAGTACAGGGCCCACCAGACCACCTAGCAAAG
AAAAAGTCAAAAGACGTATCCTTGTTAATCCTGGAATGCCATTAGGTGATAGTGGCTACTGTAATTATCAAATAATGAGAAAAAATGTT
TACTACAAACACAGTTGTGTGACAGAACATTACTTCCTTCTTATGCAATATGATGAGCTGGAAAAAACCTGTTACAACGGATTTGTGCC
ATGTAAGAATGGGATTAGGAAATGTAACAGGAGCAAGAATCTAGTAGAAGGAGTGTATTGTAATTTAACAGAAGCATCTGACATACCAA
TGTGTCGATACGAATCATTTTATAGGAGGGGATATGTCCTTATCACTTGCACATGGCAAAATGAAATACAAAAACTTATTCCTTATACT
ATAAATGATCTCGTGGAGCCACCTAAACACAAGTCTCCTTAATGA

>Cj-RNase10
ATGAAGTTGAATCTGGTGCAGGTCTTTTTTATGTTGCTGCTGCTGCTGCTGGGCCTGGGGATGGGCCTGGGGCTGGGGCTTCGTATGGC
TGCAGCAGTCTTGGAGGAGAGCGATCAGCCACTCAATGAATTTTGGTCCAGTGACTCACAGGACAAAGCTGAGGCCACTGAAGCAGGAG
AGAGCACTCAAACCACAGAAACCCTTGTGCTTAGCAACAAAGAAGTGGTGCAACCTGGCTGGCCAGAAGACCCCATCCTCAATGAAGAT
GAGGTTGGAGAAAAAGAGATGCTCAGAGCTGAGTCTCTCTTTCGGAACAACAAAGACTATCTTAGGGTTGACCAGACAGATAGAGAATG
CAATGATATGATGGCACACAAGATAAAGGAGCACAATCAGAGTTGCATAACCCAGCATGCATTCATCCATGAGGATCCAGACACGGTCA
AAGCTGTCTGTAACAGTCCTGTCATTGCCTGTGAGCTCAAGGGGGGAAAATGTCACAAAAGCTCCCGACCTTTTGATTTGACATTCTGC
GAGTTGTCCAAACCAGATCAGGTCACTCCTAACTGCAACTACCTAACTTCTGTTATAAAAAAGCACATTATTATAACCTGTAATGACAA
GAAGCTCCAGTCATCAATTGGACAATGA

>Cj-RNase12
ATGGTGATAATGGTGATCATTTTCTTGGTGCTTCTGTTCTGGGAAAATGAGATGAATAATGAAGTAGTGATATCAACCTTAGAACACTT
GCATGTGGACTACCCTCAGGAAGGTGTTCCTGTTCCGGCAAGGTACTGCAACCACATGATCATACAAAGAATCATCAGGGAGCCCGACC
ACACTTGTAAAAAGGAGCATGTCTTCATCCATGAGAGGCCTCGAAAAATCAATGGTCTTTGCACTTCTCCCAAGAAGGTAGCTTGTCAA
AACCTTTCATCCACTTTCTGCTTTCAGAGTGAGACAAAGTTCAAAATGACAGTCTGTCAGCTCATTGAAGGCACAAGATACCCTGCCTG
TAGGTACCGCTATTTCCCCGTAGAGGGGTTTGTTCTTATCACTTGTGATGAGTTGGGGCCAGATAGTTTCCAGGGATATATTAAATAA

>Cj-RNase13ps
ATGTCAACTATCCCAAGGTTATCCACCCAGATGGTTTCCAGGTCTATTGTAATGGTCTGATGGCCTATGTGCGGGACAAGATGCAAACT
TCACATTGCCCAAAGATCCATTATGTGATGCATGCCCCTTGGAAAGTCATCCAGAAGGTCTGCAAGCAAAGTGAGAGCTTCTGTGAGAA
CTACAATGAATACTGCACACTCACCAAGGATGCCTTCCCCATCACAGTCTGCTCCCTGAGCCACTGACAGCCACCCATCAGCTGCTACT
ACAATAGCACTGAAACCAACCAAAGGGCCCACCTACTCTGCTCCCGCAAGTATGAGGCTGAGCCAATAGATATCATTAGTCTCTATTAG

>Cj-RNase15ps
ATGGTTCTGAGTGGAATATCCTGCGCCTGCTACTAGATCAGACTGTTTCTTTCTGTCCCAGGACTTCTACAGATGCCCTCTTGACTACC
TGAGGTGCAGAATCCCAGGTGGGTTCAGCCACTATTGTGACGTGATAATGTAACATAGGGGAATGGTCTCCAGGGTCTGCAAGCCCATC
AACACCTTCATTCACCGGAGCATCAGAAGGATAAGTGGCGCCGGGCGCGGTAGCTCACGCCTATAATCCCAGCACTTTGGGAGGCCGAG
GCGGGTGGATCACGAGGTCAGGAGATCAAGACCATCCTGGTCAACAAGGTGAAACCCCGTCTTTACTAAAAATACAAAAATTAGCTGGG
CATGGTGGTGCGCACCTGTAGTCCCAGCTAAAACCCAGAAGGTGGAGGTTGCGGTGAGCCGA

Mouse (*Mus musculus*)

>Mm-RNase1 NM_011271
ATGGGTCTGGAGAAGTCCCTCATTCTGTTTCCATTGTTTGTCCTGCTGCTTGGATGGGTCCAGCCTTCCCTGGGCAGGGAATCTGCAGC
ACAGAAGTTTCAGCGGCAGCACATGGATCCAGATGGTTCCTCCATCAACAGCCCCACCTACTGCAACCAAATGATGAAACGCCGGGATA
TGACAAATGGGTCATGCAAGCCCGTGAACACCTTCGTGCATGAGCCCTTGGCAGATGTCCAGGCTGTCTGCTCCCAGGAAAATGTCACC
TGCAAGAACAGGAAGAGCAACTGCTACAAGAGCAGCTCTGCCCTGCACATCACTGACTGCCACCTGAAGGGCAACTCCAAGTATCCCAA
CTGTGACTACAAGACCACTCAATACCAGAAGCACATCATTGTGGCCTGTGAAGGGAACCCCTACGTACCAGTCCACTTTGATGCTACTG
TGTAG

>Mm-Ear1 NM_007894
ATGGGTCCGAAGCTGCTTGAGTCCCGACTTTGTCTCCTGCTGCTGCTAGGACTTGTCCTAATGCTTGCCTCATGCCTGGGTCAAACCCC
TTCCCAGAAGTTTGCCATCCAGCATATCAATAATAATACCAACCTCCAATGTAATGTTGAAATGATGCGTATTAACAGGGCTAGAAGAA
CATGTAAGGGCTTAAATACTTTTCTTCATACAAGTTTTGCTAATGCTGTTGGTGTGTGTGGAAATCCAAGTGGCTTGTGCAGTGACAAG
AGAAGTCAAAACTGTCATAATAGTTCATCTCGGGTACATATAACTGTCTGTAACATCACCAGTCGGGCAACAAATTATACCCAATGCAG
ATACCAATCAAGAAGATCATTGGAGTACTACACAGTTGCCTGTGACCCCAGAACTCCACAGGACAGTCCCATGTATCCAGTGGTTCCAG
TTCACTTGGATGGGACATTTTAG

>Mm-Ear2 NM_007895
ATGGGTCCGAAGCTGCTTGAGTCTCGACTTTGTCTCCTGCTGCTGCTAGGACTTGTCCTAATGCTTGCCTCATGCCTGGGACAAACCCC
TTCCCAGTGGTTTGCCATCCAGCATATCAATAATAATGCCAACCTCCAATGTAATGTTGAAATGCAGCGTATTAACAGGTTTAGAAGAA
CATGTAAGGGCTTAAATACTTTTCTTCATACAAGTTTTGCTAATGCTGTTGGTGTGTGTGGAAATCCAAGTGGCTTGTGCAGTGACAAT
ATAAGTAGAAACTGTCATAATAGTTCATCTCGGGTACGTATAACTGTCTGTAACATCACCAGTCGGAGGAGAACACCTTATACCCAATG
CAGATACCAACCAAGAAGATCATTGGAGTACTACACAGTTGCCTGTAACCCCAGAACTCCACAGGACAGTCCCATGTATCCAGTGGTTC
CAGTTCACTTGGATGGGACATTTTAG

>Mm-Ear5 NM_019398
ATGGGTCTGAAGCTGCTTGAATCCAGACTTTGTCTCCTGCTGCTGCTGGGACTTGTCCTAACGCTTGTCTCATGCCAGCGACCAACCCC
TTCCCAGAAGTTTGACATCCAGCATATCTATAAGAAATCCTCTCCCAAATGTGATGATGCAATGCGGGTCGTTAACAAGTATACAGGAA
AATGTAAGGACTTGAATACTTTTCTTCATACAACTTTTGCTGATGTTGTCCGTGTGTGTCACAATCCACCCAAGACTTGCAAAGACGGG
ACAAGTCCAAATTGTCATGATAGTTCATCTAAGGTATCTGTAACTATCTGTAAACTCACAAAACGGGCAAGGAATTATACCCACTGCAG
ATACAAAACAACAGGAGCAAAGAAGTCCTACACAGTTGCCTGTAACCCCAGAACTCCAAAGGACCGTCCCACCTATCCAGTAGTTCCGG
TTCACTTGGATCGGCTATTTTAG

>Mm-Ear6 NM_053111
ATGGGTCCGAAGCTGCTTGAGTCCCAACTTTGTCTCCTGCTGATGCTGGGACTTGTCCTAATGCTTGCCTCATGCCAGAAACCAACCGC
ATCCCAGTGGTTTGCTACCCAGCATATCACTTATAAAGCCAACCTCCAATGTAATGTTGAAATGCAGGCTATTAACATGCATAGACCAA
GATGTAAGGGCTTAAATACTTTTCTTCATACAAGTTTTATTAATGTTGTTGGTGTGTGTAGCAATCCAAGTGGCTTGTGCAGCGACAAA
ATAAGTCAAAACTGTCATAATAGTTCGTCTCGGGTACCTATAACTGTCTGTAACCTCACAACTCCGAGAAGAAATTATACCCAATGCAG
ATACCAAACAAAGGGATCAGTGGAGTACTACACAGTTGCCTGTGAGCCCAGAGTTGCATGGGATTGTCCCATCTATCCAGTGGTTCCAG
TTCACTTGGATGGGACATTTTAG

>Mm-Ear10 NM_053112
ATGGGTCCAAAGCTGCTTGAGTCCCGAATTTGCCTCCTGCTGCTGCTAGGACTTGTCCTAATGCTTGCCTCATGCCTGGGACAAACCAC
TTCCCAGTGGTTTGCCATCCAGCATATCAATAATAATGCCAACCTCCAATGTAATGTTGAAATGCAGCGTATTAACAGGTTTAGAAGAA
CATGTAAGGGCTTAAATACTTTTCTTCATACAAGTTTTGCTAATGCTGTTGGTGTGTGTGGAAATCCAAGTGGCTTGTGCAGTGACAAT
ATAAGTCAAAACTGTCATAATAGTTCATATCGGGTACATATAACTGTCTGTAACATCACCAGTTGGAGGAGAACACCTTATACCCAATG
CAGATACCAAGCTAAAAGATCATTGGAGTACTACACAGTTGCCTGTGACCCCAGAACTCCACAGGACAGTCCCATGTATCCAGTGGTTC
CAGTTCACTTGGATGGGACATTTTAG

>Mm-Ear11 NM_053113
ATGGGTCTGGAGCAACTTGAGTCTCGACTTTGTCTCCTGCTGCTGCTGGGACATGTCCTAATGCTTGCCTCATGCCAGCCATTGACCCC
CTCCCGGTGGTTTGACATCCAGCATATCTATAACAGAGCCTATCCCCGATGTGATGATGCAATGCGGGCCGTTAACAGTTACACAGGAG
TGTGTAAAGACATAAATACTTTTCTTCATACAACTTTTGCTAATGTTGTCCGTGTGTGTCATAATCCACGTAAGATCTGCAAAAATGGG
ATAAGTAGAAATTGTCATGATAGTTCAAATCGGGTACAAGTAACTATCTGTATACTCACAACTCCGGCCAGTCATTATTCCAACTGCAG
ATACCGAACAACAAGATCAATGAAGTACTACACAGTTGCCTGTGACCCCAGAACTCCTCAGGACAGTCCCATGTATCCAGTGGTTCCAG
TTCACTTGGATGGGATATTTTAG

>Mm-Ear14 AY665809
ATGAAGCTGCCTGAGTCCAGTCTTTGTCTCCTGCTGCTGTTGGGACTTGTCCTAATGCTTGCCTCATGCCAGGCACAAATCCTTTCCCA
GAAGTTTTACACCGAGTATATCTATAATAGCACCTACCCCCGATGTGATGCTGTAATGAGGGTTGTTAACAGGTATAGACCAAGATGTA
AGGACATAAATACTTTTCTTCACACAAGTTTTGCTGATGTTGTTGCTGTGTGTGGCCATCCAAATATCACCTGCAACAACTTGACAAGA
AAAAATTGTCATGCTAGTTCATTTCAGGTATTTATAACTTTTTGTAACCTCACTACGCCGACAAGAATATGCACACAATGCAGATACCA
AACGACAGGTTCAGTGAAGTACTACAGAGTAGCCTGTGAGAACAGAACTCCACAAGACACTCCCATCTATCCAGTGGTTCCAGTTCACT
TGGATGGGACATTTTAG

>Mm-Earps1 NG_001492
ATGGGTCTGAAGCTGCTTGAGTCCAGACTTTGTCTCCTGCTGCTGCCGGGACTTGTCCTAACAGTTGTCTCATGCCAGCCATCGACCCC
TTCCCAGAAGTTTGACATCCAGCATATCTATAAGAATGTCTGATGTGATAATGCAATGCTGTCTGTTAGCAGTTATACAAGAGTATGTA
AAGACTTGAATACTTTTCTTCATACAACTTTTGCTGATGTAATCCATGTGTGTAGCAATCCACATAAGACCTGCAGAGACGGGACAAGT
ACAAAATGTCATGATAGTTCATCTAAGGTACCTGTAACTATCTTTTCACTCACAACTTCAGCAAGCAGTTATGCACAATGCAGATGCAA
AACAACAAGAGCAAAGAAGTCCTACACAGTTGCCTGTGACCCCAGAACTCCACGGGACAGTCCCAGGTATCCAGTGGTTCCAGTTCACT
TGGATGGGACATTTTAG

>Mm-Earps2 NG_001503
ATGGGTCCAAAGCTGCTTGAGTCCCGAATTTGTCTCCTGCTGCTGCTAGGACTTGTCCTAATGCTTGCCTCATGCCTGGGACAAACCCC
TTCCCAGTGGTTTGCCATCCAGTATATCAATAATAATGCCAACCTCCAATGTAATGTTGAAATGCTGCGTATTAACAGGTCTAGAAGAA
CATGTAAGGGCTTAAATACTTTTCTTCATACAAGTTTTGCTAATGCTGTTGGTGTGTGTGGAAATCCAAGTGGCTTGTGCAGTGACAAT
ATAAGTCAAAACTGTCATAATAGTTCATCTCGGGTACATATAACTGTCTGTAACATCACCAGTCGGAGGAGAACACCTTAAACCCAATG
CAGATACCAAGCTAAAAGATCATTGGAGTACTACACAGTTGCCTGTGACCCCAGAACTCCACAGGACAGTCCCATGTATCCAGTGGTTC
CAGTTCACTTGGATGGGACATTTTAG

>Mm-Earps3 AY665810
GTGGGTCTGAAGCTGCTTGAGTCCCGAATTTGTCTCCTGCTGCTTCTGGGACTTGTCCTAATGCTTGCCTCATGCCAGCCACCAACCCC
TTCAAATCTAGCATTTCTATAATAGCGCCTATCCCCGATGTGATGATGCAATGAGGGTCATTAACACTTATACAAGAATGTGTAAAGAC
ACGAATACTTTTCTTCATACAACTTTGGCTGATGTTGTTGGTGTGTGTGGCAATCCAAATTCCACCTGCAAAGACGAGATAAGTAGAGA
TTGTCATGATAGTTCATCTCGGGTATCTGTAAATTTCCTCACAACTCCAGCAAGCCATTATATGGAATGGAGATACCAAGCGACACGAT
CAGTGAAGTAATACAGAGTGGCCTGTGAGAACAGAACTTCATTGGACACTCCCACCTACCCAGTATTTCTGGTTCACTTGGATGGGATA
TTTTTG

>Mm-Earps4 AY665811
ATGGGTTTGAAGCTGCTTGAGTCCCAACTTTGTCTCCTGTAGCTAGGAGTTGTCCTATCGCTTGCCTCATGCCAGCCACCAACCCCTTC
CTAGTAGTTTGAATTCCGGCATATCTATAATAGCACCTATACCCGATGTGATGGTGCAATGAGGGCCATTAACAGGTATACAGGAAGCT
GTAAGAACATGAATACTTTTCTTCATACAATTTTTCCTGATGTTCCTGGTGATGTGGCAAGCCAAATACCATCTGCAGAGATGGGATAA
GTAGAAATTGTTATGATAGTTCATCTCACGTACCTGTAACTATTGGCACCCACACAACTTCAGCAAGAAATGATAAGAAATGTAGATAC
CAAACAGTAGGAGCAAGAAAGTACTACACAGTTGCCTGGAACTCAGACAGTCCCACCAATCCAGTGGTTCTGATTCACTTGCATGGGAC
ACTTTAG

>Mm-Earps5 AY665812
CTGGCTGATAAGCTGGGTCTGGAAGTGCCATCCTGGAACCCTACAACTTCCAGCCTGAGGCAGAAGGATAGAAAATTAGAATCCAATCC
GAGACCAGCCCCTTCCCAGAAGTTTTACACCGAGCCTATCCATAATAGCACCTACCCCCGATGTGACGATCCAATGCTGGTTGTTAACA
GGTATAGACCAAGATGTAAGGATATAGATACGTTTCTTCACACAAGTTTTGCCAATGTTGGTGTGTGTGGCCATCCAAGTGGCTTCTGC
AAAGAGCATAAAAGTGCAAACTGTCATAACAGTTCATCTCAGGTACCTATAATTGTCTGTAACCTCACAACTCCAGGAAGAACTTATAC
CCAATGCAGATACCAAATGAAAGGATCAGTGGAGTACTACACAGTTGCCTGTAAGCCCAGAACTCCATGGGACAGTCCCATCTATCCAG
TGGTTCCGGTTCACTTGCATGGGACATTTTAG

>Mm-Earps6 AY665813
CTGACTCTGAAGCTGCCTGAGTCCAGTCTTTGTCTCCTGCTGCTGCTAGGACTTGTGCTAATGCTTGCCTCATGCCAGCAACCAACCCT
TCCCCAGAAGGTTTAGACCGAGTCTATCTATAATAGCACCTACCGCCTATGTGATGTTGCAATGCTGGTTGTTAACAGGTATAGACCAA
GATGTAAGGACATAGATATTTTTCTTCACACCAGTTTTGCTGATGTTGTTGGTGTGTGTGGCCATCCAAATATCACCTGCAACAACGTG
ACAAAAAATAACTGTCATACAAGTTTATTTCTGGTATCTGTAACTTTTTGTAACCTCACTACTCTGACAAGAATTTACACCCAATGCAG

ATACCAATGACAGGATCAGTGAAGTACAACAAAGTGGCCTGTAAGTCCAGAACTCCACAGGACACTCCTATCTATCCAGTGGTTCCAGT
TCACTTGGATGGGACATTTTAG

>Mm-Earps7 AY665814
GTGGTCATGAAGCTGCTTGAGTCCAGACTTTGCAGCTAGGAGTTGTCCTATTGGTTACCTCATGCCAGCCACCAACCCCTTCAAATCCA
GCATTTCTTTAATAGGGCCTATCCCCAATGTGATGATGCAGTGTGGGCCATTAACATTTATACAAGAATGTGTAAGGACATGAATACTT
TTCTTCATACAACTTTGGCTGATGTTGTTGGTGTGTGTGGCAATCCAAATAACACCTGCAAAGATGGGATGAGTAGAAACTGTCATGAC
AGTTCATCTTGGGTATCTGTAACTATCTGTAAACTCACAACTCCAGCAAGCCATTATATGGAATGCAGATACCAAATGACATGATCAGT
GAAGTACTACAGAGTGGCCTGTGAGAACAGAACTTCATGGGACAGTCCCACTTATCCAGTGGTTCTGGTTTACTGGGATAGGATAGTTT
AG

>Mm-Earps8 AY665815
CTGACTCTGAAGCTGCCTGAGTCCAGTCTTTGTCTCCTGCTGCTGCTAGGACTTGTGCTAATGCTTGCCTCATGCCAGCAACCAACCCT
TCCCCAGAAGGTTTAGACCGAGCCTATCTATAATAGCACCTACCGCCTATGTGATGTTGCAATGCTGGTTGTTAACAGGTATAGACCAA
GATGTAAGGACATAGATACTTTTCTTCACACCAGTTTTGCTGATGTTGTTGGTGTGTGTGGCCATCCAAATATCACCTGCAACAACATG
ATGAAAAATAACTGTCATACAAGTTCATTTCTGGTATCTGTAACTTTTTGTAACCTCACTACTCTGAAAAGAATTTACACCCAATGCAG
ATACCAATGACAGGATCAGTGAAGTACAACAAAGTGGCCTGTAAGCCCAGAACTCCACAGGACACTCCCATCTATCCAGTGATTCCAGT
TCACTTGGATGGGACATTTTAG

>Mm-Earps9 AY665816
ATGGGTCCGAAGCTGCTTGAGTCCCGACTTTGTCTCCTGCTGCTGCTAGGACTTGTCCTAATGCTTGCCTCATGCCTGGGTCAAACCCC
TTCCCAGAAGTTTGCCATCCAGCATATCAATAATAATGCCAACCTCCAATGTAATTGAAATGCTGCGTATTAACAGGGCTAGAGTAACA
TGTAAGGGCTTAAATACTTTTCTTCATACAAGTTTTGCTAATGCTGTTGGTGTGTGTGGAAATCCAAGTGGCTTGTGCAGTGACAAGAT
AAGACAAAACTGTCATAATAGTTCATCTCCGGTACATATAACTGTCTGTAACATCACCAGTCGGGCAACAAATTATACCCAATGCAGAT
ACCAATCAAGAAGATCATTGGAGTACTACACAGTTGCCTGTGACCCCAGAACTCCACAGGACAGTCCCATGTATCCAGTGGTTCCAGTT
CACTTGGATGGGACATTTTAG

>Mm-Earps10 AY665817
ATGGGTTCAAAGACGCTTAAGTCCCAACTTTGTCTCCTGCTGCTTCTGGGACTTCTTCTAATGCTTGTTTCATGCCAGGCACAAACCCC
TTCTCAGTGGTTTGAAATCCAGCACATATATAATAGCGCCTATCCCAGATGTGATGATGCAATGAGGGTCATTCACGGTTATAGCGGAG
TATGTAAGGACAAAAATACTTTTCTTCATACAACTTTTGCTAATGTTGTCCCTGTGTGTTGCAATTAGTGTAAGATCTGCAAAGACAGG
AGAAGTACAAATGTCATGATAGTTCATCTAAGATACCTGTAATAATCTGTGATCTCATAACTTGGTCAAACCAACATACACATTGCAGA
TACAAAACAACAGTAGCAATGAAGTCCTACACAGTTGCCTGCAACCCCAGAACTCCACGGAACAGTCCCAGGTATCCATTCGTTCCATG
TCACTTGGATGGGACAATTTAG

>Mm-Earps11 AY665818
CTGACTCTGAAGCTGCCTGAGTCCAGTCTTTGTCTCCTGCTGCTGCTAGGACTTGTGCTAATGCTTGCCTCATGCCAGCAACCAACCCT
TCCCCAGAAGGTTTAGACCGAGTCTATCTATAATAGCACCTACCGCCTATGTGATGTTGCAATGCTGGTTGTTAACAGGTATAGACCAA
GATGTAAGGACATAGATATTTTTCTTCACACCAGTTTTGCTGATGTTGTTGGTGTGTGTGGCCATCCAAATATCACCTGCAACAACGTG
ACAAAAAATAACTGTCATACAAGTTTATTTCTGGTATCTGTAACTTTTTGTAACCTCACTACTCTGACAAGAATTTACACCCAATGCAG
ATACCAATGACAGGATCAGTGAAGTACAACAAAGTGGCCTGTAAGTCCAGAACTCCACAGGACACTCCTATCTATCCAGTGGTTCCAGT
TCACTTGGATGGGACATTTTAG

>Mm-Earps14
CTGACTCTGAAGCTGCCTGAGTCCAGTCTTTGTCTCCTGCTGCTGCTAGGACTTGTGCTAATGCTTGCCTCATGCTAGCAACCAACCCT
TCCCCAGAAGGTTTAGACCGAGCCTATCTATAATAGCACCTACCGCCTATGTGATGTTGCAATGCTGGTTGTTAACAGGTATAGACCAA
GATGTAAGGACATAGATACTTTTCTTCACACCAGTTTTGCTGATGTTGTTGGTGTGTGTGGCCATCCAAATATCACCTGCAACAACGTG
ATGAAAAATAACTGTCATACAAGTTCATTTCTGGTATCTGTAACTTTTTGTAACCTCACTACTCTGAAAAGAATTTACACCCAATGCAG
ATACCAATGACAGGATCAGTGAAGTACAACAAAGTGGCCTGTAAGCCCAGAACTCCACAGGACACTCCCATCTATCCAGTGATTCCAGT
TCACTTGGATGGGACATTTTAG

>Mm-RNase4 NM_021472
ATGATGGATCTACAGAGGACTCAGTCCTTGCTTCTGCTCTTGGTGCTGACCCTGCTGGGGTTAGGGCTTGTACAGCCCTCCTATGGCCA
GGATCGAATGTACCAACGGTTCCTTCGACAGCATGTGGACCCTCAGGTGACAGGTGGCAATGACAACTACTGCAACGTGATGATGCAGA
GACGGAAGATGACTTCTGTCCAGTGCAAACGCTTCAACACCTTCATCCACGAAGACATCTGGAACATTCGTGGCATCTGCAGTACCACC
AATATCCTGTGCAAGAACGGCCAGATGAACTGTCACGAAGGTGTAGTGAAGGTCACGGACTGCAGAGAGACAGGGAACTCCAAGGCCCC
CAACTGTAGATACAGGGCAAGAACCAGCACTAGGCGAGTTGTCATTGCCTGTGAGGGTGACCCAGAGGTCCCAGTGCACTTTGACAGAT
AG

>Mm-Ang1 NM_007447
ATGGCGATAAGCCCAGGCCCGTTGTTCTTGATCTTCGTGCTGGGTCTGGTTGTGATCCCTCCCACTCTGGCTCAGGATGACTCCAGGTA
CACAAAATTCCTGACTCAGCACCATGACGCCAAGCCAAAGGGCCGGGACGACAGATACTGTGAACGTATGATGAAGAGAAGAAGCCTAA
CCTCACCCTGCAAAGATGTCAACACCTTTATCCATGGCAACAAGAGCAACATCAAGGCCATCTGTGGAGCGAATGGAAGCCCTTACAGA
GAAAACTTAAGAATGAGCAAGTCTCCCTTCCAGGTCACCACTTGCAAGCACACAGGAGGGTCTCCCCGACCTCCATGCCAGTACCGAGC
CTCTGCAGGGTTCAGACATGTTGTTATTGCCTGTGAGAATGGTTTGCCTGTCCACTTCGATGAGTCGTTTTTCAGTCTATAG

>Mm-Ang2 NM_007449
ATGGCGATGAGCCCAGGTCCTTTGTTCTTGGTCTTCCTGTTGGGTCTGGTTGTGATCCCTCCCACTCTGTCTCAGGATGACTCCAGGTA
CACAAAATTCCTGACTCAGCACTATGATGCCAAGCCAAAAGGCCGGGACGACAGATACTGCGAAAGTATGATGGTGAAAAGAAAGCTAA
CCTCTTTCTGCAAAGATGTCAACACCTTTATCCATGACACCAAGAACAACATCAAGGCCATCTGTGGAAAGAAAGGAAGCCCTTATGGA

CGAAATTTAAGAATAAGCAAGTCTCACTTCCAGGTCACCACTTGCACACACAAAGGAAGGTCTCCCCGGCCTCCATGCAGGTACCGAGC
CTCTAAAGGGTTCAGATATATTATTATTGGCTGTGAGAATGGCTGGCCTGTCCACTTTGATGAGTCTTTTATCAGTCCATAG

>Mm-Ang4 NM_177544
ATGACAATGAGCCCATGTCCTTTGTTGTTGGTCTTCGTGCTGGGTCTGGTTGTGATTCCTCCAACTCTGGCTCAGAATGAAAGGTACGA
AAAATTCCTACGTCAGCACTATGATGCCAAGCCAAATGGCCGGGACGACAGATACTGTGAAAGTATGATGAAGGAAAGAAAGCTAACCT
CGCCTTGCAAAGATGTCAACACCTTTATCCATGGCACCAAGAAAAACATCAGGGCCATCTGTGGAAAGAAAGGAAGCCCTTATGGAGAA
AACTTCAGAATAAGCAATTCTCCCTTCCAGATCACCACTTGTACGCACTCAGGAGCGTCTCCCAGGCCTCCATGCGGGTACCGAGCCTT
TAAAGATTTCAGATATATTGTTATTGCCTGTGAAGATGGCTGGCCTGTCCACTTCGATGAGTCTTTTATCAGTCCGTAG

>Mm-Ang5 AY665820
ATGGTGATCAGCCCAGGTTCTTTGTTGTTGGTCTTTTTGCTGAGTCTGGATGTGATCCCTCCCACTCTGGCTCAGGATAACTACAGGTA
CAAAAATTTCCTGAATCAACACTATGATGCCAAGCCAACTGGCCGGGATTACAGATACTGTGAAAGTATGATGAAGAAAAGAAAGCTAA
CCTCACCTTGCAAAGAAGTCAACACCTTTATCCATGACACCAAGAACAACATCAAGGCCATCTGTGGAGAGAATGGAAGGCCTTATGGA
GTAAACTTAAGAATAAGCAATTCTCGCTTCCAGATCACAACTTGCAAGCACAAAGGAGGGTCTCCCAAGCCTCCATGCCAGTACAAAGC
CTTTAAAGATTTCAGATATATTGTTATTGCCTGTGAAGATGGCTGGCCTGTCCACTTCGATGAGTCTTTTATCAGTATGTAG

>Mm-Ang6 AY665821
ATGGTGATGAGCCCAGGTTCCTTGATGTTGGTCTTTGTGCTGAGTCTGGTTGTGATCTCTCCCACTCTGGCTAAGGATAACTACAGGTA
CATAAAATTCCTGACTCAGCACTATGATGCCAAGCCAACAGGCTGGGATCACAGATACTGTTATCTGTGTATGATGAAGAAAAGAATCC
AAGAAACCTTGAAATGCAAAGAAGCCAACACCTTTATCCATGACACCAAGAAAAACATCAAGGCCATCTGTGGAGAGAATGGAGGCCCT
TATGGAGCAAACTTTAGAATAAGCAATTCTCCCTTCCAGATCACCACTTGCAATCACTCAGGAGGGTCTCCCAAGCCTCCATGCCAGTA
CAGAGACTTTAAAGATTTCAGATATATTGTTATTGCCTGTGAAGATGCCTGGCCTGTCCACTTCGATGAGTCTTTTATCAGTCTGTAG

>Mm-Angps1 NG_001342
ATGGTGATGAGCCCAGGTCCTTTGTTGTTAGTCTTTGTGCTTAGACTGGTTGTGATCCCTCCCACTCTGGCTCAGGATGCCTACAGGTA
CATAAAATTCCTGACTCAGCACTATGATGCCAAGCCAAAAGGGCCAGGACGACAGATACTGTGAACTTATGATGAAGGAAAGAAAGCTA
ACCTTGCCCTGCAAAGAAGTCAACACCTTTATCCATGACACCAAGAACAACATCAATGCCATCTGTGTAAAGAATGGAAGCCCTTACAG
AGAAGGCTTAAGAATAAGCAATTCTCGCTTCCAGGTCACCACTTGCAAGCACACAGGAGGGTCTCCCTGACCTCCACGCCTGTACCGAG
CCTCTGCAGGGTTCTGAGATATTGTTATTGCCTGTAAGAATGGCTTCCCTAATCATTTCTATGAGTCTTTTATCAGTCTGTAG

>Mm-Angps2 NG_001343
ATGGTGATGAGCCCAGGTCCTTTGATGTTAGTCTTTGTTCTTAGACTGGTTGTGATCCCTCCCACTCTGGCTCAGGATGCCTACAGGTA
TATAAAATTTCTGACTCAGCACTATGATGCCAAGCCAAAGGGCCAGGACAACAGATACTGTGAACTTATGATGAAGGAAAGAAAGCTAA
CCTCGCCCTGCAAAGAAGTCAACACCTTTATCCATGACACCAAGAACAACATCAATGCCATCTGTGTAAAGAATGGAAGCCCTTACAGA
GAAGGCTTAAGAATAAGCAATTCTCGCTTCCAGGTCACCACTTGCAAGCACACAGGAGGGTCTCCCTGACCTCCACGCCTGTACCGAGC
CTCTGCAGGGTTCAGACATGTTGTTATTGCCTGTGAGAATGGCTTGCCGGTCCACTTCGATGAGTCATTTTTCAGTCTATAG

>Mm-Angps3 AY665822
ATGGTGATGAGCCCAGGTCCTTTGTTGTTAGTCTTTGTGCTTAGACTGGTTGTGATCCCTCCCACTCTGGCTCAGGATGCCTATAGGTA
TATAAAATTTCTGACTCAGCACTATGATGCCAAGCCAAAGGGCCAGGACAACAGATACTGTGAACTTATGATGAAGGAAAGAAAGCTAA
CCTCGCCCTGCAAAGAAGTCAACACCTTTATCCATGACACCAAGAACAACATCAATGCCATCTGTGTAAAGAATGGAAGCCCTTACAGA
GAAGGCTTAAGAATAAGCAATTCTCGCTTCCAGGTCACCACTTGCAAGCACACAGGAGGGTCTCCCTGACCTCCACGCCTGTACCAAGC
CTCTGCAGGGTTCTGATATATTGTTATTGCCTGTAAGAATGGCTTCCCTAAACATTTCTATGAGTTTTTTATCAGTCTGTAG

>Mm-RNase6 NM_030098
ATGGTGGTAGACCTTCCCAGGTACCTTCCTCTCCTATTGCTGCTTGAGCTGTGGGAGCCGATGTATCTACTTTGCTCTCAGCCTAAGGG
TCTCTCCAGGGCACACTGGTTTGAAATTCAGCACGTACAGACAAGTCGTCAACCATGCAACACAGCCATGCGCGGTGTCAACAATTATA
CCCAACACTGTAAACAGATAAACACCTTTCTGCATGAATCTTTCCAGAATGTGGCTGCTACCTGCAGTTTGCACAATATCACCTGCAAG
AACGGTCGGAAGAACTGCCACGAGAGCGCAGAACCTGTCAAGATGACTGACTGCTCACACACTGGAGGAGCCTATCCTAACTGCCGCTA
TAGTAGTGATAAGCAATACAAGTTCTTCATTGTGGCCTGTGAACACCCAAAGAAGGAAGACCCTCCCTACCAACTAGTGCCTGTGCACT
TAGATAAGATTGTGTGA

>Mm-RNase9 NM_183032
ATGAAACCTTTGGTCATCAAGTTCGCCTGGCCTTTGCCATTGCTGCTGCTGCTGCTGCTGCCACCGAAGCTTCAGGGGAACTATTGGGA
TTTTGGGGAATATGAACTAAACCCAGAAGTACGTGACTTTATTAGGGAATATGAGAGTACAGGGCCCACCAAACCACCTACCGTAAAAA
GAATCATAGAAATGATCACCATTGGTGACCAACCTTTCAATGACTACGATTACTGCAACACTGAACTCAGGACTAAACAGATTCACTAT
AAGGGGCGCTGTTACCCAGAACACTACATTGCAGGAGTGCCCTATGGTGAGCTAGTAAAAGCCTGTGATGGTGAAGAAGTGCAATGTAA
GAATGGCGTTAAATCTTGCAGAAGAAGCATGAATCTAATCGAGGGAGTGAGGTGTGTTTTAGAAACAGGACAGCAAATGACAAACTGCA
CATATAAAACCATCCTCATGATTGGTTATCCAGTTGTCTCCTGTCAATGGGATGAAGAAACCAAAATTTTTATTCCTGACCATATATAT
AATATGTCACTACCCAAGTAG

>Mm-RNase10 AY226990
ATGAAGGTGACACTGGTGCATCTGTTGTTCATGATGTTGCTGCTGTTGCTAGGCCTAGGGCTGGGGCTGGGCCTGGGCCTTCACATGGC
AGCTGCCGTCCTGGAGGATCATCCACTGAATGAATTTTGGCCCAGTGACTCCCAGAATACTGAAGAGGGAGAGGGCATCTGGACCACAG
AAGGTCTGACACTTGGCTACAAAGAAATGGCACAACCTGTCTGGCCAGAAGAGGCTGTCCTCAGTGAAGATGAAGTGGGGGGAAGCAGG
ATGCTGAGGGCTGAGCCCCGCTTTCAGAGCAAACAAGACTACCTTAAGTTTGACTTGAGTGTCAGGGACTGTAATACCATGATGGCACA
CAAGATAAAGGAGCCTAATCAGAGCTCCATAAACCAGTACACGTTCATCCATGAGGACCCAAACACAGTCAAAGCTGTCTGTAACGGTT
CCCTGGTTGACTGTGACCTCAAGGGGGGCAAATGTTACAAAAGTCCCCGGCCTTTTGATCTGACATTGTGCAAATTGGCCAAACCAGGC
CAAGTCACTCCCAACTGTCACTATCTGACTTACATAACCGAAAAGGTCATTTTCATGACATGCAATGACAAGAAACAACTGGAGACTAA
ATGA

>Mm-RNase11 AY665823
ATGGCAGTCTTTCTGCTGTTGCTCGCCCTTGGACTGCTTCTTGCAAAGCCTTCAGAAAGCAGAATGAAGGGGACCACAGAACAATTTTC
ACAGGAAGAGATGCAACCTGCTGCAAAACAGACTCTAGAGGAATCAGCAAACTCAACCCTGTCAGACAAAAATACTGGCCTCAGCATAT
CCAAACATGTGATGTCTGCCACACCCCTAACACCCAGAAGGCTATCTTTTATTATCCCCAAGGGAAATACTATGAGAGATGGCAGGAAC
TGTGTAAACAGCCTAAGAGTCTGGAGAACCGAGGTAGACGGGAATGCATCCTGTCAGTTGGGCAATGACTTTATACATGGCTCCATGGA
TGTGAGCCTCAGAATTCCCAAGGCCACCCGAGGGAAGTGTGAACAAACACCCAAGCCAAGCAGCAGTGGGAGCCTAGGACTGGAGCGTA
CCACGTGCAAGGTCCTTGCAGGCCACCAGTGCCTCAGGTCCCATGAGCACAGCATCACCTCATTAAAGAAAATCCTGACAGTGCTGGCC
AGCAATTCTCTGATGAGCTGGTTAGTTAGTGGCTGTAAATTGTAA

>Mm-RNase12 AY665824
ATGGTCCTAATGGTGGTTGTTTTCTTGCTGCTTCTGTTCTGGGAGAATGAGCTCACTGAAGATGTTGTGCTGACGTCCATAGAACAATT
GCATGTGGACTATCCTCAGAATGCTGTTCCTCTGAGGTACTGCAACTACATGATTCTCCAGAGGGTCATCAGGGAACCCGACCACAGAT
GCAGAAAGGTGCATGTCTTCATCCACGAGAGGCCTCAGAAGATCAACAGAGTTTGCACTTCCTCCAAGAAGATGTCGTGCCCAAATGAT
TCTGACCTTTTCTGCTTCCAGAGTGAGACAAAATTCAGGATGACAGTTTGTCAGCTCATTGATGGCACCACATATCCTGCCTGCAGGTA
CCAAATTTCTCCCATAAAGGGGTTTGTTCTTGTGACTTGTGATGACTTAGGGCCAGTTGATCTCCAGGGATATGTGGAATAA

>Mm-RNase13 AY665825
ATGGCACCAGATGTGGCCTGGCTCCTTGTCCTCCCGCTTGTCTTCCGGCCAACTCTGGTGACAGGCATCACAATTCAGACGGCCATCAA
GAACTTCCGAACCTTACATGTTGACTACCCCATGGTTAACTACCCAAAGGGATTCCACGGATACTGTAATGGCCTCATGGCCTACGTGA
GAGGCAAACTGCAGGACTGGTACTGCCCTAAGATCCACTATGTGGTCCACGCCCCCTTGGAAGACATCCAGAAGTTCTGTAAATACAGC
GAGAGCTTCTGTGAAAACTACAACGAGTACTGCACCCTCACTCAGAACTCCTTCCCCGTCACAATCTGCACCCTGGTTCATCAACAGGC
ACCAACCAGCTGTAGCTATAACAGCACCCTGACCAACCAAAGGCTGTACTTGCTCTGCTCCCGCAAGCATGATGCTGAGCCTATAGGTA
TCATTGGTCTCTACTAG

Rat (*Rattus norvegicus*)

>Rn-RNase1 (RNase1delta)
ATGGATATGGAGAAGTACCTCTTTCCGTTTTCACTGCTTATATTGGTGCTTGGATGGGTCCATCTTTACCTGGGTGGGGAATCTAGGGA
ATCATCGGCGGATAAGTTTAAGAGGCAGCACATGGACACAGAGGGTCCCTCCAAGAGCAGCCCCACCTACTGTAACCAGATGATGAAGC
GCCAGGGGATGACCAAGGGGTCATGCAAGCCAGTGAACACCTTCGTGCATGAACCCTTGGAGGATGTCCAGGCCATCTGCTCCCAGGGA
CAAGTGACCTGCAAGAATGGGAGGAACAACTGCCACAAGAGCAGCTCCACCCTGCGCATCACTGACTGCCGCCTGAAGGGCAGCTCCAA
GTATCCCAATTGCGACTACACAACCACTGACAGCCAGAAGCACATCATCATTGCTTGTGACGGGAACCCCTACGTCCCAGTCCACTTCG
ATGCTTCCGTGTAG

>Rn-RNase1-like1 (RNase1gamma)
ATGGGTCTGGAGAAGTCCCTCATTCTGTTTTCACTGCTTGTCCTGGTGCTTGGATGGGTCCAGCCTTCCCTGGGTAGGAAACCTTCAGT
CCAGGATTTTAAGAGGCAGCACATGGATCCGGATAGTCCTCCCAACAGCAGACCCACCTACTGTAACCAGATGATGAAACGCCGGGGGA
TGACCAAGGGGTCATGCAAGCGGGTAAACACCTTCCTGCATGAATCCTGGGCAACGGTCAAGGCCATCTGCTCCCAGAGACAAATGACC
TGTAAGACCTCCAGCAGGAACAACTGCCACAAGAGCAGCTCCACCCTGCACATCACTGACTGCCGCCTGAAGGGCAGCTCCAAGTATCC
CAATTGTGACTACACAACCACTAACAGCCAGAAGCACATCATCATTGCTTGTGAGGGGAACCCCCTCGTCCCAGTCCACTTCGATGCTT
CCGTGTAG

>Rn-RNase1-like2 (RNase1beta)
ATGGGTCTGGAGAAGTCCCTCTTTCTGTTTTCACTGCTTGTCCTGGTGCTTGGATGGGTCCAGCCTTCCCTGGGTGTAGAATCTAGGGA
AACTCCAGCCCAGAAGTTTGAGAGGCAGCACATGGACGAGGAGGGTCCCTTCCCGAGCAGCCCCACCTACTGTAACGAGATGATGAAGA
GCAGGGGGATGACCTCGGGGTGGTGCAAGTCCATGAACACCTTCGTGCATGAACCCTTGGCAACGGTCCAGGCCATCTGCTCCCAGGGA
CAAGTGACCTGCAAGAATGGGAGGAACAACTGCCACAAGAGCAGCTCCACCCTGCGCATCACTGACTGCCGCCTGAAGGGCAGCTCCAA
GTATCCCAATTGTGACTACACAACCACTAACAGTCAGAAGCACATCATCATTGCTTGTGAGGGGAACCCCCTCGTCCCAGTCCACTTTG
ATGATTCCGTGTAG

>Rn-Ear3-like
ATGGGTCTGAAGCTGCTCGAGTCCAGACTTTGTCTCCTTCTGCTTCTGGGACTTGTCACATGCCAGCGACCAACCCCTTCCCAGTGGTT
TGCCATCCAGCACATCTATAATAGCTCCTACCCCCAATGTAATGCTGCCATGCTGCGTGTCAACAGTTATACAGGAAGATGTAAGGGCA
TAAATACTTTCCTTCATGCAAGTTTTGCTAATGTTGTTGGTGTGTGTGGCAATCCACATACAACCTGTAAAGACAGAATCAGTACGAAT
TGTCATAACAGTTCATCTCAGGTATCTATAACTTTCTGTAACCTCACAACTCCGGCAAGGATTTATACCCAATGCAGATACCAAACTAC
AGGATCGTTGAAGTTCTACACAGTTGCCTGTAACCCCAGAACTCCACGGGACAGTCCCATGTATCCAGTGGTTCCGGTTCACTTGGATA
GGATATTTTAG

>Rn-R17
ATGGGTCTGAAGCTGCTCGAGTCCAGACTTTGTCTCCTTCTGCTGCTGGGACTTGTCCTGATGCTTGCCTCATGCCAGCCACCAACCCC
TTCCCAGTGGTTTGAAATCCAGCACATCTATAATAGGGCTTATCCACGATGTAATGATGCAATGCGACACAGAAACAGATTCACCAGAC
ATTGTAAGGACAAAAATACTTTCCTTCATACAAGTTTTGCTAGTGTTGTTGGTGTGTGTGGCAATAGAAACATCCCCTGTAGAAACAGG
AGATATAGAAATTGTCATAACAGTCCATATCGGGTATCTATAACTTTCTGTAACCTCACAACTCCGGCAAGGATTTATACCCAATGTAG
ATACCAAACTACAAGATCCAGGAAGTTCTACACAGTTGGCTGTGACCCGAGGACTCCACGGGACAGTCCCATGTATCCAGTGGTTCCGG
TTCACTTGGATAGGATATTTTAG

>Rn-RNase2
ATGGGTGTGAAGCCGCTTGAGTCCAGACTTTGTCTCCTTCTGCTGCTGGGACTTGTCTCAATGCTTGCCTCATGCCAGCGACCAACCCC
TTCCCAGTGGTTTGCCATCCAGCACATCTATAATAGCTCCTACCCCCAATGTAATGCTGCCATGCTGCGTGTCAACAGTTATACAGGAA
GATGTAAGGGCATAAATACTTTTCTTCACACAAGTTTTGCTAGTGTTGTTGATGTGTGTGGCAATCCACATATAACCTGTAAAGACGGG

AGAAGTACAAATTGTCATAACAGTTCCTCTCAGGTATCTGTAACTTTCTGTAACCTCACAACTCCGGCAAGGATTTATACCCAATGCAG
ATACCAAACTACAGGATCGGTGAAGTTCTACAGAGTTGCCTGTAACAATAGAACTTCACAGGAGAGTCCCATGTATCCAGTGGTTCCTG
TTCACCTGGATGAGATATTTTAG

>Rn-ECP-like
ATGGGTCTGAAGCTGCTCGAGTCCAGACTTTGTCTCCTTCTGTCGCTGGGACTTGTCTTGATGCTTGCCTCATGCCAGCCACCAACCCC
TTCCCAGTGGTTTGAAATCCAGCACATCTATAATAGGGCTTATCCCCGATGTAATGATGCAATGCGACACAGAAACAGATTCACAGGAC
ATTGTAAGGACATAAATACTTTTCTTCATACAAGTTTTGCTAGTGTTGTTGGTGTGTGTGGCAATAGAAACATCCCCTGTGGAAACAGG
ACATATAGAAATTGTCATAACAGTCGATATCGGGTATCTATAACTTTCTGTAACCTCACAACTCCGGCAAGGATTTATACCCAATGTAG
ATACCAAACTACAAGATCCAGGAAGTTCTACACAGTTGGCTGTGACCCGAGGACTCCACGGGACAGTCCCATGTATCCAGTGGTTCCGG
TTCACTTGGATAGGATATTTTAG

>Rn-RNase16-like
ATGGGTCTGAAGCTGCTGGAGTCCAGACTTTGTCTCCTTCTGCCGCTGGGACTTGTCCTGATGCTTGCCTCATGCCAGCGACCAACCCC
TTCCCAGAGGTTTGCCATCCAGCACATCTATAATAGCGCCTACCCCCAATGTAATGCTGCTATGCAGCGTGTTAACAATTATACAGGAA
GATGTAAGGACATAAATACCTTTCTTCATACAAGTTTTGCTAGTGTTGTTGGTGTGTGTGGCAATAGAAACACCACCTGTAATAGTAAC
AGGACAAGAACAAATTGTCATGACAGTACATATCAGGTAAAAGTAACTATCTGCAACCTCACAAATCGGGCAGCCGTTTATCCTCAATG
CCCATACCAAACTATAAATTCATCGAAGTTCTACAGAGTTGCCTGTGACCCCAGAACTCCACGGGACAATCGCACATATCCAGTGGTTC
CAGTTCACTTGGATTTCATATTTTAG

>Rn-RNaseps
ATGGGTCTGAAGCTGCTCAAGTCCAGACTTTGTCTCCTTCTGCTGGGACTTATCCTAATGCTTGCCTCATGCCAGCCACCAACCCCTTC
CCAGCGGTTTGAAATTCAGCACATCTATAATAGCGCCTACCCCCAATGAAATGCTGCCATGCTGCGTGTCAACAATTATACAAGAAATT
GTACAGACAAAAATACTTTCCTTCATACAAGTTTTGCTGATGTTGTTGGTGTGTGTGGCAATAGAAAGACCCCCTGTAGAAACGGAAGA
ATAAATTGCCATAACAGTTCATATCAGGTAAAAATAACTATCTGCAACCTCACAAATCCGGCAGCCATTTATCCCCAATGCCCATACCA
AACTATCAATTCATCGAAGTTCTACACAGTTGCCTGGGACCCTAGGACTTCACGGGACAGTCCCATGTATCCAGTGGTTCCGGTTCACT
TGGATAAGATATTTTAG

>Rn-RNase4
ATGGACATACAGAGGACCCAATCCTTGCTTCTGCTCTTGTTGCTGACCCTGCTGGGGTTAGGGCTTGTACAGCCCTCCTATGGCCAAGA
TAGAATGTACCAACGGTTCCTTAGACAGCATGTGGACCCTGAGGGGACAGGCGGCAGCGACAACTACTGCAACGTGATGATGCAGAGAC
GGAGGATGACTTCTACCCAGTGCAAACGCTTCAACACCTTCATCCACGAAGACATCTGGAACATTCGCAGCATCTGTGATACTGCCAAT
ATCCCATGCAAGAATGGCAATATGAACTGTCACGAAGGCATAGTGAGGGTCACTGACTGCAGAGAGACAGGGAGCTCTGTGCCCCACAA
CTGTAGGTACAGGGCGAGAGCCAGCACTAGGCGAGTTGTCATTGCCTGTGAGGGTACCCCAGAGGTCCCAGTGCACTTTGACAGATAG

>Rn-Ang1
ATGGAGATGAGCCTGCGTCCTCTGTTGTTGGTTTTTGTGCTGGGTCTGGTTTCGACCCCTTCAACTCTGGCTCAGGACGACCCCAGGTA
CACGAAGTTCCTGACTCAGCACTATGATGCCAAGCCCAAGGGTCGGGATGCCAGATACTGCGAAAGTATGATGAGGAGAAGAGGCCTAA
CCTCGCCCTGCAAAGAGGTCAACACCTTTATCCATGGCAACAAGGGCAGCATCAAGGCCATCTGTGGCGCGAATGGAAGCCCTTACGGA
GAAAACTTAAGAATAAGCCAGTCTCCCTTCCAGATCACCACCTGCAAGCATACAGGAGGGTCTCCCCGGCCCCCTTGCCGGTACCGAGC
CTCTGCAGGGTTCAGACATGTTGTTATTGCCTGTGAAAATGGCTTGCCTGTCCACTTTGATGAGTCTTTTATCAGTCTCTAG

>Rn-Ang2
ATGAGCCTGCGCCCTCTGTTGTTGGTTTTTGTGCTGAGTCTGGTTGCGACCCCTTCAACTCTGGCTCAGGACGACCCCAGGTACACGAA
GTTCCTGACTCAGCACTATGATGCCAAGCCCAAGGGTCGGGATGCCAGATACTGCGAAAGCATGATGAAGAGAAGAGGCCTAACCTCGC
CCTGCAAAGAGGTCAACACCTTTATCCATGGCAATAAGGGCAGCATCAAGGCCGTCTGTGGTGCGAATGGAAGCCCTTACGGAGAAAAC
TTAAGAATAAGCCAGTCTCCCTTCCAGATCACCACTTGCAAGCATACAGGAGGGTCTCCCCGGCCCCCTTGCCGGTACCGAGCCTCTGC
AGGGTTCAGACATGTTGTTATTGCCTGTGAGAATGGCTTGCCTGTCCACTTTGATGAGTCTTTTATCAGTCTCTACTCAGCAGGCCCCC
GGCGCAGACCTAGCTCTGTTCTCTTTTTATCTCCCCTCACCCCAGAACACTGA

>Rn-RNase6
ATGGCGGTAGACCTTCCCAGGTGGCTTCCTCTCCTATTACTGCTGGGGCTGTGGGAGCCCATGGGTCTGCTTTGTGCTCAGCCTAAGGG
TCTCTCCAAGGCGCGCTGGTTTGAAATTCAGCACATATGGGCTAGTCCTCAGCAGTGCAATGCAGCCATGCGTGGTGTCAACAACTATA
CCCGATACTGTAAGCAGAAGAACACCTTTCTGCATGAATCTTTCCAGAATGTGGCTGCTACCTGCGGTTTGCCCAACATCACATGCAAG
AACGGTCGGAAGAACTGCCACGAGAGTGTGAAGCCTGTCAAAATGACTGACTGCTCACACACAGGGCAAGCCTATCCTAACTGCCGCTA
CAGTGGTGATGTCCAATACAAACTCTTCGTTGTGGCCTGTGAACACCCAAAGAAGGACGACCCTCCCTACCAACTGGTTCCTGTGCACT
TAGATAAGGTTGTGTAA

>Rn-RNase9
ATGAAGTCTCTGGTCATCAAGTGCACATGGCCTTTGCCATTGCTGTTGTTGCTGCCACTGAAGCTGCAGGGGAACTACTGGGATGAAAA
GGAATATGATCTAGAGCCAGGACTACGTGAATTCTTTAGGGAAATTGCGAGTACAGGGCCTACCAAACCACCTACCAAAGAAAGAATCA
TAGAAATGATCACTGTTGGCGAACGACCTTTGCAGGATTATGATTACTGCAACTCTGAACTCAGGGCCAAGCAGATTCACGATAAGGGG
CGCTGCTACCCAGAGCGCTACATTGTAGGAATGTCTTACGAAGAGCTGTCAAAAGCCTGTTACGGTCAACGAGTGCAGTGTAAGAATGG
CGTGAAATTTTGCAGAAGAAGCATGGATCTAACAGACGGAGTGCGGTGTGTTTTAGAAAGTGGAGAGCGGATGGTAGACTGCATGTACA
CAACCATCTACATGACCGGTTATCCAGTCGTCACGTGTCAATGGGACAAAGAATCCCAAGAGTTTATTCCTAACTATATACATAATATG
TCACTGCCTGAGTAG

>Rn-RNase10
ATGAAGGTGACACTGGTGCATCTGTTGTTCATGATGTTACTGCTGTTGCTAGGACTGGGGGTGGGGCTGGGGCTGGGCCTTCACATGGC
TGCTGCCATCCTGGAGAATCAGCCACTGGATGAGTTTTGGCCCAGTGACTCCCAGGACACAGCTGAGGCCACTGAAGAGGGACAGGGCA
CCAGGACCACAGAAGCCCTGGTACTTGACAACAAAGAAATGGCAGTACCTGTCTGGTCAGAAGATACTGTCCTCAGTGAAGATGAAGTG

GGGGGAAGCAGGATGCTGAGAGCCAAGACCCTCCTTCAGAGCAAGCAAGGCTACCTTAAGTTTGACTTGAACATCCGGGACTGTAACGT
CATGATGGCCCACAAGATAAAGGAGCACAATCAGAGCTGCATAAACGATTATACATTCATCCATGAGGATCCAAGCACAGTCGGAGCCG
TCTGTAACAGTCCCCTGGTTGACTGTGATCTCAAGGGAGGCAAATGTCACAAAAGCCCCCGGCCTTTTGACCTGACATTGTGCAAGTTG
GCCAAACCAGGCCAAGTCACTCCCAACTGCCACTATCTGACTTACATAACTGAAAAGGTCATTATCATAACATGCAACAACACGAAACA
ACTGGAGATTAAATGA

>Rn-RNase11
ATGGCAGCCTTTCTGCTGCTGCTTGTCCTTGGACTGCTTCTTGTAGAGCCTTCCGAAAGCAAAACGAAGGGGGCCAGGGAACAATTTTC
CCAGGAAGAGACTCGATCTGCTGCAAAACAGACTCCTGAGGAATCAACAAACTCAACCCTGTCAGATGAAAACATCAGCCTCGGCATAT
CCAGGCACGTGATGTCTGCCCCACCCCGAACATCCAGAAGGCTGTCTTTTGTCATCCCCAAGAGAAATACTGTGAGGAATGGCAGGGAC
TGTGTAAATAGCCCGAGTGTGTGGAGAACAGATGTGGAGGTGAATGAGTCTTGCCAGTTGGGCAATAACTTTATACATAGCTCCATGGA
TGTGAGCCACGGAATTCCCAAGGCCACCAGCGGGAAGTGCGAACGAACACCTAACCTAAGATACTGTGATAGCTTAGGACTGGAGTGTA
CCATGTGCAAGGTCCTTGCAGGCCACCAGTGCCCCAGGTACCATGAGCACAGGATAACCTCATTAAAGAGAATCTTGACGGTGCTGACA
AGCCATTCTCTGATGAGCTGGTTAGTTACTGGCTGCTAA

>Rn-RNase12
ATGATCCTAATGGTGATCGTTTTCTTGCTGCTTCTCTTCTGGGAAAACGAGCTAACTGAAGATGTTGTGCTGACGTCTATGGAACATTT
GCATGTGGACTACCCTCAGAGTGCTGTTCCTCTGAGGTACTGCAACTACATGATTCTACAGAGGGTCATCAGGGAGCCTGACTACACGT
GCAGAAAGGTGCACGTCTTCATCCATGAGAGGCCTCAGAAGATCAACAGGATTTGCACCTCCTCCAAGAAGATGACTTGCCCAAACTAC
TCCGAAATTTTCTGCTTCCAGAGCGACACAAAATTCAGGATGACAGTTTGTCAGCTCACTGGTGGCTCCAAATACCCTGCCTGCAGGTA
CCAAATTTCTCCCACAGAGGGGTTTGTTCTTGTGACTTGTGACGACTTAGGGCCAGTTAATTTCCAGGGATATGTCGAATGA

>Rn-RNase13
ATGGCATCAGATGCGGCCTCGCTCCTTGTCCTCCAGCTTGTCTTACAGCCAACTCTGGTGACAGGCATCACGATACAGACGGCCATCAA
GAACTTCCGAATCTTACATGTCGACTACCCCATGGTTAACTACCCGAAGGGTTTCCACGGGTACTGTAATGGCCTCATGGCCTACGTGA
GAGGCAAACTCCAGGACTGGTACTGCCCTAAGATCCACTATGTGGTCCATGCCCCCTTTGAAAGCATCCAGAAGTTCTGCAAATACAGC
GAGAGCTTCTGTGAGGACTACAACGAGTACTGCACCCTCACTCAGAACTCCTTCCCCATCACTGTCTGTACCCTGGATCATAAGCAGGC
CCCAACCAGCTGTAGCTACAACAGCACTCTGACCAACCAAAGGCTGTACTTGCTCTGCTCCCGCAAGCATGATGCTGAACCAATAGGTG
TCATCGGCCTCTACTAG

Nake mole rat (*Heterocephalus glaber*)

>Hg-RNase1
ATGGCTCTGGAGAAGTCCCTGGTCCTCTTCCCACTACTTGTCCTGGTGCTCCTGGGGCTGGTGTGTGTCCAGCCTTCCCTGGGCAAAGA
ATCCTCTGCCATGAAGTTCCGGCGGCAGCACATGGACTCAGAGGGTTCCCCCAACACCAACGCCAACTACTGCAATGAAATGATGAGAC
GCAGGAATATGACGGAGGGACGCTGCAAGCCCGTGAACACCTTTGTGCATGAGCCCCTAGCAGATGTCCAGGCTGTCTGCTTCCAGAAA
AATGTCGCCTGCAAAAATGGGCAGACCAACTGCTACCAGAGCACCTCCAGCATGCACATCACAGACTGCCGCCTGACGAGCAACTCCAA
GTACCCCACCTCCTACCGCAGCACCAGCCAGATGGAGAGGAGCATCATCGTGGCCTGTGAGGGGAACCCGTATGTGCCAGTCCACTTCG
ATGCTTCTGTGGAGTCCTCCACCTAA

>Hg-RNase1psA
ATGGCTCAGAAGCAGTCCCTTGTCCTCTTCCCACTGCTCATCCTGGTGCTCCTGGGGCTGGTGTGGGTCCAGCCTTGCTTAGGCAAAGA
ATCTTCAGAGATGAGGTTTCAGCAGCAGCTCATGGACTCAGATCATTTGTTCCTCCAGCAGCAACACCAATTACTGCAATGAAATGATG
AAGTGCAGGAATATGACAGAGAGATGCTGCAAGCTGGTGAACACCTTCATGCATGATCCCCTGGCAGATGTCCAGGCTGTCTGCTTCCA
GAAAAATGTTACCTGCAAGAATGCACAGACCAACTTCTACCAGAGCAGCTCCAACATGCACATCACAGGCTGCCGCCTGACGAGCAACT
CCAAGTACCCCACCTGCTCCTACCGCACCAGGCAAGTGGAGAGGAGCATCACTGTGGCCTGTGAGGGGAACCCCTATGTGCCAGGCCAC
TTTGATGCTTTGTGGAGTCCTCCACCTCA

>Hg-RNase1psB
ATGGCTCAGAAGCAGTCCCTTGTCCTCTTCCCACTGCTCATCCTGGTGCTCCTGGGGCTGGTGTGGGTCCAGCCTTGCTTAGGCAAAGA
ATCTTCAGAGATGAGGTTTCAGCAGCAGCTCATGGACTCAGATCATTTGTTCCTCCAGCAGCAACACCAATTACTGCAATGAAATGATG
AAGTGCAGGAATATGACAGAGAGATGCTGCAAGCTGGTGAACACCTTCATGCATGATCCCCTGGCAGATGTCCAGGCTGTCTGCTTCCA
GAAAAATGTTACCTGCAAGAATGCACAGACCAACTTCTACCAGAGCAGCTCCAACATGCACATCACAGGCTGCCGCCTGACGAGCAACT
CCAAGTACCCCACCTGCTCCTACCGCACCAGGCAAGTGGAGAGGAGCATCACTGTGGCCTGTGAGGGGAACCCCTATGTGCCAGGCCAC
TTTGATGCTTTGTGGAGTCCTCCACCTCA

>Hg-RNase1psC
ATGGCTCAGGAGCAGTCCCTTGTCCTGTTCCCACTGCTCATCCTGGTGCTCCTGAGGCTGGGGTGGGTCCAGCCTCTTAGGATATAGAA
TCCTCAGCCATGATGTCTCAGCAGCAGCACGTGGACTCAGGCCATTCTCCCAGCAACAACCCCAACTATTGCAATGAAATGATGATGCG
CAGGAATATGACACAGAAACACTGAAAGTCAGTGCATATCTTTGTGCTCTTGTCCAGGCTGTCTGCTTCAAGAAAAATATCACCTGTAA
GAATGGGCAGACCAACTGCTACCAGAGCAGCTCCAGCATGCACATCACAGGCTGCCGCCTGACAAGCAACTCCAAGTACCCCACCTGCT
CCTACTGCACCAGCCAGGTGGAGAGGAGCATCACTGTGGCCTGTGAGGGGAACCCGTATGTGCCAGGCCACTTCGATGCTTCTGTGGAG
TCCTCCACCTCA

>Hg-RNase1psD
ATGGCTCAGGAGCAGTCCTTTATCCTCTTCCCACTGCTCATCCTGGTGCTCCTGGGGCTGAGGTAGGTCCAGCCTTTCTTGGGAAAAGA
TCTTCAGAGGTGAGATCTCAGCAGCAGCTCATGGACTCAGACAGTTCCCCCAGCAGCAACACCAACTATGGCAATGAAATGATGAAGCA
CCGGTATTTGACCCAGAGATGCTGCAAGCTGGTGAACACCTTCATGCATGATCCCCTGGCTAATGTCCAGGCTGTCTGCTTCCAGAAAA
ATTTCACCTGAATGAATGGGCAAACCAACTGCTACCAGAGCACCTCCAGCATGCACATCACAGACTGCCACCTGACGAGCAACTCCAAG
TACCCCACCTGCTCCTACCACACAAGCCAGGTGGAGAGGAGCATCACTGTAGCCTGTGAGGGGAATCTGTATGTGCCAGTTCACTTAGA
CACTTCTGTGGAGTCCTCCACCTAA

>Hg-RNase4
ATGGCTCTTCAAAGGACCCATTCACTGCTTCTGCTCTTGCTGCTGACCCTTCTGGGGCTGGGGCTGGTGCAGCCCTCCTATGGCCAGGA
TCGAATGTACCAGCGATTCCTGCGGCAGCACGTGGACCCCCAGGAGACAGGCGGTGATGACAGTTACTGCCACGTGATGATGCAAAGAC
GGAAGATGACTAGTCCAAAGTGCAAGCCCTTTAACAGTTTCATCCATGAAGACATCTGGAACATTCGCAGTATCTGCAGCACCACCAAT
ATTCAGTGCAAGAACAACCAGATGAACTGTCATGAAGGTGTCATGAAGGTCACAGATTGCAGGCAGACAGGAAATTCCAAGGCACCTAA
CTGCAGATATCGGGCTAGGGCGAGGACCAGACGAGTTGTCATTGCCTGTGAGGGTTACCCTCCAGTGCCTGTGCACTTTGATGCATAG

>Hg-RNase6A
ATGGTTCGAGATGTGAGATTTCCTCTCCTACTGCTGCTGGGGCTACTTGTGCTACCGTGTCCATTGTGGGCTTTGCCTAAGAAACTCAC
GAAGGCTCAATGGTTTGAAATCCAGCACGTACAGCCGAGTCCTCTCCGATGTGACAAGGCAATGAGTGGTGTCAACAATTATACCCGGC
ACTGTAAGCCTACAAATACCTTTCTGCATGACTCTTTCCAAAATGTGAGTGATTCCTGCACCTCGCCCAATGTCACCTGCAAGAATGGT
CAGAAAAACTGCCACCAGAGTGCAAGTCCTGTCAACCTGACTATCTGCAGGCTCACCAGAGGGAAGTATCCTCACTGTCCGTATAAAGA
TGTTCCCCAGATCAAATTCTTCATTGTGGCCTGCGAACCACCTCGGAAGAATGACCCTCCCTATCCACTGGTTCCTGTACACTTAGATG
GGATTGTCTAA

>Hg-RNase6B
ATGGTGGGAGATGTGAGATTTCCACTCCTACTGCTGCTGGGGCTACTGTGTCCTCTTTGGGCTGTGTCTCAGCCCTTCACACGGTTTCA
GTGGTTTGCCATTCAGCATGTAAGCCCAAATCCAATCGCCTCATGCAATGTGGCCATGAGACCTATCAACCAACATCTCCCTCGCTGTA
AGGGTACAAACACCTTCCTGCATGACTCTCTCCAAAATGTGATTAATGTCTGTAGTTTGCCCAACATGCGCTGCCAGAATGGTGTGAAC
AACTGCCACCGGAGTGCAAGTCCTGTGAACAAGACTGTCTGCCGGCTCATTAGAGGGAGCACCAAACCTAACTGCCGCTACACGGCTAG
AGCCATGGTTGCAAACTTTGTTGTGGCCTGTAGTCCACCTCGGGCAGGTGACCCTCCGGATCCGCTGGTTCCTGTACACTTAGATTAG

>Hg-RNase6C
ATGATGCGAGATGTGAGATTTCCGCTCCTATTGCTGCTGGCACTCCTCTGTCCTCTTTGTAGTGCACCTCCGCCTCAGGGCTTCACCAG
TTTTCAGTGGTTTTCTACTCAGCACGTACAGCCAAATCAGAACATCTCATGCAATGTGGCAATGACAGCTATCAACCAACATCTCCCTC
GCTGTAAGCCTACAAACACCTTTCTGCATGACTCTCCCCAAAATGTGATTAATGTCTGTACTTTGCAAAGTGTGTGCTGCAGGAATTGT
CAGAACAACTGCCACCGGAGTGCACAACCTGTGGGCGCGACTGTCTGCCGGCTTACTAGAGGGAGCACCAAACCTAACTGCCACTACAC
GGCTAGTGTCATATTAACAGACTTCTTTGTGGCCTGTAATCCACGTCAGGCAGGTGACCCTCCGAATCCACTGGTTCCTGTACACTTAG
ATTAG

>Hg-RNase6D
ATGGTGCGAGATGTGAGATTTCCGCTCCTATTGCTACTGGGGCTATTTGTGCTACTGGGTCCTGTTTGGCCTGCACTTGGGGGCCACAC
CCCACTTTCATGGTTTGAGAACCAGCACATACTGAAAAAAAACCTCTCATGCGACAAGCTAATGGACGATATCAACAACTACAGGCCTG
GGTGTAAGGCATCAAACACCTTTCTTCATGTCTCTCTCCAAAGTGTGATTGATGTCTGTGCTTTGCCCACTATCCCATGCAAGAAGATA
AAAGGGAACATTTGCCACCGGAGCAGAGATCTTGTCCCTACGACTCGGTGTGTAATCAAGAATAAGAGGGATTCTTGCATCTACAATGA
ATTTTCCAAGTTGGCACACGTCACTGTGGCCTGCAAGCGTCAGTTCGATAGCCCTCTTTATCCACTACTGCCTTTTCACTTAGATTAA

>Hg-RNase6psA
ATGGTGCGAGATGTGAGATTCCCTCTCCTATTGCTGCTGGGGCTACTTGGGCTACTGTGTCCTCTTTAGGCTGTGGCTCCGGCCTTCGC
CCCATTTGGGTGGTTTGAAGTTCAGCAGTACACCCAAATGCTCTCACCTCATGCAGTGGGGCAACGAGAGATATCAGCCGACATCTCCC
ACACAGGGGCTGTAAGCCTACAAACACCTTTCTGCACGACTCTCTCCAAAATGTGATTTATGTCTGTACTTTGGCCAATGTCCCGTGCA
GCGATTGTCTGTACAACAACTGCCACCAGAATACAAGTCCTGTGAACAAGACTGTCTGCCGGCTCATTAGAGGGAGCACCCCTCCTAAC
TGCCACTACACAGCTAGTACTAGGTTCGCAAACTTCACTGTGGCCTGTAATCCACCTCGGGCAGGTGACCCTCTGGATCCGCTGGTTCC
TGTACACTTAGATTAGATTTGCTAA

>Hg-RNase6psB
ATGGTGTGAAATATGAGATTTCGTCTCCTGTTGCTGCTGGGGCTGTTTGCGCTACTGTGTCCTCTTTGGGCTGTGACTCAGGGCCTCAC
TGGGGCTCAGTGATTTCACATCCAGCACATTCAGCCCAGTGCTCTCCAATGCATGGGAGCAATGTTTCATGTCAACAGTTTCCTCCAGT
GTTGCAAGCCTACAAACAGCTTTCTGCATGACTCTTTCCAAAACATGGCTGTTACCTGTGCAGTGCCCAACATTACCTGCTGTAATGGA
AACAAAAATTACCACTGGGGTGCAAATCCTGTCAACCTGACTCTCTGCAGGCTCACTGGAGGGACGTTTCCTAACTGTTGCTAATCCCC
CAGATTGTTCGCTGTGGCCTGTAATTATTGCCAGAATGTTGACCCTGCCTCTCTGTTGCTGGCTCCTGTACACTTAAGAGTTTTGATTG

>Hg-RNase6psC
ATGGTGTGAGACATGACATTTCCTCTCCTGTTGCTGCTGGGGCTGTTTGCACTACTGTGTCCTCTTTGGGCTGTGCTCTGGGCCTCTCT
GGGGCTCAGTGTTTTGCCATTCAGTACATCCAGCCCAGTGCTCTCCAATGCCCTGGGGCAATGGTTCATGTCAGCAATTTCCTCCACTC
CTGCAAGCCCACAAACAGCTTTCTGCATGACTCTTTCCGCAACATGGCTGTTACCCTTGCAACGCCCAACATTGCCTGCTGTAATGGAA
ACAAAGATCCCCACTGGAGTGCAAATCCTATCAACCTGACTCTCTGCAGGCTCACTGGAGGGACGTTTCCTAACTGTCGCTATGCTGGA
ATCCCCCAGATTGTTCACTGTGGCCTGTAATTATCGCCAGAATGTTGACTCTGCCTCTCTGTTGTTGGCTCCTGTACATTTAAGAGTTT
TGATCGCTTTT

>Hg-RNase7/8
ATGACACCAGCCAGACCAAGATGCTTCTCCCTGCTGCTGCTCCTGCTGCTGGGGCTGTGGGTGGCAGAGATCCCAGTCAGTGCCAAGCC
CAGAAACATGACCTCAGCTCAGTGGTTTGAGACTCAGCACGTGCAACCCAAACCTCAAGCCTGCAACCCAGCAATGGACCGCATCAACA
AGTACACAAAGCACTGCAAACCCCTCAACTCCTTCCTGCATGCATCCTTCTCCAATGTGGCTGCCACCTGCCAGACCCCCAGCATACCC
TGCAAGAACGGCCATAAAAACTGCCATAAGAGCCCAAAGCTTGTGTCCCTAACCACCTGCAAGCATGCCTCAGGGAGGTACCCCAACTG
CAGGTACAAAGAGAAGCATCTGAATGCATCTGAATACATAGTGGCCTGTGACCCTCCCCAGAGCAAGGACTCTGGGAAATTCCACCTGG
TTCCTGTGCACCTGGAAAAAGTCATTTAG

>Hg-RNase7/8ps

ATGGCACCAGCCAAGACAGAGTTCCATTCCCTGCCACTGTTCCTGCTGCTGGGGCTGTGGGTGGCAGAGATCCCAGTCAGTGCCAAGCC
CAGAAACATGACCTCAGCTCAGTGGTTTAAAACTCAGCACATGCACCCCAGCCCTCAAGCCTGCAACTCAGTGATGGGCCACATCAAGT
ACACAAAGCACTGGAAAGCCCTCAGCTCCTTCCTGCATGAACCCATGCCCGTTGCAGCCACCACTTGCCAGACCCCCAACATAGCCTGC
AAGAAGGGACACAAAAACTGCCACCCGAGGCTATATCCCTGACCATGTGTCAGTATACTTCTAGAAGGTACCCAGACTGAAGATACAAG
GAAAAGTGTGTAGATGCATCTTGCATGTGGCCGGTGACCCTCCACAATCGAAGGATAAGCATGACACTCATTGGTTCCTGTGCATTTAG
ATGGTATTATCTAA

>Hg-RNase9
ATGCTGTTCACCAAGCACCCACTGCCTCTGCTGTTCCTGCTGCTGCTACAACCAATGCAGCTTCAAGGCACATTCTCAAATTTCTACCT
TTTACCAAATTTTACAAAGGAAGAGTTTGATAATTACATATATGAACTTTTTGGTACAGGGCCTACAAGACCACCTTCCAAAAGAAAAA
TTGAAAAAATGGTCCTTGTTGAGGAAGATTGGAGACCTTTACATGATCCTCAATACTTTACTGAGGAAACTAAATACAAAAATGTTCAT
TATAAGATGCGGTGTATGAACCACCATTACTTTCTTCAAACATCCTATGAGTTGCTGCAACAGACCTGTTACAATCTCATAGCACTATG
TAGTGATGGAACTAACATGTGTAAGATGAGCAACAAAACAGTAGAGGGGGTGTTTTGTAAGTTAACAGAAGGAACTACAATTCCAGAAT
GTGACTATGAATCTACTTACATGCATGGTTATGTCATTATTACTTGTCGATGGAACAATGAAACTCAAGAATTTATTCCTCATACTATA
AATAATATGGTACCACATACCTAG

>Hg-RNase10
ATGAAGCTGACCCTGGTACAGATCTTTGTCATCATGCTTCTGCTGCTACTGGGCCTGGGGTCGGGCCTAGGGCTGGGGCTTCAAATGGC
TGCAGCAGTCCTGGAGGACAGTGATCAGCCACTGAATGAATTTTGGTCCAGTGACTTACAGGACAAAGCTGAGGCCACTGAGGAGAGAA
AGGGCACTCAAACCCCAGAAACCCTGGTGCTTAGCAACAAAGCAATTGTGCAGCCTGGCTGGCCAGAAGAGACCGTCTTCAGTGAAGAT
GAGGTTGGAGGGAACCAAGGTCTCAGAGCGGAGGCCCGCTTTCAGAGCTACAGAGACTATCTTAGGCTTGACTTGACAGCCAGAGAATG
TAATGCCATGATGGCACCCAAGGTGATGCAGCGTAATCACAGTTGCATACCTGAGTACACATTCATCCATGAGGATCCCAAAATAGTCA
AAGCCGTCTGTAACAGTCCTGCTGTAGCCTGTGAGCTCAAGGGGGCCAAATGTCACAAAAGCCCTCGCCCTTTTGACTTAACATACTGC
AAGTTGTCTAAACTAAGCCAAGTCACCCCCAACTGCAATTACCTAACTTTCATTATGGAAAAGGTCATTCTTATAACCTGTAAGGACAT
GAAGCTCCAGTTAACAGCTATACAATGA

>Hg-RNase11ps
ATGGAGATCTTCTCTCTGCTGCTGCTTGGCCTAGGACTGATTCTTGCAGAAGCTTCAGAAAGTATAATGGAGATAAAGAAGAAGTTGCA
GAGGAAAAGATGCAACATGAGATAGTAAAAAGTGGCCAAGAAAAGACTGATGAGGTATTAATGAACTTGACTCTGTTTGATAAAAATGC
CAGCCTCAGCTTGTCCAAGGACATTATGCCTTCTTCATTACTGACATTCAGGTTATGTTATAGCATATCTAAAGGAAACAGTCCCTTTA
ATGACAAAGAGTGTTGCAATAACATGATAGTTTGGAGAAAGGTTTTAGAAGCTAATATGTCATTCAAATCAAGCAATAACTTCATCCAT
GGCCCGGGTGAAGTGATTCACAGGATCCACAAAGCCTCCAGCTGCAAGTGTGGACAGAATTCTGGCATACGTGGCTGTGAGAGCCTGGA
ACTGGAGAATACTGCGTGCCAGCTCACCACAGGCAAACAATTCCCCAGGTGCCAATACCACAGCGTTACTTCATTAAAGAAGATACTGA
CAGTGCTGGCAGGTCATCCTCTGATGAGCTGGTTGGTTGGTGGCTCTAAATTGTAA

>Hg-RNase12
ATGATACTAATGGTCATCGTTTTCTTGCTGCTTCTCTTCTGGGAAAAGGAGCCAGATGAGGATTTAGTGGTGAAGTCCATAGAGCACTT
GAATGTGGACTACCCAAAGAGCAGGATTCCTGTGAGGTACTGTAACTCCATGATCCGACAAAGAGTCATCAGGGAACCCAACCACACAT
GCAAAAAGGAGCATGTCTTCATCCACGAGAGGCCCCCAAAAATCAACAGCATCTGCGTTTCTCCCAGGAAGATGGTGTGCCCAAACCAT
GCTACCTATTTCTGCTTCCAGAGTGAGGCAAAGTTCAAAATGACACTCTGCCAACTCGTTGGCGGCACAATATATCCTGCCTGCAGGTA
CCACATTTCCACCTTGGAGGGGTATGTTCTTGTCACTTGTGATAACTTGGGGCCAGTTAATTTCCAGGGATATGTTGAGTAA

>Hg-RNase13
ATGGCACCAGCTGTGGCCCAGCTCCTTTTCCTTCAGCTTGTTCTAGGGCCAGCTCCGGTGGTGAACATCAAGGTGCATCCTGAAGCTCA
AAACTTCCATAACTTACACATCGACTATCCCAAGGTTACCTTCGCAGAGGGTTTCCAGGGCTACTGCAATGGCTTTATGGCCTATGTGA
GGGGCAGAAGGCAAAACTGGTTTTGCCCAAAGGTCCATTATGTGCTACATGCCCCCTGGAAAGACATCCAGAAATTCTGCAACTATACT
GACTACTTCTGTGATGTCTACAATGAATACTGCACACTTACTCGGGACTTCTTTTGGCTCACAACCTGCACCCTGGACCCTAAGCATCC
ATCCACTGGCTGTTCCTACACCACCAGTGTAACCAGACAAAGGGTCTATCTGGTCTGCTCCCGCAGGTATAAAGGTAGCCCAATAGGTA
TCATCAGCCTCATTTAG

Guinea pig (*Cavia porcellus*)

>Cp-RNase1A
ATGGAGAAGTGCCTTGTCCTGTTCCCACTGCTTGTCCTGGTGCTGCTGGGACTGGGGTGGGTACAACCTTCCCTGGGCGCAGAAAGCTC
GGCTATGAAGTTTGAGCGGCAGCATGTGGACTCAGGCGGTTCCTCTAGCAGCAATGCTAATTACTGCAATGAAATGATGAAGAAACGGG
AGATGACAAAGGACCGCTGCAAGTCACTGAACACCTTTGTGCATGAGCCCCTGGCAGAGGTGCAGGCTGTCTGCTCCCAGAGAAATGTC
TCCTGCAAGAACGGGCAGACCAACTGCTACCAGAGCTACTCCAGCATGCACATCACAGAGTGCCGCCTGACGAGCGGCTCCAAGTTCCC
CAACTGTTCATACCGCACCAGCCAGGCGCAGAAGAGCATCATCGTGGCCTGTGAGGGGAAACCGTATGTGCCTGTTCACTTTGACAATT
CTGTGTAG

>Cp-RNase1B
ATGGAGAAGTGCCTTGTCCTGTTCCCACTGCTCGTCTTGGTGCTACTGGGACTGGGGTGGGTACAACCTTCCCTGGGAGCAGAAAGCTC
GGCTATGAAGTTCCAGCGGCAGCACATGGACCCAGAGGGTTCCCCTAGCAACAGTAGTAACTACTGCAACGTAATGATGATTCGCCGGA
ACATGACACAGGGCCGCTGCAAGCCAGTGAACACTTTCGTGCATGAGTCCCTGGCAGATGTGCAGGCTGTCTGCTTCCAGAAAAATGTC
CTCTGCAAGAATGGGCAGACCAACTGCTACCAGAGCTACTCCAGGATGCGCATCACAGACTGTCGCGTGACGAGCAGCTCCAAGTTCCC
CAACTGCTCATACCGCATGAGCCAGGCGCAGAAGAGCATCATCGTGGCCTGTGAGGGAGACCCGTATGTGCCTGTTCACTTTGATGCTT
CTGTGGAGCCCTCCACCTAA

>Cp-RNase1C
ATGTCTCTTGTCCTGTTCCCACTGCTTGTCCTGGTGCTCCTGGGGCTGAAGTGGCTCCAGCTTACTCAGGACAAAGAATCCTCAGCCAT
GAGGTTCCAGCGGCAGCACATGGACTCAGACAGTACCCACATCAGCAACCCCAGCTACTGCAATGAAATGATGCAGTACCGGAACATGA

CACGGGGCCGCTGCAAGCCAGTCAACACCTTCGTGCACGAGCCCCTGGCAGATGTGCAGGCTGTCTGCTTCCAGAAAAATGTCCTCTGC
AAGAATGGGCAGAGCAACTGCTACCAGAGCTACTCCAGCATGCACATCACAGACTGTCGCCTGACGAGCAGCTCCAAGTTCCCCGACTG
CTCATACCGCATGAGCCAGGCGCAGAAGAGCATCATCGTGGCCTGTGAGGGGAACCCGTATGTGCCAGTCCACTTTGACACTTCAGTGG
AGCCCTCCACCTAA

>Cp-RNase1ps
ATGGCTCTGAAGATGTCCCTTGTCATGTGCCCACAATCATTCTGGCGCTCTGGGGCTGGGGTGTGTCCAGCCTCACCTGTGCAAAGAAG
CTACACCCACCAAGGTCCAGCAGCAGCACACGAGCTCAGGCAGCTCCCCCAACAGCATCCCCAACCACTATGGTGACGTGATGAAGCAC
TGGAAAACTTACTGCACTGATTGGCAGATGTGCAGGCTGTCTGCTTCCAGAAAATTGTCACCTGCAAGAATGGTCAGACCAGCTGCTAC
CACAGCCACTCCAGCATGCACAGCACAGAGTGCCACATGACAGGGAAATCCAAGTTCCCTGACTGCTCTTACCATTCCAGCCACGTGGA
AAGGAACAGCATCATGGCCTATGAGGGGAACTCGTATGTGCCAGTCCAATTTGATACTTCTACAGAGCCCTCCACCTAAGCCAGAGCAG
CGAGACACCCACCTCACCCCTTGTCATCA

>Cp-RNase4A
ATGGATCTACAGAGGACCCATTCACTGCTTCTGCTCTTGCTGCTGACCCTGATCGGGCTAGGGCTGGTGCAGCCCTCCTACGGCCAGGA
TCGCATGTACCAGCGGTTCCTGCGGCAGCACGTGGACCCCCAGGTGACAGGTGGCAATGATTATTACTGCAACTTGATGATGCAGAGAC
GGAGGATGACTAGTCCGAGGTGCAAGCCCTTCAACAGTTTCATTCATGAAGACATCTGGAACATTCGCAGTATCTGCAGCACCAGCAAC
GTCGAGTGCAAGAATGGTTTGATGAACTGTCATACAGGTGTTGTGAAGGTCACAGACTGCAGGGAGACAGGAAATTCCAGGGCCCCTAA
CTGCAGATATCGGGCTATGGCGAGGACCAGGCAAGTTGTCATTGCCTGTGAGGGTAGCCCTGAGGTGCCTGTGCATTTTGATGCATAG

>Cp-RNase4B
ATGGATCTACAGAGGACCCATTCACTGCTTCTGCTCTTGCTGCTGACCCTGATCGGGCTAGGGCTGGTGCAGCCCTCCTACGGCCAGGA
TCGCATGTACCAGCGGTTCCTGCGGCAGCACGTGGACCCCCAGGTGACAGGTGGCAATGATTATTACTGCAACTTGATGATGCAGAGAC
GGAGGATGACTAGTCCGAGGTGCAAGCCCTTCAACAGTTTCATTCATGAAGACATCTGGAACATTCGCAGTATCTGCAGCACCAGCAAC
GTCGAGTGCAAGAATGGTTTGATGAACTGTCATACAGGTGTTGTGAAGGTCACAGACTGCAGGGAGACAGGAAATTCCAGGGCCCCTAA
CTGCAGATATCGGGCTATGGCGAGGACCAGGCAAGTTGTCGTTGCCTGTGAGGGTAGCCCTGAGGTGCCTGTGCATTTTGATGCATAG

>Cp-RNase6A
ATGGTGCGAGCTGTGAGGTTTCCTCTCCTGTTGCTGCTGTGCCTGTTTGGGCTGCTGTGCCCTTTTTGGGCTGTGTCTCAGGATCTCAC
CCCATTTCAGATGTTTCAGCATAAACATGTACAGCCAGACCCTCGCCCATGTGACCCGGCGATGACTGCTGTCAATGAATTAGAAAAGA
ATCATAAGTGCAGGCCTACAAACACCTTTCTGCATAACTCTCTCCAGAATGTGATTGATGTGTGTACACTGCCCAACCAGCTCTGCAGG
AATGGCCAAAACAACTGCCACCAGAGTATACATCCTGTGAAGATGACTGTCTGCCAGTTAACTAAAGGTCAGTACCCGAACTGCTACTA
CAGGACTGATTCCACTCTCAAGAACTTCACTGTGGCCTGTGAGCCACCTCAGCAGGAGGATCCACGTCAGTACCCTCTGGTTCCTGTTC
ACTTTGACGCTATTGTCCACAATTCCCAGCGGTTTTCCTCTGTCTTGACAGGCAGTTGCTATTATAGTTTGTTCTGCATTTTTGGTCTG
CTCTTTTTCCTTCTTCATCTTCTTTTTTGA

>Cp-RNase6B
ATGGCACGAAATGTGACATTTCCTCTCCTGTTGCTGCTCGGGCTATTTGCGATACTGTGTCCACTGTGGGCTATGGGTCCACAACCACC
AGCGGGCTTCACTCCAGTGTCATGGTTTCAAAAGCAGCACATACTAGAGAAACCAAAGTCATGTCAAGCAGCAATGAATGATATCAACC
AGTTTCTTAATGGTCTCTGTAAGAGGTCAAACACCTTCCTTCATGTCTCTCTCCAAAATGTGAAGGATGTCTGTGCTTTGAAAGCGTTT
CAGTGCAGGGATAAATCGACCACATGCCACAGGAGTCCAGCTCCTGTTCAAATGACTGTATGCAAGATCATGAAAGATACAAATTCTTG
CACCTATAGAGAGAGTCACACGACCGAGCACTTCACTGTGCAGTGCATGCATAGGTTTAATGACCCTAAGATGGAGCTGCTTCCTGTTC
ACTTAGAATAA

>Cp-RNase6C
ATGGAGCGAGATGTGAGGTTTCCTCTCCTGTTGCTGCTGGGGTTGTTTGGGCTTCTGTATCCACTGTGGGCTTTGCCTAAGGGCCTCAC
CAAGGCTCAGTGGTTTGAGATCCAGCACATACAGCCAATTCCTCTCCAATGTGATGGAGCAATGAGCGATGTCAACAATTATACCAAGC
GCTGTAAGCCTTTAAACACTTTTCTGCATGACTCTTTCCAAAATGTGAGTGATTCCTGTACCTTGCCCAATATCACCTGCAAGAATGGC
CAGCACAACTGCCACCAGAGTGCAAGTCCTGTCAGCCTGACCAACTGCAAGCTCACTGGAGGGAAGTATCCCAACTGTCGCTACAAGAA
TGCTTCCCAGTTCAAATTCTTCATTGTGGCCTGTGAACCACCTCAGAAGAATGACACTTCCTATCCGTTGGTTCCAGTACACTTAGATA
AGATTGTCTGA

>Cp-RNase6psA
CCTTTTCCTACACAGACCAGAAGGTGCAAGATATAAGATTTCCTGGAGGGGCTATTTGGGTTACTGTGTCCTCTTTGGGCTGTGCCTAA
GGGGCTAACCAAGGCTAAGTGACTTGAAACCCAGTACCTACAGCTAGGAAGAATCCCATGCAACAATTCAATGCTTCATATCAACATGG
GTCTGTTAAGCCCATATACATCTTTCTCTCTGACTCTCCAAAATGTGACTGGTGTCTGCTTTGTGCAACATCTAGTGCAAAATGGCCAG
AACAACTGCCACCAGAGGACAAATCCTATTAGTGTGACTTTCTGGCAGCTCACTGGGACCTATCCTTACTGCCACTACATCAATGATGC
CCACTTCACATACTTCACTGTGGCCTGTGAGCATCTGAATGGTGACTCTTGCCATCCACTGGTTCATGATCACTTAATTTTGGAAAGTT
TTGA

>Cp-RNase6psB
ACACAGAAAATGGTTGGAGATGTGAGATCTCCTCTCCTGGAACTGCTAGGCCTATGTGAGCTACTGTGCCCTCTTTGAGCTGTGCCCCA
GGCTCAGCAGGGCTCAGTGGTTTGCCATTCAACACATCCAACCAATTTGATGCTAATGCCCTAAGGCAGTGAGTGGTGTCAATAGTTAT
ACTCAGCAATGTACACCTACAAACACCTTCCTGTGTGACTCTTTCCAAAACATGAGTGATTCCTGCACCTCGGCCAACATCACCTGCAT
GAATGATCAGAAAAACTGCCACCAGAGTGCAAGTCCTGTCAACCTGATCATCTGCAGGCTCCCCAGAAGAGAGTACCCACTCTGTCAGC
ACAAGGATGTTACCCAGTTCAAATTGTTCACTGTGGCCTGTGAACCACCTCAGAAGAGTAACATTTCCCCTCCCTATCTAGTGGTTCCT
TATGCTTAGATAAGATTGTGAGA

>Cp-RNase6psC
ACAGACATGAAAGTGTGAGATATGACGTTTCCTCTCCTGTTTCTGCTGGAGCTGCGTGGGCTCCTGTGTCCTCTTTGGACTGTGCCTCT
GGGCCTCTCTGGGGCTCAGTGGTTTGAAATCTAGCTCATACAACCACGTCCTCTTCAATCTTGGCAGCAGCGTTGGGTGCCAGTATTTC

CTCGGCAAGTCTGCAAACAGCTTTCTGCATGACTCTTTTCCCAACATGGCTATTACTTGTGCTATGCCCAGCGTTATCTGCATTAATGG
AGACAAAAATTGCAAATCTTTTCACCCTGATTCTGCACGTTTCTTGGAGGGATGTTTCTTAACTGCCATGACACTTGAAGTCCTCATTT
CAAATTGTTCACTGGGTCTGTATTCATCACCCCCTCTCCACTGCTGGCTTCTGTACAGTTTTGATTGCTTTTTTTTTTTTTTAATCTTT
CGAC

>Cp-RNase7/8
ATGACACAAGCCAGAGCAAGATGCTGCTCGCTGCTGCTGCTCCTGCTGCTGGGGCTGTGGGCGGCAGAGCTCCCAGTCAGTGCCAAGCC
CAAAAGCATGACCTCGGCTCAGTGGTTTGAAACTCAGCACGTGCAGCCCAACCCTCAAGCCTGCAGCTCAGCGATGGGCCACATCAACA
CGTACACAAAGAGCTGCAAACGCCTCAATGACTTCCTGCACACGTCTCTCTTCAACGTGGCTGCCACCTGCCAGACCCCCAGCAAAACC
TGCAAGAATGGTGGTAAAAACTGCCACCAGAGCCCGAAGCCTGTGTCCCTGACCACATGTAAACTTGCCTCAGGGAAGTACCCCAACTG
CCATTACCAAGAGAAGCATCGGAATGCACGTTACGTAGTGGCCTGTGAGCGACCCCAGAAAAAGGACTCCGGGAAATTCCGCTTGGTTC
CTGTGCACTTGGATGAAGTCATTTAG

>Cp-RNase7/8ps
AAAGCACCAGCCAGGACAGAGCTCCATTCTCTGCTGCTGTTTCTGTTGCTGGGGCTGTGGATGGCCGGGGTCCAGACAATGAGAAGCCC
AGAAACATGATCTCAGCTCAGCGGTTTGAAACTCACAAGCCCCCTGGCCCTCAAGCCTGCAGCTCAGCAATGATGGGCCACATCGACAA
GTACACAAAGCACCACAAACCCCTCAACACCGTCCTGCAAACACTCTTCTCTAGTGTGGTTGCTACCTGCCAGACCTCCATCATAGCCT
GCAGGAAGGGATATAAAATCTGCCACTGGAACCTGTGTCCCTGGCCAGGTGTGAGTATACTCCTGGGAGGTACTCAGACTACAAGTGGA
AGGAAAAGTGTTCGGATGTGCCTTACTATGGGGCCTGTGACCTTCTACACTGGAAGGATAATCACAACACTCATTGGCTCCTGGACATT
TAGGGAGTATTATCTAA

>Cp-RNase10
ATGAAGCTGACCCTGGTACAGATCTTTTTCATCATGCTTCTGCTGCTGCTGGGCCTGGGGCTGGGCCTAGGACTGGGACTGCACATGGC
TGCAGCAGTCCTGGAGGACAGTGACCAGCCACTGAATGAGTTTTGGTCCAGTGACTCACAGGACAAAGCTGAGGCCACTGAGGAGGGAG
AGGGCACTCCAACCCCAGAAACCCTGGTGCTTAGCAACAAAGCAGTTGTGCAACCTGACTGGCCAGAAGAGACCATCCTCAGTGAAGAT
GAGGTGGGAGGGGACCCGGCTCTCAAAGCGGAGGTCTCCTTTCAGAGCTACAGAGACTATCTTAGGCTTGACTTGACGGACAGAGAGTG
TAACACCCTGATGGCGAACAAAGTGAAACAGCCCAATCAAAGTTGCGTATCTGAGTACACATTCATACACGAGCATCTTCACACAGTCA
AAGCTGTCTGTAACAGCCCTGTTGTTCCCTGTGAGCTCAAGGGGGGCCAAATGTCACAAAAGCCCTCGTGCTTTTGACTTGACGTACTGC
AAGTTGTCTAAACCAGGCCAAGTCACTCCTAAATGCAATTACATAACCTTCATTATGAAAAAGGTTATTCTTATAACCTGTAAGGACAT
GAAGCTCCATTTAACAGCTATACAATGA

>Cp-RNase11
ATGGAGACCTTCTCTCTGCTACTGCTTGGCTTAGGGTTCATTCTTGCAGAAGCCTCAGAAAGTATAATGGGAATAATTAAAGAAGAATT
TGCAGAGGAAAAGATGCAACCTGAGACAATAAAAAGTGGCCAAGAAAAGGAGACTGATGGGGTGTTAATGAATTTAACTCTGTTTAATA
AAAATGACAGCCTCAGTCTGCCCAAGGATGCTATGCCCTCTTTACTACTGACATTTAGGTCATGTTGCAGCATCCCCAAAGGAAACAGT
CCTGTTAGTGACAAAGAGTGCTTTGGTAACAAGATGACCTGGAGCAAATTTTCAGAAGCTAATGAGACGTACGAGTTAAACAATTTTAT
CCACAGCCCTGCTGAAGTGATTCACAGAGTCCACAAAGCTTACAGCTGCAAGCGTGGGAAGAATTGTGGCATATGGGTCTGTCAGAGCC
CAGAACTGGAGAATACTGTGTGCCAGCTCTCCCCAGGCAAACAATTCCCCAGGTGTCAATACCACAGCATTACCTCATTAAAGAAGATA
TTGATGGTTCTGGCAGGTCATTCTCTGATGAGCTGGTTGGTCAGTGGCTCTAAATTGTAA

>Cp-RNase12
ATGACACTAATGGTGCTCATTTTCTTGCTGCTTCTCTTCTGGGAAAGGGAGCCAGCTGAGGAAATAGTGGTGACATCCATAGAGCACTT
GCATGTGGATTACCCAAAGAGTAATCCTATAAGGTACTGCAACTCCATGGTCCTACAGAGAGTCATCAGGGAACCCAACGACACATGCA
AAAAGAAGCATGTCTTCATCCATGAGAGGCCCCAAAAACTCAATAGTGTCTGCATTTCTCACAGGAAGATGGTTTGCCCAAGCCAGTCT
ACCATTTTCTGCTTCCAGAGTGAGACAAAGTTTAAAATGACACTCTGCCAACTCATTGGTGGCATAACATATCCTGCCTGCAGGTACCA
GGTTTCTGCCTTGGAGGGGTATGTTCTTGTCACTTGTGATAACTTGGGGCCAGTTCATTTCCACGGATACATTGAGTAA

>Cp-RNase13
ATGGCATCTGCTGTGGCCCAGCGCCTCCTACTTCTTCAGCTTGTTCTAGGACCAACTCTGATCACAGACCTCAAGCTGAGTCCCGCCGC
GCAAGACTTCCTCAAGTTACACATCGACTACCCCAAGGTTACATACGCAGATGGTTTCCGTGGTTACTGTAACGGATTTATGGCTTATG
TGAGGGGCAGAAGACAGCATTGGTTCTGCCCAAAGATCCATTATGTGCTGCATTCCCCCTGGAAAGACATCTGGAAATTCTGCAATTTT
ACCGACTACTACTGTGACGTTTATAATCAATATTGTACACTCACTCAGGACTCTTTTCCCCTCACAATCTGCTCCCTGGCGCCTGAACA
GCCATCTACCAGCTGCTTCTACAACACCAACACATCCAGACAAAGGCTCTATCTGCTTTGCTCCCGCAAGTATAAAGGTCACCCAATAG
GTATCATCAGCCTCTACCAGGGAATGTAA

>Cp-RNase15ps
TTTCTCTTTTCATGAGCAAAAAATGTGCTGTGACACAGACCTGCCTCATGCCCCTCCTCATGAGGCCCATGACTTCCTCCTTCATTGAT
GGATGGGTCCAAAGTATCACTGAGCAGGCACAGACCTTCCTGTGATGTGATCCTGAGGGGTCGAGGGCTGGCTATGAAGAACAGCAGTG
AGAAGACCTCCCCGTGTGTTCCCTTGTGGTAGAGAACTGTCCAAGGCCCAGCTGTGAGGCATGTGAGGTGCCCAGCTATAGCGGCACTG
TGTCACCTTCAAAGCCGCTCAGTGAATGCTGCCTTGGGTCTCGTCCCAACCTGTGCCTTCCAAGCCATTTCCAGAAGGCACATTTGGGT
GGTCTGTGTGGCCTAGTGTGTCTGGGTGACAGTATGCTTCC

Rabbit (*Oryctolagus cuniculus*)

>Oc-RNase1
ATGGCTCTGGACAGACCCCTCATCCTACTTGTCCTGGGGCTGCTGATGCTGGGATTGGCACAGTCTGTTTTGGACAACGAGTCCCCGGC
CAAGAAGTTCCAGCGGCAGCACATAGATCCAAAACTTCCCCTCAGCAGCACCTACTGCAACAAAAAGATGGAGCAACAGGACATGACAC
AGGGCTGCAAGCCACTGAACACCTTTGTGCACGAACCCCTGAAAAAGATCCAGGCAGTCTGCTTCCAGGAGAAGGTCACCTGCAAGGAC
GGGAAGACCAACTGCTACCGGAGCACCTCCAAAATGCACACCACAGACTGCTCCCTGTTAGATACATCCAAGTACCCTGACTGCAAGTA
CCAGACCGTTCAGAAAGAAAGATACATCATTTTAGCTTGTGAGGGGAACCCATTTGTGCCAGTCCACTTTGACGCTTCTGTGGAGTAA

>Oc-RNase2/3A
ATGCTCAGTGCCCGACTTTGTTTCTTGCTGTTGCTGGGGATCTTGGGAACAGCAACCTCATTCCAGGCCCCACCTGCTGGTTTCACACG
GGCTCAGTGGTTTGAAGTCCAACACATAAATATGACGCACTCCCGATGTGACAATGCAATGAGAGTGGTTAACAGATATAGAGGATACT
GCAAAGGGAAAAATACTTTCCTTCATGTAACCTTTGTGGATGCTGTGAATACCTGTCATAATCCAAATATACCCTGCACCGCAGGTGGT
AGGCAAAATTGCCATCGGAGCTCAACCCAGGTGCCTTTAACTGACTGTGACCTCACAAGAAACAGTTCAAATATTAGGCAGTGCCAATA
TCGACGGAGAAGGAAAACTAAATTCTATGTAATTGCCTGTGACCCAAGATCTCCCCGGGACCCTCCTTCCTACCCTATAGTTCCAGTTC
ACTTGAATGGGACGGTTTAG

>Oc-RNase2/3B
ATGGCTTCAAAGCTGGACAATTTCCAACTATGTCTCTTGCTGTTGCTGGGGATCTTAGGAACAGCTGTGTCATTCCAGGCCATCCCTCC
TAATTTAACCCCAGCTCAGTGGTTTGCAATTCAGCACATAAAAATGGCCAACAGCATTGTATGTGGCAGTGCAATGCGGGTAGTAAACA
GTTACACAGGACACTGCAAAGGCAAGAACACTTTTCTTAATATAACTTTCAGTGATGCTGTGGGTACTTGTGTTCACACCCCAAATATG
CGCTGCCTGAGAAGCTCCAGGACAAATTGCCATCAGAGCTCACGCCGGGTACCTTTGACAGACTGTATCCTGACCAGAAATGCAAAGAA
CTACAGGCTTTGCACATATAGAAAGTTAACAAAAAATAAATCCTATGTAATTGCTTGTGCCCACAAATCTCCGAACGATTCTCCTGTAT
TCCCAATAGTTCCAGTTCACTTAGATGGGACAGTGTAG

>Oc-RNase2/3C
ATGGCTTCAAAGCTGGACAATTTCCAACTATGTCTCTTGCTGTTGCTGGGGATCTTAGGAACAGCTGTGTCATTCCAGGCCATCCCTCC
TAATTTAACCCCAGCTCAGTGGTTTGCAATTCAGCACATAAAAATGGCCAACAGCATTGTATGTGGCAGTGCAATGCGGGTAGTAAACA
GTTACACAGGACACTGCAAAGGCAAGAACACTTTTCTTAATATAACTTTCAGTGATGCCGTGGGTACTTGTGTTCACACCCCAAATATG
CGCTGCCTGAGAAGCTCCAGGACAAATTGCCATCAGAGCTCACGCCGGGTACCTTTGACAGACTGTATCCTGACCAGAAATGCAAAGAA
CTACAGGCTTTGCACATATAGAAAGTTAACAAAAAATAAATCCTATGTAATTGCTTGTGCCCACAAATCTCCGAACGATTCTCCTGTAT
TCCCAATAGTTCCAGTTCACTTAGATGGGACAGTGTAG

>Oc-RNase4
ATGATGACTCTGCAGAGAACCCATACACTGCTGCTTCTACTGCTTTTGACCCTGCTGGGGCTGGCACAGCCTTCCTTTGGCCAGGATCG
CATGTACCAACGATTCCTGCGGCAACACGTGCACCCTCAGGAGACAGGTGGCAATGATAGCTACTGCAACCTGATGATGCAGAGACGGA
AGATGACTTCACATCACTGCAAGCCCTTCAACACCTTCATCCATGAGGACATCTGGAACATCCGTAGTATCTGCAGCACCACCAGCATC
CGGTGCAAGAATGGCAAGATGAACTGTCACGAGGGGGTAGTGAAGGTCACAGACTGCAAGGAGACAGGAAGTTCCAGGGCCCCCAACTG
CAGATACCGCGCCGCAGCAAGCACTAGGCGTGTTGTCATTGCCTGTGAGGGCAACCCAGAAGTGCCTGTGCATTTTGACAGATAG

>Oc-RNase5
ATGGTCATGGGCCTGGGTCCGTTGGTGTTGATCTTCGTGCTGGGTCTGGGTGTGACCCCTCCGACCCTGGCGCAGGACGACTCCAGGTA
CAAACACTTCCTGACCCAACACTATGATGCCAAGCCATTTGGCCGGAATGACAGATACTGTGAAACCATGATGAAGAGACGAGACCTGA
CCTCACCCTGCAAAGACACCAACACATTTGTTCATGGCAACAAGGGCAGCATCAAGGACGTCTGTGAGGATAAGAATGGAAAGCCTTAT
GGGAAAAACTTCAGAATAAGCAAGTCTTCTTTCCAGGTCACCACTTGCAAACACGTGGGAGGGTCCCCCTGGCCTCCTTGCCGGTACCG
AGCCACATCAGGGTCCCGAAACATTGTTATTGCCTGTGAAAACGGCTTGCCTGTGCATTTTGATGAGTCCGTTTTTCAACAAAAAGCCC
ACTGA

>Oc-RNase5ps
AGAGATTTCAAGAGACCAGGCCCTATACTGTTGACTTTCACACTGGATCTGAGTGTGACCCTGCAAACCCTGGCTCAGGATGACTCCAG
TTACCAATACTTCCTTACCAGGCACTGTGATGTCCAACTAAAGTGACAGACACTGTGAAAATTTAACACACCTGGCCTCACCCTTCAAG
GGCAGCACACATTTGTTCACGGCAACAGGGGCAACACTAAGGCCACCTATGGAAATAGTGACGGGAACTCTCATGGATAAAGAAGTGTC
CTCTCCAGGGTACCACTTTTTTTTTTTTTTAACAGGCAGAGTTAGACAGTAAGAGAGAGAGACAGAAAGGTCTTCCTTCCGTTGGTTCA
CCCCCCAAATGGCTGCTATGGCTGGCGCACTGCGCCAATCCAAAGCCAGGAGCCAGGTGCTTCCTCCTGGTCTCCCATGCAGGTGCAGG
GACCAAGCACTTGGGCCATCCTCCACTGCACTCCTGGGCCACAGCAGAGA

>Oc-RNase6
ATGGTGCTAGATCTTCAGGGATGTTTGCCCCTCTTCCTATTGCTGCTGCAACTATTGGGTCCCATGTGTTCACTTTGTGCTTGGCCTAA
GTACCTCACCAAGGCTCGTTGGTTTGAAATTCAGCACATTCAGCCAAATCTTCTCCAGTGCAACAGGGCGATGAGAGGTGTCAATAATT
ATACACAGCACTGCAAGCCCTTCAACACTTTTCTGCACGACTCTTTTCAGGATGTGGCTGCTGTCTGTGATTTTCCCAATGTCACCTGC
AGGAATGGTAGGCACAACTGTCACCAGAGCCCAAAGCCTATCAACATGACCAACTGCAGACTTACTGCGGGGAAGTATCCTGACTGCTC
CTACAGTGATGCAACCCAGTACAAATTCTTCATTGTCGCCTGTGACCCCCCTCAGAAGAGCGACCCTCCCTACCACTTGGTTCCTGTAC
ACTTAGATGAGATTGTCTAA

>Oc-RNase7/8A
ATGGCACCAGCCAAGACAGAGTTCCGATCCCTGCTGCTGCTCCTGCTGCTGGGCCTGTGGGTGGCCGAGTACCCAGTCACTGCCAAGCC
CAAGGACATGACCTCCGCTCAGTGGTTTGAAACTCAGCACGTGCAGCCCACCCCTCAACCATGCAACTCAGCGATGAGCCGCATCAACC
AGTACACAAAACAATGCAAAAACCTCAACACCTTCCTGCACGAGTCCTTCACCAGCGTGATCCCTACCTGTGACACCCCCAATGTAGCC
TGCAAGAATGGTCGTAACAACTGCCACCAGAGCCCGGGGCCCGTGTCCATGACCAATTGTCAGCATGTGTCAGGGTCGTATCCAGATTG
CAAGTACAAGCAAACAGAAAAATATGCATCCTTCATTGTGGCCTGTGACCCTCCACAAACAAAAGACGACTCATCATATCCACTGCTCC
CTGTGCACTTTGATAAAGTTGTGTAA

>Oc-RNase7/8B
ATGACACGAGCCCAGGTAGGATTCTGCTCCCTGCTGCTGCTCCTGCTGCTGGGCCTGTGGGTGGCGGAGCGTCCAGTCACTGCCAAGCC
CAAGAACATGACCTCGGCTCAGTGGTTTGAAACTCAGCACGTGCAGCCCACCCCTCAACCATGCAACTCAGCGATGAGCCGCATCAACC
AGTACACAAAGCATTGCAAAAGCCTCAACACCTTCCTGCACGAGTCCTTCACCAGCGTGATCCCTACCTGCAACACCCCCAACGTGGCC
TGCAAGAATGGTCGTAACAACTGCCACAAGAGCCCACAGCCTGTGTCCCTGAGCACGTGTGCCCATGTCTCAGGGTCATATCCAGGCTG
CAAGTATAAAGAGAAGCACCTGAAGAAAGCTTACATAGTGGCCTGTGACCCTCCCCAGAAAGGGGACTCTTGGCATTCCAAGCTGTTGC
CTGTGCATTTGGACAAAGTCTTTTAG

>Oc-RNase9
ATGAAAATTCTCATCTCCAAACATCCGCTGCCTCTAGTGCTACTGCTGCTGCAGCTGCTACAGCCACTACAGTTTCAAGACAATGAGAG
ACGCTACGATTTGCCACCTGATAAAGCAGATGAATTTGAAGATTATTTGGAAGAATTTAGTAGTACAGGGCCTACCAGACCACCTACCA
AAAAAAAATTCATAAGGCTTTCCCTTATTGGAACAGAAAGGACATTAAGTGATTCCTTCTTCTGCACTGATACAGTCAGGCTAAAAAAT
GTTCACAACAAGTCTCGTTGTGTGACGGAACATAATTTCATCGTAATGCCATACGATGATGTGAAAAACCTCTGTTACACAAGGTATGT
CGAGTGTAAGAATGGGATCAAGAAATGTCACAGAAGCAGGCTGCCGATAGAGGGAGTGTATTGTAAATTAAGAAGAGGGACCACTCTAC
CTGACTGTGATTATGATTCAACTTATAAGATGGGCTTTGTCCTAATTACTTGTCGATGGCAAAATAGTATCAAAAAACTTGTTCCTGTT
CATGTAAATGATATCCTGGTGCTACAGGATTGA

>Oc-RNase10
ATGAAGCTGACTCTGGCGCAGATCTTCTTCATGCTACTGCTGCTGCTTCTGGGCCTGGGGATGGGCCTGGGGCTGGGTCTTCGCATGGC
CGCAGCCGTCCTGGAGGACAGCAGCCAGTCGCTGGAAGAGTTTTGGTCCGGTGACTTTCAGGACAGAGCTGAGGCCATTGAGAAGGCAA
AGAGCGCCCGAACCACAGAAACCCCTGTGCTTAGCAACAATGAAGTAGAGCAGTCTGGCTGGCAAGAAGACAGCATCCTCGGCGAAGAT
GAGGTTGCAGGAAACAAGGTGCCCAGAGCGGGGACCCCCTCTCGGGGCAATAAAGAGTATCTGCGGTTTGACTTGCTCTACAGGGAATG
CAACACCCTGATGGCAGAGAAGGTGAAGGAGAGCAATCGCAGCTGCATAACACAGTACATCTTCATCCACGAGGAACCAAAAACGGTCA
AAGCTGTCTGTAGCAGTCCTGCTGTTGCCTGTGAGTTCATGAAGGGCAAATGTCACAAAAGCCCCCGGCCTTTTGATTTGACATTCTGC
AAGTTATCCAAACCAGGCCAACTTACCCCTCACTGCAATTATCACACTTTTGTTCTGGAAAAGCACATTTTTATAACCTGCAATGACCA
GAAGATCCAGATAGCATCCGGACAATGA

>Oc-RNase11
ATGGAGACCTCACTGCTGCTGCTCAGCCTGGGACTGGTTCTCACAGGAGCTTCCGAAAGCACAGTGGAGGTAACTAAAGAAGAATTTGC
AGCGAAGAAAGTGCAATATGCCTTGGCAAAGAGCGGTCGAGAAACACAAACTGATAAGGTATTAATGAACTTGACCTTGTTAGATAAAA
ACACCAGTCCCATCCTGTCCAAGGATATGATGTCTTCCTCCTTACTGACACTCAGGAAGTTACAGTATAGAATCCCCAAGGTAAACAGT
CTGAGTAGTGACAGGGAATGCTGCAATGAAGTGACTATCTGGAGAAAAGCTTCCGAAGCCAACGGCTCTTGCAGGCTGAGCAATGACCT
CACCTGTGGCACCATGGAAGGGATCCGTGGGGTCCAGAAGATGCCCAGCTGCAAGTGTGGAGAGAATCCTGGCGTAAGCTGCTGTCAGA
GAGCGGAACCGGAGAAGACCATGTGCCAGCTCCCCACGGGCAGACAGTTCCCCAGGTGCCGGTACCACAGTGTTACTTCACTGAAGAAA
CTGCTGACGGTGCTGACCGGCCATTCTCTGATGAGCTGGTTAGTCAGTGGCTCTAAACTCTAA

>Oc-RNase12
ATGGTTCTAATGGTGATCATTTTCTTGATGCTTCTGTTCTGGGAAAATGAGCTAAATGAGAACGGAGTGCTGTTCACCTTGGAGCACCT
GCACGTGGACTACCCGCAGGACAGTGCTCCTGTCCGGTACTGCAACCACATGATCCAGCAGAGACTCATCAGGGAGCCCGACCACACGT
GCAAACTGGAGCACGTGTTCATCCACGAGAGGCCCCAGAAAATCAACAGCATCTGCATGTCTCCCAAGAGGGTGGCTTGCCTCAACCAT
TCCACCATATTCTGCTTCCAGAGCGAGACCAAGTTCAGAGTGACGGTCTGTGAGCTCATTGAGGGCACGCGGTACCCCGCCTGCAGGTA
CCGCATTTCCCCCACGCAGGCCTTCATCCAGGTGACCTGCGATGACTTGGGGCCGGTTAGTTTCGAGGGCTATGTTGAATGA

>Oc-RNase13
ATGGCAGCAGCTGTGGCCCGGCTCCTTTTCTTCCAGCTTTTTCTAGGGCCAGCTCTTTCCCTGGACATCAATATGCAGTCTGACACCGA
GAACTTCCGCACCTTATACATCGACTATCCCAAGGTTAAGTTCATAAAGAGCTTCCTGGGCTACTGTAATGGTATGATGGCCTATGTGA
GGGGAAGAATAGAGCACTGGTACTGCCCAAAGATCCATTATGTGATACATGCCCCCTGGGATGCCATCCAGAAGTCCTGCAAGGACAGC
AAGAGCTTCTGTGAGAACTACAACAAATACTGCACTCTGACCCAGGACTCCTTCCCTGTCACGGTCTGCAGCCTGGGCGCCAAACAGCC
CCCCACAAGCTGCCGCTACAACAGCACCCTAACCAACAAATATCTGTACCTGCTCTGCTCTGGCAGGTATGATGCTGAACCAATTGGTA
TCGTAGGCCTCTACTAG

Cow (*Bos taurus*)

>Bt-RNase1_brain
ATGGCTCTGAAGTCCCTGGTCCTGTTGTCACTGCTGGTCCTGGTGCTGCTGCTGGTGCAGGTCCAGCCTTCCCTGGGCAAGGAATCTGC
GGCCGCCAAGTTCCGGAGGCAGCACATGGACTCTGGCAGCTCCTCCAGCAGCAACCCCAACTACTGCAATCAGATGATGAAGCGCCGGA
GGATGACACATGGACGATGCAAGCCAGTGAACACCTTTGTGCACGAGTCCCTGGACGATGTCAAGGCCGTGTGCTCCCAGAAAAACATC
ACCTGCAAGAATGGGCATCCCAACTGCTACCAGAGCAAATCTACCATGAGCATCACAGACTGCCGCGAGACAGGCAGCTCTAAGTACCC
CAACTGTGCCTACAAGACTAGCCAGAAGCAGAAATACATCACTGTGGCTTGTGAGGGAAACCCATACGTGCCAGTCCACTTTGATGGGG
CGGTGCTCTTACCTGCCACACCCGTACCCTCACTGCCACCTCCACACAGGCTTCTCTGA

>Bt-RNase1_seminal
ATGGCTCTGAAGTCTCTAGTCGTGTTGCCACTGCTGGTCCTGGTGCTGCTGCTGGTGCGGGTCCAGCCTTCCCTGGGCAAGGAATCTGC
AGCTGCCAAGTTCGAGCGGCAGCACATGGACTCTGGCAACTCCCCCAGCAGCAGCTCCAACTACTGCAACCTGATGATGTGCTGCCGGA
AGATGACCCAGGGGAAATGCAAGCCAGTGAACACCTTTGTGCATGAGTCCCTGGCCGATGTTAAGGCCGTGTGCTCCCAGAAGAAAGTC
ACTTGCAAGAATGGGCAGACCAACTGCTACCAGAGCAAATCCACCATGCGCATCACAGACTGCCGCGAGACTGGCAGCTCCAAGTACCC
CAACTGCGCCTACAAGACCACCCAGGTGGAGAAACACATCATAGTGGCTTGTGGCGGTAAACCGTCCGTGCCAGTCCACTTCGATGCTT
CAGTGTAG

>Bt-RNase1_pancreatic
ATGGCTCTGAAGTCCCTGGTCCTGTTGTCGCTGTTGGTCCTGGTGCTGCTGCTGGTGCGGGTCCAGCCTTCCCTGGGCAAGGAAACTGC
AGCAGCCAAGTTTGAGCGGCAGCACATGGACTCCAGCACTTCCGCTGCCAGCAGCTCCAACTACTGTAACCAGATGATGAAGAGCCGGA
ACCTGACCAAAGATCGATGCAAGCCAGTGAACACCTTTGTGCACGAGTCCCTGGCTGATGTCCAGGCCGTGTGCTCCCAGAAAAATGTT
GCCTGCAAGAATGGGCAGACCAATTGCTACCAGAGCTACTCCACCATGAGCATCACCGACTGCCGTGAGACCGGCAGCTCCAAGTACCC
CAACTGTGCCTACAAGACCACCCAGGCGAATAAACACATCATTGTGGCTTGTGAGGGAAACCCGTACGTGCCAGTCCACTTTGATGCTT
CAGTGTAG

>Bt-RNase2/3A

ATGGTTCCAATACAGCAGGATTCTCGGCTTCGTCTCATTTTGCTGCTGGGGCTCTTGGGAATGGTGATCTCACTCCATGCCCAACCTGG
TACTTTAACCCGGGCTCAGTGGTTTGAGATTCAGCACATAAATATGGCCCACCATCAATGTAATGCCGCAATGAGAGTGGTTAACCGTT
ACAGAATGGTATGTAAAGGTAGAAATACTTTTCTCCACAGAACATTTGCTTATGTAGCTGGTATTTGTAACACCCCAAATGTAACCTGC
TCTAAACCAGGCAGGATGAACTGTCATAATAGCTCAGTCCAAGTGCCTATAACCTTCTGCAACCTCACAAGAAATGCAATGAACTACAC
AAACTGCCGTTACGGACGGACAAGGGCACGGAAGATCTTCATCGTTGCCTGTGAGAAGAGATCACCTCGGGACAGTCCCAGGTACCCTG
TGGTTCCAGTTCACTTGGATAACATCATCTAA

>Bt-RNase2/3B
ATGGTTCCAATACAGCAGGATTCTCGGCTTCGTCTCATTTTGCTGCTGGGGCTCTTGGGAATGGTGATCTCACTCCATGCCCAACCTGG
TACTTTAACCCGGGCTCTGTGGTTTGAGATTCAGCACATAAATATGGCCCACCCTCAATGCAATGCCGCAATGAGAGTGGTTAACCGTT
ACAGAATGGTATGTAAAAATAAAAATACTTTTCTCCACAGAACATTTGCTTATGTAGCTGGTATTTGTAACACCCCAAATGTAACCTGC
TCTAAACCAGGCAGGATGAACTGTCATAATAGCTCAGTCCAAGTGCCTATAACCTTCTGCAACCTCACAAGACCTGCATTGAATTACAC
AAACTGCCAATACCAACAGACAAGGGCATGGAGGATCTTCATCGTTGCCTGTGACAACAGATCACCTCGGGACAGTCCCAGGTACCCTG
TGGTTCCAGTTCACTTGGATAACATCATCTAA

>Bt-RNase2/3ps
CAGAGGATGAGATGGTTGGATGGCATCACTGACTCAATGGACATGAGTTTGAGCAAGCTCCTGGAGACAGTGAAGGGAAGCCTGGCGTG
CTGCAGTCCATGGGATCACAAAGAGTTGGACATGATTGAGCAACTGAACTGAACTGACCCTCAATGCAATGCTGCAATGTTACAGGATG
GTATGCAAAGGATGAAGTACTTTTCTCCACACAGTGCTTGCTCATGTAGCTCATATTTGTACCATCAAAAATGTAACCTTTCCTACATT
ACTCGGGATGAATTATCCTAACAGCTCGATTCAAATGCCTATAACCTCCTGAAACCTCATAGGAAATGCACTGTATTCATTATACAAAC
TGCCCAATGGTCAGACACAGACACAGAGTCTGTGGTCACCCTGTCCTGTGGTCACACAATCTTCACTGTTGCACGTGATCACAGGACAA
CACCATGTGGTTCTAGTTCACTTGATGAGATCATCTAG

>Bt-RNase4
ATGGCTCTCCAGAGGACCCAGGCCTTTCTTCTGCTCTTGCTGCTGACCCTGCTGGGGCTAGGGCTGGTACAGCCCTCCTATGGCCAGGA
TCGCATGTACCAACGATTCCTGAGGCAACATGTGGACCCTGATGAGACAGGAGGCAATGACAGCTACTGCAATTTGATGATGCAAAGAC
GGAAGATGACTTCACATCAGTGCAAGCGCTTCAACACTTTCATTCATGAAGATCTTTGGAACATCCGTAGTATCTGCAGCACCACCAAC
ATTCAGTGCAAGAATGGCCAGATGAACTGCCATGAGGGTGTAGTGAGGGTCACAGACTGCAGGGAGACAGGAAGCTCCAGGGCTCCCAA
CTGCAGATACCGGGCCAAGGCCAGCACCAGACGTGTTGTCATTGCCTGTGAGGGTAACCCGGAAGTGCCTGTGCACTTTGACAAGTAG

>Bt-RNase4ps
ATGGCTCTCCAGAGGACCCAGGCCTTTCTTCTGCTCTTGCTGCTGACCCTGCTGGGGCTAGGGCTGGTACAGCCCTCCTATGGCCAGGA
TCGCATGTACCAACGATTCCTGAGGCAACACGTGGACCCTGATGAGACAGGAGGCAATGACAGCTACTGCAACTTGATGATGCAAAGAC
GAAAGATGACTTCACATCAGTGCAAGCGCTTCAACACGTTCATTCATGAAGATCTTTGGAACATCCGTAGTATCTGCAGCACCACCAAC
ATTCAGTGCAAAAATGGCCGGATGAACTGCCATGAGGGTGTAGTGAGGGTCACAGACTGCAGGGAGACAGGAAGCTCCAGGGCTCCCAA
CTGCAGATACCGGGCCAAGGCTCCACCACAAGTCAGCCTTTGA

>Bt-Ang1
ATGGTCATGGTCCTGAGCCCCCTGTTTTTGGTCTTCATACTGGGTCTGGGTCTGACCCCAGTGGCCCCGGCTCAAGATGACTACAGATA
CATACACTTCCTGACCCAGCACTACGATGCCAAACCAAAGGGCCGGAATGACGAATATTGTTTTAACATGATGAAAAATCGACGCCTGA
CCAGACCTTGCAAAGACCGCAACACCTTTATTCATGGCAACAAGAATGACATTAAGGCCATCTGTGAGGACAGAAATGGACAGCCTTAC
AGAGGCGATCTCAGAATAAGCAAGTCTGAATTCCAGATCACCATCTGCAAGCATAAAGGAGGTTCCTCCCGGCCTCCATGCCGGTACGG
AGCCACAGAAGACTCCAGAGTCATTGTTGTCGGCTGTGAAAATGGCTTGCCCGTCCACTTTGATGAGTCCTTTATCACTCCACGCCACT
AG

>Bt-Ang2
ATGGTCATGGTCTTGAGCCCCCTGTTTTTGGTTTTCATGCTGGATCTGGGCCTGACCCCACAAACCCTGGCCCAAAATGATGCGTACAG
GGGATTTCTAAGGAAGCACTATGATCCTAGCCCAACAGGCCACGATGACAGATACTGTAATACCATGATGGAGAGACGAAACATGACTA
GACCCTGCAAAGACACCAACACCTTTATTCATGGCAACAGTGATGACATCAGGGCCGTCTGTGATGATAGGAATGGAGAACCTTACAGA
AACGGTCTTAGAAGAAGCAGGTCTCCCTTCCAGGTCACCACCTGCAGGCATAGAGGAGGGTCCCCCCGGCCTCCATGCCGGTATAGAGC
CTTCAGAGCAAACAGGGTCATTGTTATTCGCTGTCGAGATGGCTTTCCCATCCACCTTGAGGAGAACTTTATCCCTCCAAGACCGTAG

>BT-Ang3
ATGGTGATGGTCCTGAGCCCCCTGTTTTTGGTCTTCATGCTGGGACTGGGTCTGACACCACTGACCCTGGCTGAGGATGACCGCAGATA
CCGACACTTCCTGATTCAGCACTATGATCGTAGTCCAAAGGGCCGGGATAACAAATACTGTGAAACGATGATGGAGAAACGACACCTGA
CCAAACCCTGCAAAAGCATCAACACCTTTGTTCATGGCAACAAGAATGACATCAAGGACATCTGTAAAGATAAGAATGGAAAACCTTAC
AGAGGCAATCTCCGAATAAGCAAGTCTCCCTTCCAGGTCACCATTTGCAAGCATAAAGGAGGGTCCACCCGGCCTCCATGCCATTACAA
AGCCACCAAAGACTACAGAGTCATCGTTATCGGCTGTGAAAAGGGCTGGCCCGTCCACTTTGATGAGTCCTTTGTCCCTCCACACAAG

>Bt-RNase6
ATGGGGCCACATCTTCTGGGACGTTCTTCCCTCCTCTTATTGCTGCTGGGAATGTGGTGGTCAGTGCGTCCACTTTGTGCTGTGCCAAA
AGGTCTCACCAAGGCTCGCTGGTTTGAAATTCAGCACATACAGCCAAGGCTTCTCCAGTGCAACAAGGCAATGAGTGGTGTCAATAACT
ACACTCAGCACTGTAAACCTGAAAACACCTTTTTGCACAACGTCTTCCAGGATGTGACTGCCGTCTGTGATATGCCCAACATCATCTGT
AAGAATGGCCGGCACAACTGTCACCAGAGTCCAAAGCCTGTCAACCTGACTCAGTGCAATTTCATCGCGGGAAGGTATCCTGACTGCCG
CTACCACGACGACGCCCAATACAAGTTCTTCATTGTTGCCTGTGACCCCCCTCAGAAGACCGACCCCCCTTATCATTTGGTTCCTGTAC
ACTTAGATAAGGTTGTTTAA

>Bt-RNase7/8A
ATGGCTCCAGCCAGAGCAGGATTCTGCCCTCTGCTGCTGCTCCTGCTGTTGGGGCTGTGGGTGGCCGAGGACGCAGTCAGTGCCAGGCC
CGGGAACATGACCCCAGCTCAGTGGTTTGAAACTCAGCACGTGCAGCCCAGTCCTCAAGGCTGCAACGCTGCGATGCGCAAAATCAACA
AGTTCTCCAAACACTGCAAAGACCTCAACACCTTCCTGCATGAGTCCTTCTCCAGTGTGGCCACCACTTGTCAGACTCCCAACATAACC

TGCAAGAATCATGAGAATAACTGCCACCAAAGCCAGAAGCCTGTATCCCTGACCGAGTGTAAGCTCACCTCAAGGAGATACCCAGACTG
CAAGTACAAGGAGAAGAAACAGATCACTTCTTACATCGTGGCCTGTAAGCCTCCCAAGAAAGGGGACTCTGGGAAATTCAAGCTGGTTC
CAGTCCACTTGGAAAAAGTCCTTTAG

>Bt-RNase7/8B
ATGGTGCCAGCCAGAGCAGGATTTTGCCCTCTGCTGCTGCTCCTGCTGCTGGGGCTGTGGGTGGCCGAGGACGCAGTCAGTGCCAGGCC
TGGGAACATGACCCCAGCTCAGTGGTTTGAAACTCAGCACGTGCAGCCCAGTCCTCAAGGCTGCAGTGCCGCGATGCACAAAATCAACA
AGTTCTCCAAACGCTGCAAAGACCTCAAAACCTTCCTGCATGAGTCCTTCTCCAGTGTGACCACCAGTTGTCAGATGCCCAACATAGCC
TGCAAGAATGGCCGTAATAACTTCCACCAGAGCCAGATGCCTGTATCCCTGATTGAGTGTAAGCTCACCTCAGAGAAATACCCAGACTG
CAGGTACAATGAGGAGGAAAGGAATGCTTCTTATATTGTGGCCTGTGAGCCTCCTGAGAAAAAGTACTCTAAGAAATTCAAGCTGGTTC
CTGTCCATCTGGACAAGGTCCTTAATATTTTTATCTTTCCTTGGCCTTGGGTCCTGCCGTAA

>Bt-RNase9A
ATGGGGACTCTGATCAACGTGCAGCCCCTGTTTCTGCTCTTCCTGCTGCTGAAGCCACTGCAGTTTGTGAGGATAAATGATTCATATTT
ATCAGATGAACGACGAGAAGAATTAGAAGACTATATAGATGACTTACATGCTACAGGGCCTACCAAACCACCTACCAAGGAAGCATTCA
AAACCCATGTTATTATTGATTCTGAAATGCCGTTGACTGACGAAGATTATTGCAATCTAGAAATGAAGAGGAAACGTGTTCACAATAGG
CTTTATTGTGCTAAAGAGCATTTCTTCCTCCAAGCATCATATGAGGACATTGAAGAGATGTGTTATAACATATTTGTGAAATGTACAAA
TGGGATTAGGAAATGTCACAGGAGCCGGCAAATAATATCAGGAGTGCATTGTGTTTTAACAAGTGGGATCATGATGCCATTTTGTGAAT
ACACATCATCTTTCAAGGAGGGATGGGTCTTTATCACTTGTCAATGGGAAGATGATATTGGAGAAATTATCCCTGTGTCTGTAATTGAT
ATTTTGGCCATATAA

>Bt-RNase9B
ATGGGGACTCTGATCAACATGCTGCCCCTGTTTCTGCTCTTCCTGCTCCTGAAGCCACTGCAATTCGAGCTGATAATTGATGGTTCTCT
AACAGATAAAGAATTTGAAGAGCATTTAGATGGTACAAGTCCTACCAGAGCACCTACCAAGGAATTATTCAAAGCCCATGTCATTATTG
ATCGTAAAAGACCATTACATGATCCAACATACTGTAGTGATGAAATGAAGATAAAAAATATTCACCGTAGGCTTTATTGTGTTAAGGAA
CATTTCTTTCTTCAAGCATCGTACGAGGACATTGAAAAGATCTGTCATAACACGTTTATGTCCTGTGGCGAAGAAGTTGGGACTTGTTA
CTCGAGCCAGAAAAACATAAAAGCAGTGCATTGTGTTTTAACAAGTGGGATCAAGATGCCAGATTGTGAATACTCTTCTTCTTATGAGA
AGGGATGGGTCTTTATCACTTGTCGATGGCAAAATGATATCAGAGAAATTATCCCTGAATCTGTGGTTGGTATGTTGACATCCACCAGG
AAAGTACCTTCCCCCTACCGTTCTTAA

>Bt-RNase9ps
ATGGGGACTCTGATCAACATGAAGCCCCTATTTCTGCTCTTCCTGCTGCTGAAGCCACTGCAGTTCATGAGGATATATGATGAATCTTT
ATCAGACGAACAACGAGAAAGATTAGAAACCTAGATGACTTATATGGTACAGGGCTTATGAGACCACTAGCCTAAGAAACATTCAAATC
CCATGTCATTATTGATTCTGAAAGACCATAGTGATCCAGACCACTATAATGATGAAATGAGATGAAATGTGTTCACAATAGACTTTATT
GTGTTAAAGAGCATTTCTTCCTCCAAGCATCATATGAGGACATTCAACAGATCTGTGACAACACATTTGTGCAATGTAGCAGTGGGATT
AGGAAATGTCACAGGAGCTGGAATGTAATAGCCAGAGTGCATTGCATTTTAACAAGTGGGATCATGATGTCATTTTGTGAATACACTTC
ATCTTACAAGGAGTGATGGGTCTTTATCACTTGTCAATGGAGAAATGATGTTGGAGAAATTATTCCTGATTCTATAATTGATATTATTA
AATTGTAA

>Bt-RNase10
ATGAAGCTGACTCTGGTGCAGATCTTTTTCATGATGTTGCTGCTGCTTCTGGGCCTAGGGGTGGGCCTGGGTGTGGGACTCCAGATGGC
CGCAGCCGTCCTGGAGGAAAGTGATCAGCTACTGGATGAGTTTCTGTCCAGTGACTCACAGGACAAAGCTGAGGCTACTAAGGAGGGAC
TGGCCAGCCGAAGCACAGAAACCCTGCTGGTTAGCAACAAAGAAGTGGTGCAACCAGAAGACACCATCATCAGTGAAGATGAAGTTGGA
GGAGACAGGATGCTCAGAGCTGAGGTTCTTTTACACAGCAACAAAGACTATCTTAGATCTGACGTGATGGACAGGGAATGCAATGCCCT
GATGGCACTGAAGGTGAAGTCAAAAGACCACACTTGCATACCCCAGTACATATTTATCCATGAGGAACTAGATGCAGTCAAAGCTGTCT
GTAAGAGTCCTGCTGTTGCCTGTGATCTCAAGGGAGGCAAATGCCACAAAAGCCCCCGTCCTTTTGATTTGACATTCTGCAAGCTATCC
AAATCAGGCCAAGTGATTCCTCACTGTAATTATGTAACTTTCATTCTTGAAAAGTACATTCTTATGTCCTGTAGTGACATGAAAGTCCA
GATAACATCATGA

>Bt-RNase11
ATGGAGATCTTCTTTCTGCTGCTACTTGGCCTGGGGGTGATTTTTGCAGGAGTTTCAGAAAGTATAATGGAGATAATTAAAGAAGAATT
TTTAGAGAAAGAGATGAAATATGACATGGCAAAAAGTTACCAGGAAAAACACACCATTGAGGTATTAATAAATTTGACTGTGTCATATA
AAAATACCAGCCTCAGCATGTCCAAAGATGTGTCTTCCTCATTATTGACCTTTAGAAGATTACATTATAGCTTGCCCCCCAAATGCAAT
CCAGGTAATAACAAACATTACTGCAATGACATGACAGTCTGGAGAAAAGTTTCAGAAGCTAACGGGTCATTCAAGTTGAGCAATAACTT
CATCCATGGCTCCGTGGAAGTGGTCGATGGGGTACCCAAAGCCCCCAGCTGCAAGTGTGGACAGACTTCAGGCATAAGCTGCTCTGAGA
CTCCAGAACTGAGGACCACTACATGCCAGTTCACTGTGGGCAAACAATCCCCCAGTTGCCAACACCATGGTGTTACTTCATTAAAGAAA
ATTTTGGTGGTGCTGACAAGTCATTCTCTGATGAGCTGGTTAGTTAGTGTCTCTAACTTGTAA

>Bt-RNase12
ATGATACTAATGGTGATAATCTTCCTGGTGCTTCTATTCTGGGAAAATGAGCTGGATGAGGAAGCAATGGCGGCAACTTTAGAGCAGTT
GCATGTGGATTACCCTCAGAGTGACATTCCAATAAGGTACTGCAACCACATGATCATACAAAGAGTCATCAAGGAACCCAACAACACCT
GCAAAAAAGGGCATTTCTTCATCCATGAGAGGCCTCGAAATATCAACAGTGTTTGCAGTTCTCCCAGGAGGGTGACTTGCCAAAACCAT
GCCCCCAGTCTCTGCTTCCTGAGTGAAATCAAGTTCAAAATGACAGTCTGTAAGCTCATTGAAGGCACCAGATATCCTGCCTGCAACTA
CCATGTTTTCGCCACAGAGGGGTTTATTGTTGTCACTTGTGATGACATGGGGCCAGCTAAAATCCAGAGATACACTGAATAA

>Bt-RNase13
ATGGCACCACTTGTGGCCCAGCTCCTCTTCCTCCAGGTTGTTCTAGGGACAGCTTTGCTTGAAAACATCAAGACTCAGCTTGCCATCAA
GAACTTCCGTACCTTACACGTTGACTATCCCAAGGTTACCTACGCCCAAGGTTTCCAAGGTTACTGCAATGGTCTGATGTCCTATGTTC
GGGGCAGGCAAGAAAGCTGGTATTGCCCAAGGATCCATTATGTCTTACATGCCCCTTGGACAGTCATCTGGAAGTTCTGCAAATACAGT
GAGAGCTTCTGTGAGAATTACAATGAATACTGCACGCTCACCAAGGACTCCATCCCGCTCACAATTTGCTCCCTGTACCCCAGACAGCC

GCCGACCAGCTGCCGTTACAACAGCACCCTGACCAACCAAAGGCTCTATCTGCTCTGCTCTGAAAAGTATGATGGTGAACCCATAGATA
TCATTGGCCTCTACTAG

>Bt-RNase14
ATGAATCTGACGTGGACCCTTCTCCTCCTCCTCCTGCTGGAGCTAACTGTCTTCGCTTCAGGCCTGCCCTTCTCAAGACGGCACATAGA
TAACCCCAGGTCATGGGTTCCTGGGGGACAGCACCGATACTGCGATGTGATGATGAGGCGACGGTGGCTGATCCACAGGGGTAGATGCA
AGCAGATCAACACCTTCATTCACGAGGATCTGGCCACCATAGCAGATTTCTGCACAACTCCAGCTGTGCCCTGTACCAGCAGCGGCTCC
TTGCTGAGCTGCCACAACAGCTCTCACGACGTCAGCGTCACAGACTGCTTTGCCAAGGCAGGAACCCGGCCGCCCTACTGCCACTACCA
AAAGAAGGACTCCATCAGGCCCATCTGTGTGGGCTGTAAGAACGGGGCCCCTGCTCACCTGGATAGCTAG

>Bt-RNase15
ATGCTTCTGACATGGACTCTCCTCTTGTTCCTCCTAATGGACCAGCCTTCCTTCTCTGAGGCCCAGGACCTCCTCCATCACCAAACTGA
CAGGCCCAGAACAAAGATCTCTGCCAACCACAAATATTACTGTGACTTGATGATGAAGGCTCGAGGGCTGGCCACTGATCAGAGCTGTA
AGAAGAACAAGACCTTTGTCCACAGTGTGTCACCCGGCACTCGTGGCCTTTGCGAGGGTCCAGCTGTGACATGTAGGAAGATGTCTGAG
GTTTACAACTGCCACCTTATCATCTTCAAAGTCACTCAGTGTAATCTCTACCCAGAGGCTGTGCCCCCACACTGTTACTATGAAGGTGT
CACCTTCCAGATGGATGTTAGAATAGTCTGTGTGGGCAAACGTCCAATTCATTTGGATGAATAG

Horse (*Equus caballus*)

>Ec-RNase1
ATGGCTCAGGAGAAGTCTCTCATCCTGTTCCCACTGCTGGTCCTGGTGCTGCTGGTGCTGGGGTGTGTCCAGCTTTCCCTCGGCAAGGA
ATCACCCGCCATGAAGTTTGAGCGGCAGCACATGGACTCAGGCAGCACCTCCAGCAGTAACTCCACTTACTGCAACCAAATGATGAAGC
GACGGAATATGACACAGGGATGGTGCAAGCCCGTGAACACCTTTGTGCATGAGCCCCTGGCAGATGTCCAGGCCATCTGCCTCCAGAAA
AACATAACCTGCAAGAACGGGCAGTCCAACTGCTACCAGAGCAGCTCCAGCATGCACATTACAGACTGCCGCCTCACGAGCGGCTCCAA
GTACCCTAACTGTGCATACCGGACCAGCCAGAAAGAGAGACACATCATCGTGGCCTGTGAGGGGAACCCGTATGTGCCAGTCCACTTTG
ACGCTTCTGTGGAGGTCTCCACTTGA

>Ec-RNase2/3
ATGGTTCCAACACAGCGGGATTCTCGGCTTTGTCTCCTTCTGCTGCTGGGGATCCTGGGAATGGTGATCTCATACCATGCCACACCGGC
TGGTTTAACCAGGACTCAGTGGTTTGAAATTCAGCACATAAAAAATATGACCCACCGTCGATGCAATAATGAAATGCTGAGGGTTAACA
ATTATACGAAGAGGTGCAAAAATATAAACACTTTTCTCCACACGACGTTTGCTTTTGTAGCTAGTGTTTGTAACACCCCAAATGTAACC
TGCCCTACGACTCGCTACATGAATTGTCATAATAGCTCAGTGCAGGTAGACATAACAGACTGCAACCTCACCTCAGCACCACAGCCATA
TAAGAACTGCAACTATAGACAGACCAGCGCACGGAAGTACTTCATTGTTGCCTGTAACAACAGTCAACCAGGGGACAATGCCACCTATT
CAGTGGTTCCAGTTCACTTGGATTGGATCTCCTAA

>Ec-RNase2/3ps
ATGGTTCCAACACAGCGGGATTCTTGGCTTTGTCTCCTTCTGCTGCTGGGGATCCTGGGAATGGTGACCTCATTCCATGATCCACCGGC
TGGTTTAACCAGGGCTCAGTAGTTTGAAACTCAGCACATAAATATGACCCACCATCCCTGCGATAATGCAATGCAGGTGGTTAACCGTT
ATACGAAGAGGTGCAAAAATATAAACACTTTTCTCGACGCAACGTTTGCTTTTGTAGCTAACATTTGTAACACCACAAATGTAACCTGC
CCTACGACTGGCTACATGAATTGTCATAATAGCTCAGTTCAGGACGTAACAGACTGCGACCTCACCTCAGTACCACGGCCATATAGGAA
CTGCAGATGTGGACAGACCAGGGCACGGAAGTACTTCATTGTTGCCTGTAACAACAGTCAACCAGGGGACAATGCCACCTATTCAGTGG
TTCCAGTTCACTTGGATTGGATCTTCTAA

>Ec-RNase4
ATGGCTCTCCAGAGGACCTTGTCATTGCTTCTGCTCTTGCTGCTGACCATGCTGGGGCTGGTGCTGATACAGCCCTCCTACGGCCAGGA
TCGCATGTACCAACGATTCCTGCGGCAACACGTTGACCCTGAGGTAACAGTTGGCAGTGATGCCTACTGCAACTTGATGATGCAAAGAC
GGAAGATGACTTCCTACCAGTGCAAGCGCTTCAACACCTTCATCCATGAAGACATCTGGAACATTCGTAGTATCTGCAGCACCACCAGT
ATCCAGTGCAAGAACGGCAAGATGAACTGCCATGAGGGTGTAGTGAAGGTCACAGACTGCAGGGAGACAGGAAGTTCCAGGGCCCCCAA
CTGCAGATATCGGGCCATGACCAGCACTAGACGTGTTGTTGTTGCCTGCGAGGGTAACCCGGAGGTACCTGTGCACTTTGATAGATAG

>Ec-RNase4ps
GTGGCTCTCCAGAGGACCCTGTCATTGCTTCTGCTCTTGCTGCTGACCATGCTGGGTCTGTTGCTGGTACAACCCTCCTATGGCCAGGA
TCACATGTACCAACGATTCCTGTGGCAACACATAGACCCTGAGGTGACAGGTGGCAGTGATACCTACTGCAACTTGATGATGCAAAGAT
GGAAGAGGACTCATATCAGTGCAAGGGCTGCAACGCCTTCATCCGTGAAGACTTCTGGGACATTCGTAGTATCTGCAGCACTCCCGGCA
TCCAGTGCAAGAACGGAGGATGAACTGCCATGAGGGTGTAGTGAAGGTCCCAGCCTGCAGGGAGACAGGAAGCTCCAGGGCCCCCGACT
GCAGATATCAGGTCTTGGCCAGCGCTAGGCGTGTTGTCATTGCCTGTGAGGGAAACCCGGAGGTGCTTGTGCACTTTGATAGACAG

>Ec-RNase5
ATGGCGATGAGCCTGTGCCCCCTGTTGTTGGTCTTCGTGCTGGGTCTGGGTCTGACCCCACCATCCCTGGCTCAGGATGATTCCAGGTA
CAGACAGTTCCTGACCAAGCACTATGATGCCAATCCAAGGGGCCGGAATGACAGATACTGTGAAAGCATGATGGTGAGACGACACCTGA
CCACACCCTGCAAAGACACCAACACTTTTATTCATGGCAGCAAGAGCAGCATCAAGGCCATCTGTGGAAATAAGAATGGAAACCCTTAC
GGAGAAACTTTAAGAATAAGCAAGACTCGTTTCCAGGTCACCACTTGCAAGCATGCAGGAGGGTCCCCCCGGCCTCCATGCCGATACAG
AGCCACACCAGGGTTCAGAAGCATTGTCATTGCCTGTGAAAACGGCTTGCCTGTCCACTTTGATGAGTCCTTTTTCCGTCCATAA

>Ec-RNase6
ATGGTGCTAAGTCTCCTGGGACGCTGTCCTCTGTTCCTATTGCTGCTGGGACTATGGGTGCTGGTGCATCCACTTTGTGCTTGGCCTAA
GTCTCTCACCCAGGCTCGCTGGTTTGAAATTCAGCATATACAGCCAATCCCTCTCCAATGCAACAAGGCAATGAATGGTGTCAATAATT
ATACCAAGCACTGTAAACCTGAAAACACCTTTCTACATGACTCCTTCCAGAACGTGGCTTCTGTCTGCAATTTGCGCAGCATCATCTGC
AAGAATGGCCAGAAAAACTGCCACCAGAGTTCCAGGCCTGTTCACATGACCCAGTGCAGTCTCACATCAGGGACGTATCCTAACTGCCG
CTACAGGGATGCTGCCCCATACAAGTTCTTCATTGTTGCCTGTGAACCCCCTCAGCAGGGTGACCCTCCATATCACTTGGTTCCTGTAC
ACTTAGATAGTATTGTTTAA

>Ec-RNase7/8
ATGGCAGCTGCCAGAGCCGGATTCTGCCCCCTGCTGCTGCTTCTGCTGCTAGGGCTCTGGGTGGTCAAGGTCTCAGTCAGTGCCAAGCC
CAAGCACATGACCCCAGCTCAGTGGTTTGAAACTCAGCATGTGCAGCCCAAGCCCCAGGGATGCAACACGGCGATGGGCAACGTCAACA
AGTACACAAAACGCTGCAAAGACCTCAACACCTTCCTGCATGAATCCTTCTCCAGTGTGGCCACCACCTGTCAGACCCCCAGCATAGCC
TGCAAGAACGGCCATAAGAACTGCCACCAGAGCCAGAAGTCCGTGTCCCTGACCATGTGTGACCTCACCTCTGGGAGGTACCCAGACTG
CAGGTACAAAGAGAAGCAACTGGATGCGTTCTTCATTGTGGCCTGTGACCCTCCCCAGAAAGGGGACTCTGGGCAATTCCAGCTGGTTC
CTGTGCACTTGGACAAAGTCTTGCAGATTTCCAGCCTTCCTCACTCTTGGGTTGTGCCTTAA

>Ec-RNase7/8ps
ATGGCACCAACCAGAGCAGGGCTCTGCTCCCTGCTGCTGCTCCTGCTGCTGGGGCTCTGGGTGGCCAAGGTCCCAGTCAGTGCCAAGCC
CAAGCACATGACCCCAGCTCAGTGGTTTGAAACTCAGCACGTGCAGCCCAAGCCTCAGGAATGCAACACGGCAATGGGCAACGTCAACA
AGTACACAAAACGCTGCAAAGACCTCAACACCTTCCTGCATGAATCCTTCTCCAGTGTGGCCACCACCTGTCAGACCCCCAGCATAGCC
TGCAAGAACGGCCATAAGAACTGCCACTAGAGCCAGAAGCCCGTGTCCCTGACCATGTGTGACCTTACCTTGGGAAGATACCCAGACTG
CAGGTCCAAAGAGAAGCAATTGGACACATTCTTCCTTGTGGCCTGTGACCCTCTCCAACAGAGGGACAACCTGAGGTACCAGCTGCTTC
TTGTGCACTTGGATAATGTTGTCTAAGGCCAGGTGCTCACCCCACCCCAAGCTCTGCAG

>Ec-RNase9
ATGTGGATTCTGATCACCACGCAGCTCCTGCCTCTGCTGCTCCTGATGCTGCAGCCACTGCAGTTTGAAAAGCTTTTTAAATATTCTGA
TTTATCAGATGTTGACTCAGAAGAATTTGAAGATTATTTGGAGGAATTATACAGTACAGGGCCTACCAAAGCACCTACCAAAAAAACTT
TCCAAAATCAGGTCCTTGCTGATCCTGACAGACCATTATCTGATCCACATTACTGCAATGATGAAGTTAGGATGAAAAATGTTCACAAC
AGGTTATATTGTAAGAAAGAACATTTCTTCCTCCAAGCTACATATGAGGAGGTGCAAAAGATATGTCGCCACCTGTTTGTACCATGTAA
GAACGGAGTTAAGAAATGTCATAGGAGCAAGAAACTAATAGAAGGAGTGTATTGTAGTTTAACATCAGGAACCATGATAGTAGACTGTA
TCTATGAATCTTTTTACAAGCGTGGATTTGCCCTTATCACGTGTCGATGGCAAAATGATATCCAAGAAATTATCCCTGATCATGTAGAT
GATCTTCAGGAATTATATAGCTAG

>Ec-RNase10
ATGAAGCTGACTTTGGTGCAGATCTTTTTCATGATGTTGCTGCTGTTGCTGGGCCTGGGGATGGGCCTGGGGTTGGGGCTTCAGATGGC
TGCAGCAGTCCTGGAGGATAGTGATCAATCACTGAATGACTTTTGGTCCAGTGACTCACAGGAGAAGGCCGAGACCACTAAGGAGGGAG
ATGGCACCCGAACCACAGAAACCCTGCTGCTTAGCAACAAAGGAGTGGTTCAACCTGTGTGGCCAGAAGAGACTATCCTCGCTGAGGAT
GAAGTTGGAGGAAACAAGATGCTCAGAGCTGATGCTCTCTTTCAGAGTGACAAAGACTATCTTAGGCTTGACCTGATGAACAGGGAATG
CAATTCCCTAATGGCACATAAGGTGAAGAAGCGCAACCACACATGTATACCTGAGTACACATTCATCCATGAGGAACTAGATACAGTCA
AAGCTGTCTGTAAGAATCCTGTTGTTGCCTGTGATCTCAAGGGAGCCAAATGTCACAAAAGCTCCCGTCCTTTTGATTTGACATTCTGC
AAGTTATCCAAACCAGGCCAAGTCACTCCTCACTGCAATTACCTAACTTTCATTTTTGAAAAGTTCATTATTATATCCTGTAATGACAT
GAAGGTCAAGATAACATGA

>Ec-RNase11
ATGGAGACTTTTTCTCTGCTGCTACTCGGCCTGGGGTTGGTCCTTGCAGGAGCTTCAGAAAGCATAATGAAGATAATTAAAGAAGAACT
TTCAGAGGAAAAGATGAAATACGGCATGACAAAAAGTGACCAAGGAAAACAGACCGTTGAGCTATTAATGGACTTGACTCTGTTATATA
GAAATACCAGCTTCGGCATGTCCAAGGATATTATGTCTTCCTCATTACTGACATTCAGAAGATTACATTATAGCTTCCTTAAGGGAAAC
AGCCCAGGTAATGACAAAGAGTATTGCAATGACATGACGGTCTGGAGAAAAGTTTCAGAAGCTAATGAGTCATGCAAGTTCAGCAACAA
CTTCATCCACGGCTCCATAGAAGTGATCCACAGCACCCCCAAGGCCCCCTGCTGCAAATGTGGACAGAATGCTGTCATAAGCTGCTCTG
AGAACGCAGAACTGGAGACTACTATGTACCAGCTCACTAGAGGCAAACAATTCCCCAGGTGCCAATACCACAGTGTTACCTCATTAAAG
AAAATATTGGCAGTGCTGACAGGTCATTCTCTGATGAGCTGGTTAGTTAGTGGCTCTAAGTTGTAA

>Ec-RNase12
ATGATACTAATGGTGATAATTTTCCTGCTGCTTCTGTTCTGGGAAAATGAGCTGGACGACGAAGGAGTGGTGTCAACCTTAGAGCACTT
GCATGTGGACTACCCTCAGAGTGACATTCCTGTAAGGTACTGTAACTCCATGATCTTACAAAGAAGCATCAAGGGACCTGACAACGCCT
GCAGAAAGGAGCACGTCTTCATCCATGAGAGGCCTCGAAATATCAATAGGATTTGCTCTTCTCCCAATAAGATGGCTTGCCAAAACAAT
TCCACCATTTTATGCTTCCAGAGCAAGACAAAGTTTAAAATGACGGTCTGTAAGCTCATTGAAGGCACCAGATATCCTGCCTGCAGGTA
CCGCGTTTCCCCCACAGAGGGGTTTATTCTTGTCACTTGTGATGACATGGGGCCAGTTAATCTCCAGAGATATGTTGAATAA

>Ec-RNase14ps
GTCTGCTCAACTAAGGAGCAAGAATCACTTTAGGGTTTGGAAATAACTGTGTATGAGAGGCCCATAAAGACGAGAATGGGTGGTAGGCA
CTTCCTCTATTGACAAGTACTTTTCTCTTCACCTCTTGCCCAGAGAAAAGAAGGGGTGTGATGATGAGGCGAAGGTGGCTGATCCATAA
GGATAAATGCAAGCTGATCAATACATTCATTCAGGAGGATTTGGCCACCATAGCAGATTTCTGCAGCATTCCATCAGTGCCCTGCACCG
ACAGTTGCTCCATGCACAACTGCCACAACACGACTCATGATGTCAACGTCACTGACTGCTCTGCCAGTACAGGGCCCTGACCTTCTTAC
TGCCACTACCATAACAAGGACTCCACCAGGCCCATCTGGGTGGGCTGTGAGGGAGGGCACCTGTTCACCTGGATGGCTAG

>Ec-RNase15ps
ATGCTTCTGGAATGACCCTCCTCTTGCTCCTACTAATGGACCAGCCTGGCTTCTCTGAGACCCGTGACTTCCTCCATCCTCATGTTGAC
AGGCCCAAAGCAAAGGTCTCTGGCAGGTACAAAGAATGCTGTGACATGATAATGAAGATTCCAGGGTTGGCCACTGAGAAGAGCTATAA
GAAGAGTAAGACCTTTATCCATAGTGTGTTTCCCAACACAGGTGGCCTCTGTGAAGGTCCAGCTGTGAGGTGTGTGAGGAAGCCTGGCC
TTTATAGCTGCCACCTCACCACCTTCAAAATCAGTCAACGTAATCTCTAACAAAAGTCTTGTCCTCACACTGTTACTACCATCACACTA
CCTTCTGGAGGGATGTGAGGGTGGTCTGTGTGTGGAAAAACCCAATTTGCTTGGATGAATAG

Dog (*Canis familaris*)

>Cf-RNase1
ATGGCTCAGGAGAAGTTCTTGGTGCTGTTGCCACTAGTGGTCCTGGCGCTGCTGGGGCTGGCCTGCGTGCAGCCTTCTCTGGCCAGGGA
GTCGAAGGCCATGAAGTTCCAACGGCAGCACATGGACTCGCACCCCGCCGCCATCAGCGCCAGCTACTGCAACCTGATGATGAAGCGCC

GCAACATGACGGATGGGTGGTGCAAGCCCGTGAACACCTTCGTGCACGAGCCTCTGGCAGACGTCCAGGCCGTCTGCTCCCAGAAGGAT
GTGCTCTGCAAGAACGGGCAGTCCAACTGTCACCAGAGCCGCTCCCAGATGAACATCACCGACTGCCGCCTGAAGAACGGCTCCAAGTT
CCCCAAGTGCGTGTACACGACCACGCAGAAGGAGCAGTACATCGTCGTGGCCTGTGAGGGGAACCCCCACGTGCCAGTGCACTTTGATG
CGTGTCTGTAG

>Cf-RNase4
ATGGCCCCGCCGAGGACCCGCGCTCTGCTCTTGCTGCTGACCCTGCTGGGGCTGGGGCTGGTGCAGCCCGCCGACGGCCAGGACCGCAT
GTATCAGCGATTCCTGCGGCAGCACGTGGACCCTGAGGGGAAGGGTGGCAACGACACCTACTGCAACGTCATGATGCAAAGGCGGAAGA
TGACCACGCGCCAGTGCAAGCGCTTCAACACCTTCGTCCATGAAGACATCTGGAACATTCGTAGTATCTGTAGCACCACCAACATCCAG
TGTAAGAATGGCAAGATGAACTGCCATGAGGGCGTAGTGAAGGTCACGGACTGCAGGGAGACGGGCAGCTCCCGGGCCCCCAACTGCAG
GTATCGGGCGTCGGCGAGCACTCGGCACGTGGTCATTGCCTGCGAGGGGGACCCGGAGCTCCCTGTGCACTTTGACAGATAG

>Cf-RNase5ps
GTGGTGATGGGCCTGGGCCCCTGCTGGTCTTCTTGCTGCTGGGGCGGCTGTGACCCCGGGGAGCCGGGCCCAGGAGGACTCCAGGTACT
GACACGTCCTGCCCGGGCACCATGGTGCCAAGCCGCGGGCCGGGATGACAGGTAGCGTGAGAGCAGCGGGCGAGTTGAGGCCGGCCGCA
CCTGCCGGGACACCCCGCAAGGACACCAACACCTTTATTCACGGCAACAGGGCCGGCGTCAGGCCGCGTGCGGAAATAAGAATGGGACC
CTTAGGGACAAGCTTTAAGACTGAGCAAGTCTCCCTTCCAGGTCACCACTTGCAGGCACGTAGGCGGGTCCCCCGGCCCCCGGCGGGCA
CAGAGCGACCCGGACTCCGGCACGTCGTCGTCCGAGTCCCTTCTGCAGCGCAGCCAGAGGCCCCAGCGGCTCTGTTCTCCTCCCCCGGC
TCCCTCCAGGCCCCGG

>Cf-RNase6
ATGATGCTGCATCTCCCAGGGCCGTGGCCGCTGCTGCTGCTGCTGCTGCTGGGATCATGGGGACCAGTGCCTCCAGCTGCTGCTCCGCC
TCCAGGCGTCACTGCCGTTCGGTGGTTTATCACCGAGCACGTCAGAGCGGGTCCGGTGAGATGCAGCACTGAAATGCCTCAGATCAATT
ATCGCCCTAACATATGTAAGGGGCAAAACACCTTTCTGCAAGAGTCCTTCCAAAACGTGGTTGCTACCTGTCAACAGCCCAACAGGACC
TGTAAGAATGGCCTGGGAAACTGCCACAAGAGCGCAGGGAGGGTGAACATGACCTACTGCCTTCTCACTGGGCGCCGGCCCCAGTGCAC
CTACCGTACTACGTACCAGAACCAGTTCTACATTGTTGCCTGTAACAACTCGCAGCCAGGCTACCCTCCCAATCTTCTGCTTCCCGTCC
ACTTAGACGACACCGTCCCGCTGGGTCCTACACACTTCGGTTAA

>Cf-RNase6ps
ATGATGCTGTGTCCCCCCGGGGCCGTGGCCTCTGCTCCTGCTGCTGCTGGGATCATGGGGGCCGGTGCCTCCAGCTGCTGCTCGGCCTC
CAGGCTTCACCGCCCTTCAGTGGTTTAACACCCAGCACTTAAGAGCGAATCAGGTGCAATGCAGAACTGAAATGCCTCGGATCAATAAT
CTGCTTCCCAGATATAAGGGGAAAAACACCTTTCTGGTTGAGTCCTCGAAAACGTGCTCGCTACCTGTCAACAGCCCAACATGAACTGT
ACCACCAGTGGGGAAAACTGCCACAAGAGCGCAGGGAGGGTGAACATGACCTACTGCCTTCTCACTGGGCGCCGGCCCCAGTGCACCTA
CCGTACTACGTACCAGAACCAGTTCTACATTGTTGCCTGTAACAACTCGCAGCCAGGCTACCCTCCCAATCTTCTGCTTCCCGTCCACT
TAGACGACACCGTCCCGCTGGGTCCTACACACTTCGGTTAA

>Cf-RNase9
ATGTGGACTCTGCTCACCATGCAGCCCCTGGCTCTGCTGCTCCTGCTGATGATGCAGCCACTGCAATTTATAATTCTGAAGAACTTTGG
TGATTTCACAGATGAAAAGCTAGAAGAGTTTGAAGATTACTTGGGGGACATATACAGCCCAGGACCTGCCAAACCTCCTACCAAAGATA
CTTTCCTAAGGCGTATTATTATCGATCCTGGAAGACCACTAACTGATTCAGCCTACTGTACTGAAGAAATCAAGATGAAAAACGTTCAC
AACAATTTCAGTTGTGTCAGAGAACATTTCTTCCTCCAAATAGCATATGAGGACGTGCAAAAGACCTGTAAAAACTTACATGTGCCTTG
TAAGAATGGAGTTAAGAAATGTCACAGGAGCAAGGAAGTAATAGAAGGAGTCTATTGTAATTTAACACGAGGAGCTAAAATGACAGACT
GTGAATATGACTCTTTTTATAGGCAGGGCTATGTCCTTATTACTTGTCGATGGCAAAATAATATTCAGGAAATTGTTCCTGTTTACGTA
GATGATATTATGGCTCTGGATAATGATGTGAAAGCCAACATCTACCACAATGCAAAAAAGTAG

>Cf-RNase10
ATGAAGCTGACTGTGGTGCAGATCCTCTTTATGATGCTGCTGCTCCTGCTGGGTCTGGGAATGGGCCTGGGCTTGGGGCTTCGGATGGC
TGCAGCCGTCCTGGAGGACAGCGATCAGTCCCTGATGGAGCTTTGGTCTAGCGACTCGGAGGACACGGCCGAGGCCACTAAAGGAGAGG
GCAGCCGGACTACGGAGAACTTGGTGCTTAGCAGCAAAGGCGTGCTGCCCCCCGGCTGGCCCGAAGACCCCATCCTCGGTGAAGACGAA
GTGGCGGGAAACAAGGTGGGAGGGGAGCCTCTCCTTCCCAGCAACAAAGCCTACCTTCGGTCTGACCTGCTGGCCAGGGAATGCAACAC
CCTGATGGCGCCCAAGGTGAAGGGTCACAACCGCACGTGCATAAGCCAGTACACGTTCATCCATGAGGACCTTGACACCGTCCAGGCCG
TCTGCAACAGTCCCGTCGTGGCCTGTCAGCTCAAGGGAGGCAAATGCCACAGAAGCTCCCGTCCTTTTGACTTGACCTTCTGTAGGTTG
TCCAAACCGGGCCAGGTCACTCCTCACTGCCATTACGTCACTTTCATTTTTGAAAAGTATATTATCATATCCTGCAATGACATGAAGGT
CCAGGTAGTGTCTGGACAATGA

>Cf-RNase11ps
CTGGACACCCGGGCCCTGCTGCCCCTCGGCCTGGCTCGGTGTTGCAGGGGTCGCGAGTGCCTCCCCAACATAGTCAAGGAAGGACTCTC
AGGGGCAGAGGGGCGACGTGACTTGGCAAGTGGTGTCCGGGGCGCAGACTCGTGGGTCACTAACGAACGTGACTCTTATGTAGACGTAC
CAGCCTCGGGGGGACCGACGATGTCCCGTCTTCCCCATCACGGGTTCTCGGGAGGTGACACGGTGGCTGCCCGGGGGCACGGCCCAGGT
AGCGGTGGCGGCCCTCGCAAGGACACGGTGGCCGCAGACGAGCTCCGGGAGCCGGTGGTCACGCGAGCTGAGCTGGGACTTAGCGACTC
GAGGCCCCACGGCTCCGGCTGCGGGCGGGGGTTGGCACAGCCGCACCTGGAGCCCCCGCGGCCAGCTCAGCCTCTGCAGGTGACCCTCC
GTGCCTACGCCGCGGTGCCCGGTGCAGGACCTGGCCGCGCACACAGGCGGGTCCCTGGCAGGCTGGGCAGTGAGCCGCCTTAGTAGTGA
CCCCCCGGCGCCCATGTCCCCCAGCGCCCATGCCCCCCGGTGCCCCTGGTGCCCCCCGATGCCCCTAGAACCCCAGTGCAGGACCTGGC
CA

>Cf-RNase12
ATGATCCTAATGGTGATCATTTTCCTGCTGCTTCTGTTCTGGGAGAATGAGCTGCATGAGGAAAGAGAGGTGCCAACCCTGGAGCACTT
GCACGTGGACTACCCTCAGAGCGACATTCCTGTAAGGTACTGCAACCGGATGGTCTTACAAAGAGTCATCAGGGGACCTGACAACACCT
GCAAGAAGGAACACGTTTTCATCCACGAGAGGCCTCGAGAGATCAACAGGGTGTGCACCTCTCCCAAGAAGAGGGTTTGTCAGAACCAT
TCTTCCATCTTATGTTTCCAGAGTGTGACAAAGTTCAAAATGACAGCTTGTCAGCTCATCGAAGGCACCAGATATCCCGCCTGCAGGTA
CCACATTTCCCCCATAATGGGGTTCGCTGTTGTCACTTGTGATCACATGGGGCCAGTGACTCTGCAGAGGTATGTTGAGTAA

>Cf-RNase13
ATGGCACACGCTGTGGCCCGGCTCCTGTTTCTCCAGCTCCTTCTAGGGCCAACTCTGGTTGTGGACATCAGGGTGCAGATTGCCACCAA
GAAATTCCGCATGTTACACATCGAGTATCCCAGGGTTAACTACCCAGAGGGCTTCCAGGGCTATTGTAACGGTCTCATGGGCTACGTGC
GGGGCAAAAAGCAGAGCTGGTTTTGCCCCCAAACCCATTATATGGTGCATGCCCCCTGGAGAGAGATCCAGAAGTTCTGCAGGTACAGC
GACAGCTTCTGCGAGAATTACAATGAATACTGTACGCTCACAGAGGACTCCTTCCCTGTCACCATCTGCTCTCTGGCCTCTCAACAGCC
GCCCACGAGCTGCAACTACAACGACACCCTAACCAACCAAAGGCTCTATCTGCTCTGTTCTGGAAAGCGTGATGCTGAACCGATAGGTA
TCATTGGCCTCTACTAG

Giant panda (*Ailuropoda melanoleuca*)

>Am-RNase1
ATGGCTCAGGAGAAGTTCTTCATCCTGTTCCCACTACTGGTCCTGGTGCTGCTGGCGCTGGGCTGTGTCCAGCCGTCTCTGGGCAAGGA
GTCCCGGGCCAAGAAGTTCCAGCGGCAGCACATGGACTCGGACACCTCCATCGTCGGCTCCAGCTACTGCAACCAAATGATGAAGCGGC
GGAACATGACGGCCGGGTGGTGCAAGCCGGTGAACACCTTTGTCCACGAGCCTCTGCCCGACGTCCAGGCCGTCTGCCTCCAGAAAAAT
GTCACCTGCAAGAACGGGCAGCCCGACTGCCACCAGAGCAGCTCCAGGATGCACATCACCGACTGCCGCCTGAAGCGTGGCTCCAAGTA
CCCGAAATGCGAGTATCAGACGGAGCAGAAGGAGAAATCCATCATCGTGGCCTGTGGGGGGAACCCGTATGTGCCGGTCCACTTCGACG
CTTCCGTGTAG

>Am-RNase4
ATGGCTCTCCACAGGACCCGTTCACTGCTTCTGCTCTTGCTGCTGACCCTGCTGGGGCTGGGGCTGGTGCAGCCCTCCTATGGCCAGGA
TCGCATGTATCAACGATTCCTACGGCAGCATGTGGACCCTGAGGGGACAGGTGGCAATGACACGTACTGCAACTTGATGATGCAAAGAC
GGAAGATGACCACACATCAGTGCAAGCGTTTCAACACCTTCGTCCATGAAGACATCTGGAGCATTCGTAGTATCTGCAGCACCACCAAT
ATCCAGTGCAAGAATGGCAAGATGAACTGCCATGAGGGTGTCGTGAAGGTCACAGACTGCAGGGAGACAGGAAGCTCCAGTGCCCCCAA
CTGCAGATATCGGGCGTCAGCGAGCACTAGGCATGTGGTCATTGCCTGTGAGGGTGACCCACAGTTGCCTGTGCACTTTGACAGATAG

>Am-RNase5
ATGGTGAAGGGCCTGGGCCCCCTGTTGTTGGTCTTCATGCTGGGTCTGGGTCTGACCCCACCAGCCCGGGCTCAGGATGACTCCAGGTA
CAAACACTTCCTGACCCAGCACTCTGATGCCAAACCAAGGGGCCGGAATGACAGATACTGTGAAAGCATGATGGAGAGACGAGGCCTGA
CCACACCCTGCAAAGACACCAACACCTTTATTCATGGCAACAAGGGCAGCATCAAGGCCATCTGTGGAAATAAGAATGGAAACCCTTAC
GGAGAAGCTTTAAGACTAAGCAAGTCTCCTTTCCAGGTCACCACTTGCAGGCATGTAGGAGGGTCCCCGCGGCCTCCGTGCCGGTACAG
AGCTACCCCAGGGTTCAGACACATCGTTGTTGCCTGTGAACATGGCTTGCCTGTCCACTTTGATGAGTCCTTTTTCCGTCTGTAA

>Am-RNase6A
ATGATGCTGGATCTTCTGGGACCCTTTCCTCTCCTCCTGTTGCTGCTGGGATCATGGGGGCCGGTGCACCCACTTGGTGCTTCGGCTCA
ACCCCCCACCAGGGCTCAATGGTTTCAAATTCAGCATATAAGTGGAGGTCCTGTCCAATGCAACAATGCAATGCGTCGTGTCAATAATA
ATAATCAGCGCTGTAAACCTCAAAACACCTTTTTGCACACCACCTTCCAGAACGTGGCTGCTACCTGTAGTTTGCCCACCAGGCCCTGC
AGGAATGGTCAGAATAACTGCCACCGGAGTGCACGTCGTATCGGCATGACCTACTGCAATCTCACTGGAGGGAGATTTCCCAACTGCCG
CTACAGCACCACTCCCCAGAACCAGTTCTACACTGTTGCCTGCAACCCTCGTCAGCCGGGCGACCCTCCCTATCGTCTGGTTCCTGTAC
ATTTGGATTAA

>Am-RNase6B
ATGATGCTGGATCTTCTGGGACCCTTTCCTGTCCTCCTGATGCTGCTGGGATCATGGGAGCCGGTGCATCCACTGTGTCACTGCGGTCA
ACCCTCTGCCAGCGTTCAAACGTTTATACTTCGGCATCTACGAGCAGGTCCTCTCCAATGCAACGATGCAATCTCCGCCACTGTTCGAA
CGTTTCAAATTAAGCATCTAAGAGAACATCGTGTCCAATGCAACAGGGAAATGCGTCGTGTCAACATTCTTGATCAGCGATGTAAAGAT
CAAAACACATTTCTGTATGACTCTCTCCACAATGTGGCTGCCACCTGTCTTTTGCCCAGCATGAGGTGCAGGAATAGAACTCAGATGAA
CTGTCACCAGAGTGAAAGTCCTATTAACATGACCTACTGCAATCTCACTGGAGGGAGATTTCCCAACTGCCGCTACAGCACCGCTCTCC
AGTACAGGTTTTACGTTGTTGCCTGTAACCCTCGTCTGCCACGTGACCCTCCTTATCCTCTGGTTCCTGTCCACTTAGATTTAACCATT
CCTGTGGTTTCCTTACACTTAGGTTAA

>Am-RNase6C
ATGATGCTGGATCGTCTGGGACCCTTTCCTGTCCTCCTGTTGCTGCTGGGATCATGGGCGGCAGTGCTTCCCCCTGGTGCTTGGACTCA
ACCCCCCACCAGGGTTCAAAAATTTATAATTCAGCATTTAAGTGCAGGTCCTGTCCAATGCAACATTGCAATGCGTGCTGTTAATAATC
TTAATCAGACCTGTAAGGATCAAAACACGTTTCTGCACGACAACTTCCCAAATGTGACTGTTACCTGTGGTTGGCCCAATAGGAATTGC
AGGAATGGCCAGAATAACTGCCACCAGAGTACATATGCTGTTGGCAAGACTCACTGCAGTCTCACTGGAGGGAGATTCCCCAACTGCAG
CTACAGGACTACTCCCCAGCACGGGTTGTACATTGTTGCTTGTGACCCGCGTCAGCGAGGCTACCCTCCCAATTGTCTGTTTCCTGTGC
ACTTAGACTAA

>Am-RNase6D
ATGATGCTGGATCTTCTGGGACCCTTTCCTGTCCTCCTGATGCTGCTGGGATCATGGGAGCCGGTGCATCCACTGTGTCACTGCGGTCA
ACCCTCTGCCAGCGTTCAAACGTTTATACTTCGGCATCTACGAGCAGGTCCTCTCCAATGCAACGATGCAATGCGTCTTGTCAATCTTT
CTAATCGGACTTGTATGCGTCAAAACACCTTCCTGCATGACACCTTCCGAAATGTGGCTGATACCTGTGGTTGGCCCAATAGGATCTGC
AGGAATGGCCAGGATAAGTGTCACCTGAGTGCAAACCGTATTAACATGACTCACTGCTATCACACTGGAGGGAGATATCCCAACTGCAG
CTACAGAACTATTCCCCGGCAGGGGTTGTACACTGTTGCCTGTGACCCTCGTCAGCCAGGCTTCGATGCCAATTGTCTCTTTCCTGTAG
AGTTAGACTAA

>Am-RNase6E
ATGATGCTGGATCTTCTGGGACCCATTCCTGTCCTCCTGTTGCTGCTGGGATCATGGGCGCCGGTGCATCCATTGGGTGACTGGGCTCA
ACCTTCCAGCAGCGTTCAAACGTTTATACTTCGGCATCTAAGAGCAGGTCCTGTCCAATGCAACACGGAAACGCCTCCTGTCATTCATC
TTGAACAGTGTCGGAAGCCTGAAAACACCTTTCTGCATGACTCCTTCCACAATGTGTCAGATACCTGGCTTTTGCCCAACAGGACCTGC
AGTAGAGGCCAGAAGAACGGCCAGCAGGGTGCAAATCTTAGTGACATGACTCACTGCAATCTCACTGGAGGGAGATACCCCCACTGCCG

CTACAACACTGCCCCCCAGAACCGGTTCTACATTGTTGCCATAACGACTCTCAGCCAGGCTACGCTCCCCATCTCCTGCTTCCTGTACA
CTTAG

>Am-RNase7/8
ATGGCAGCAGTCAGCATGGGCTCCTGCCCTCTGCTGCTGCCCCTGCTGCTGGGGCTGCGAGTGGCTGAGGTCCCAGTCAGGGCCAAGCC
CAGCAACATGACCTCGGCTCAGTGGTTTGAAATTCAGCACGTGCAGCCCAAGCCTCAGAGATGCGATGCGGCGATGGGCAAGGTCAACA
AGTACACAAAACACTGCAAACCCTTCAACACTTTCCTGCATCAGTCCTTCTCCAGTGTGGCTGCCACCTGTCAGACCCCCACAGTAGCC
TGCAAGAGGGGCCGAAGGAACTGCCACCAGAGCAAAAAGCCTGTGTCCCTGAGCACATGTGAGCTCACCTCAAGGAAATACCCAGGCTG
CAAGTACAAAGAGAAGCGACAGGTCGCTTCTTACATTGTGGCCTGCGACCCTCCTGAGAAAGGGGACTCTAGGAAATTCAAGCTGGTTC
CTGTGCACGTGGACAAAACCCTTTAG

>Am-RNase7/8ps
AGGGCGGCAGCCAGAGCGGGCTCCTGCCCTATGCGGCAGCCCCTGCTGCTGGGGCTGTGGGTGGCTGAGGTCCCAGTCAGGGCCAAGCC
CAGTCACATGACCTCAGCTCAGGGGTTTGAGACTCAGCACATGCAGCCCAGGCCCCAGGGTTGCAACACGGCAATGGGCAATGTCAACA
AGTCTACAACGCATTGTAAAGGCCCCAGCACCTTCCTGCATGAATCCTTCTCCCGCGTGGCCACCACCTGTCAGACCCCCACCGTAGCC
TGCAAGAATGCCCAGGAGAACTGCCACCTAGGCCAAGGTGTCCAGACTGCTGTATAAAGAGAAGCAGCTGGCCACTCCCTACACCATGG
TCTATGTCCCTCCACAGCAGAAAGATGGCCCGGTTCCAGCTGGTATCAAGAGTCCCCGTGCACTTGGCAAAACTATCAAAAGCCGGGTG
TCCCCCCCATCCCCACCCCAAGCTCTGCAG

>Am-RNase9
ATGCGGGCTCCGAGGGCGGCGCGGCCCCTGGCTCTGCTGCTCTTGTTGCTGCAGCCGCTGCAGTTTAAATTTTTGCGTATGCATCTCGG
CAAGTCAGAGGAAGAGACGGAAGAGTTTGAAGATTACTTGGAGGAGCTGCTCAGGCCAGGACCTGCCAGACCTCTTACCAAAGATGCTT
TCCAACAACGTACGATTATCGATCCCACAAGACCACTAACTGATCCAGAGTACTGTACTGCGGAAATGAAGATGAAAAATGTTCACAAC
AAGTTCCGTTGTGTCAGAGAACATTTCTTCCTCCAAGTGGCATATGAGGAGTTGCAAAAGCTCTGTAAAAACATATTCGTGCCTTGCAA
GAATGGAGTTAAGAAATGTCACAGGAGCAGGCAACTGATAGAAGGGGTGTATTGTAATTTAACAAGAGGGGTTAGAATGTCTGACTGTG
AATACGAGTCATCTTATAGGCAGGGCTATGTCCTTATCACTTGTCGATGGCAAAACGACATTCAAGAAATTATTCCTGCTTACGTAAAT
GATATTATGACTTTAGATAAACATGTCAAAGGTGGTAACATCTTCTACAGTGAACTAGAGTGGCCGTTCTGA

>Am-RNase10
ATGAAGCTGACCCTGGTACAGATCTTCTTCATGATGTTGCTGCTCCTGCTGGGCCTGGGGCTGGGCCTGGGGCTGGGCCTTCAGATGGC
TGCAGCCATCCTGGAGGACAGCGATCAGTCCCTGAGTGAGCTCTGGTCCAGCGACTCAGACGACAAGACCAAGGCCACTAAAGGAGAGG
GCAGCCGAACTGCAGAAACCTTGCTGCTTAGCAACAAAGGAGTGGTGCAACCAGGCTGGTCCGAAGACACCGTCCTCGGGGAAGACGAA
GTTCGGGGAAGCAAGATGCTCAGAGACGAGCCTCCCCTTCACAGCCACAAAGACTACCTTAGGTCTGACCTGATGACCAGGGAGTGCAA
CACCCTGATGGCCCAGAAGCTGAAGGAGCACAACCGCACGTGCATAAGCCAGTACACGTTCATCCACGAGGATGTGGATACGGTCAAGG
CCGTCTGTAACAGTCCTGTCGTTGCCTGTGAGCTCAAGGGGGGCAAATGCCACAAAAGCTCCCGTCCTTTCGATTTGACACTCTGCAGG
CTGTCCAAACCAGGCCAGGTCACTCCTCACTGCAATTACCTCACATTTATTTTTGAAAGGCGTATTATTATATCCTGTCCTGACATGAA
GGTCCAGATAATGTCTGGACAATGA

>Am-RNase11
ATGGAGACCTTCTCTCTGCTGCTACTCAGCCTGGGTTTGGTTCTTGCAGGAGATTCAGAAAGCCTAATGGAGAGAATTAAAGAAGAATT
CTCAGGGGGAGAGATGCAATATGACATGGGAAACAGTGACCAAGGCAAACAGACTATTGAGGTATTAATGAACTTGACTCTGTTATATA
AAAACACCAGCCTCCGTATGTCCAAGGATATTCTGTCTTCCTCATTATTGACATTTAGAAGATTACGTTATAGCTTCGCCAAAGGAAAC
AGTCCAGGTAATGACAAAGAGTATCGCAATGACATGGTGGTCTGGAGAGAAGTTTCAGAAGCTAATGGGTCACGCAAGTTAAGTTATAA
CTTCACCCATGGTTCCATGGGAGTCATTCACGGGGCCCCCAAAGCCTCCAGCTGCAAGTGTGGACAGAATCTCGGCACAAACTGCTCTA
GGAGCCCAGAACTGGACACCACTACGTGCCAGCTTGCTGTGGGCGAACAATTCCCCAGGTGCCAATACCACAGTGATACCTCATTAAAG
AAAATACTGGCAGTGCTGACAGGTCATTCTCTGATGAGCTGGTTGGTTAGTGGCTCTAAGCTG

>Am-RNase12
ATGATTCTAATGGTGATCATTTTCCTGATGCTTCTGTTCTGGGAGAACGAGCTGAATGAGGAAGGAGAAGTGTGGACCATGGAGCGGTT
GCACGTGGACTACCCCAAGAGTGACATTCCCGTAAGGTACTGCAACCGTATGGTCTTACAAAGAGTCATCAGGGGACCTGACAACACCT
GCAAGAAGGAGCATGTTTTCATCCACGAGAGGCCTCGAAATATCAATAGGGTCTGCACCTCTTCCAAAAAGAGGATTTGTCAGAACCGT
TCTTCCACTTTATGTTTCCAGAGTATGACCAAGTTCAAAATGACAGTGTGCCAGCTCGTGGGGGGCACCAGATACCCTGCCTGCAGGTA
CCGCATGTCCTCTATGAAGGGGTTCGTTGCCTTCACTTGTGATGACATGGGGCCAGTTAGTCTCCAGGGATACGTTGAA

>Am-RNase13
ATGGCACCGGCTGTGGCCCGGGTCCTGTTCCTCCAGCTTGTTCTAGGGCCAACTCTGGTCGTGGACATCAAGGTGCAGATCGCCATCAA
GAACTTCCGCATGTTACACGTCGACTACCCCAGGGTCAACTACCCAGAGGGGTTCCAGGGCTACTGCAATGGTCTCATGGCCTATGTGC
GGGGCAGAAAACAGAGCTGGTTTTGCCCCCAAATCCATTATACGGTGCACGCCCCCTGGGGAGAAATCAAGAGGTTCTGTTTGTACAGC
GACAGCTTCTGCGAGAATTACGATGAATACTGTACAGTCACAGAGGACTCCTTCCCTGTCACGATCTGCTCTCTGGCCTCTGGACAGCC
GCCCGTGAGCTGCCACTACAATGACACCCTAACCAACCAAAGACTCTACCTGCTGTGTTCTGGAAAGCATGATGCTGAACCGATAGGTA
TCATCGGCCTCTACTAG

>Am-RNase6ps (?)
GTGGGGCTGGAACTCACAACCCCGAGATCACATGCTCCACTGACTGAGCCAGCCAGGTGCCCCAAGGCCTAGCCCTTCTATGGTGGCAT
CCGGGCTGCCGGAGGTCCAGGATACCACGTGGGTCCAGTCAGTATGGCCTGGTGAGGAAGCAGCTCAGGGGAATGGTCTCCAGGGTCTG
CAAGTCCATCAATACCCTCATTCATGAGAGCTTCTGAAGGATAAATGACATACACAGCGCCATACCTAGAAGGTGACAGAATCCTGCAA
TGGACAACTATTACCTCAGCCTCTGAAAACGCATGATCGCCAAGTGCGTGGTCTCCGGGGGTCCAGGCCCCCTGACTGTCCTTCCAGAT
CTAGGATCTGGTCCCTTTGTCTCCTCGTGGCCCGTGTGTGGAACTAGCCAGTTCAGCTGGAGGGCTGG

Microbat (*Myotis lucifugus*)

>Ml-RNase1A
ATGGCTCAGGAGAAGTCCCTCATCCTGTCCCCACTGCTAGTCCTGGTGCTGTTAGTGCTGGGGTGGGTCCAGCCTTCCCTGGGCAAGGA
ATCACGGGCCATGAAGTTCCAGCGGCAACACATGGACCCAGACGGCCCCAGCAACAACTCCAACTACTGCAACCAAATGATGAGACGCC
GGCATATGACAGAGGGACGGTGCAAGCCAGTGAACACCTTCGTGCATGAGCCTCTGGTAGATGTCCAGGCCGTTTGCCTCCAGGGAAAC
ATCACCTGCAAGAATGGGCAGCCCAACTGCCACAAGAGCAGCTCCAGCATGAAGATCACGGACTGTCGTGTGACGAGAGGCTCCAAGTA
CCCCAACTGTGACTACCGAACCAGCCAGAAAGAGAGGCACATCATCGTGGCCTGTGAGGGAAACCCGTACGTGCCCGTCCACTTTGATG
CTTCAGTAGAGGCCTCCAGCTGA

>Ml-RNase1B
ATGGCTCAGGAGAAGTCCCCCATCCTGTTCCCACTGCTAGTCCTGGTGCTGTTAGTGCTGGGGTGGGTCCAGCCTTCCCTGGGCAAGGA
ATCACGAGCCATGAAGTTCCAGCGGCAACACATGGACCCAGATGGCCCCAGCAACAACTCCAACTACTGCAACCAAATGATGAGGCGCC
GGCTTATGACAGAGGGACGGTGCAAGCCAGTGAACACCTTCGTGCACGAGCCTCTGGTAGATGTCCAGGCCGTTTGCCTCCAGAGAAAC
ATCACCTGCAAGAATGGGCAGCCCAACTGCCACAAGAGCAGCTCCAGCATGAAGATCACGGACTGTCGTGTGACAAGCGGCTCCAAGTA
CCCCAACTGTGACTACCGAACCAGCCAGAAAGAGAGGCACATCATTGTGGCCTGTGAGGGAAACCCGTACGTGCCCGTCCACTTTGATG
CTTCAGTAGAGGCCTCCAGCTGA

>Ml-RNase1C
ATGGCTCAGGAGAAGTCCCTCATCCTGTTCCCACTGCTAGTCCTGGTGCTATTAGTGCTGGGGTGGGTCCAGCCTTCCCTGGGCAAGGA
ATCACGGGCCATGAAGTTCCAGCGGCAACACATGGACCCAGATGGCCTCAGCAACAACTCCAACTACTGCAACCAAATGATGAGGCGCC
GGCATATGACAGAGGGACGGTGCAAGCCAGTGAACACCTTCGTGCACGAGCCTCTGGTAGATGTCCAGGCCATTTGCCTCCAGGGAAAC
ATCACCTGCAAGAATGGGAAGCCCAACTGCCACAGGAGCAGCTCCAGCATGAAGATCACGGACTGTCGTGTGACGAGTGGCTCCAAGTA
TCCCAACTGCGACTACCGAACCAGCCAGAAAGAGAGGCACATCATCGTGGCCTGTGAGGGAAACCCATACGTGCCCGTCCACTTTGATG
CTTCAGTAGAGGTCTCCAGCTGA

>Ml-RNase1D
ATGGCTCAGGAGAAGTCCCCCATCCTGTTCCCACTGCTAGTCCTGGTGCTGTTAGTGCTGGGGTGGGTCCAGCCTTCCCTGGGCAAGGA
ATCACGGGCCATGAAGTTCCAGCGGCAACACATGGACCCAGATGGCCCCAGCAACAACTCCAACTACTGCAACCAAATGATGAGGCGCC
GGCTTATGACAGAGGGACGGTGCAAGCCAGTGAACACCTTCGTGCACGAGCCTCTGGTAGATGTCCAGGCCGTTTGCCTCCAGGGAAAC
ATCACCTGCAAGAATGGGCAGCCCAACTGCCACAAGAGCAGCTCCAGCATGAAGATCACGGACTGTCGTGTGACAAGCGGCTCCAAGTA
CCCCAACTGTGACTACCGAACCAGCCAGAAAAAGAGGCACATCATCGTGGCCTGTGAGGGAAACCCATACGTGCCCGTCCACTTTGATG
CTTCAGTAGAAGTCTCCACCTGA

>Ml-RNase1E
ATGGCTCAGGAGAAGTCCCTCATCCTGTTCCCACTGTTAGTCCTGGTGCTGTTAGTGCTGGGGTGGGTCCAGCCTTCCCTGGGCAAGGA
ATCACGGGCCATGAAGTTCCAGCGGCAACACATGGACCCAGGCGGCCCCAGCAACAACTCCAACTACTGCAACCAAATGATGAGGCGCC
GGAATATGACAAAGGGATGGTGCAAGCGAGTGAACACCTTCGTGCACGAGCCTCTGGTAGATGTCCAGGCCGTTTGCCTCCAGGGAAAC
ATCACCTGCAAGAATGGGGAGCCCAACTGCCACAAGAGCAACTCCAGCATGAATATCACAGACTGTCATGAGACGAGTGGCTCCAAGTA
CCCCAACTGTGACTACCAGACCAGCCACAAAGAGAGGCACATCATCGTGGCCTGTGAGGGAAACCCATACGTGCCCGTCCACTTTGATG
CTTCAGTAGAGGTCTCCATCGGAGATCAGAGCAGTGAGATGCCCCTCGAGTCATCTGTCTCTCCCTCATCCTTGCTACCACTGAGGGAA
ATGACTCAAGGTAGGGCTGCTATGCAACACAAAAGTGTTTCCCTGGCCTGA

>Ml-RNase1F
ATGGCTCAGGAGAAGTCCCTCATCCTGTTCCCACTGCTAGTCCTGGTGCTGTTAGTGCTGGGGTGGGTCCAGCCTTCCCTGGGCAAGGA
ATCATGGGCCATGATGTTCCAGCGGCAACACATGGACCCAGATGGCCCCAGCAGCAACTCCAACTACTGCAACCAAATGATGAGGCGCC
AGCTTATGACAGAGAGACAGTGCAAGCCAGTGAACACCTTCATTCACGAGCCTCTGGTAGATGTCCAGGCCATTTGCCTCCAGGGAAAT
ATCATCTGCAAGAATGGGAAGCCCAACTGCCACAAGAGCAGCTCCAGCATGAAGATCACGGACTGTCGTGTGAAGAGCAGCTCTGAGTA
CCCCTTCTGTGACTACGAGACCAGCCACAAAGAGAGGCACATCATCGTGGTCTGTGGGGGAAACCCGTACGTGCCCGTCCACTTTCATG
CTTCAGTAGAGGTCTCCACCTGA

>Ml-RNase1G
ATGGCTCAGGAGAAGTCCCTCATCCTGTTCCCACTGCTAGTCCTGGTGCTGTTAGTGCTGGGGTGGGTCCAGCCTTCCCTGGTCAAGGA
ATCACCAGCCATGAAGTTCCAGAGACGACACATGGACCCAGGCGGCCCCAGCAACAACTCCAACTACTGCAACCAAATGATGAGGCGCC
GGAATATGACAGAGGGACGGTGCAAGCCAGTGAACACCTTCATTCACAAGCCTCTGGCAGATGTCCAGGCCATTTGCCTCCAGGGAAAC
ACCCCCTGCAAGCATGGGCAGCCCAACTGCCACAAGAGCAGCTCCAGAATGAAGATCACGGACTGTCGTGAGACGAATGGCTCCCAGTA
CCCCAACTGTGATTACCGAAACAGCCACAAAGAGAGGCACATCATCGTGGCCTGTGCAGGAAACCCATACGTGCCCGTCCACTTTGATG
CTTCAGTAGAGGTCGCCACCTGA

>Ml-RNase2/3A
ATGGTTCCAACACAGCGGGATTCCCAGCTTTGTCTCCTTCTGCTGCTGGGGCTCATGGGAATGGTGATCTCCTTCCATGCCCCACCTAG
GGGTCTAACCTGGGCTCAGTGGTTTGAAGTTCAACACGTAAACATGACCAACACTCGATGCACCATTGCAATGCAGGCGATTAACCGTT
TACGGAGGCCATGCAAAGGCCAAAATACTTTCATCCACACATCACTTGCTGCTGTCGTTAATCTCTGTTACACAGCAAATATAACCTGC
CGTAATGGACGTGATAAAAATTGCTATAGAAGCCGAGTTGCAGTGCATCTTACCTACTGCAACCTCACCAGACCAGCACATCCTTACTA
TCAGTGCCAATACCAGCAAGTAGCCTTACTGAGAAACTACAGTGTTGCCTGTGACAACGGACACATTCCAGTTCACTTCGATAGAATCA
TCTAA

>Ml-RNase2/3B
ATGGTTCCTACACAGCGGGATTCCCAGCTTTGTCTCCTTCTGCTGCTGGGGCTCATGGGAATGGTGATCTCCTTCCATGCCCCACCTGG
GGGTCTAAGCTGGGCTCAGTGGTTTGAAGTTCAACACGTAAACATGACCAATGCTCAATGCACCATTGCAATGCAGCCGATTAACCGCC
TTAATTATGTGAGTTTCCAGAGGCCGTGCAAAGGCCAAAATACTTTCATCAACACATCGCTTGCTGCTGTTGTTAATCTCTGTTCCGGA
ACAAATATAACCTGCCGTAATAGGGTTGATAAAAATTGCTATAGAAGCCGAGTTGCAGTGAATCTTACCTACTGCAACCTCACAGGACC

GGCACGACATTACAATCAGTGCCAATACCAGCAAGTAACCTTACTGAGAAACTACAGTGTTGCCTGTGACAATGGACACATTCCAGTTC
ACTTCGATAGAATCATCTAA

>Ml-RNase4A
ATGGCTCTCCTGAAGACCCTTTCATCGCTTCTGCTCTTGCTGCTGACCCTGTTGGGGCTGGGGCTGGTGCAGCCCTCCTATGGCCAGCC
CATGTACAAACGATTCCTGCGGCAACATGTGGATCCTACAGTGACAGGTGGCGATGTTAAATACTGTAACTTGATGATGCAAAGACGGA
GGATGACTCAGTTTCGTTGCAAGCAATTCAACACCTTCATCCATGAAGACATCTGGAACATTCGTAGCATCTGCAGCACCACCAACATC
CAGTGCAAGAATGGCAAGATGAACTGCCATGAGGGTGTAATGAGTGTTACAGATTGCAGAGATACAGGAAGTTCCCCAGCCCCCAACTG
CAGATATGGGGCCAGGGCCAGCACTAGGCGTGTTGTCATTGCCTGTGAGGGTAACCCCCCAGTGCCTGTGCACTTTGACGGCTAG

>Ml-RNase4B
ATGGCTCTCCTGAAGACCCTTTCATCGCTTCTGCTCTTGCTGCTGACCCTGCTGGGGCTGGGGCTGGTGCAGCCCTCCTATGGCCAGCC
CATGTACAAACGATTCCTGCGGCAACATGTGGATCCTACAGTGACAGGTGGCGACGTTAAATACTGTAACTTGATGATGCAAAGACGGA
GGATGACTCAGTTTCGTTGCAAGCAATTCAACACCTTCATCCATGAAGACATCTGGAACATTCGTAGCATCTGCAGCACCACCAACATC
CAGTGCAAGAATGGCAAGATGAACTGCCATGAGGGTGTAGTGAGTGTTACAGACTGCAGAGATACAGGAAGTTCCCCAGCCCCCAACTG
CAGATATGGGGCCAGGGCCAGCACTAGGCGTGTTGTCATTGCCTGTGAGGGTAACCCCCCAGTGCCTGTGCACTTTGACAGCTAG

>Ml-RNase4psA
ATGGCTCTCCAGAAGACCCTTTCATTGCTTCTGCTCTCGCTGCTGACCCTGCTGGGTTTGGGGCTGGTGCAGCCCTCCTATGGCCAGCC
CATGTACCAACGATTCCTGCGGCAACATGTGGATCCTATGGTGACAAGTGACGACGTTAAATACTGTAACTTGATGACACAAAGATGGA
GGGTGACTCAGTTTCATTTCAAGCAATTCAACACCTTCATCCATGAAGACATCTAGAACATTCATAGCATCTGCAGCACCACCAATATC
CAGTGCAAGAATGGCAAGATGAACTGCCATGAGGGTGTAGTGAGTGTTACAGACTGCAGAGATACAGGAAGTTCCCCAGCCCCCAACTG
CAGATATGGGGCCAGGGCCAGCACTAGGCGTGTGGTCATTGCCTGTGAGGGTAACCCCCCAGTGCCTGTGCACTTTGACAGATAG

>Ml-RNase4C
ATGGCTCTCCAGAAGACCCTTTCATTGCTTCTGCTCTTGCTGCTGACCCTGCTGGGGTTGGGGCTGGTGCAGCCCTCCTATAGCGAGAC
CATGTATGAACGATTCCTGCGGCAACACGTGGACTTTCCAGCGACAGGTGGCACCAACTTGTACTGCAACACGATGATGCAAAGACGGG
GGATGACTACCCCTAGTTGCAAGCAATTCAACACCTTCATCCATGAAGACAACAGCACCATTGATACCATCTGCATCACCCGCAATATC
CTGTGCAAGAATGGCATGATGAACTGCCATGCGGGTGTAGTGAATGTTACAGATTGCAGAGATACAGGAAGAACCCGGCCCCCCAACTG
TTCATATCAGGGCAGGGCCAGCACTAGGCGTGTTGTCATTGCCTGTAATGGTAACCCCCCGCTGCCTGTGCACCTTGACAGATAG

>Ml-RNase4D
ATGGCTCTCCAGAAGACCCTTTCATTGCTTCTGCTCTCACTGCTGACCCTGCTGGGGCTGGGGCTGGTGCAGCCCTCCTATGGCCAGTT
CATGTACCAACGATTCCTGCGGCAACATGTTGATTCTACACAGAGAGGTGTCACCAGCTCGTACTGCAACACGATGATGGAAAGACGGG
GGATGACTAGGCCTAGGTGCAAGCAATTCAACACCTTCATCCATGCAGACATCGGCGCCATTAACAACATCTGCAGGACCCCCAATATC
CGGTGCAAGAATGGCAAGGAGAACTGCCATGAGGGTGTAGTGAGAGTCACAGGCTGCAGGCTTACAGGAAGTTCCTGGGGAAACTGCAG
ATATCAGGGCAGGGGCAGCTCTAGGCGTGTTGTCATCGCCTGTGAGGGTAACCCCCAGGTGCCTGTGCACTTTGACGGATAG

>Ml-RNase4E
ATGGCTCTCCAGAAGACCCTTTCATTGCTTCTGCTCTCGCTGCTGACCCTGCTGGGGCTGGGGCTGGTGCAGCCCTCCTATGGCGAGAC
CATGCACCAACGATTCCTGCGGAACATGTGGACTCTAATTGGACAGGTGGCACAACTTGGTACTGCAACACGATGATGCAAAGACGGG
GGATGACCAGGCCTAGGTGCAAGCAATTGAACACCTTCATCCATGCAGACATCGGGACCATTAAGAACATCTGCAGGACCCCCAATATC
CGGTGCAAGAATGGCAGGATGAACTGCCATGGGCGTCATGTGAGAGTCACAGAATGCAGTCTTACAGCAAGTTCCCAGCGAAAGTGCAG
TTATCAGGGCAGGGGCCGCTCTAGGCGTGTTGTCATCGCCTGTCAGGGTAACCCCCCAGTGCCTGTGCGCTTTGACAAATAG

>Ml-RNase4F
ATGGCTCTCCAGAAGACCCTTTCATTGCTTCTGCTCTCGCTGCTGACCCTGCTGGGGCTATGGCTGGTGCAGCCCTCCTATGGCGAGAC
CAGATATGAAAAATTCCAGCGGCAACATGTTGACTCTACAGGGCCAGGTGGCACCGACTTTTACTGCATCAATAGGATGGACCAAAAGG
GGATGACTAGGCCTAAGTGCAAGGATTTCAACACCTTCATCCACGAAAACATCAAGACCATTAATAACATCTGCAACAACCCCAATATT
CTGTGCAAAAATCGCTATGACATGAACTGCCATGAGGGTGTAGTGAGAGTCACAGACTGCAGGCTTACAGAACGTTCCCCGCACTGCAG
ATATCAGGGCAGGGGCCGCACTAGGCGTGTTGTCATTGCCTGTGACGGTTACCCCCTGGTGCCTGTGCACTTTGACAGCTAG

>Ml-RNase4G
ATGGCTCTCCAGAAGACCCTTTCATTGCTTCTGCTCTCGCTGCTGACCCTGCTGGGGCTATGGCTGGTGCAGCCCTCCTATGACCAGGG
CAACTATGAAAAATTCCAGCGGCAACATGTGGACTCTAGAGGGCCAGGTGGCACCGACTTGTACTGCAACACGATGATGCAAAAAAGAG
GGATGACTTTGGATGGTTGCAAGAAATTCAACACCTTCATCCATGAAGACATCGGGACCATTAATAACATCTGCAACGCCCCCAATATC
CGGTGCAAGAAAGGCAGGATGAACTGTCATGCGGGTGTAGTGAGAGTCACAGACTGCAGTCTTACAGGAGGTCACTCGCCCTACGACTG
CAGATATGGGGCCAGGGCCAGCACTAGGCGTGTTGTCATTGCCTGTGAGGGTAACCCCCTGGTGCCTGTGCACTTTGACAGCTAG

>Ml-RNase4H
ATGGCTCTCCAGAAGACCCTTTCATTGCTTCTGCTCTTGCTGCTGACCCTGCTGGGGCTGGGGTTGGTGCAGCCCTCCTATGGCCAGTC
CACGTACCAACGATTCCTGCGGCAACACGTGGACTCTAGAGGGCCAGGTGGCACCAACTTGTACTGCAACACGATGATGCAAAGACGGG
GAATGACCAGGCCTACGTGCAAGCAATTCAACACCTTCATCCACGAAAACATCAGGACCATTAACAACATCTGCAGGACCACCAACATC
CGGTGCAAGAATGGCAGGATGAACTGCCATGCAGGTGTAGTGAGAGTCACAGACTGCAGGCTTACAGCACGTTTCCCACAAAACTGCAG
ATATCAGGGCAGGGGCAGCTCTAGGCGTGTTGTCATTGCCTGTGAGGGTAACCCCCTGGTGCCTGTGCACCTTGACAGCTAG

>Ml-RNase4I
ATGGCTCTCCAGAAGACCCTTTCATTGCTTGTGCTCTCGCTGCTGACCTTGCTGGGGCTGGGGCTGGTGCAGCCCTCCTATGGCGAGAC
CAGAAATGAAAAATTCCAGCGGCAACATGTTGACTCTATAGGGCCAGGTGGCACCAACTTGGTACTGCAACAGGAGGATGGACAAACAGG
AGATGACTAAGCTTAAGTGCAAGCCATTCAACACCTTCATCCACGAAAACATCGGGACCATTAATAACATCTGCAACAACCCCAATATC

CGGTGCAAGAATGGCATGATGAACTGCCATGCAGGTGTAGTGAGAGTCACAGACTGCAAAGATACAGGAAGTTCCTCTCCCCCCAACTG
CTCATACAAGGCCACTGCCAGCACTAGGCGTGTTGTCATCGCCTGTCAGGGTAACCCCCTGGTGCCTGTGCACATTGACAGATAG

>Ml-RNase4J
ATGGCTCTCCAGAAGACCCTTTCATTGCTTCTGCTCTCACTGCTGATGCTGCTGTGGCTGGGGCTGGTGCAGCCCTCCTATGGCGAGAC
TATGTACCAACGATTCCTGCGGGAACATGTGGACTCTACAGGGACAGGTGGCAACAGCTCGTACTGCAAAAAAATGATGCAAGAACGGG
AGATGACTAGGCCTAGGTGCAAGCGATTCAACACCTTCATCCATGAAGACATCGGCACCATTAAGAACATCTGCAAGACCCCCCAAATC
CCGTGCAAGAATGGCAAGATGAACTGCCATGAGGGTGAAGTGAGAGTCACAGACTGCAGGCTTACAGCACGTTTCCGGGGAAACTGCAG
ATATCAGGGCAGGGGCAGCTCTAGGCGTGTTGTCATTGCCTGTCAGGGTAAGCTGCCTGTGCACTTTGACAAATAG

>Ml-RNase4psB
ATGACTCTCCAGAAGAATCTTTCATAGCTTCTGCTCTTGCTGCTGACCCTGCTGGAGCTGAGGCTGGTGCAGCCCTCCTATGGACAGAA
CATGTACCAACGATTCCTGGGCCAACATGTGGGCCCTACAGTGACAAGGGCAACAACTTGTACTGCAACAACACGATGAAAACCCGGGG
GATGACTCCGTTTAGTTACAAGCGATTCAACACTTTCATCCATGAAAATATCGGGACCACTAACAGCATCTGCAGCACCACCAATATCC
AGCGCAAGAATGGCAAGATGAGCTGCCTGCGAGTGTAGTGAAAGTCACAGACTGCGCGATTATGGGATGTACCCTGCCCCCCAAATGCA
ATTATCAGGCCACGGCCAGCACTAGGCGTGTTGTCATTGCCTGTGAGGGTAACCCCCTGGTGCCTGTGCACTTTGACAGATAG

>Ml-RNase4K
ATGGCTCTCCAGAAGACCCTTTCATTGCTTCTGCTGTTGTTGCTGACCCTGCTGGGGTTGGGGTTCGTACAGCCCTCCTATGGCCAGTT
CATGTACCAACCATTACAGCAACAACACGTGGACTCTACACAGAGAGGTGGCACCAGCTTATACCGCAACACAATGATGCAAAGACAGG
GGATGACTAGGCTTAGGTGCAAGCAATTCAACACCTTCATCCACGAAAACATTGGTGCCATTAACAACATCTGCAGGACCCCCAATATC
CAGTGCAGGAATGGCAAGGAGAACTGCCATGAGGGTGTAGTGAGAGTCACAGACTGCAGGCTTACAGCAAGTTTCCCGGTAAACTGCAG
ATATGAGGGCAGGGGCAGCTCTAGGCATGTTGTCATTGCCTGTGAGGGTAACCCCCAGGTGCCTGTGCACTTTGACGGATAG

>Ml-RNase4psC
ATGGCTCTCCAGAAACCCTTTCATTGCTTCTGCTGTTGTTGCTGACCCTGCTGGGGTTGGGGTTCGTACAGCCCTCCTATGGCCAGTTC
ATGTACCAACCATTACAGCAACAACACGTGGACTCTACACAGAGAGGTGGCACCAGCTTATACCGCAACACAATGATGCAAAGACAGGG
GATGACTAGGCTTAGGTGCAAGCAATTCAACACCTTCATCCACGAAAACATTGGTGCCATTAACAACATCTGCAGGACCCCCAATATCC
AGTGCAGGAATGGCAAGGAGAACTGCCATGAGGGTGTAGTAAGAGTCACAGGCTGCAGGCTTACAGCAAGTTTCCTGGTAAACTGCAGA
TATCAGGGCAGGGGCAGCTCTAGGCATGTTGTCATTGCCTGTGAGGGTAACCCCCAGGTGCCTGTGCACTTTGACGGATAG

>Ml-RNase5A
ATGATGATAGGCCTGGGTCCCCTGTTGTTGGTCTTCATACTGGGGCCCTGTCTGACCCCACCGACCCTGGCTCAGGATGACTACAGGTA
CAGACACTTCCTGGAACAGCACTATGATCTCAATCCAAGAGGCCGGAATGACGCATACTGTGATACCATGATGAGAAGACGAGGCTTGA
CTTCACCCTGCAAAGACACCAACACCTTTATTCATGGCACCAGTAACAACATCAAGGCTGTCTGTGAAGATGAGAATGGAAAGCCTTAT
GGGGCAAATTTCAGAATAAGCAAGTCTCCTTTCCAGGTCACTACTTGCAAGCTCAGAGGAGGGTCCAACCGGCCTCCCTGCCGGTACAG
AGCCACAGCAGGGTCCAGAGACATTGTTATTGCCTGTGAACATGGCTTGCCTGTCCACTTTGATGAGTCCTTTTACCGTCCATAG

>Ml-RNase5B
ATGATGATGGGCCTGGGTCTACTGTTGTTGGTCTTCATGCTGGGGCCCTGTCTGACCCCACCAACCCTGGCTCAGGATGGCAGGTACAA
ACACTTCCTGAACCAGCATTATGATGCCAAACCAAGTGGCCGGAATGACGCATACTGTGATACCATGATGAGGAGACGAGGCTTGACTT
CACCCTGCAAAGACACCAACACCTTTATTCATGGCACCAGTAACAACATCAAGGCTGTCTGTGAAGATAAGAATGGAGTGCCTTATGGG
GCAAATTTAAGAAGAAGCAAGTCTCCTTTCCAGGTCACTACTTGCAAGCTCAGAGGAGGGTCCAACCAGCCTCCCTGCCGGTACAGAGC
CACAGCAGGGTCCAGAGACATTGTTATTGCCTGTGAACGTGGCTTGCCTGTCCACTTTGATGAGTCTTTTTACCGTCCATAG

>Ml-RNase5C
ATGATGATAGGCCTGGGTCCCCTGTTGTTGGTCTTCATGCTGGGGCCCTGTCTGACCCCACCAACCCTGGCTCAGGATGACTACAGGTA
CAGACACTTCCTGGACCAGCACTATGATGCTAATCCACGTGGCCGGAATAACAGATACTGTGATACCATGATGAGGAGACGAGGCTTGA
CTTCACCCTGCAAAGACACCAACACCTTTATTCATGGCACCAGTAACAACATCAAGGCTGTCTGTGGAGATGAGAATGGAATGCCTTAT
AAAGATAACTTCAGAATAAGCAAGTCTCCTTTCCAGGTCACTACTTGCAAGCTCAGAGGAGGGTCCAACCAGCCTCCCTGCCGGTACAG
AGCCACACCAGGGTACAGAGACATTGTTATTGCCTGTGAACATGGCTTGCCTGTCCACTTTGATCAGTCCTTTTACCAACCGTAG

>Ml-RNase5D
ATGATGATGGGCCTGGGTCTACTGTTGTTGGTCTTCATGCTGGGGCCCTGTCTGACCCCACCGACCCTGGCTCAGGATGATCGCAGGTA
CAGACACTTCCTGGATCAGCACTATGATGCCAATCCACATGGCCGGAATAACAAATACTGTGATACCATGATGAGGAGACGAGGCTTGA
CTTCACCCTGCAAAGACACCAACACCTTTATTCATGGCATCAAGAAGAACATTAAGGCTGTCTGTGAAGATGAGAATGGAGTGCCTTAT
GGGACAAATTTAAGAAGAAGCAAGTCTCCTTTCCAGGTCACTATTTGCAAGCTCAGAGGAGGGTCCAACCGGCCTCCCTGCGAGTACAA
AGCCACAGCAGGGTCCAGAGACATTGTTATTGCCTGTGAACGTGGCTTGCCTGTCCACTTTGATGAGTCTTTTTACCGTCCATAG

>Ml-RNase5E
ATGATGACGGGCCTGGGTCCCCTGTTGTTGGTCTTCATGCTGGTGCCCTGTCTGACCCCACCGACCCTGGCTCAGGATGTCGACAGGTA
CAAACACTTCCTGAACCAGCACTATGATCTCAATCCAAGAGGCCGGAATGACGCATACTGTGATACCATGATGAGAAGACGAGGCTTGA
CTTCACCCTGCAAAGCAAAAAACACCTTTATTCATGGCACCAGTAACAACATCAAGGATGTCTGTGGAGATAAGAATGGAGAGCCTTAT
AAGGAAAATTTCAGAAGAAGCAAGTCTCCTTTCCAGGTCACTACTTGCAAGCACATAAGAGGGTCCACCCGGCCTCCCTGCCGGTACAG
AGCCACACCAGGGTACAGAAGCATTGTTATTGCCTGTGAACATGGCTTACCTGTCCACTTTGATGAGTCCATTTACAGTCCCTAA

>Ml-RNase5F
ATGATGACGGGCCTGGGTCCCCTGTTGTTGGTCTTCATGCTGGGGCCCTGTCTGACCCCACCGACCCTGGCTCAGGATGTCGACAGGTA
CAAACACTTCCTGGAACAGCACTATGATCTCAATCCAAGAGGCCGGAATGACGCATACTGTGATACCATGATGAGAAGACGAGGCTTGA
CTTCACCCTGCAAAGACACCAACACCTTTATTCATGGCACCGAAAACAACATCAAGGATATCTGTACAGATAAGAATGGAGAGCCTTAT

AAGGAAAATTTCAGAAGAAGCAAGTCTCCTTTCCAGGTCACTACTTGCAAGCTCAGAGGAGGGTCCACCCGGCCTCCCTGCCGGTACAG
AGCCACAGCAGGGTCCAGAAATATTGCTATTGCCTGTGGACCTGGCTTGCCTGTCCACTTTGATGAGTCCTTTTTCTAA

>Ml-RNase5G
ATGATGATGGGCCTGGGTCCCCTGTTGTTGGTCTTCATGCTGGTGCCCTGTCTGACCCCACCGACCCTGGCTCAGGATGATCGCAGGTA
CAAACACTTCCTAAACCAGCACTATGATGCCAAACCAAGTGGCCGGAATGACGCATACTGTGATACCATGATGAGGAGACGAGGCTTGA
CTTCACCCTGCAAAGCAAAAAACACCTTTATTCATGGCAACAGTAACAACATCAAGGATGTCTGTGGAGATAAGAATGGAGTGCCTTAT
GGGAAAAATTTAAGAAGAAGCAAGTCTCCTTTCCAGGTCACTGCTTGCAAGCACAGAGGAGGTTCCAACCGGCCTCCCTGCCGGTACAG
AGCCACAGCAGGGTCCAGAAGCATTGTTATTGCCTGTGAACATGGCTTGCCTGTCCACTTTGATGAGTCCTTTTTCTAA

>Ml-RNase5psA
ACGATGATGGCCCTGGGTCCCCTGTTGCTGGTCTTCATGCTGGGTCTGTGTCTGACCCCACTGGTCCTGGCTCAGAATGACAACAGGTA
CAGACTCTTCCTGGCCCAGCACTATGATGCCAATCCAAGTGGACACAATGGCAGATACTGTGAAACCATGATGAGGAGATGAGGCCTAA
CTTCACTCTGCAAAGACACCAGTAACAACATCAAGGTTATCTGTGAAGATGAGAATGGAATGCCTTACAGAAAAAAATTTAGAATAAGC
AGGTCTCCTTTCCAGGGCACTACTTGCAGGCATAAAGAGGGTCCCCCTGGCCTCCCTGCCAGTACAAAGCCACATCAGGGTCCAGATGA
GCCCTGTTGATGAGTCCTTTTACTATCCATAACCAGCAAGATCCTGACCCAGAGTTGGTTCTGATCTCCATTCCTGG

>Ml-RNase5psB
ATGATGATGGGCCTGGGTCTCCTGTTGCTGGTCTTCATGCTGGGTCTGGGTCTGACCCCAATGACTCTGGCTCAAATGACAACAGGTAC
AGACTCTTCCTGGCCCAGCACTATGATGCCAATCCAAGTGGCCAAAATGGCAGATACTCTGAAACCATGATGAGGAGATGTGGGCTAAC
TTCACTCTGCAAAGACACCAATAACAACATCAAGGTGGTCTGTGAAGATGAGAATGGAATGCCTTACAGAAAAAATTTCAGAATAAGGA
GCTCTCCTTTCCAGGTCACTACTTGCAGGCATAAAGAGGGTCCCCCTGGCCTCCCTGCCGGTATAGAGAAATATCAGGGTCCACAGACA
TAGTTGTTGCCTGAGAACATGGCTTGCCTGTCCCTTTGATGAGTCCTTTTACCGTCCATAA

>Ml-RNase5psC
ATAATGATGGGCCTGGGTCCCCTGTTGCTGGTCTTCATGCTGGGTCTGGGGTTGACCCCACTGACCTTGGCTCAAATGACAACAGTATA
GACTCTTCCTGGCCCAGCACTATGATGCCAATCCAAGTGGCCACAATGGCAGATACTGTGAAGCCATAATGAGGAGATGAGGCCTAACT
TTACTCTACAAAGACACCAGTAACAGCATCAAGGTCATCTTTGAAGATGAGAATGGAATGCCTTACAAAAATTTCAGAATAAGGAGGTC
TCCTTTCCAGATCACTACTTGCAAGCATAAAGAGGGTCCCCCTGGCCTCCCTGCCAGCACAGAGCCACATCAGGGTCCAGAGACATAGC
TGTAGCCTGTGAACATGGCTAGCCTGTCCCTTTGATGACTCCTTTTACCGTCCATAG

>Ml-RNase5psD
ATGATGATGGGCCTGGGTCTCCTGTTGCTGGTCTTCATGCTGGTTCTGGGACTGACTCCACTGACTCTGGCTCAAATGACAACAGGTAC
AGACTCTTCCTGGCCCAGCACTATGATGCCAATCTAAGTGGCCACAATGGCAGATACTGTGAAACCATGATGAGGAGATGAGGCCTAAC
TTCACTCTGCAAAGACACCAGTAACAACATCAAGGTCATCTGTGAAGATGAGAATGGAATGTCTTATAAAAACTTTTTAAGAATAAGCA
GTTCTCCTTCCCAGGTCACTACATGCAGGCATAAAGAGAGTCCCCTGGTCCCCCTGCCGATGCAGAGACACATCAGGGTACAAGGACAT
AGATGTTGCCTGTGAACATGGTTGCCTGTCCCTTTGATGATTCTTTTTTCCATTCCTGG

>Ml-RNase5psE
ATGATGATGGGCGTGGGTCTCCTGTAGCTGGTTTTCATGCTGGGTCTGGGTCTGACCCCGGTGACCCTGGCTCAAATGACAACAGGTAC
AGACACTTCCTGGCCCAGCACTATGATGCCAATCCAAGTAGCCACAATGGCAGATACTGTGAAACCATGATGAGGAGATGAGGGCTGAC
TTCACTCTGCAAAGACACCAATAACAACTCAAGGTGGTCTGTGAAGATGAGAATGGAATGCCTTACAGAAAAAATTTCAGAATAAGGAG
GTCTCCTTTCCAGGTCACTACTTGCAGGCATAAAGAGGGTCCCCCTGGCCTCCCTGCCAGTACAGAGCCACATCAGGGTCCACAGACAT
AGTTGTTGCCTATGAATATGACCTGCCTATCCCTTTGATGAGTCCTTTTACCGTCCATAA

>Ml-RNase5psF
GTGATGATGGACCTGGGTTCCCTGTTGTTGGTCTTCATGCTGGGTCTGGGGTTGACCCCACTGACGCTGGCTCAAATGACAACACTATA
GACTCTTCCTGGCCCAGCACTATGATGCCAATCCAAGTGGCCACAATGGCAGATACTGTGAAACCATGATGTAGAGATGAGGCCTAACT
TCACTCTGTAAAGACACCAGTAACAACATCAAGGTCATCTGTGAAGATAGGAATGGAATGTCTTACAAAAAATTTCAGAATAAGCAGGT
CTCCTTTTCAGGTCAATATTTGCAGGCATAAAGAGGGTCCCCCTGGCCTCCCTGCCAGTACAGAGCCACATCAGGGTTCAGAGACATAG
TTGTTGCCTGTGAACATGGCTAACCTGTCCTTTTGATGATTCCTTTTACCGTCCATAA

>Ml-RNase5psG
ATGATGATGGCCCTGGGTCCCCTGTTGCTAGTCTTCATGCTGGGTCTGTGTCTGACCCCACTGATCCTGGCTCAGAATGACAACAGGTA
CAGACTCTTCCTGGCCCAGCACTATGATGCCAATCCAAGTGGACACAATGGCAGATACTGTGAAACCATGATGAGGAGATGAGGCCTAA
CTTCACTCTGCAAAGACACCAGTAACAACATCAAGGTCATCTGTGAAGATGAGAATGGAATGCCTTACAGAAAAAAATTTAGAATAAGC
AGGTCTCCTTTCCAGGTCACTACTTGCAGATATAAAGAGGGTCCCCCTGGCCTCCCTACCAGTACAAAGCCACATCAGGGTCCAGATGA
GTCCTGTTGATGAGTCCTTTTACTGTCCATAACCAGCAAGATCCTGACCCAGAGTTGGTTCTGATCTCCATTCCTGG

>Ml-RNase5psH
ATGATGATGGGCTGGGGACCCTGTTGCTGGTCTTCATGCTGGGTCTGGGTCTGAACCCACTGATCCTGGCTCAAATGACAACAGGTACA
GACTCTTCCTGGCCCAGCACTATGATGCCAATCCAAGTGGCCAAAATGGCAGACACTGTGAAACCATGATGAGGAGATGAGGCCTAACT
TCACTCTGTAAAGATACCAGTAACAACATCAAGGTCATCTGTGAGGATGAGAATATAATGCCTTACAGACAATTTCAGAATAAGCAGGT
CTCCTTTCCAGGGCACTACTTGCAGTCATAAAGCGGATCCCCCTGGCCCCCCTGCCAGCACAGAGACACATCACCGTCCAGAGACATAG
CTGTTGCCTGTGAACGTGGCTTGCCTGTCCCTTTGATGAGTCCTTTTACCGCCCATAA

>Ml-RNase5psI
ATGATGATGGGTCTGAGTCTCCTGTTGCTGTTCTGACCCCACTGACCCTGGCTCAAAGGACAATAGGTATAGACTCTTTCTGGCCCAGC
ACTATGATGCCAATCCAAGTGGCCAAAATAGCAGATACTGTGAAACCATAATGAGGAGATGAGGACTAACTTCACTCTGCAAAGATACC
AATAACAACATCAAGGTCCTCTGTGAAGTTGAGAATGGAATGCCTTACGGAAAATAATTTCAGAATAAGCAGTTCTCCTTTCCTGGTCA

CTACTTGCAAGCATAAAAAGGGTCCCCCTGGGCTCCCTGCCCGTACAGAGCCACATCAGTGTCCAGAGACATAGTTGTTGCCTGTGAAC
ATGGCTTGCCTGTCCCTTTGATGAGTCCTTTTACCGCCCATAA

>Ml-RNase6
ATGGTGCTAGATCTCTTGAAATGCTTTCCTCCCCTCCTATTGCTGCTGGGACTATGGGGACCAGTATATCCACTTTATGCTTTGCCTAA
GAATCTCACCAAGGCTCAGTGGTTTGAAATTCAGCATATACAACCAAGCCCTCTCCAATGCAACAAGGCAATGCATGGTGTCAATAATT
ATACTCGGCACTGTAAGCGTCAAAACACCTTTCTGCATGACTCCTTCCAGAATGTGGCTGCTGCCTGTGAGTTGCCCAAGATTGTCTGC
AAGAATGGCCGGAACAATTGCCACCAGAGTTCAAAGCCTGTTAACCTTACTAACTGCAATCTCACTGCAGGGAAGTATCCTAACTGCAG
CTACAAAGATTCTGCCCAGTACAAGTTCTTCATTATTGCCTGTGACCCCCCTCAGAAGAGAGACCCTCCCTATCATTTGATTCCTGTAC
ACTTAGATAAGCTTGTTTAA

>Ml-RNase6psA
ATGGTGCTAGATCTTCTGGAATGATTTCCTCCCCTCCTATTGCTGCTGGGACTTTGGGGACCAGTGTATTCACTTTATGCTTTGCCTAA
GAATCTCACCAAGGCTCAGTGGTTTGAAATTCAGCATATACAACCAAGCCCTCTCCAATGCAACAAGGCAAAGCATGGTGTCAATAATT
ATACTCAGCACTGTAAGCCTCAAAACACCTTTCTGCATGACTCCTTCCAGAATGTGGCTGCTGCCTTGGAGCTGCCCAAGACTGTCTGC
AAGAATGGCCGGAACAATTGCCACCAGGGCCCAAAGCCTGTTAACCTGACTAACTGCGATCTCACTGCAGGGAAGTACCCTAACTGCCG
CTACAAAGATGCTTCCCCGTACAAGTTCTTCATTGTTGCCTGTGAACCCCCTCAGAAGAGAGACCCTCCCTATCATTTGGTTCCTGTAC
ACTTAGATAAGGTTGTTTAA

>Ml-RNase6psB
CTGGTGCTAGATCTTCTGGAATGCTTTCCTCCCCTCCTATTGCTGCTGGGACTATGGGGACCAGTGTATCCACTTTATGCTTTGCCTAA
GAATCTCACCAAGGCTCAGTGGTTTGAAATTCAGCATATACAACCAAGCCCTCTGCAATGCAACAAGGCAATGCATGGTGTCAATAATT
ATACTCAGCACTGTAAGCCTCAAAACACCTTTCTGCAGACTCCTTCCAGAATGTGGCTGCTGCCTTGGAGCTGCCCAAGACTATCTGCA
AGAATGGCCTGAACAATTGCCACCAGAGCCCAAAGCCTGTTAACCTGACTGACTGCAATCTCACTGCAGAGAAATATCCTAACTGCCAC
CAGAAAGATGCTGCCCCGTACAAGTTCTTCATTATTGCCTGTGAGCCCACTCAGAAGAGAGACCCTCCCTATCATTTGGTTCCTCTTCA
CTTAGATAAGGTTGTTTAA

>Ml-RNase6psC
ATGGTGCTAGATCTCCTGGAATGCTTTTCTTCCCTCTTCCTGCTGCTGGGATTATGGGGACCAAATCTTTATGCTTTGCCTAAGAATCT
CACCAAGGCTCAGTGGTTTGAAATTCAGCATATACAACCAAGCCCAACCCAATGCAACAAGGCAATGCATGGTGTCAATAATGATAATG
AAAGGTGTTTTGTAAGCCTCAAAACACCTTTCTGCATGACTCCTTCCAGAATGTGGCTGCTGCCTGTGAGCTGCTGAAGACTGTCTGCA
AGAATGTCCGGAACAATTGCCACCAGAGCCCAAAGTCTGTTAACCTCACTAACTGCAATCTCACTGCAGGGAAGTATCCTAACTGCCGC
TACAAAGATGCTGCCCAGTACAAGTTCTTCATTATTGCCTGTGACCCCCCTCAGAAGAGAGACCCTCCCTCTCATTTGGTTCCTGTTCA
CTTAGATAAGGTTGTTTAA

>Ml-RNase6psD
ATGGTGATCCTTCCCCTCTGTTACCAAACAAAATTCAACAGAGTAAATTTGGCTTTATTAAGTGATTCATAAATCCAGCAGCATCCAAT
CTAGCAACAATGCTTTGCCTAATAATCTCACCAAGGCTCAGTGGTTTGAAATTCATCATACACAACCAAGCCCTCTGCAATGCAACAAG
GCAATGCATGGTGTCAATAATTATACTCAGCACTGTAAGCCTCAAACCACCTTTCTGCATGACTCCTTCCAGAATGTGGCTGCTGCCTG
TGAGTTGCCCAAGATTGTCTGCAAGAAAGGCCACCAGAGCCCCAAACCTATTAACCTCACTAACTGCAATCTCACTGCAGGGAAGTATC
CTAACTGCTGCTACAAAGATGCTGCACAGTACAAGTTCTTCATTAGTGCCTGTGACCCCCCTCAGAAGAGAGACCCTCCCTCTCATTTG
GTTCCTGTTCACTTAGATAAGGTTGTTTAA

>Ml-RNase6psE
ATGGTGATCCTTCCCCTCTGTTACCAAACAAAATTCAACAGAGTAAATCTGGCTTTATTAAGTGATTCATAAATCCAGCAGCATCCAAT
CTAGCAACAATGCTTTGCCTAAAAATCTCACCAATGCTCAGTGTTTTGAAATTCATCATACACAACCAAGCCCTCTGCAATGCAACAAG
GCAATGCATGGTGTCAATAATTATACTCAGCACTGTAAGCCTCAAAACACCTTTCTGCATGACTCCTTCCAGAATGTGGCTGCTGCCTG
TGAGCTGCCCAAGACTGTCCGCAAGAAAGGCCACCAGAGCCCAAAACCTATTAACCTCACTAACTGCAATCTCACTGCAGGGAAGTATC
CTAACTGCCACCACAAAGATGCTGCCCAGTGCAAGTTCTTCATTATTGACTGTGACCTCCCTCAGAGGAGAGACCCTCCCTCTCCTTTG
GTTCCTGTTCACTTAGATAAGGTTGTTTAA

>Ml-RNase6psF
ATTGTGATCTTTCTCTCTTTACCAAATAAAATTCAACAGAGTAAATTTGGCTGTATTAAGTGATTCATAAATCCAGCAGCATCCAATCT
AGCAACAATGCTTTGCCTAAAAATCTCACCAAGGCTCAGTGTTTTGAAAGTCATCATACACAACCAAGCCCTCTCCAATGCAACAAGGC
AATGCATAGTGTCAATAATTATACTCAGCACTGTAAGCCTCAAAACACCTTTCTGCATGACTCCTTCCAGAATGTGGCTGCTGCCTGTG
AGCTGCCCAACACTGTCTGCAAGAAAGGCCACCAGAGCCCAAAACCTATTAACCTCACTAACTGCAATCTCACTGCAGGGAAGTATCCT
AACTGCCACCACAAAGATGCTGCACAGTACAATTTCTTCATTATTGCCTGTGACCCCCTCATTTGCGTCCTGTACACTTAGATAAGGTT
GTTTAA

>Ml-RNase6psG
ATAGTGATCCTTCCCCTCTGTTACCAAACAAAATTCAACAGAGTAAATTAGGCTTTATTAAGAGATTCATAAATCCAGCAGCATCCAAT
CTAGCAGCTATGCTTTGCCTAAAAATCTTACCAAGGCTCAGTGTTTAGAAATTCATCATACACAACCAAACCCTCTGCAATGCAACAAG
GTAATGCATGGTATCAATAATTATACTCAGCACTGTAAGCCTCAAACCACCTTTCTGCATGACTCCTTCCAGAATGTGGCTGCTGCCTG
TGAGCTGCCCAAGACTGTCCGCAAGAATGGCAACCAGAGCCCAAAACCTATTAACCTCACTAACTGCAATCTCACTGCAGGGAAGTATC
CTAACTGCCGCTACAAAGATGCTGCCCAGTGCAAGTTCTGTCTAGAGTTTGTTCTTGTGATTTATTAGTAGTCATCGAAATGCTGGCAT
CACAGGAAACTCTCTTCAAATTAATTTAA

>Ml-RNase7/8
ATGGCCCCAGCCAGAGCAAGATTCTGCCCCCTGGTGCTGCTCCTGCTGCTGGGCCTGTGGGTGGCTCAGGTCCCAGTCAGCGCCAAGCC
CAAACACATGACCTCAGCTCAGTGGTTTGAAATTCAGCACGTGCAGCCCAGCCCCCAGGCATGCAAAGGGGCGATGAGCAACATCAACA
AACACACAAATCACTGCAAACGCCTCAACACCTTCCTGCATGAATCCTTCTCCAGTGTGGCCGCCACCTGCCAGAACCCCACCATAGCC

TGCAAGAACGGCCAGAAAAACTGCCACCAGAGCAAGGGGCCTGTGTCCCTGACCACGTGTGAGCTCACCTCGGGGAAGTACCCAAACTG
CAATTACAAAGAGAACGCACTGCACGCTTCTTACATCATAGCCTGTAACCCTCCTCAGAAAGGGGATGCGGAAAAAGTCCACCTGGTTC
CTGTGCACTTGGACAAAGTCGTTTAG

>Ml-RNase7/8ps
ATGGCCCCAGCCAGAGCGGGATTTGCCCCCTGGTCTGCTCCTGCTGCTGAGGCCATGGGTGGCCCAGGTCAGCACCAAGTCCAAACACA
GTTTCAGCTCAGTGGTTTGAAACTCAGAATGTGCAGCCCAGCCCTCAGGGATGCAAAGGGGTGACGGCAACATCAACAAGCACGCAAAT
CACTGCAGAGGCCTTAACACCTTCCTGCATGAAGCCTTCTCCAGTGTGGCCACCACCTGCCACACCCCCACCATAGCCTGCAAGATCGG
CCAAGAAAGCTGCCACCAGAGCCAGAAGCCCGTGTCCCTGACCACGTGTGAGCTCCCCTAGGGGAGGTACCAAGGTCGCAGGTACAAAG
ACAAGCAACTGGACCTATTCTTCATCGTGGCCCGTGACCCTCCACAACAGGACGGCCTGCCGGCCCTGCCGGTGCCTGTGCCCTGGGTA
ACGCTGTCCAA

>Ml-RNase9
ATGTGCACCCTGCTCACCTCACAGTCCCTGCCTCTGCTGCTCCTGCTGCTGCAGCCACTTCCACTGCGGGTTCTAATGCTTGATAAACA
TGGTTTCTCGGATGAGATGGACGAAAAATTTAAAGATTATATCGATGAATTATATAGTACAGGGCCTACCAGACCACCTACCAAAGAGA
GTTTCCAAAAGTATGTCATGCTTGAACCTGATTACAAATTATATTATGATCACCTGTGTACTCATGAAATTTTATTCAGAAATATCCAC
AACAGATTCTACTGTAAGAAAGAACATTTCTTCCTCCACATAGCATATGAGGAGTTGCAAAAGACCTGTCACACCAAGTATGTGGCATG
TAAGAATGGCATTAGGAAATGTCACAAGACCAAGGAACAAATAGAAGGAGCGCACTGTGCATTAACAGAGGGAACTAACATGCCAGGCT
GTCGGTATGAAACAACTTACAAGAAGGGATATGCTCTTATTACTTGTCGATGGCAAGATGATATTGGAGAAATTATTCCTGATTATGTA
AATAGTATTTTGGAAATACCTGGCAAATAG

>Ml-RNase10
ATGAAGCTGACTCTGGTGCAAATCTTTTTTATGATGTTGCTGCTGTTGCTGGGCCTTGGGATGGGCCTGGGTTTGGGCCTTCGGATGGC
TGCAGCGGTCCTGGAGGATAGTGATCAATCCCTGAATGAATTATTTTGGTCCAGTGACTCACAGGACAAGGCCGGAGTCACTAAGGAGG
GAGAGGGCACCCGAACAACAGAAACCCTGCTGCTTAGCAACAAAGGAGTGGTACAACCTGGCTGGCCTGAAGAGACCATCGTCAATGAA
GATGAAGTTGGAGGAAACAAGATGCTCAGAGCTGAGGCTCTCTCTCAGAGCAACAAAGACTATCCCAGGCTTGACCTGATGGCCCGGGA
ATGCAATACCATGATGGCACACAAGATGAAGGAGCACAACCACACATGCATCACCCAGTACACATTCATCCACGAGGAGCTAGATACAG
TCAAAGCTGTCTGTAAGGGTCCTGTCATTGCCTGTGAGCTCCAGGGACGCAAATGTCACAAAAGCTCCCGTCCTTTTGATTTGACATTT
TGCAGGTTATCCAAACCAGGCCAAGTCACTCCTCACTGCAATTACCTAACTTTCATTTTTGAAAAGTTCATTATTATATCCTGTAGTGA
CATGAAGGTCCAGGTAATACCTAATAGATAG

>Ml-RNase11
ATGGAGGCCTCTCTGCTGCTACTCTGCCTGGGGCTGATTCTTGTAGGATCTTCAGGAAACAAAATGGAGACAATTAAAGAAGAATTTTC
AGAGGGAGAGATGCAAAATGACTTGGCAAAAAGTGACCAAGAAAAACAGACCATTGAGATATTAATGAACTTGACTCTGTTGTATAAAA
ATACTAGCCTTGGCATATCCAAGGATATTACGTCTTCATTATCGACATTCAGAAGATTACATGATAGCTTCTCCAAGGGAAACAATCCA
GGTAATGACAAAGAGTGTTACAATGACATAGTGATCTGGAGAAAAGTTTCAGAAGCTAATGGGTCATGCAAGTTGAGCAATAACTTTAT
TCTTGGCTCCATGGAAGTGATCTGCAGGGCTCCCAAGACCCCCAGCTGCAAGCGTGGACAGAATCTTGGCATAAGCTGTTTTGAAAGCC
CAGACCTGGTGATAACTATGGGCCAGCTTACTACAGGCAAACAGTTCCCCAGGTGCCAATACCACAGTGTTACCTCGTTTAAGAAAATA
TTGGCAGTGCTAACAGGTCATTCTCTGATGAGCTGGTTAGTTAGTGGCTCTAGATTGTAA

>MlRNase12
ATGATACTAATGGTGATAATTTTCCTGATGATTGTGTTCTGGGAAAACGAGCTGTATGAAGAAACAGTGATGTCAACCATAGAACACCT
GCACGTGGACTACCCTCAGAGTAATATTGCTGTAAGGTACTGCAACCACATGATCTTAGAAAGAATCATCAAGGAACCTGACAACACCT
GCAAAAAGGAGCATGTCTTCATCCACGAGAGGCCTCGACAGATCAATCGTGTTTGCACTTCTCCCAAGAGGACGGCTTGCCAAAACCAT
TCCAGCATTTCATGTTTCCAGAGTGAGACAAAGTTCAAAATGACAGTCTGTAAGCTCATTGAAGGTATCAGATATCCTGCCTGCAGGTA
CCACATTTCCCCCACAGAGGGGTTTATTGTCGTCACTTGCGATGACATGGGGCCCGTTAACTTCCAGAAATATGTTGAATAA

>Ml-RNase13
ATGGCACCAGCTGTGGCCTGGCTCCTGTTCCTCCAGCTTGTTCAAGGACCAACTCTGGTCATGGACATCAGCATAGAGGTTGCCATCCA
GAACTTTCGAACCATGCACATTGACTATCCCAAGGTTAACTACCCAGAGGGTTTCCAGGGCTACTGTAATGGTCTGATGGCCTATGTGA
GGGGCAGACAGCAAAGCTGGTATTGCCCAAAGAGGCATTATGTGATACATGCTCCCTGGACGGACATTGAGAAGTCCTGCAAGTACTGC
GAGAGCTTCTGTGAGAATTACAATGAATACTGTACACTCTCTGAGGACTCCTTTCCCCTCACAATCTGCTCTCTGGAGACCAAACAGCC
ACCCACCAGCTGCCGCTACAATAGCACCCTAAACAACCAAAGGCTGTACCTGCTCTGTTCCCAAAAGTATGATGCTAAACCAATAGGTA
TCATTGGCCTCTACTAG

>Ml-RNase14ps
ATGAATATGATGTGGACCCTGCTTCTCCTCCTTCTCTTCTTGGAGCAAACTGTCTTCACTCTAGGCCACAACATGTAGACCACCTCAGG
TCAGGGGTTCCTGGAAGACACCACTGGTAGATACTGTGATATGATGATGAGATGATATTGGCTGATCCATAGGTGTAAATGCAAGCAGA
TCAACACCTTTATTCGGGAGGATTTGGCCACCATAGCAGATTTCTGTAGAACTCCATGTGTGCCCTGCACCAACAGTCATTCCGTGCAG
AATGGTCACAACAGTACTCACAATGTCAGAGTCACTGACTGCTTTGCTAGTGCAGAGATGCCACCCCCGTGCCCTCCAACACACACACA
CACACACACACACACACACTGCCACTACCATAACAAGGACTCCACAAGGCCCATCCAAGGCCCATCCTTGTGGCCTGTGAGAAGGGGAC
ACCTATCCACCTGGATGACTAG

>Ml-RNase15ps
ATGTTCTTACATTAACACAGAATATGTGCTTCTGATGTGGGTTCTCCCAAAGGATCAGTCTGGCTTCTCTGAGGGCCACAAACTTCACG
TTGACAGACCCAAAGCAAAGGTCTCTGGCAAGTACAAATATTATTGTGACGTGATAATGAAGGTTGAGGGCTGGCTACCTAGAAGAGCT
GCAAGAGAGCAAATGTGTCCCCTGACACAGGTGGCCGGTGTTGAGTCCAACTGTGAGGTATGTGTGGAAGCCTGACTTTTACAGTTGTC
AACTCATCATCTTCAAAGTCACTCAATGTAATCTCTACCCAGAGGTCTTGCCACACGCACACTGTTACTACCATGGCATTAGCTCCTGG
AGGAACGTGAGGGTGGTCTGTGTGGGGAAATAGTCTCCACAATTTGCTTGGAGGAATAG

African elephant (*Loxodonta africana*)

```
>La-RNase1
ATGACTCTGGAGAAGTCCTTTGTCCTGCTCCCTCTGCTAGCCCTGATGTTGCTGGTGCTGGGGTGGGCCCAGCCTTCCCTGGGCAAGGA
ATCCCCAGCCAGGAAGTTCCAGCGGCAGCACATGGATTCAGACAGCAATCCTAGCAACAGCTCCAGCTACTGCAACCTAATGATGAGGC
GCCGTAATATGACAAAGGGATATTGCAAGTCAGTGAACACATTTGTGCACAATCCCCTGGTAGATGTCCAGGCCATCTGCCTCCAGAAG
AACATCACCTGCAAGAATGGGCAGCCCAACTGCCACCAGAGCAACTCAAGCATTCCTATCACAGACTGCCGCCTGACGGGCAGCTCCAA
GTACCCCAACTGTGCGTACCGGACCAGCAACAAGATGAGACATATCATCGTGGCCTGTAAGGGGAAACCGTATGTGCCTGTCCACTTTG
ATGGTTCAGTGGAGGTCTCCTCCTTAGGCCAGAGCACACATACCCTCACCACCTCATGA

>La-RNase1psA
ATGGCTCTGGAGAAGTCCTTCGTCCTGTTCCCCTGCTAGCCCTGATGCTGCTGGTGCTGCGGTGGGCCCAGCCTTCCCTGGGTAAGGAA
TCCCCAGCCAGGAAGTTCCAGCGGCAACACATGGATTCCCAGAAAGCAATCCTAGCAACAGCTCTAGTTACTGCAACCTAATGATGAGG
TGCCGTAATATGACAAAGGGATATTGCAAGTCAGTGAACATATTTGTGCAAAATCCCCTGGTAGACATCCAGGCCATCTGCTTCCAGGA
GAACATCACCTGCAAGAACGGGCAGCCCAAATGCCACCACAGCAACTCAAGCATTCCTATCACAGTCTGCCACCTGATGGGCAGCTCCA
AGTACTCCAACTGTGCATACGGGACCAACAACACGATGAGACATATCATCCTGGCCTGTAAGGGGAAACCGTATGTGTCTGTCCACTTT
GATGGTTCCGTGGAGGTCGTCTCCTTAGGCCAGAGCACACATGCCTCCACCACCTCATGATTGTGACA

>La-RNase1psB
ATGACTCTGGAGAAGTCCTTTGTCTTGTTCCCCCTGCTAGCCCTGATACTGTTTGTGCTGGGGTGGGCCCACCTTCCCTGGGCAAGGAA
TCCCCAGCCAGGAAGTTCCAGCAGCAACACATGGATTCAGACAGCAATCCTAGCAACAGCTCTAGCTACTGCAACCTAATGATGAGGCG
CCATAATATGATGAAGGGATATTGCAAGTCAGTGAACACATTTGTGCACAATCCCCTAGTAGATGTCCAGGCCATCTGCCTCCAGGAGA
ACATCATCTATAAGAATGGGCAGCCCAACTGCCACCGGAGCAACTCAAGCATTCGTATCACAGACTGCTGCCTGACAGGCAGCTCCAGT
ACCCCAACTGTGCGTACCAGACCAGCAACAAGATGAGACATATCATCGTGGCCTGTAAGGGGAACCCCTATGTGCCTGTTCAATCTGAT
GGTTGAGTGGAGGTCTCCTCCTTAGGTCAGAGCACAGATGCCCCCACCACCTCATGATTGTGA

>La-RNase2/3ps
ATGGCTCCAAACCTGTACAACTCTCGACTGTGCCTCCTTCTGCGGTTGGGGTTCTTGGGAATGATGAGCACATGCCATGCCCAATCTCA
TTTAACTAGGGCTCAGTGGTTCAATACCCAGCATGTACGACACACCCAGGTCCAGTGCAAAAATAAAATGCAAAGAAGTAACCAATACA
AGAAAATTGCAAAGGCAAAAATGTGTTCCTCCAAACAACCTTTGCTGCGGTAGCTAATGTTTGTCACAGTCCAAATGTCCACTGTAGGA
ATGGCCGCTGGAGCTGTCATAATAGTACAGCACAGGTGCCTTTAACCTACCGTCACCTCACAAGAGGAAGGTATCCAAGCTGCAGGTAT
AGCCGAAGACTCAAAAAGATGTTCTACACTATTGCCTGTGACCAACGATCTCCGCAGGACTCACCTCAATTTTAGATGGTTCCAGTTTG
CTTAGACAGAATCTACTAA

>La-RNase4
ATGACTCTACAGAGGACCGGTTCATTGTTTCTGCTCTTGCTGCTGACCCTGCTGGGGCTGGGGCTGGTGCAGCCCTCCTATGGCCGAGA
GAGCAAGTACCAACGATTCCTGAGGCAACACATGGATCCTGAGGGCTCGGGTGGCAATGATGGCTACTGCAACTTGCTGATGCAAAGAC
GGAAGATGACTACGAGCTGGTGCAAGCCCTTCAACACCTTCATCCATGAAGACATCTGGAACATCCGTAGTATCTGCAGCACCACCAAT
ATCCAGTGCAAGAATGGCAGTATGAACTGCCACGAGGGTGTAGTGAAGGTCACAGACTGCAAGGAGACAGGAAGTTCCAGGGCCCCTAA
CTGCAGATATCGGGCCAAGACCAGCACTAGGCGGGTTGTCATTGCCTGTGAGGGTAATCCAGAGGTACCTGTGCACTTTGACAAATAG

>La-RNase5ps
ATGGTGATGGGCCTGGGCTCCCTGTTGTTGGTCTTCATGCTGGGCCTGGGTCTGACCCCACCAACCCTGACTCAAGACGGCTCCAGTTA
CAAACACTTCCTGACCCAGCACTATGATGCCAAACCAAGGGGCTGGAATGAGAGATATTCTGAAAGCGTAATGGGGAAATGAGGCTTGA
CCACACCCTGCAAAGGCACCAATTCCTTCATTCATGGCAACAAGGGGAACATCAAGGCCATCTGTGGAAATTGGAATGGAATCCCTCCC
ATTATCTCAGGGAACATCTAGCTCAATTGGCATAACACAGCTTATAAAGAAAATGTTCTGCATGCTCTACTTTTTTTTGTTTGTTTGTT
TTTCTACTTTTGTGAGTAGCATCTGGGGTCTTAAAAGCCTGTGAGTGGCCATCTAGGATACGCCACTGGTCTCACCCCTTCGGGAG

>La-RNase6A
ATGGCGCTAGATCTTCTGGGACACTTTCCTCACCTCCTATTGCTGCTGGGATTATGGGGGCCAATGCATCCGCTTTTTGCTGTGCCTCC
GAATTTGACCAGGGCTCAATGGTTTCAAATCCAGCATTTACGGCCAAGTCCTCTACCATGCAATCAGGCAATGAGAGGTGTCAACAATT
ATACCCACCATTGTAAGGATCTAAATACCTTTCTGCATGATTCCTTCCACAATGTGGCTGCTGTCTGTAACTTGGCAAACATCACTTGC
AGGAATAGATCCACCAACTGCCACCGGAGCCCAAACCGTGTTAACATGACCGTGTGCAACCTCACTGCAGGAAGGTATCCTAATTGCAA
CTACAACAGGGCTGCAGCATACAAGTTTTTCATTGTTGCCTGTAATACCCCTCAGGCAGGAGGTCCTCCCGTTCCTGTGCACTTAGATG
GGGTCATTTAA

>La-RNase6B
ATGGTGCTAGATCTTCTGGGACACTTTCCTCACCTCCTATTGCTGCTGGGATTATGGGGGCCGATGCATCCGCTTTTTGCTGTACCTCA
GAATTTGACCAAGGCTGAATGGTTTGAAATCCAGCATTTACAGCCAAGTCCTCTCCTATGCAATCAAGGAATGCGGGGTGTAAACAATT
ATATCCAGCACTGTAAGCCTGTAAATACCTTTCTGCATGACTCCTTCCAGAACGTGACTGCTGTCTGTAACTTGACAAACATCACCTGC
AAGAATGGACTGAACAACTGTCACCAGAGCCAAAACCCTGTTAACATTACTCATTGCAGACTCACTGCAGGAAAGTATCCTAAATGCAC
CTACAAGAATGCTGCCCTATACAAGTCCTTCATTGTTGCCTGTGATCCCCCTCAGACGGGAGACCCTCCCTATCCGTTAGTTCCTGTGC
ACTTAGATAAGATCATTTAA

>La-RNase6C
ATGGTGCTAGATCTTCTGGGACGCTTTCCTCACCTCCTATTGCTGCTGGGATTATGGGGGCCAATGTATCCGCTTTTTGCTGTGCCTCC
GAATTTAACCAAGGCTCAATGGTTTCAAATCCAGCATTTACTGCCAAGTCCTTCCCAATGCAATCAGGCAATGAGAGGTGTCAACAATT
ATACCCAGCACTGTAAGGATGTAAATACCTTCCTGCATGACTCCTTCCAGAATGTGGCTGCTGTCTGTAACTTGACAAACATCAAATGT
AGGAATGGACGGAACAACTGCCACCGGAGCCAAAACCCTGTTAACATGACTAAGTGCACCCTCACTTCAGGAAACTATTCTAACTGCAT
CTACGGGAATGCTGCCCTACGCAAGTCCTTCATTGTTGCCTGTGATCCCCGTCAGAGGGGAGACCCTCCCTATCCATTATTAGTTCCTG
TGCACTTGGATTAG
```

>La-RNase7/8
ATGACACCAGCCAGAGCAGGATTCTGGCCCCTGCTGCTCCTGCTGCTGGGGCTGTGGGTAGCCAATGTCCCAGTCAGTGCCAAACCAAA
GGACAAGACTCCAGCTCAGTGGTTTGAAATTCAGCACGTGCAGCCTCACCCTCGAGGATGCAATGCTGCGATGGGCAGCATCAACAAGG
ACAAAAAACACTGCAAAGGTCTCAACACCTTCCTGCACGATTCCTTCTCCAGTGTGGCCACCACCTGCAAGAACCCCACCATAGCCTGC
AAGAACCATCGAAAAAACTGCCACAAGAGCCGAGGGCCTGTGTCCCTGACCATCTGTGTGCACACCTCGGGGAAGTACCCCAACTGCAA
GTACAAAGAGCAAAGCAAGAAAGCATCCTACATCGTGGCCTGTGACCCTCCCCAGAAAAGTGACTCTGGGCACTTCCAGCTGGTTCCTG
TGCACTTGGACAAAGTCTTTTAG

>La-RNase7/8ps
ATGGCACCAACCAAGACACCAGTTCAGCCTCTGCTGCTGCTCCTGCTGCTGGGACTGTGGGTGGCCCAGGTCCCAGTCAGTGCCAAGCC
CAAGAACATGACCTCAGCTCAGTGGTTTGACACTCAGCACGTGTAACACTGCAATGGAGAACATCAACAAGTACACAAAACTTTGCAAA
AACCTCAACAGCTTTCTGCATGAGTCCTTCTCCAGTGTGGCCACTGCCTGCCAGACCCCCGTTATAGCCTGCAAGAACAGCCGTGAAAA
CTGCCACCAGAGCCAAGAGCCCATGTCCCTGACCCAGAGTGAGCACGTCTCAGGGAGGTACCCAGACTGCAAGTACAGAGAGGAGAACC
TGGATGCATTCTTCCTTGTGGCCTGTGACCCTCCACAACAGAAAGATTACCTGGGGTACCAGCTGGTTCCTGTGCACTTGGACAATGTT
GTCTGA

>La-RNase9
ATGTGGCAGCCTTCTGTCGTCATGTACCCACTGTCTGTGCTACTATTGCTGTTCCCACAAGCGCAGTTCAAAACAACACATAATCCTTT
CATTGTATCAGGTAATTTCGAAGAAGATTTTGAGGATTATTTGACAGAGTTTTATGGTACAGGATCTACCACAGAGCCTACCAAAGAAA
AATTCAAACGTAAGATGATTATTGTACCCGGAAGACAAATACATATAAGTAATGAAGAATACTGTAGCCACGCAATCCTGTTTAAAAAT
ATTCACAATAAGTTGCGCTGTGTGAAAGAACACTACTTCCTCCAAGAGCCATACGAGGAGATAAGAAAAATCTGTTACAACTCATTTAT
AAAATGTAAGAATGGCATTAGAAAATGTAACAGGAGCAAGAAAATGATGGAAGGACTGTATTGTAAATTAAGAAGAGGAACCACATCAC
CAGACTGTGAATATGAATCATTTTATAAGAGAGGACTTGCCATTATCACTTGTAAGTGGCAAAATTCAATTGGAGAACTTGTTCCTGTT
AGCATAAATGATATATCGATGCCATATAGCTATTAG

>La-RNase10
ATGAAGCTGACTCTGGTGCCGATCTTTTTCATGCTGCTGCTGCTGCTGCTGGGCCTAGGGATGGGCCTGGGCCTGGGACTTCACATGGC
CGCAGCAGTCCTGGAGGATAGTGATCAGCCACTGAATGAGTTTTGGGCCAGTGACTCCCAGGACAAGGCTGAGGCTGATGAAGAGGGAG
AGGGCACCCGAACCACAGAAACTCTGGTGCTTAGCGACCAAGGACTAGTACAACCTGGTTGGCAACCAGAAGGCACCATCTTCAATGAA
GATGAGGTTGGGGACAAGATGCTCAGGGCTGATGTTCTCTCTCAGAGCAGCAAAGACTACCTTAGGTATGACCTGCTCTCCAGGGAATG
CAATGCCATGATGGCCCACAAGATGAAGAAGCACAACCAAACCTGCATAACCCAGTACACGTTTCTCCATGAGGATGAAGACACAGTCA
AGGCTGTCTGTAACAGTCCAGTCATTGCCTGTGAGCTCAAAGGGGGCAAGTGTCACAAAAGCACCCGCCCCTTCAATTTAACACACTGC
AAGTTATCCAAACCAGGGCAGGTCACTCCTCACTGCAATTACCTAACTTTCATTTTTGAAAAGTATATCCTTATAACCTGCAATGACAC
GAAGCCCCAGTTATCCCCTGGACAGTGA

>La-RNase11
ATGGAGACTTTTTCTCTGTTATTGCTGGACCTGGGCCTGGTTCTTGCAGGAACCTCAGGAAGCACAATGGAGACAATTAAAGAAGAGTT
TTCAGAGGAAGATATAGAATATGACATAGCAAAAGCTTGCCAAGAAAAACAGACCATTGAGGTATTCATGAACTCGACCCTGTTAGATA
AGAATACCAGCCTCAGCATGTCCAAGGATGTCATGTCTTCCTCATTATTGACATTCAGAAGGTTACATTATAACTTTCCCAAGGAAAAC
AGTCTGGGTAATGGCAAACAGTATTGTAATGCTATGGTGGTCTGGAGAATTGTGTCAGAAGCTAATGGGTCATGCAAATTGAGAAATAA
CTTCACCCATGGCTCCACAGAAATGATCCATGGAATCCACAAGGTCCCCAGTTGTGACTATGAACCAAATTTTGATATGGTTCAAGGCA
ATATGGTGGAACCAAAGTGCTATAAAAGCCCAGAACTGGAGACCACTATGTGCCAGCTCACTACAGGAAAACAACTCTCCAGATGCCAA
TACCACAGTGTTACCTCATTAAAGAAAATGTTGATAGTGCTGACAGGCCATTCTCTAATGAGCTGGTTAGTTAGTGTCTCTAAATTGTA
A

>La-RNase12
ATGGTGATTATTTTCCTGCTGCTTCTCTTCTGGGAAAATGAGATGAACGTAGAATCAGGGATGCCAACCTTGGAGCAGTTGCATGTGGA
TTACCCTCAGAGTGATGTTCCTGTGAGATACTGCAACTACATGATCTTACAAAGAGTCATCAAGGAACCTGACAACACCTGCAAAAAGG
AGCACGTCTTCATCCACGAGAGGCCTCAAAAAATCAACGCTATCTGCACTTCCGTCAAGAAGAAGGCTTGCAGTAACCATTCCAAACTT
TTCTGCTTCCAGAGCAAGATAAAGTTCAAAATGACGGCCTGCGAGCTCATAGAAGGTTCCAGATACCCTGCCTGCAGGTACCACATTTC
TCCCAAGGAGGGGTTCATTCTTGTCACTTGTGATGAAATGGGGGCAGTTAATTTCCAGGGATTTGTTGAATAA

>La-RNase13
ATGGCACCAGCTGTGGCCCGGCTACTTTTCCTCCAGCTTGTTCTAGGATCAGCTCTGGTCCTGGGTATCAACCTGTGGAGTGCCATCAA
GAACTTCAACCACTTATTCATAGACTTCCCCCGGGTTGAGTACTCAAGCAAGTTCCAGGGCTACTGTAATGGCATTATGAGCTATGTGA
GAGGCAGAATGCAAAACTGGGATTGTCCAAAGATCCATTACTTGGTACATGTCCCCTTTAAATCCATCCGGAAGTACTGCAAGCATAGT
GAGAACTTCTGTGAGAACTACAACCACTACTGCACATTAACTAAGGACTCCTTCCCCCTCACGATCTGCCAACTGTCGGACAAACAGCC
GCCTACCAGCTGCTACTACAATACCACCCTAACCAACCAAAAGCTCTACCTGCTCTGTTCCCGCAAGTATGATGCTGATCCAGTAGATA
TCATTGGCCTCTTCTAG

>La-RNase14ps
ATGAATCTGAGGTGGATCCTCTTCCTCCTCCTCCTGGACCAGACTGTCATCAAGCCAGCCCTGTCTTTCTTAACTGGGCATATAGACAA
GCCCAGCTCTTGGGTCCCTGGAGGACTCCACAGGTACTGTGAGGTGATGATGAGACGACGGCGGATGACTCATGGGAACAAATGCAAGC
ACATCAACACCTTCCTCCTAGGATCTGGCCACCAAACCAAACTTCTTCAGAACTGCGCCAGTGCCCTACATCAACAGCCTCTCCATGAA
CAACTGCCACAACAGTACCCAAGACGTCCACGTCACTGACTGCTTTGCCAGCGGAGGGTCCAGGACCCCTCACTGCCACTATCGGAACA
AGGACTCCACCAGGCCCATCCGTGTGGGCTGTGAGGAGGGAACACCCGTTCACCTGGATGGCTAG

Opossum (*Monodelphis domestica*)

>Md-RNase1

ATGGCTCTGAAGGGCTCCCACCTAATGCTCCTCCTGTTGGGGCTGTTCCTAGCAGAGCTGGTCCACCAATCGACTGGCAAAGAGTCCCC
CTCAAAGAAGTTTCAGCGGCAGCACATGGATCCAGAGAATTCGGCCTCCGATGACACTAAATACTGCGACCACATGATGAAGGCCAGGA
ACATGACCAAAGGGAGATGCAAACCAGTCAATACCTTCATACACGAGCCTAAGGAAATAGTCGATGCCGTCTGCAGGGAGACTAACATC
ACCTGCAAGAATGGGCAGTCCAATTGTTACCAGAGTAGCCAGCCTATGTCCCTCACCCACTGCCGCCAGACTGGAGCCTCCAAATACCC
CAACTGCCAGTACCGGGGTTCTGATATCACTAAACAAATCATTGTGGCCTGTGAGGGAAACCCCTATGTGCCCGTGCATTTTGATGCTT
ATGTGTAG

>Md-RNase4
ATGGCTATGCAGAAGATTGGATCATTGTTTCTGCTGTTGGCCTTGCTTGGCCTATGGTTGGTGCAACCTTCTTCTGGCCAGAATCGGAG
GTACCAACAGTTCTTGAGACAGCATGTGGATCCTTCTAGTGAAGGTGGTGACTCTACTTATTGCAACCAGATGATGCACCGGAGGCGGA
TGACTGAGCCAAGATGCAAGCCTGTTAACAGCTTCATCCATGAGGACATCTGGAACATCAACAGTATCTGCCGTACCACTGACATCAGA
TGCAAGAATGGTCAAATGAACTGCCATGAAGGCATTATGAAGACCACAGACTGCAGAGTGACAGGTGGCTCCACTTCCCCAAACTGTAG
GTACAGGGCGATGACCAGAACCAGGCATGTTGTCATTGCCTGTGAAGATTTGCTGCCTGTGCACTTTGATAGGTAG

>Md-RNase5
ATGGTGATGTTTCTTAAGGCCATGGACCCTTTCTTGCTGCTCTTCATCCTGGGGCTATGGCTGGTTCCACCTTCCTTGACCACACAAGC
AAATGCCAGGGAGAAACACTTCTTGATTCAGCACTATGACTCCAAACCAAAGGGCCGGGATGACAAGTACTGTACAGCGATCATGAGAC
AGAGAGGTTTGACCCAGCCCTGCAAGGATATGAATACCTTCATCCATGGTGATTACCCTAGCATCAAGGCTGTCTGTGGAGATAAAGCT
GGGAATCCCTATGAAGGGGGAAGGCTTAGAATAAGCAAGTCACGATTTCAGGTCACTAACTGTGAGCATCGAGGAGGGTCTACAAAACC
TCCCTGCAAGTACAGGGCCACTTCTGACTTTAGATATATCATCATTGCCTGTGAGAATAATTTACCTGTCCACCTTGACCAGACCATCA
TTGCAAAATAA

>Md-RNase7/8
ATGGCATCAGTTCTGGCAAGTCCGTGCCCTTTATTGCTTCTCTTTCTGGGACTGTGGGAGGTCCCGGTCAGCACAAAGCCCGAGAACCT
GACCCATGCCCAGTGGTTCCAACTTCAACATGTACAGCCTAGCCCTCTCAAGTGCAACAGGGCCATGGGAAGGATTAACTATTACAAAC
AGCACTGTAAGCGCCTTAACACCTTCCTACATGACTCTTTCCCAAATGTGGCCACTGTCTGTGAATCCCCCAGTATGGCCTGTAAGAAT
GGACAGGAGAACTGTCATCAGAGCCCTGGCACGATTTCTATGACCCAATGTGACCTCACCTCTGGGAAATACCCCAACTGCAAGTACAG
CAACACTGCCCTTGACAGAGCCTTCATTGTGGCCTGTGATCCCCCCGAAGCTGGGGATCCCCTTGGGTATCAGCTGGTTCCTGTGCACC
TGGAGCAAACCATTTAG

>Md-RNase12
ATGGAGGCAAAAAGTCTCTTATTCTCCTCTGCCCCTTTTCCTGTAGGAGAGAGAACAAGAAGAAATGTGAATGGGATCTTTCCCCTGTT
GGTGCTATTGTTGCTCACTTTCCTTCTGATGTTGGGTCTGAGTAAAAGTCTGGATGAGGACCTTCAAGAGAAGTCTTTTGAGGAGGAGC
ACATAGACTACCCTAAGTCAAACCGCTTGTTCAGGTACTGTAACTCCATGATGCTGTTGAAAAAAATCCGAGGACCCAATGACACCTGC
AAGCGAAAGCATGTATTCATCCACGAGAGGCTAGACAACATTGTTAGCATGTGCAACAACTTTACTACTATCACTACATGCAAGTACCC
CTCAAGGATGAACTGCCACCACAGCCAGAGGAAGCTCCAGCTGACAGACTGTAAACTCACTGAAGGTAGACGGTTCCCAGGATGCAAGT
ATCAATCTCTCCCCAAGTTCAACTCTATCCTCTTCAACTGTGATGAAATAGGGCCTGTGTACTTATATAAAATTGTGGAGGATTCCTAG

>Md-RNase13
ATGGTTCTGGTCAGGATTCTAGTCCTCCAGCTGCTCTTGGTACCAGCTCTGACCTTCCTCTCCTTCACATCCAAAGAGGCTGAAAATTT
CCGTGCTGTAAACATTGACTTTCCTAAGGTAAATTTTTCCAAAGGCTTCCAGGGCTACTGCAATGGCCTGATGTCTTATGTTCGAGGAA
GGCAAAAGTTATTGAAGAACTGCCCCAATGTCCACTATGTTCTGCATGCTCCCTGGGAAAAAGTGAAGATCAGTTGCCATAAAACTGAC
AGTTTTTGTGAGGACTTCAATGAATATTGCTCCCTCAGCCAGGATGTCTTTCCTATTACCACCTGTAGCCGTGTAAAAGAAACTCCACC
CACCACTTGCCTATACAATGAAACCACTTCCACCCAAAGAGTCTACCTACTTTGCTCCAGAAAATACAATGCTAAACCCATACATATTG
TTGGCCTGTTCTAA

>Md-RNase13ps
AAGACACTTCTGGTCAGGATTCTGGTCCTTCCAGCCACTCCTGGTACCAGCTGTGACCTTCCTCTCCTTCACATCCTCTTCAGTCAAAA
GTTTCCATGCTTTGCACACGGACTATCCTGAGGCTACATTCTCTACAGGTTTTCATGGCTACTTCAGTGTGTCTTGACTGTCCTATGTT
AAAGGAAGGAGAAAGGTGAGGCACGGCCTGAGGGTCCACTATGTGCTTCACACCACTGGACAAGAGTTTCCACCACCCGAAAGGATACT
GACAGCTTTTGTGAAGACTTCTGTGAGTATTGCACCATCAGCCAAGATGCCTTTCCTCTTACCACCTGTAAAAGCATAAAAAACGGGGA
TCTACCCACCACCTGCCAATACAATGAAACCACTTCCATGCAAAGACTCTACCCACTTTGTTCCAGAAAATACAATGCTGACCTCATAT
ATATCATTGGCCTCCTCTAA

>Md-RNase16
ATGATTCCAGAGAGGTCACTCCTTTTGTCCCTGCTGACATTGATGGTCCTGGGGCTGAGCCATCATTCCTTAGCTGAGTCCCAAGAAGA
TAAGTTTAAGAGACAGCATTTGGACTCAGGCACCAAAGGCAATCCTGGGAAAAAATACTGTGACCAGATGATGGCTAAACGGGGCATGA
CAAAAAGGAAATGCAAGCCAGTCAATACCTTTTTGCATGAATCTTACCAGAACATTGAGAATATTTGCCATGAACCAAATGTGCCATGT
GCAAACAAAAATATGCACAATTGTCACAAGAGTAATCACCCCATGAAAATCACTGAGTGCCACCTCTCAGGAGGTTCTAAGCCAGGCAA
ATGCAGATACCGGATGAACAATGTTAAAAAGAATGTCACCGTGGCTTGTGTAGGGAAGACCTTGAAACCAGTACACCTTGATCCTCCAA
AATAG

>Md-RNase17
ATGGCCCTGGACAGAGCCGCCCTGCTCTTCCCACTGCTGGCAATGGTAGTGGTGGGGCTGGTCTCATCAGAGTCTGCAGCAGAGAAGTT
TCAGCGGCAACATGTGGACTCAAAAAGCTTAGCCTTCAATGACAATACTTACTGTAACCACATGATGAAAATCAGGGGTATGACAAAAG
GGAATTGCAAGGTATTCAATACCTTCATATTGGAGTCTATCTGGAAGATCCGGGCTATCTGCTGGAATAAAAAGACCGCATGCAAGCAG
AAATTCTTTAACTGCCACATGAGCCGTGAACCCCTCAAGGTCACTGAATGCCAAATAAAGGGCAACCCCAAGGACTCTCCATGCAAGTA
TCAGACTATAAATGACAAGAAATGTGTCACTGTGGCCTGCAACGGGTGGCCCCCTCTGCCTGTGCATTTTGATTCTTCAAAAGATGATA
TTTGCCAGAGACTTCCCACCACCTAA

>Md-RNase18

ATGACAAGGAGGGAAGAGAACTTTTGGCAGCAACATTACAATGATCAGAGAAGTGACGAAGCGATCCAAAGACAAATGCGGCTGATCAG
CATGGTCAAAGAAAGATGCAAACCCAAAAACACCATCATTCATGATTCACCCGACAAGATCAGGGATATCTGCACCAACCCCAACAGCA
CGAATGTTCGTTGCAGGAATGGAAGCGACAACTGCTATGAAGGTCCAAACCCGTTCTCTGTCACTGTCTGTGAGGATACAGGCTCTATA
TACTCTGTATACCCTAACTGTCAGTACAACTGTCACAAGGAAGAACCTGTCAAAGTCACTGTGGCCTGTGAGAATGGCAAACCTGTCCA
TTTTGATGCTTCCTAA

>Md-RNase19
ATGATTCATACTCTGAAGGGAACTTTTCCACTGTTGCAGTTGACACTTTGGGTGACTATGTTCCTGGTCACTGCAGAACTTCCCTCAAA
TTTCACTCCAGCTCAATGGTTCAATAAGCAGCACGTGCAGCACCCCAAGACCAAAGCCTCCAATGATGACATATATTGCAACAATGAGA
TGAGACAAATCAATAACTATACCCATAGGTGTAAATCTTTCAACACCTTCCTAGACTACATGCTTGAGGATATCATCAATGCTTGTTTC
ACACCCAACATCACCTGTAAGAATCTGCAGAAGAACTGTCATAAAAGTACATTGAAAGTTCCCATTACTAACTGTGTATTGACCAGTGA
TCATTATCCCAACTGCCGTTACCATGGCATTTCTAAAATGGCATTCTTTGTTATTGCCTGTAATCCCCCCTTGCTAGCGGACCATAGCA
AAAGTAAACTACTTCCTGTGCATTTGGACAGCAGTACTGAGGGCCCAGGTCCCATACTTCTGTCTTCTTAA

>Md-RNase20
ATGGCTCAGAATGTGATGGGAAACTCACAATTCTTGCTGCTGAGGCTTTCGTTGATGGTGCTTCTGGTGTACTCACAGCCTCCTCCGGG
TTTCAATTGGGCTCAGTGGTTCAATGAGCAACATGTGCAATACCCCAAGAGCAATGCTCCCAATGACAATCAGTATTGTAATGCAGAGA
TGTACCGGGTGAATAACCACACCAATGTATGCAAAGCCTTTAACAGCTTTCTGCACAACCAGACACAGAATATCATCAATGTTTGCTTG
AATCCCAACACCCCCTGTAAGAATAGTAGGACAAACTGCCATAATAGTACATTCAGAAATGACATCACTGAGTGCACATTGACCAGAGG
CCAATTTCCTAACTGCCAGTACAGAGGCAGGGCTGTAATGAAGTACTTCGTTATTGCCTGTAATAGCATTGTGCCACGTGGTCCATTGC
TTCCTGTGCATTTAGATAATACCTTTTAG

>Md-RNase21
ATGGCTCAGAATGTTATGGGAAACTCACAACTGTTGCTGCTGGGGCTTTCATTATTGGTGCTCCTGGTATATTCACAGACTCGTCCAGG
TTGGGAAAGGTTTCAAAGGTTCAAGAATCAGCATGTGAGATACCCCAAGCACCTTGCTTCCAATAATGATCAGTATTGTATGATGGAAA
TGAAGTGGGTGAATGACTACAACAATGCATGCAAAGGCTTTAACAGTTTTCTGCACAATCAGACACAAAATATCATCAATGTTTGCTTT
CATCCCAATATCTTCTGTAGGAATAATGAGACAAACTGCCATAGAAGCATTTTTAGAAATAGTATCACTGAGTGCAGATCAGCTGGAGG
TCAGTATCCTGATTGCTATTACAATGGCACAGCTGTAAGGAAGTACTTTGTTGTTGCCTGTATTCCCATCAGCCCAAGTGGTCGATTGC
TTCCTGTGCATTTAGACAGCACCTTTTGGAACCCAGGCCAAGTATTTCTCCTCCCACCCCCTTGA

>Md-RNase22
ATGGCTCTTGAGACAATCCATCTGCCTCAGTCCCTCTTGCTGCTGCTGCTGCTCCTGCTGTCATTGCTATTGCCGCCAGGGGTGTGTGA
TATCTATGGAAAGGAGACCAGGATCCAAAGATTCTGGAGGTTTCATGTGGACAATCCAAAGAGCCTCATACCTGGGGGGAATACAAAGT
ATTGCACAGTCATGATTCGGAGGCGTCAGATCTCGGAGAGGGGCAAATGCAAGCGGGAGAACACTTTCATCCATGCCATGGCAGGAGCC
ATTCTGGACGTGTGCAAAGCCCAAAGTGTGAAGGCCTGCAAAGTAGGGAGAAAAAATTGCCACACAAGTGACCATTCCTTCAAACAGAC
CCTATGTGTCCTCCAAGGATTTGGTTATTACCCCAACTGTGCATATGTGAGCTTCCCCAAGTTCAAGAAGATCAGCTTGGCCTGTACAG
GCTTACCCCGGGTTCCTGTGTACGTTGATCCGTAA

>Md-RNase23
ATGGTATATATGGCTCTTAAAATGGCTTTTTCTCTGATGATTCTTCTGATGGCACAACCAATCGCTGGAACAATTGAATATGTAGAAGT
TACTGAAGAGCAGCATATGGGTTTTGGAATTTCTTGGGATCAAAATCCAGCAAGAAATTTCCAAATAAAGCACATCACTGATGGATACT
TTACAAACTGTACTGTCATGATGAATAGAGTTAATAGAAAAGGTTCTTGCAAATCAATCAATACTTTTATTCATAGTCATAAAAATGAA
GTAAATAAAGTGTGTAAGGAAAAAGGAACTTTTATAGACTGCAAACAAAATCGTAATTATAAATGTTGCTTTATAAGTAAGATTAAATT
TAATTTGACTATCTGTGAGGAAAAGGAATCAAGGAAATCATGTGAATATCATGCTTTAAAAAAGGAAGCACATGTGGTGCTTGGTTGCA
ACAACAATAATCAACCTGTTCACTTTCAAGGTTATTTTTGTGAAACCAAACCCTACAGAGTGTATATGGAGAAAAGAAATCTGAATGAC
CACTTACCTTCAACCTATATGTCTGGAGCTCAAGAGGAATGA

>Md-RNase24
ATGTATGCTGGGATGTGGACATTTTTTTTGGCCTTATTCTTGGACCTGGCCAATTTTTCCTACACCAATGACTTCTGGAGGCGACATGT
GGACTATCCCAGGTCTACTCCCAAAGAAGAAGACAATTACTGTAACTGGATGATGGAACGGAGGGGGATGGTTTGGGGCAATAAATGTG
TACCAGGTAACACCTTCATCCATGAGAAAAACTCCACCATTGAAAACATGTGCAATCGAACTCCTAGGCCCTGTAAAAATCACCGTAAC
CAATTCTGTCACACCAGTGTTCGTTCATTCTCTGTGTCAGATTGTTTTCTTGAGGGTGGTAGTCAGCCTCCTTATTGCAAATACTGGGT
ATTGGTTCAGTCTCGGAAGATTAGTGTCTTTTGTCAGTATGAACTTCCTTTCCACTTGGAAGACTAA

>Md-RNase25
ATGGTTCATGCTGGAATGAAGACTCTTTTCTTGCTCCTCCTCCTGGACCTGACTAACTTCTCCCAGGCTTTCCACTTCTGGAATTACCA
CGTGGACTATCCCAAGACCCAGGTCTCAGGAACACCAGATCAATATTGTAATGTGATAATGCAACGGAGGGGGATGATTACACGGAATC
ACTGCTTTCAAATTAATGCGTTCATCCACGAGACAAACAGCACTCTCCAAAATATCTGTAAAACTAGCCCAAAGACTTGTGGAGGTCCC
AGTTGGTTACTATGTCATGAAAGTTCCAAACCATTGAAGTTGACATATTGCTCTACACAGTCTTATGCCCGGCCCCCTAACTGTGGGTA
TAAGGCACAGGCCTTTTCTGAAAAGATCTTGGTAGTTTGTGTACGTGGATCCCCTATCTACTTGTTCCAAACCTAA

>Md-RNase26
ATGTCTCAAGCTGTGATGTGGACTGTTTTCTTGCTCATCTTTCTGGATTTGATTCACTTCTCCCAGACTCAAGACTTCTGGACTCACCA
TGTAGATTTCCCCAAGACCCACGTCTTTGATAACCCATCTCAGTACTGTGATTTGATGATAATGAATAAAGGGCTGGTTAACAATAGTG
ACTGCAAGCCATTTAACTCCTTCATCCACGAGTCAAATAAAACCATCTTCAATATTTGTGCTGATTCATATGGCTCCTTCCAAGATCCC
AATGAACTAAACTGCTATGAAAGTACCTACCCACTTAGGATTACAGATTGTTTTGCCAGATGCATTAGTAACTGTTATCCTAGGTGTAG
TTATGTGGAAATATTCCATTTAGCAAAAATTAGGGTAGCATGTGTGAATGGACGACCACAACGCATAATTAGACAGATTTATTGTCCCC
CCTTCTGGATCAGATATTGGGTCCCATATGGGTACTACACCTACCCATGA

>Md-RNase27ps

ATGTTATTCTCATACGGTGGGAGCATGTATTGGGAGAAGTGCTAATATCAGCAAAGGAACAGACATTTTGGCAGGAGCATCACAATGAA
GGGAGCAGTAGCCAAGAGATCCAAAGACAAATGAGGCAGATCAACTTGGGCAAAAGCAGATGCAAACCCATCAATACCATCATTCATGG
CCCACGTGAAGAGATCCAGGATATCTGCACTAACTCCAACAGCATCAATGTTCCTTGCAAGAATAGACAAGGCAACTGCTTTGGATGTC
CAACCTTATACATCCATGATGAACTGTGATTGCCTTGAAAACCTCTCATGAAATATGATCCCCTCCTCCAGTGTGATCAGCAATAGCTG
TCATGTTGGCCATGCTCATTTTTTTCCTTCTTAATATGCAATATTAATACCAGCCTTGAGACTTAACTGACCCTAGATCTAG

>Md-RNase28
ATGGCTTGTGCTGTGATATGGATCTTTTTCCTGGTCTCCTTCATGCACCTTGCTAATGTTTCCCAGTCCCAGGATTTCTGGACTCGCCA
TGTGGATTACCCCAGGACCAATATCTCTAGAATCCCATCCAAATATTGTAATGGGATCATGAAGCAGAGGGGGTTAGAGATACATAATT
CCTGCGTGTATTTCACCACTTTCATCCATGAGATGAATCAAACCATTCTCAATATCTGTGCAAATTCCAATGTGCACCACCAATGTCCC
AATGGATTAACCTGTCATATCAGTCCCTACCCAATTATGGTGACAGATTGTTTTATGAAATTAGATGCCAATTTCCCTGACTGTAGATA
TAGAGGTAAAATCTATTTTACAAGAATCAAGGTAATTTGTGTGAATGGAAGACCAAAGTATGTGTTTAGTCAGGCTTATCTTCCTCCCT
TCTAG

>Md-RNase29
ATGTGGATCCTTTTCCTGGTCTCCTTCATGCACCTTGCTAATGTTTCCCAGTCCCAGAGCTTCTGGACTCATCATGTGGATTACCCCAG
GACCAATATCTCTGGAAGCCCATCCAAGTACTGTAAAATGATGATAAGGCAGAGGGAATTGAAGATGCATGATTCCTGCCTCTATTTCC
ACACTTTCATCCATGAGACAAATCAGACCTTGCTCAATATCTGTGCAAATTCTAATGTACACCATGAATGTCTCAATAGATTAACCTGT
CACATAAGCCACTACCCACTTATGGTGACAGATTGTTTTAAGATAGCCCATCCCAATGTCCCTGACTGTAGATATAGAGGTAAAATCTA
TTTTACGAGAATCAAGGTAATTTGTGTGAAAGGAAGGCCGGAGTACCTGATTCATTTTCCTGACTTGAAAGAATGA

>Md-RNase30
ATGTCTTCTGCGGTGAAGTGGACCGTTTTCTTGCTCATCATTCTGAACCTGACTCACTTCTACCAGGCCAAAGACTTCTGGACTCTCCA
TGTGGACTATCCCAAGACCAAAGCCTCGGGAAAACCACCCGAGTACTGCAATAATATTATGAGGCAAAGGGGGCTCATTAACAATACTT
ACTTAAGGCCTTTCCATACCTTCATCCATGAGTCAAACAGTACCATCCTCAATATCTGTGCTAACTCTACCAACCCCCACTATGCTTCC
TTTGCACCAAACTCTTATGAAAGCCAAAATCCCCTTCACCTGACAGAATGTTGTATCAAGACCTGTAGCCCTAATTTTGTTTGTACTTA
TAAGGAAGTAATCAGAATGTCAAAAATTAGATTAGCTTGTTTAAATGGACACCCAGACCACTTAATTACCTACATTAATTATTCTCCCA
CAAAGATTCTAATTAATCTCCTATTTGGGGGCCCCCGTCGCTGA

>Md-RNase32
ATGGATTGTGCTGTGATGTGGATCCTTTTCCTGGTCTCCTTCATGCACCTTGCTAATGTTTCCCAGTCCCAAGACTTCTGGAAGCAGCA
TGTGGATTATCCCAGGACTGAAGTCTCAGGAGGCCCATCCAAATACTGTAATGCCATGGTGAAGCAGAGGGGTTTAGAGATGCAGAATT
CCTGCCTGTATTCCAACATTTTCATCCATGAGACAAACCACAGCATTGTCAATATTTGTGAAAATACCAATCATTGCTTTCCATCATTC
AAAGGATTACCCTGTCATAGAAGCCTCTACCCAGTTTTGGTGACACATTGTTTTATTAGGTTACATTCTGTTTTCCCTCACTGTAGATA
TCGGGGGGAAATCTATTTTGCAAGAATCAAAATTATTTGTATGAATGGAAAACCAGGCTTCATAATCTGA

Platypusm (*Ornithorhynchus anatinus*)

>Oa-RNase4
ATGTTTTGGCAGGGGACCTACTCACTGCTGCTCCTGGCACTGCTGGCACTGCTTGGACCAGCCCTGCTGCCTGTTTCAGGGCAGAAGAC
CATGTACCAGCGGTTCCTGCGCCAGCATGTGGACCCAGAGGGTGAAAATGGCGGCGACGCCACAGCCTACTGCAACCGCATGATGCAGG
CGAGGAAGATGACCGCTCAGCGCTGCAAGGCCTTCAACACCTTCATCCACGAGCCCATCTACAAAATCAACAGCATCTGTAGCACAGAC
AGCATCTCATGCAAGAACGGCCGCATGAATTGCCACGAGGGTGTCGTCCGGGTAACCGACTGCAGAGAGAAGGGGGGCTCCAGGCCAGG
CAACTGTAGGTATCGGGCCCAGGTCAGCACCCGCCGTGTGGTCATTGCCTGTGAGGGGGACCCTCAAGTGCCCGTGCACTTTGACCGCT
GA

>OaRNase13
ATGGCACCAGCTCCCCCCAGGACCTCCACGCTGTTCCCGCTATTCTTCATCCTCGCCCTGGACCCCGTGCCGGCCCCGGCTTTGCTCCC
CTCCCTGTCGTCGAAGGCCCGAACCTTCCGGCTGATGCATATTGATTTTCCCAGGTCCGAGTTCGCACCCGGGTTCCGGGGCTACTGCA
ACGGCCTGATGGCTTATGTACGCGCGCAGCGTGAGAGCTGGCAGTGCCCGGAGAAGCACTTTGTGCTGCACGCGCCCGCGGCCACCGTG
CGCGCCATCTGCGCGCACACTGACTCCTTCTGCGAAGACTTTGGCGAGTTCTGCACGCGCAGTCACAAGCCCCTTCCAGTGACCACATG
CGCCCGCACCCCTGGGCTGCCCCCCTCGGTCTGCCGCTACAATGCCACCGTGCATGTCCAGTCCCACAGAGTCTGGCTACTCTGCTCCA
GCAAGTTCGAGGGCTTCCCGATGGATGTCATTGGTGTCTCTTAG

>Oa-RNase33
ATGGCAGCCACCACCAAGACCCCCGGCCACATGCTGCCCCAGGTGCTGCCCCTGGCCTTGCTACTCCTGGCCACCTGCACCTTCGGTCA
GTTCGCAGTCTTCCTACAGCGCCATGTTGACCAGCCCAAGTCACAGGTCCCAGGAGGGCCGAGGCAATACTGCAATGTGCTGATGCAGC
GCCGCAGGCTCAACACGGGGAACCACTGCAAACCCCTCAACACCTTCATCCACGAGAACCAGGGAGCTCTGGTCGCCCTGTGCCGCACC
CCGGCCGGGCCATGCCGCAACCCAAAGAAGAAAAACTGTCACCGCAGCCCCCAGAGACTGAATGTCACCGACTGCAAGGTCATCCCCGG
TAGCCAGCCCCCCAATTGCCGGTACCACTCCTTAGCCCAGTCCCGCAACATCGTTGTGGCCTGTGTTGGGGGGCAGCCTGTCCATCTCG
ATGGGTAG

>Oa-RNase34
ATGGTAGCCACCACCATGACCCCTGGCCGCATGCTGCCCCAGGTGCTGCCCCTGGCCTTGCTACTGCTAGCCACCTGCACCTTCGGTCA
GTTCGCAGTCTTCCTACAGCGCCATGTTGACCAGCCCAAGTCGCAGGTCCCAGGAGGGTCGAGGCAATACTGCAATGTGCTGATGCAGC
GCCGCAGGCTCAACACGGGGAACCGCTGCAAACCCCTCAACACCTTCATCCACGAGAACCAGGGAGCTCTGGTCGCCCTGTGCCGCACC
CCGGCCAGGAGGTGCCGCAACCCGCGGATGCACAACTGTCACCGCAGCCCCCAGCGTCTGAGAGTCACCGACTGCAGGGCCATCCCCGG
TGGCCAGCCCCCCGGATGCCGTTACCGCTCCTTAGCCCGGTCCCGCAACATCGTTGTGGCCTGTGTTGGGGGGCAGCCTGTCCATCTCG
ATGGGTAG

>Oa-RNase35

ATGGTGCTGATCCTGATGGGGACCACCCACCCTCTGCCCCTCGTCGCCTTGTTCCTCCTCCTCCACCTGACTCTCCCAAGTCCACCCTT
GATCGCGACCTTTGACGATTTTTACAAGTGCCATATAGTTGAGGAAGGGGCAAATATCCAAGACCTTGACAAGTTCTGCACGGATAAGA
TGATGGAAAGCAAATGCAGTAAATACAAGAAAGTCAACACTTTCATCAGTGAGGGAGTTAAGACAGTGATTGATATCTGCAAGGAAAAG
CGCGATTATTGCCGTAATCCAAAATGGAAAAACTGCCACAACAGCAGCCAAGAACTTGAACTCATCAACTGCTGTCCAAACACCTTCAA
AGATGGCACCTTCCTCTACCAGGGCTTCATGAAGAGAAGAAAAATTTCAGTAGCCTGTGTTTGTCACCAGCCTGTTCACTTTGACGCAC
AGTTTCCTTATGACTCACTCTGTAACAGCAATTACTCATACCTTCCTGAGGCCCCTCCCACTTAA

Other miscellaneous sequences

The large flying fox (megabat, Pteropus vampyrus)

>Pv-RNase1
ATGGCTCAGGCGAAGTCCCTGATCTTGTTCTCACTGCTACTCCTAGTGCTGCTGGCACTGGGGTGGGTTGAACCTTTCCGGGAAAGGGA
ATCACAGGCCAAGAAGTTCATTCGGCAGCACATGGACCCAGACAGCTACCCCAACATCGGCTCCAGCTACTGTAACCAAATGATGAGGC
GCCGGGACATGACGAAAGGACGGTGCAAGCCAGTGAACACCTTTGTGCACGAGCCCTTGGTTGATGTCCAGGCTGTTTGCTTCCAGGAA
AAGGTCACCTGTAAGGATGGGCAGTCTGACTGCTACCAGAGCAGCTCCAGCATGAGCATTACGGACTGCCGCCTGACAAATGGCTCCAA
ATACCCCAATTGTGCATACCGAACCAGCCAGAAAAAGAGGCACATCATCGTGGCCTGTAACGGGAACCCATACGTGCCGGTCCACTTTG
ACGCTTCAGTAGAGGTCTCCACCTGA

>Pv-RNase4
ATGGCTCTCCAGAGGATCCTTTCATTGCTTCTGCTCTTGTTGCTGACCCTCCTAGGGCTGGGGCTGCTGCAGCCCTCCTATGGCCAGGA
TCACATGTACCAACGATTCTTGCGGCAACATGTGGACTCTAAGGTGACAAATCGCAATGAAAGCTACTGCAACTTGTTGATGCAAAGAC
GGAAGATGACTTCGCGTTATTGCAAGTACTTCAACACCTTCATCCATGAGGACATCTGGAAAATTATTAATATCTGCAGCACCACCAAT
ATCCAGTGCAGGACTGGTGAGATGAACTGCCATGAGAGTGGAGTGTAGTGAATGCCACAGACTGCAGGCTGATAGCAGGTTCCAAGGCC
CCCAAGTGCAGATATCGGGCCATGACTGGTACTAGACAGGTTGTCATTGCCCGTGACGGTAATCCGGCGCTGCCTATGCACTTTGCCAG
ATAG

>Pv-RNase5
ATGGTGATGGGCCTAGGACCCCTATTATTGGTCTTCATGATGGGTCTGGGTCTAACCCTGCCCACCCTGGCTCAAAATAACTACAGGTA
CGAACTCTTCCTGACCCAGCACTGTGATGCCAATCCAAGTGGCCGAGATAAGAAATACTGTGAAAGCATGATGGCGAAACGAGGCATGA
CTACACCCTGCAAAGATCGCAACACCTTTATTCATGCCAGCAAAAAAGAAATTAAGGCTGTCTGTGAAGATAAGAATGGAACACCTTAC
AATGGAGGACTCAGGAAAAGCAAGACTCCCTTCCAGGTCACCATTTGCAAGCATACGGGAGGATCCCCCCGGCCTCCCTGCTATTACAA
AGCCACATCAGGGTCCAGAGACATTGTTATTGCCTGTGAAAAAGGCTGGCCTGTCCACTTTGATGAGTCCTTCTACCGTCCATAA

>Pv-RNase6
ATGATGCTTGATCTCCTGGAACGTTTATTTCTTCTCCTATTGCTGCTGGGACTATGGGGACCAATGTGTCCACTTTATGCTTTGCCTAG
GAATCTCACCAAGGCTCGCTGGTTTAAAATTCAGCATATACAGCCACTCTATCTTCAATGCGACACGGCAATGAGTGGTGTCAATACTT
ATACTCAGGTCTGTAAGCCTCAAAACACCTTTCTGCACGACTCCTTCCAGAACGTGGCTATTGCCTGTATGTCACCTAACATCTTCTGC
AAGAATGGTCGGAAGAATTGCCACCAGAGTACAAAGCGTGTGTATATGACCAACTGCAATCTCACTGGAGGGACGTATCCTGCCTGCCG
CTACAAAGAGGCTAACCAGACCAAGTTCTTCATTATTGCCTGTGACCCCCCTCAAAAGGGTGACCCTCCCTATCAGTTGGTTCCTGTAC
ACTTAGATAAGGTTATTTAA

Tarsier (*Tarsius syrichta*)

>Ts-RNase2/3A
ATGGTTCCAAAACTGCTCGATTCTCGACTTTGTCTGCTTCTGCTTCTGGGACTCATGGGAATGGAGGGCTCATTCCATGTCAAACCCAG
CCAGTTTACCTGGGCTCAGTGGTTTACCATCCAGCATATAAATATGACCCACACCCGATGCGACGACGCAATGCGAGTAGTTAACGGTT
ATTTAAGGCGATGCAAAAATCGCAATACGTTTCTTAGAACAACTTTTGACGATACAGCTGGTGTCTGTGGTACCCCAAATATAACCTGT
CCTAGTAACAATACTATGAAAAATTGTCATCAAAGCCCAGATCAGGTGAATATAGTTGACTGTAACCTCACAAAAAGTTCCAAAAATAT
TACAAACTGCCTATATGCACAGTCATCAGCACAGAAGTACTACGTGATTGCTTGTGCCAACAGAACTAACCAGGAGCCTAGCAAGTATC
CACTGATTCCAGTTCACTTGGATAGAATCTTCTAA

>Ts-RNase2/3B
ATGATTCCAAAACAGCTCGATTCCCGATTTTGTCTGCTTCTGCTGCTGGGGCTCATGAAAATGATAGGCTCACTCTGTGCCGCACCTGG
TAACTTAACCCGGGCTCAGTGGTTTACCATTCAGCACATAAACATGACCCACCCCAAATGTGATGACGCAATGCGGGTAGTGAACGGCT
ATACAGGACGATGCAAAAACCAAAATACTTTTCTGAATACAACTTTTCCTAATGTAGTTAATGTTTGTCTTACTCCAGCTATAAAATGC
ATTACAAGCAAAAGTCAAAATTGTCATAAAAGCTCAGTCAAGGTGAATTTAACCTACTGTAACCTCACAACTCCCGGGACTGTTCAAAC
CTGCAAATATGCACAGACAGAGGCAGAGATGTTCTACATCGTTGCTTGTGACAACAGATCTGCACTGGACCCTCCCATATATCCCGTGG
TTCCGGTTCACCTGGATGCAACAGTAATGCTCCTGACCCAGGACTTGGTGCCAACTCTCAACAGTCAAGCTTCACAATCTAGAAGCCTC
TTGAGGCATGTTTTTGACACTGTGTGGTCATTTGGTCACTCTCTGTTGCAGAGACTTTTCCCTTCTTCTACCTGA

>Ts-RNase2/3C
ATGGCTCCAAAACTGCTCAGTTCCCGATTTTGTCTGCTTCTGCTTCTGGGGCTCATGGGAATTGTGGGCTCATCCCATGCCAAACCCAG
TCATGTAACCTGGGCTCAGTGGTTTAATATCCAGCATGTCAATATGACCCACTCCCGATGCGACGATGCCATGCAGGTAGTTAATGGTT
ATGAGAAACGATGCAAAAACCTGAATACTTTTCTCCACACAAATTTCTCTTCTGTGGTTAGTGTTTGTAACACTCAAAATATCACCTGT
AATAATGGCCGCATGAACTGTCATGAAAGTACAGAGCAGGTGCCTTTAACCATCTGTAACATCACAAGACACTCGACCAATTATAGAAA
CTGCACCTATAGACAGACACAAGCACAAAAGCGCTACATTATTGCCTGCAACAACAGTGCTCCACAGGACGCTGAATATCCAGCAATGG
TTCCAGTTCACTTAGATCGACTTACCTAA

>Ts-RNase2/3D

ATGATTCCAAAACAGCTCGATTCCCGATTTTGTCTGCTTCTGCTGCTGGGGCTCATGGAAGTGATAGGCTCACTCTGTACCCCAGCTGG
TAATTTAACCCAGGCTCAGTGGTTTACCATTCAGCACATAAACATGACCCACCCCCAATGTGATGATGTAATGCAGGTAGTGAACAGCT
ATACAAGACGATGCAAAAACCGAAATACTTTTCTGAATTCAATTTTTCCTAATGTAGTTGATGTTTGTCTTACTCCAGCTATAAAATGC
ATTACAAGCCAAAGTCAAAACTGTCATAATAGCTCAGTCAAGGTGAATTTAACCTACTGTAACCTCACAACTCCAAGGACTGTTCAAAC
CTGCAGATATGCACAGACAGAGGCAGAGATGTTCTACATCGTTGCTTGTGACAACAGATCTGCACTGGACCCTCATATATACCGTGGTT
CCAGTTCACCTGGATGCAACAGTAATGCTCCTGACCCGGGACTTGGTGCCAACTCTCAACAGCCAAGCTTCACAATCTAG

>Ts-RNase7/8A
ATGGCACCAGTCAGAGCAGGATTCTGCCCCCTGCTGCTCCTGCTGCTGGGGCTATGGGTGGCCATGATCTCAGTCAGTGCCAAGCCCGG
GCATGTGACCGACGCTGGGTGGTTTAAAACTCAGCACGTGCAGAACAAACGTCGATCATGCAAAGCCGCCATGGAGAACATCAACAAGC
ACACAAAACATTGCAAAGACCGCAACACCTTCCTGCAAACAACCTTCTCCAAGGTGGCCGCCACCTGCAGGACCCCCAGTATAGCCTGC
AAGGATGGCCAGAAAAACTGCCACAAGAGCCGCGGGGCTGTTTCCATGACCTTGTGTGAAGTTACCTCAGGGAAGTACCCAAATTGCAA
GTACAAGGAGAATCATCTGAAGAAACGTTACATAGTGGCCTGTAACCCTCCTCAGAAAGGGGACTCTAAGAAATTCAAGCTGGTTCCCG
TGCACTTGGACAACATCCTTTAG

>Ts-RNase7/8B
ATGGTGCCAGTCAGAGCCCTATCCTGCCCCCTGTTGCTGTTCCTGCTGCTGGGGCTGTGGGTGGCCAAGATCCCAATCAATGTCAAGCC
CACGAACACTACCTCAGCCCAGTGGTTTGAAATTCAGCATGTGCAGCCTAGCCCTCAAGCATGCAACTCAGCCATGGGCAAAATCAACA
AGGACAAGAAAAATTGCAAAAACCTCAACACCTTCCTGCATGAATCCTTTTCCAGCGTGGCCACCACCTGCCAGACCCCCAGTATAGCC
TGCAAGAACGGTCGTAAAAACTGCCACAAGAGCCAAGAGCCCATGTCCCTGACCCAGTGTGAGTATACCTCAGGGAGGTACCCCGACTG
CAAATACAAAGGGAAACAACTGGATGCATTCTTCATTGTGGCCTGTGAACCTCCACAAGAGGGTGACAAGAAGTATTCCCTTGTTCCTG
TGCACTTGGATGATGTTGTCTAA

Bushbaby (*Otolemur garnettii*)

>Og-RNase2/3A
ATGGTTCCAAAGCTGCTCAATTCCCGATTCTGTCTGCTTCTGCCGCTGGTGCTCATGGCGATGGTGGGCTCCTTCCATGCCAAACCTCC
TCAGTTTACCTGGGCTCAGTGGTTTGAGATCCAGCACATAAATATGACCAACCCCCAATGCACTATTGCAATGCGGGTAATTAACCGTT
ACTATCCACAATGTAAAAGGAGGAATACTTTTCTTCATACAACCTTTGCTGCTGTAGCTGGTGTTTGTGGTACCCCAAATACACCCTGC
GTTTCAAACAGCAGTAGAACAAATTGTCATAACAGTTCAGTCCCGGTGGCTATAACCTATTGTAACCTCACAAGTCGACCAACTCCTGT
TGCAAACTGCAGATATACAAACACAGCGGCACAGAGTCTCTATGTAGTTGCTTGTAACAACAGGGATCCACAGCGGGACTCTCTGCTGT
ATCCAGTGGTTCCAGTTCACTTGGATGCAATCTAA

>Og-RNase2/3B
ATGATTCCAAAGCTACTGGATTCCCAACTTTGTCTGCTTCTGCTGCTGGGGCTCGTGATGATGGTGGGCTCCTTCGATGCCCCACCTGG
TAATTTCACCCCAACTCTGTGGTTTCAGATCCAGCATGTAAATATGACCAACCCCCAATGCACTATTGCAATGCGGTCAGTTAACCATT
ACAAACCACAGTGCAAACCCAGGAATACTTTTCTTCATACAACCTATCCTGATGTAGTTAAGGTTTGTGGTACCCCAAATATACCCTGC
CCTACAAACCCCACAAGAAAAAATTGTCACAACAGTTCAGTCCCGGTGTCTTTAACCTATTGTAACCTCACAAGTCGACCGACTCCTGT
TGCAAACTGCAGATATTCACAGACGTCGGCACAGATGTTCTATGTAGTTGCTTGTGACAATAGAGATCCACGGGACTCGCCCCTGTATC
CAGTGGTTCCAGTTCACCTGGATGCCATAATGGCAATAATCCTGGATCAGCATGATGTGCCAAGCCTCGCGCACCAAGTGCCACCATCT
AGGAGACTCTGTGTGTCTACTTTTGCCACTCTTCTGTTATTTAGTTACAATCTGTTCTAG

>Og-RNase2/3C
ATGGTTCCAAAGCTGCTCGATTCCCAACTCTGTCTGATTCTGCTGCTGGGGTTCGTGGCAATGGTGGGCTCCTTCGATGCCCCACCTGG
TAATTTCACCCCAACTCAGTGGTTTCGGATCCAGCATGTAAATATGACCAACCCCCGGTGCACTATTGCAATGCGGTCAGTTAACCATT
ACAAACCACAGTGCAAACCCAGGAATACTTTTCTTAATACAACCTATCCTGATGTAGTTAAGGTTTGTGGTACCCCAAATATACCCTGC
CCTACAAACCCCACAAGAAAAAATTGTCACAAGAGTTCAGTCCCAGTGCGTTTAACCGATTGTCACCTCACAAGTCGACCGACCCCTGT
TAAAGACTGCAAATATTCAGACAAGCCAGAAGAGAAGTTCTATGTAGTTGCTTGTAACAACAGAGATCCACGGGACTCGCCCCTGTATC
CAGTGGTTCCGGTTCACCTGGATGCCATAGTGTCACCGACCCTGGATCAGCATGATGTGCCAAGCCTCGCGCACCAAGTGCCACCATCT
AGGAGACTCTGTGTGTCTACTTTTGCCACTCTTCTGTTATTTAGTTACAATCTGTTCTAG

>Og-RNase7/8
ATGGCACCAGCCAGAACAGGATTCTGCCCTTTGCTGCTGCTGCTACTCCTGCTGCTGGGGCTGTGGGTGGCCAAGGTCCCAGTCAGTGC
CAAGCCCAGGCACATGACTTCATCCCAGTGGTTTGAAATTCAGCACGTGCAGCCCCACCCTCGGGCTTGCAACTCAGCAATGGGCAACA
TCAACAAGCACACAAAACATTGCAAAGACCGCAACACCTTCCTGCATGAATCCTTCTCCCGTGTGGCCACCACCTGCCAGACCCCCAAA
ATAGCCTGTAAGAATGGCCATAAGAACTGCCACCAGAGCCGTGGGCCCGTGTCCCTAACCTTGTGTGAGCGCACCTCAGGGAAGTACCC
AAACTGCAGGTACAAAGAGAAGCACCTGAACAAGTCTTACATAGTGGCCTGTGACTCTCCCCAAAAAAGAGACTCTCAGCCATTCCCAC
TGGTTCCGGTGCACTTGGACAAAGTCCTTTAG

Tree shrew (*Tupaia belangeri*)

>Tb-RNase7/8
ATGGCACCCGTCAGACTGGGATTCTGCCACTTGCTGCTGCTCCTGCTGCTGGGGCTGTGGGTAGCCGAGATCCCAGTGAGTGCCAAGCC
CAAGCACGTGACATCGTCTCAGTGGTTTAAAATCCAGCACGTGCAGCCCAGCCCTGAAGCATGCAACTCAGCAATGGGCAAGATCAACA
AGGACACAAAACGGTGTAAAAATCTCAACACCTTCCTGCACAAATCCTTCTCCGATGTGGCCGCCACCTGCCAGAACCCCGTCAAGACC
TGCAAGAACAAACGTAAAAACTGCCACAAGAGTCGCGGGCCTGTGTCCCTGACCCTGTGTGAGCATACCTCGGGGAAGTACCCAAACTG
CAGGTACAAAGAGAAGCACATGAAGGCATCTTACACGGTGGCCTGTGACCCTCCCCAGAAAGGGGACACTAGGAAATTCCGCCTGGTGC
CTGTGCACTTGGAACCTCTTTAG

PROTEIN SEQUENCES

Human (*Homo sapiens*)

>Hs-RNase1
MALEKSLVRLLLLVLILLVLGWVQPSLGKESRAKKFQRQHMDSDSSPSSSSTYCNQMMRRRNMTQGRCKPVNTFVHEPLVDVQNVCFQE
KVTCKNGQGNCYKSNSSMHITDCRLTNGSRYPNCAYRTSPKERHIIVACEGSPYVPVHFDASVEDST*

>Hs-RNase2
MVPKLFTSQICLLLLLGLLAVEGSLHVKPPQFTWAQWFETQHINMTSQQCTNAMQVINNYQRRCKNQNTFLLTTFANVVNVCGNPNMTC
PSNKTRKNCHHSGSQVPLIHCNLTTPSPQNISNCRYAQTPANMFYIVACDNRDQRRDPPQYPVVPVHLDRII*

>Hs-RNase3
MVPKLFTSQICLLLLLGLMGVEGSLHARPPQFTRAQWFAIQHISLNPPRCTIAMRAINNYRWRCKNQNTFLRTTFANVVNVCGNQSIRC
PHNRTLNNCHRSRFRVPLLHCDLINPGAQNISNCTYADRPGRRFYVVACDNRDPRDSPRYPVVPVHLDTTI*

>Hs-RNase4
MALQRTHSLLLLLLLTLLGLGLVQPSYGQDGMYQRFLRQHVHPEETGGSDRYCNLMMQRRKMTLYHCKRFNTFIHEDIWNIRSICSTTN
IQCKNGKMNCHEGVVKVTDCRDTGSSRAPNCRYRAIASTRRVVIACEGNPQVPVHFDG*

>Hs-RNase5
MVMGLGVLLLVFVLGLGLTPPTLAQDNSRYTHFLTQHYDAKPQGRDDRYCESIMRRRGLTSPCKDINTFIHGNKRSIKAICENKNGNPH
RENLRISKSSFQVTTCKLHGGSPWPPCQYRATAGFRNVVVACENGLPVHLDQSIFRRP*

>Hs-RNase6
MVLCFPLLLLLLVLWGPVCPLHAWPKRLTKAHWFEIQHIQPSPLQCNRAMSGINNYTQHCKHQNTFLHDSFQNVAAVCDLLSIVCKNRR
HNCHQSSKPVNMTDCRLTSGKYPQCRYSAAAQYKFFIVACDPPQKSDPPYKLVPVHLDSIL*

>Hs-RNase7
MAPARAGFCPLLLLLLLGLWVAEIPVSAKPKGMTSSQWFKIQHMQPSPQACNSAMKNINKHTKRCKDLNTFLHEPFSSVAATCQTPKIA
CKNGDKNCHQSHGAVSLTMCKLTSGKHPNCRYKEKRQNKSYVVACKPPQKKDSQQFHLVPVHLDRVL*

>Hs-RNase8
MAPARAGCCPLLLLLLGLWVAEVLVRAKPKDMTSSQWFKTQHVQPSPQACNSAMSIINKYTERCKDLNTFLHEPFSSVAITCQTPNIAC
KNSCKNCHQSHGPMSLTMGELTSGKYPNCRYKEKHLNTPYIVACDPPQQGDPGYPLVPVHLDKVV*

>Hs-RNase9
MMRTLITTHPLPLLLLPQQLLQLVQFQEVDTDFDFPEEDKKEEFEECLEKFFSTGPARPPTKEKVKRRVLIEPGMPLNHIEYCNHEIMG
KNVYYKHRWVAEHYFLLMQYDELQKICYNRFVPCKNGIRKCNRSKGLVEGVYCNLTEAFEIPACKYESLYRKGYVLITCSWQNEMQKRI
PHTINDLVEPPEHRSFLSEDGVFVISP*

>Hs-RNase10
MKLNLVQIFFMLLMLLLGLGMGLGLGLHMATAVLEESDQPLNEFWSSDSQDKAEATEEGDGTQTTETLVLSNKEVVQPGWPEDPILGED
EVGGNKMLRASALFQSNKDYLRLDQTDRECNDMMAHKMKEPSQSCIAQYAFIHEDLNTVKAVCNSPVIACELKGGKCHKSSRPFDLTLC
ELSQPDQVTPNCNYLTSVIKKHIIITCNDMKRQLPTGQ

>Hs-RNase11
METFPLLLLSLGLVLAEASESTMKIIKEEFTDEEMQYDMAKSGQEKQTIEILMNPILLVKNTSLSMSKDDMSSTLLTFRSLHYNDPKGN
SSGNDKECCNDMTVWRKVSEANGSCKWSNNFIRSSTEVMRRVHRAPSCKFVQNPGISCCESLELENTVCQFTTGKQFPRCQYHSVTSLE
KILTVLTGHSLMSWLVCGSKL*

>Hs-RNase12
MIIMVIIFLVLLFWENEVNDEAVMSTLEHLHVDYPQNDVPVPARYCNHMIIQRVIREPDHTCKKEHVFIHERPRKINGICISPKKVACQ
NLSAIFCFQSETKFKMTVCQLIEGTRYPACRYHYSPTEGFVLVTCDDLRPDSFLGYVK*

>Hs-RNase13
MAPAVTRLLFLQLVLGPTLVMDIKMQIGSRNFYTLSIDYPRVNYPKGFRGYCNGLMSYMRGKMQNSDCPKIHYVIHAPWKAIQKFCKYS
DSFCENYNEYCTLTQDSLPITVCSLSHQQPPTSCYYNSTLTNQKLYLLCSRKYEADPIGIAGLYSGI*

Chimpanzee (*Pan troglodytes*)

>Pt-RNase1
MALEKSLVLLPLLVLILLVLGWVQPSLGKESRAKKFQRQHVDSDSSPSSSSTYCNQMMRRRNMTQGRCKPVNTFVHEPLVDVQNVCFQE
KVTCKNGQGNCYKSNSSMHITDCRLTNGSRYPNCAYRTSPKERHIIVACEGSPYVPVHFDASVEDST*

>Pt-RNase2
MVPKLFTSQICLLLLLGLLAVEGSLHVKPPQFTWAQWFETQHINMTSQQCTNAMQVINNYQRRCKNQNTFLLTTFANVVNVCGNPNMTC
PSNKTRKNCHQSGSQVPLIHCNLTTPSPQNISNCRYAQTPANMFYIVACDNRDQRRDPPQYPVVPVHLDRII*

>Pt-RNase3
MVPKLFTSQICLLLLLGLMGVEGSLHARPPQFTRAQWFAIQHISLNPPRCTIAMRVINNYRWRCKNQNTFLRTTFANVVNVCGNQSIRC

PHNRTLNNCHQSRFRVPLLHCDLINPGAQNISNCGYADRPGRRFYVVACDNRDPRDSPRYPVVPVHLDTTI*

>Pt-RNase4
MALQRTHSLLLLLLLTLLGLGLVQPSYGQDGMYQRFLRQHVHPEETGGSDRYCNLMMQRRKMTLYHCKRFNTFIHEDIWNIRSICSTTN
IQCKNGKMNCHEGVVKVTDCRDTGSSRAPNCRYRAMASTRRVVIACEGNPQVPVHFDG*

>Pt-RNase5
MVMGLGVLLLVFVLGLGLTPPTLAQDNSRYTHFLTQHYDAKPQGRDHRYCESIMRRRGLTSPCKDINTFIHGNKRSIKAICENKNGNPH
RENLRISKSSFQVTTCKLHGGSPWPPCQYRATAGFRNVVVACENGLPVHLDQSIFRRP*

>Pt-RNase6
MVLCFPLLLLLLVLWGPVCPLHAWPKRLTKAHWFEIQHIQPSPLQCNRAMSGINNYAQHCKHQNTFLHDSFQNVAAVCDLLSIVCKNRR
HNCHQSSKPVNMTDCRLTSGKYPQCRYSAAAQYKFFIVACDPPQKSDPPYKLVPVHLDSIL*

>Pt-RNase7
MAPARAGFCPLLLLLLLGLWVAEIPVSAKPKGMTSSQWFKIQHVQPSPQACNSAMKNINKHTKRCKDLNTFLHEPFSSVAATCQTPKIA
CKNGDKNCHQSHGPVSLTMCKLTSGKYPNCRYKEKRQNKSYVVACKPPQKKDSQQFHLVPVHLDRVL*

>Pt-RNase8
MAPARAGCCPLLLLLLLLLGLWVAEVLVSAKPKDMTSSQWFKTQHVQPGPQACNSAISNINKYTERCKDLNTFLHEPFSSVAITCQTPN
IACKNSCKNCHQSHGPMSLTMGELTSGKYPNCRYKEKHLNTPYMVACDPPQQGDPGYPLVPVHLDKVV*

>Pt-RNase9
MMRTLITTHPLPLLLLPQQLLQPVQFQEVDTDFDFPEEDKKEDFEEYLEQFFSTGPTRPPTKEKVKRRVLIESGMPLNHIEYCTHEIMG
KNVYYKHRCVAERYFLLMQYDELQKICYNRFVPCKNGIRKCNRSKGLVEGVYCNLTEAFEIPACKYESLYRKGYVLITCSWQNEMQKRI
PHTINDLVEPPEHRSFLSEDGVFVIPP*

>Pt-RNase10
MKLNLVQIFFMLLMLLLGLGMGLGLGLHMAAAVLEESDQPLNEFWSSDSQDKAEATEEGDDIQTTETLVLSNKEVVQPGWPEDPILGED
EVGGNKMLRASALFQSNKDYLRLDQTDRECNDMMAHKMKEPNQSCIAQYAFIHEDLNTVKAVCNSPVIACDLKGGKCHKSSRPFDLTLC
ELSKPDQVTPNCNYLTSVIKKHIIITCNDMKRQLPTGQ*

>Pt-RNase11
METFPLLLLSLGLVLAEASESTMKIIKEEFTDEEMQYDMAKSGQEKQTIEILMNPILLVKNTSLSMSKDDMSSSLLTFRSLHYNDPKGN
SSGNDKECCNDMTVWRKVSEANGWCKWSNNFIHSSTEVMRRVHRAPSCKFVQNPGISCCESPELENTVCQFTTGKQFPRCQYHSVTSLE
KILTVLTGHSLMSWLVCGSKL*

>Pt-RNase12
MIIMVIIFLVLLFWENEVNDEAVMSTLEHLHVDYPQNDVPVPARYCNHMIIQRVIREPDHTCKKEHVFIHERPRKINGICISPKKVACQ
NLSAIFCFQSKTKFKMTVCQLIEGTRYPACRYHYSPTEGFVLVTCDDLRPDSFLGYVK*

>Pt-RNase13
MAPAVTRLLFLQLVLGPTLVMDIKMQIGSRNFYTLSIDYPRVNYPKGFRGYCNGLMSYMRGKMQNSDCPKIHYVIHAPWKAIQKFCKYS
DSFCENYNEYCTLTQDSLPITVCSLSHQQPPTSCYYNSTLTNQKLYLLCSRKYEADPIGIAGLYSGI*

Gorilla (*Gorilla Gorilla*)

>Gg-RNase1
MALEKSLVLLPLLVLILLVLGWVQPSLGKESRAKKFQRQHMDSDSSPSSNSTYCNQMMRRRNMTQGRCKPVNTFVHEPLVDVQNVCFQE
KVTCKNGQGNCYKSNSSMHITDCRLTNGSRYPNCAYRTSPKERHIIVACEGNPYVPVHFDASVEDST*

>Gg-RNase2
MVPKLFTSQICLLLLLGLLAVEGSLHVKPPQFTWAQWFETQHINMTSQQCTNAMRVINNYQRRCKNQNTFLLTTFANVVNVCGNPNMTC
PSNKTRKNCHHSGSQVPLIHCNLTTPSPQNISNCRYAQTPANMFYIVACDNRDQRRDPPQYPVVPVHLDRII*

>Gg-RNase3
MVPKLFTSQICLLLLLGLMGVEGSLHARPPQFTRAQWFAIQHISLNPPRCTIAMRVINNYRWRCKNQNTFLRTTFANVVNVCGNQSIRC
LHNRTLNNCHRSRFRVPLLHCDLINPGAQNISNCRYADRPGRRFYVVACDNRDPQDSPRYPVVPVHLDTTI*

>Gg-RNase4
MALQRTRSLLLLLLLTLLGLGLVQPSYGQDGMYQRFLRQHVHPEETGGSDRYCNLMMQRRKMTLYHCKRFNTFIHEDIWNIRSICSTTN
IQCKNGKMNCHEGVVKVTDCRDTGSSRAPNCRYRAMASTRRVVIACEGNPQVPVHFDS*

>Gg-RNase5
MVMGLGVLLLIFVLGLGLTPPTLAQDNSRYTHFLTQHYDAKPQGRDDRYCESIMRRRGLTSPCKDINTFIHGNKRSIKAICENKNGNPH
RENLRISKSSFQVTTCKLHGGSPWPPCQYRATAGFRNVVVACENGLPVHLDQSIFRRP*

>Gg-RNase6
MVLRFPLLLLLLVLWGPVCPLHAWPKRLTKAHWFEIQHIQPSPLQCNRAMIGINNYTQHCKHQNTFLHDSFQNVAAVCDLLSIVCKNRR
HNCHQSSKPVNMTDCRLTSGKYPQCRYSAAAQYKFFIVACDPPQKSDPPYKLVPVHLDSIL*

>Gg-RNase7
MAPARAGFCPLLLLLLLGLWVAEIPVSAKPKGMTSSQWFKIQHVQPSPQACNSAMKNINKHTKRCKDLNTFLHEPFSSVAATCQTPKIA
CKNGDKNCHQSHGPVSLTMCKLTSGKYPNCRYKEKRQNKSYVVACKPPQKKDSQQFHLVPVHLDRVL*

>Gg-RNase9
MMRTLITIHPLPLLLLLQQLLQPVQFQEVDTDFDFPEEDKKEEFEEYSEQFFSIGPTRPPTKEKVKRRVLIEPGMPLNHIEYCNHEIMG
KNVYYKHRCVAEHYFLLMQYDELQKICYNRFVPCKNGIRKCNRSKGLVEGVYCNLTEAFEIPACKYESLYRKGYVLITCSWQNEMQKLI
PHTINDLVEPPEHRSFLSEDGVFVIPP*

>Gg-RNase10
MKLNLVQIFFMLLMLLLGLGMGLGLGLHMAAAVLEESDQPLNEFWSSDSQDKAEATGEGDGTQTTETLVLTNKEVVQPGWPEDPILGED
EVGGNKMLRASALFQSDKDYLRLDQTDRECNDMMAHKMKEPNQSCIAQYAFIHEDLNTVKAVCNSPVIACELKGGKCHKSSRPFDLTLC
ELSKPDQVTPNCNYLTSVIKKHIIITCNDMKRQLPTGQ*

>Gg-RNase11
METFPLLLLSLGLVLVEASESTMKIIKEEFTDEEMQYDMAKSGQEKQTIEILMNPILLVKNTSLSMSKDDMSSSLLTLRSLHYNDPKGN
SSGNDKECCNDMTVWRKGSEASGSCKWSNNFIHGSTEVMRRVHRAPSCKFVQNPGISCCESPELENTVCQLTTGKQFPRCQYHSVTSLE
KILTVLTGHSLMSWLVCGSKL*

>Gg-RNase12
MIIMVIIFLVLLFWENEVNDEAVMSTLEHLHVDYPRNDVPVPARYCNHMIIQRVIREPDHTCKKEHVFIHERPRKINGICISPKKVACQ
NLSAIFCFQSETKFKMTVCQLIEGTRYPACRYHYSPTEGFVLVTCDDLRPDSFLGYVK*

>Gg-RNase13
MAPAVTRLLFLQLVLGPTLVMDIKMQIGSRNFYTLSIDYPRVNYPKGFRGYCNGLMSYMRGKMQNSDCPKIHYVIHAPWKAIQKFCKYS
DSFCENYNEYCILTQDSFPITVCSLSHQQPPTSCYYNSTLTNQKLYLLCSRKYEADPIGIAGLYSGI*

Orangutan (*Pongo pygmaeus*)

>Pp-RNase1
MALEKSLVLLPLLVLILLVLGWVQPSLGKESRAKKFQRQHMDSGSSPNSNSTYCNQMMRRRNMTQGRCKPVNTFVHEPLVDVQNVCFQE
KVTCKNGQGNCYKSNSSMHITDCRLTHGSRYPNCAYRTSPKERHIIVACEGSPYVPVHFDASVEDST*

>Pp-RNase2
MVSKLFTSQICLLLLLGLLAVDGSLHVKPPQFTWAQWFETQHINMTSQQCNNAMQVINNFQRRCKNQNTFLRTTFANVVNVCGNPNITC
PSNRSRNNCHHSGVQVPLIHCNLTTPSPQNISNCRYAQTPANMFYIVACDNRDPRRDPPQYPVVPVHLDRII*

>Pp-RNase3
MVPKLFTSQICLLLLLGLSGVGGSLHAKPRQFTRAQWFAIQHVSLNPPQCATAMRVINNYQRRCKDQNTFLRTTFANVVNVCGNPNITC
PSNRSRNNCHHSGVQVPFIYCNLTTPSPQNISNCSYANITGRRFYIVACDNRDPRDSPQYPVVPVHLDTII*

>Pp-RNase4
MALQRTHSLLLLLLLTLLGLGLVQPSYGQDGMYQRFLRQHVHPEETGGNDRYCNLMMQRRKMTLYHCKRFNTFIHEDIWNIRSICSTTN
IQCKNGKTNCHEGVVKVTDCRDTGSSRAPNCRYRAMASTRRVVIACEGNPQVPVHFDG*

>Pp-RNase5
MVMGLGVLLLVFMLGLGLTPPTLAQDNSRYTDFLAQHYDPKPQGRDDRYCESIMRRRGLTSPCKGINTFIHGSKRSIKAICENKNGNPH
RENLRISKSSFQVTTCKLHGGSPWPPCHYRATADFRNIVVACENGLPVHLDQSIFRRL*

>Pp-RNase6
MMLRFPLLLLLLVLWGPVCPLHAWPKRLTKAHWFEIQHIQPSPLQCNRAMSGINNYTQHCKHQNTFLHDSFQNVAAVCDLLSIVCKNHR
HNCHQSSKPVNMTDCRLISGKYPQCRYSAAAQYKFFIVACDPPQKSDPRYKLVPVHLDSIV*

>Pp-RNase7
MAPARAGFCPLLLLLLLGLWVAEIPVSAKPKGLTSSQWFKIQHVQPSPQACNSAMKNINKHTKRCKDLNTFLHEPFSSVAATCQTPKKA
CKNGEKNCHQSHGPVSLTMCKLTSGKYPNCRYKEKRQNKSYIVACKPPQKNDSQKFHLVPVHLDRVL*

>Pp-RNase8
MAPARAGWCAVVMLLLGLWVAEIPVSAKPKDMTSSQWFKTQHMQPSPQACNSAMNNINKYTEQCKDLNTFLHELFSSVATTCQTPNIAC
KNSRKNCHQSHGPMSLTMCELTSGKYPNFRYKEKHLNAPYIAACDPPQQGDPGYPLVPVHLDKVV*

>Pp-RNase9
MMRTLITTHPLPLLLLLQQLLQPVQFQEVDTDYDLPEDKREEFEEYVEQFFSTGPTRPPTKEKVKRLLLIEPGMPLYHVDYCNSEIMRK
NVYYKHRCVAEHYFLLMQYDELQKICYNRFVPCKNGVRKCNRSKGLVEGVYCNLTEAFRIPACKYESFYRKGYVLITCAWQNEMQKLIP
HTINDLVEPPEHRSFLSEDGVFVILP*

>Pp-RNase10
MKLNLVQIFFMLLMLLLGLGMGLGLGLHMAAAVLEESNQPLNEFWSSDSQDKAEATEEGEGTQTTETLVLSNKEVVQPGWPEDPILGED
EVGGNKMPRASALFQSNKDYLRLDQTDRECNDMMAHKMKEPNQSCIAQYAFIHEDLNTVKAVCNSPVIACELKGGKCHKSSRPFDLTLC
ELSKPDQVTPNCNYLTSVIKKHIIITCNDVKRQLPTGQ*

>Pp-RNase11
METVPLLLLSLGLVLAEASESTMKIIKEEFIEEEMQYDMAKSGQEKQTIEILMNPIQLVKNTSLSMSKDDMSSSLLTFRRLHYSGPKGD
SSGNDKECCNDMTVWRKVSEANRSCKWNNNFIHGSTEVMRRVHKAPSCKFVQNPGISCCESPELENTVCQLTTGKQFPRCQYHSVTSLE
KILTVLTGHSLMSWLVCGSKL*

>Pp-RNase12
MIIMVIIFLVLLFWENEVNDEVVMSTLEHLHVDYPQNDVPVPARYCNHMIIQRVIREPDHTCKKEHVFIHERPRKINSICISPKKVACQ
NVSAIFCFQSETKFKMTVCQLIEGTRYPACRYHYSPTEGFVLVTCDDLRPDSFLGYVK*

>Pp-RNase13
MAPAVTRLLFLQLVLGPTLVVDIKMQIGSRNFYTLSIDYPRVTYPKGFRGYCNGLMSYMRGTMQNSDCPEIHYVIHAPWKAIQKFCKYS
DSFCENYNEYCTLTQDSFPITVCSLSHQQPPTSCYYNSTLTNQKLYLLCSRRYEADPIGIAGLYSGI*

Gibbon (*Nomascus leucogenys*)

>Nl-RNase1
MALEKSLVLLPLFVLMLLVLGWVQPSLGKESRAKKFQRQHMDSDSSPSSNSTYCNQMMRRRNMTQGRCKPVNTFVHEPLVDVQNVCFQE
KVTCKNGQANCYKSNSSMHITDCRLTNGSRYPNCAYRTSPKERHIIVACEGSPYVPVHFDASVEDST*

>Nl-RNase2
MVPKPFTSQICLLLLLGLMGVEGSLHAKPRQFTWAQWFEIQHINMTSQQCTNAMRVINNYQRRCKNQNTFLRTTFANVVNVCGNPNMTC
PSNKTRKNCHQSGSQVPLIHCNLTTPSPQNISNCGYAQTPANMFYIVACDNRDQRRDPPQYPVVPVHLDRII*

>Nl-RNase4
MALARTHSFLLLLLLTLLGLGLVQPSYGQDGMYQRFLRQHVHPEETGGNDRYCNLMMQRRKMTLYHCKRFNTFIHEDIWNIRSICSTTN
IQCKNGKMNCHEGVVKVTDCRDTGSSKAPNCRYRAMASIRPVVIACEGNPQVPVHFDG*

>Nl-RNase6
MVLRFPLLLLLLVPWGPVCLLHAWPKHLTKAHWFEIQHIQPSPLQCNRAMSGINNYTQHCKHQNTFLHDSFQNVAAVCDLLSIVCKNRW
HNCHQSSKPVNMTDCRLTSGKYPQCRYSAAAQHKFFIVACDPPQKSDPPYKLVPVHLDSIV*

>Nl-RNase7
MAPARAGFCPLLLLLLLGLWVAEIPVNAKPKGMTSSQWFKIQHVQPSPQACNSAMKNINKHTKRCKNVNTFLQEPFSSVATTCQTPKIA
CKNGDKNCHQSRGPVSLTMCELTSGKYPNCRYKEKRQNKSYIVACKPPQKKDSQQFHLVPVHLDRVL*

>NlRNase9
MMRTLITTHPLPLLLLLQQLLQPVQFQEVDTDFDFPEDKKEEFEEYLEQFFSTGPTRPPTKEKVKRRVLIEPGMPLDHIDYCNSEIMRK
NVYYKHRCVAEHYFLLMQYDELQNICYNRFVPCKNGIRKCNRSKGLVEGVYCNLTEAFEIPACKYESFYRKGYVLITCAWQNELQKLIP
HTINDLVEPTEHRSFLGEDGVFVIPP*

>Nl-RNase10
MKLNLVQIFFMLLMLLLGLGMGLGLGLHMAAAVLEESDQPLNEFWSSDSQDKAEATEEGEGTQTTETLVLGNKEVVQPGWPEDPILSED
EVGGNKMLRASALFQSNKDYLRLDPTDRECNDMMAHKMKEPNQSCIAQYAFIHEDLNTVKAVCNSPVIACELKGGKCHKSSRPFDLTLC
ELSKPDQVTPNCNYLTSVIKKHIIISCNDMKHQLPTGQ*

>Nl-RNase11
METFPLLLLSLGLVLAEASESTMKIIKEEFTEKEMQYDMAKSGQEKQTIEILMNPVPLVKNTSLSMSKDDMSSSLLTFRRLHYNDPKGN
SSGNDKECCTVWRKVSEANGSCKWSNNFIHGSTEVMRRVHKAPSCKFVQNPGISCCESPKLENTVCQLTTGKQFPRCQYHSVTSLEKIL
TVLTGHSLMSWLVCGSKL*

>Nl-RNase12
MIIMVIIFLVLLFWENEVNDEVVMSTLEHLHVDYPQNDVPVPARYCNHMIIQRVIREPDHTCKKEHVFIHERPRKINGICISPKKVACQ
NLSAIFCFQSETKFKMTVCQLIEGTRYPACRYHYSPTDGFVLVTCDDLRPDSFLGYVK*

>Nl-RNase13
MAPAVTRLLFLQLVLGPTLVMDIKMQIGSRNFYTLSIDYPRANYPKGFRGYCNGLMSYMRGKMQNSDCPKIHYMIHAPWKAIQKFCKYS
DSFCENYNEYCTLTEDSFPITVCSLSHQQPPTSCYYNSTLTNQKLYLLCSRKYEADPIGIAGLYSGI*

Rhesus monkey (*Macaca mulatta*)

>Mmu-RNase1
MALDKSVILLPLLVLVLLVLGCLGRESRAKKFQRQHMDSGSSPSSNSTYCNQMMKRRSMTHGRCKPVNTFVHEPLVDVQNVCFQEKVTC
KNGQTNCFKSKSSMHITDCRLTNGSRYPNCAYRTSPKERHIIVACEGSPHVPVHFDASVEDST*

>Mmu-RNase2
MVPKLFTSPICLLLLLGLMGVEGSLHAKPGQFTWAQWFEIQHINMTSGQCTNAMLVINNYQRRCKNQNTFLLTTFADVVHVCGNPSMPC
PSNTSLNNCHHSGVQVPLIHCNLTTPSRRISNCRYTQTTANKYYIVACNNSDPVRDPPQYPVVPVHLDRVI*

>Mmu-RNase3A
MVPKLFTSPICLLLLLGLMGVEGSLHARPPQFTKAQWFAIQHINVNPPRCTIAMRVINNYQRRCKNQNTFLRTTFANTVNVCRNRSIRC
PHNRTLHNCHRSSYRVPLLHCDLINPGAQNISTCRYADRPGRRFYVVACESRDPRDSPRYPVVPVHLDTII*

>Mmu-RNase3B
MVPKLFTSQICLLLLLGLMGVEGSLHARPPEFTRAQWFAIQHINMNPPSCNIAMRVINKYQRCCEKQNTFLRTTFANVVNVCRNPSIPC
VHNRTLQNCHHSSVQVPLLQCEHINRGAQNVSTCEYADRQGQSFYVVACENRDPRDPPQYPVVPVLLDRII*

>Mmu-RNase4
MALQRTHSLLLLLLLTLLGLGLVQPSYGQNGMYQRFLRQHVHPEETGGNDRYCNMMMQRRKMTLYHCKRFNTFIHEDIWNIRSICSTTN
IQCKNGKMNCHEGVVKVTDCRDTGSSKAPNCRYRAMASTRRVVIACEGNPQVPVHFDG*

>Mmu-RNase5
MVMGLGLFLLVFMLGLGLTPPTLAQDNPRYRDFLAKHYDATPQGRNDRYCESTMRRRHLTSPCKDINTFVHGNRHHITAICGDENGSPY
GGNLRISTSPFQVTTCKLRGGSPRPPCQYRATRGSRNIVVGCENGLPVHLDESIFRP*

>Mmu-RNase6
MVLRFPLLLLLLVLWGPVCLLHAWPKHLTRAHWFEIQHIQPSPLQCNRAMSGINNYTQHCKHQNTFLHDSFQNVAAVCDLLSIICKNRQ
HNCHQSSKPVNMTDCRLTSGKYPQCRYSAAAQYKFFIVACDPPQKSDPPYKLVPVHLDSIV*

>Mmu-RNase7
MAPARAGFCPLLLLLLLGLWVAEIPVSAKPKGMTPSQWFQIQHVQPSPQACNSAMKNINKHTKRCKDLNTFLHEPFSRVATTCQTSNKA
CKNGDKNCHQSHGAVSLTMCQLTSGKYPNCRYKEKRQNKSYIVACKPPQKKDSQQFHLVPVHLDRVL*

>Mmu-RNase8
MAPARAGCCPLLLLLLGLWVAEIPVSAKSKDVTSSQWFKTQHVQPSPEACYSAMSNISKYTEWCKDLNTFLHEPFSGVATTCQTPNIAC
KNRHKNCPQSSGPVSLTMCELTSGKYPNCRYKKKHLNAPYIVACDPPQQGDPGYPLVLCTWIKLS

>Mmu-RNase9A
MMRTLITTHPLLLLLLLQQLLQPVQFQEVDTDFDSPEDKMEEFREYLEEFRRTGPTRPPTKEKVERRVIIEPGMPLYHRDYCNEEIMRK
NVYHKQRCVTEHYFLLMQYDELEKICYNRFVPCKNGVRKCNRSKGLVEGVYCNLTEAFKIPRCKYKSFYRRGYVLITCAWQNEIHKLIP
HTINDLVEPPKHRSFLNEDGVFVILP*

>Mmu-RNase9B
MMRTPITTYPLLLLLLLQQLLQPVQFQEVDTDFDSPDDDMEEFEEYLEEFHRTGPTRPPTKENVKRRVIIEPGMPLYDREYCNAEIMRK
NVYHKQRCVTEHYFLLMQYDELEKICYNRFVPCKNGVRKCNRSKGLVEGVYCNLTEAYEIPWCRYKSFYRRGYVLITCAWQNEIHKLIP
HTINDLVEPPKHRSFLNEDGVFVIPP*

>Mmu-RNase10
MKLNLVQILFMLLMLMLGLGMGLGLGLQMAAAVLEESDQPLNEFWSSDSQDETEATEEGDGTQTTETLVLGNKEVVQPGWPEDPILNED
EVGGNKMLRASALFQSNKDYLRLDQTDRECNEMMAHKMKEPNQSCIAQYAFIHEDLKTVKAVCNSPVIACELKGGKCHKSSRPFDLTLC
ELSKPDQVTPNCNYLTSVIKKHIIITCNDVKLQLPTG*

>Mmu-RNase11
METFPLLLLSLGLVLAEASESTMKIIKEEFTEEEMQYDMAKSGQGIQTIEILMNSILLVKNTSLSMSKDDMSSSLLTFRRLHYNDPKGN
SSGNDKECCNDMIVWRKVSEANRSCKWSNNFIHDSTEVMHGVHKAPSCKFVQNPGISCRESPELENTVCQLTADKQLPRCQYHSVTSLE
KILTVLTGHSLMSWLVCGSKL*

>Mmu-RNase12
MIIMVIIFLVLLFWENEVNDEVVTSTLEHLHVDYPQNDVPVPARYCTHMIIQRVIREPDHTCKKEHVFIHERPRKINGICTSPKKVACQ
NLSAIFCFQSETKFKMTVCQLTEGTRYPACRYHYFTTEGFVLVTCDDLRPDTFLGYVK*

>Mmu-RNase13
MAPAVTRLLFLQLVLGPTLVMDIKTQIGKKKFYALNVDYPRVIFPKSFKGYCNGLMSYMRGKVQNSDCPKIHYVIHAPWKVIKRFCKYS
DNFCDNYNEYCTLTEDSFPITVCSLNYQQPPTSCYYDSTLTNQKLYLLCSRKYEADPIGIIGLYSGV*

Marmoset (*Callithrix jacchus*)

>Cj-RNase1
MALEKSLALLPLLVLVLLVLGWAKPSLGKESRAEKFQRQHMDSDGSPSSNPTYCNNMMRRRNMTQGRCKPVNTFVHEPLVDVQNVCFQE
KVTCKNGQPNCYKSSSSMRITDCRLTNGSRYPNCAYRTSQKERHIIVACEGNPYVPVHFDASVEGST*

>Cj-RNase2/3
MVPKLFTSQICLLLLLGLLSAEGSPHARPQQYSRAQWFSIQHIQTAPLHCTSAMRAINKYQSRCKNKNTFLHTTFADVVNVCGNTNMTC
PHNASLNNCHHSGVQVPLTYCNLTGPQTISNCVYSSTQANKFYVVACENRDPRDPPQYPVVPVHLDTII*

>Cj-RNase4
MALQRTHSLLLLLLLTLVGLGLVQPSYGQDRMYQRFLRQHVHPEEIGGNDGYCNLMMQRRKMTSNHCKRFNTFIHEDIWNIHSICSTAN
IQCKNGKMNCHEGVVKVTDCRETGSSRVPNCRYRATASTRRVVIACEGNPGVPVHFDS*

>Cj-RNase5
MVMGLHLLLLVFILGLGLTPPTLAQNDIRYIRFLEEHYDPKTRNGNDRYCEKMMRLRNMISPCKGTNTFIHGNKESIKAICGTENGESY
NGNKRISKSAFQVTICKHRGGSPRPPCQYRATAGFRNVVVACENGLPVHLDESIFRP*

>Cj-RNase6
MVQHFPLLLLLLVLWGQVCPLHAIPKNLTKAQWFEIQHIRPSPLQCNRAMSGINNYTQHCKPQNTFLHDSFQNVTAVCELLSITCKNGL
HNCHQSLKPVNMTDCRLTSGNYPQCRYSTAAKYKFFIIACDPPQKDDPPYKLVPVHLDRIF*

>Cj-RNase7
MVPARAGYFPLLLLLLLGLWVAEIPVSVKPKGMTPSQWFNTQHVQPSPQACASAMRNINKHTKRCKDLNTFLHKPFSSVAATCQTPNIT
CKNGHKNCHQSHGPVSLTMCRLTSGKYPNCRYKEKHLNKSYIVACDPRQKKDPQQFHLVPVHLDRVL*

>Cj-RNase8
MALAREGCCPLLLLLLLELWVTEIPVSAKQKGMTPSQWFKTQHVQPSPQACDLAMRNINNYKKWCKDLNTFLHEPFSSVAATCQTPNIT
CKNGHKNCHQSHGPVSLTMCGLTSGKYLNCRYKEEHQNKSYIVACDPLQQGDPEYPLVPVHLDKVV*

>Cj-RNase9
MRMLITTYSLPLLLLLLQPLQFQEVYYEDYYLPAYRSTEGFEDFWVEFHSTGPTRPPSKEKVKRRILVNPGMPLGDSGYCNYQIMRKNV
YYKHSCVTEHYFLLMQYDELEKTCYNGFVPCKNGIRKCNRSKNLVEGVYCNLTEASDIPMCRYESFYRRGYVLITCTWQNEIQKLIPYT
INDLVEPPKHKSP**

>Cj-RNase10
MKLNLVQVFFMLLLLLLGLGMGLGLGLRMAAAVLEESDQPLNEFWSSDSQDKAEATEAGESTQTTETLVLSNKEVVQPGWPEDPILNED
EVGEKEMLRAESLFRNNKDYLRVDQTDRECNDMMAHKIKEHNQSCITQHAFIHEDPDTVKAVCNSPVIACELKGGKCHKSSRPFDLTFC
ELSKPDQVTPNCNYLTSVIKKHIIITCNDKKLQSSIGQ*

>Cj-RNase12
MVIMVIIFLVLLFWENEMNNEVVISTLEHLHVDYPQEGVPVPARYCNHMIIQRIIREPDHTCKKEHVFIHERPRKINGLCTSPKKVACQ
NLSSTFCFQSETKFKMTVCQLIEGTRYPACRYRYFPVEGFVLITCDELGPDSFQGYIK*

Mouse (*Mus musculus*)

>Mm-RNase1
MGLEKSLILFPLFVLLLGWVQPSLGRESAAQKFQRQHMDPDGSSINSPTYCNQMMKRRDMTNGSCKPVNTFVHEPLADVQAVCSQENVT
CKNRKSNCYKSSSALHITDCHLKGNSKYPNCDYKTTQYQKHIIVACEGNPYVPVHFDATV*

>Mm-Ear1
MGPKLLESRLCLLLLLGLVLMLASCLGQTPSQKFAIQHINNNTNLQCNVEMMRINRARRTCKGLNTFLHTSFANAVGVCGNPSGLCSDK
RSQNCHNSSSRVHITVCNITSRATNYTQCRYQSRRSLEYYTVACDPRTPQDSPMYPVVPVHLDGTF*

>Mm-Ear2
MGPKLLESRLCLLLLLGLVLMLASCLGQTPSQWFAIQHINNNANLQCNVEMQRINRFRRTCKGLNTFLHTSFANAVGVCGNPSGLCSDN
ISRNCHNSSSRVRITVCNITSRRRTPYTQCRYQPRRSLEYYTVACNPRTPQDSPMYPVVPVHLDGTF*

>Mm-Ear5
MGLKLLESRLCLLLLLGLVLTLVSCQRPTPSQKFDIQHIYKKSSPKCDDAMRVVNKYTGKCKDLNTFLHTTFADVVRVCHNPPKTCKDG
TSPNCHDSSSKVSVTICKLTKRARNYTHCRYKTTGAKKSYTVACNPRTPKDRPTYPVVPVHLDRLF*

>Mm-Ear6
MGPKLLESQLCLLLMLGLVLMLASCQKPTASQWFATQHITYKANLQCNVEMQAINMHRPRCKGLNTFLHTSFINVVGVCSNPSGLCSDK
ISQNCHNSSSRVPITVCNLTTPRRNYTQCRYQTKGSVEYYTVACEPRVAWDCPIYPVVPVHLDGTF*

>Mm-Ear10
MGPKLLESRICLLLLLGLVLMLASCLGQTTSQWFAIQHINNNANLQCNVEMQRINRFRRTCKGLNTFLHTSFANAVGVCGNPSGLCSDN
ISQNCHNSSYRVHITVCNITSWRRTPYTQCRYQAKRSLEYYTVACDPRTPQDSPMYPVVPVHLDGTF*

>Mm-Ear11
MGLEQLESRLCLLLLLGHVLMLASCQPLTPSRWFDIQHIYNRAYPRCDDAMRAVNSYTGVCKDINTFLHTTFANVVRVCHNPRKICKNG
ISRNCHDSSNRVQVTICILTTPASHYSNCRYRTTRSMKYYTVACDPRTPQDSPMYPVVPVHLDGIF*

>Mm-Ear14
MKLPESSLCLLLLLGLVLMLASCQAQILSQKFYTEYIYNSTYPRCDAVMRVVNRYRPRCKDINTFLHTSFADVVAVCGHPNITCNNLTR
KNCHASSFQVFITFCNLTTPTRICTQCRYQTTGSVKYYRVACENRTPQDTPIYPVVPVHLDGTF*

>Mm-RNase4
MMDLQRTQSLLLLLVLTLLGLGLVQPSYGQDRMYQRFLRQHVDPQVTGGNDNYCNVMMQRRKMTSVQCKRFNTFIHEDIWNIRGICSTT
NILCKNGQMNCHEGVVKVTDCRETGNSKAPNCRYRARTSTRRVVIACEGDPEVPVHFDR*

>Mm-Ang1
MAISPGPLFLIFVLGLVVIPPTLAQDDSRYTKFLTQHHDAKPKGRDDRYCERMMKRRSLTSPCKDVNTFIHGNKSNIKAICGANGSPYR
ENLRMSKSPFQVTTCKHTGGSPRPPCQYRASAGFRHVVIACENGLPVHFDESFFSL*

>Mm-Ang2
MAMSPGPLFLVFLLGLVVIPPTLSQDDSRYTKFLTQHYDAKPKGRDDRYCESMMVKRKLTSFCKDVNTFIHDTKNNIKAICGKKGSPYG
RNLRISKSHFQVTTCTHKGRSPRPPCRYRASKGFRYIIIGCENGWPVHFDESFISP*

>Mm-Ang4
MTMSPCPLLLVFVLGLVVIPPTLAQNERYEKFLRQHYDAKPNGRDDRYCESMMKERKLTSPCKDVNTFIHGTKKNIRAICGKKGSPYGE
NFRISNSPFQITTCTHSGASPRPPCGYRAFKDFRYIVIACEDGWPVHFDESFISP*

>Mm-Ang5
MVISPGSLLLVFLLSLDVIPPTLAQDNYRYKNFLNQHYDAKPTGRDYRYCESMMKKRKLTSPCKEVNTFIHDTKNNIKAICGENGRPYG
VNLRISNSRFQITTCKHKGGSPKPPCQYKAFKDFRYIVIACEDGWPVHFDESFISM*

>Mm-Ang6
MVMSPGSLMLVFVLSLVVISPTLAKDNYRYIKFLTQHYDAKPTGWDHRYCYLCMMKKRIQETLKCKEANTFIHDTKKNIKAICGENGGP
YGANFRISNSPFQITTCNHSGGSPKPPCQYRDFKDFRYIVIACEDAWPVHFDESFISL*

>Mm-RNase6
MVVDLPRYLPLLLLLELWEPMYLLCSQPKGLSRAHWFEIQHVQTSRQPCNTAMRGVNNYTQHCKQINTFLHESFQNVAATCSLHNITCK
NGRKNCHESAEPVKMTDCSHTGGAYPNCRYSSDKQYKFFIVACEHPKKEDPPYQLVPVHLDKIV*

>Mm-RNase9
MKPLVIKFAWPLPLLLLLLLPPKLQGNYWDFGEYELNPEVRDFIREYESTGPTKPPTVKRIIEMITIGDQPFNDYDYCNTELRTKQIHY
KGRCYPEHYIAGVPYGELVKACDGEEVQCKNGVKSCRRSMNLIEGVRCVLETGQQMTNCTYKTILMIGYPVVSCQWDEETKIFIPDHIY
NMSLPK*

>Mm-RNase10
MKVTLVHLLFMMLLLLLGLGLGLGLGLHMAAAVLEDHPLNEFWPSDSQNTEEGEGIWTTEGLTLGYKEMAQPVWPEEAVLSEDEVGGSR
MLRAEPRFQSKQDYLKFDLSVRDCNTMMAHKIKEPNQSSINQYTFIHEDPNTVKAVCNGSLVDCDLKGGKCYKSPRPFDLTLCKLAKPG
QVTPNCHYLTYITEKVIFMTCNDKKQLETK*

>Mm-RNase11
MAVFLLLLALGLLLAKPSESRMKGTTEQFSQEEMQPAAKQTLEESANSTLSDKNTGLSISKHVMSATPLTPRRLSFIIPKGNTMRDGRN
CVNSLRVWRTEVDGNASCQLGNDFIHGSMDVSLRIPKATRGKCEQTPKPSSSGSLGLERTTCKVLAGHQCLRSHEHSITSLKKILTVLA
SNSLMSWLVSGCKL*

>Mm-RNase12
MVLMVVVFLLLLFWENELTEDVVLTSIEQLHVDYPQNAVPLRYCNYMILQRVIREPDHRCRKVHVFIHERPQKINRVCTSSKKMSCPND
SDLFCFQSETKFRMTVCQLIDGTTYPACRYQISPIKGFVLVTCDDLGPVDLQGYVE*

>Mm-RNase13
MAPDVAWLLVLPLVFRPTLVTGITIQTAIKNFRTLHVDYPMVNYPKGFHGYCNGLMAYVRGKLQDWYCPKIHYVVHAPLEDIQKFCKYS
ESFCENYNEYCTLTQNSFPVTICTLVHQQAPTSCSYNSTLTNQRLYLLCSRKHDAEPIGIIGLY*

Rat (*Rattus norvegicus*)

>Rn-RNase1 (RNase1delta)
MDMEKYLFPFSLLILVLGWVHLYLGGESRESSADKFKRQHMDTEGPSKSSPTYCNQMMKRQGMTKGSCKPVNTFVHEPLEDVQAICSQG
QVTCKNGRNNCHKSSSTLRITDCRLKGSSKYPNCDYTTTDSQKHIIIACDGNPYVPVHFDASV*

>Rn-RNase1-like1 (RNase1gamma)
MGLEKSLILFSLLVLVLGWVQPSLGRKPSVQDFKRQHMDPDSPPNSRPTYCNQMMKRRGMTKGSCKRVNTFLHESWATVKAICSQRQMT
CKTSSRNNCHKSSSTLHITDCRLKGSSKYPNCDYTTTNSQKHIIIACEGNPLVPVHFDASV*

>Rn-RNase1-like2 (RNase1beta)
MGLEKSLFLFSLLVLVLGWVQPSLGVESRETPAQKFERQHMDEEGPFPSSPTYCNEMMKSRGMTSGWCKSMNTFVHEPLATVQAICSQG
QVTCKNGRNNCHKSSSTLRITDCRLKGSSKYPNCDYTTTNSQKHIIIACEGNPLVPVHFDDSV*

>Rn-Ear3-like
MGLKLLESRLCLLLLLGLVTCQRPTPSQWFAIQHIYNSSYPQCNAAMLRVNSYTGRCKGINTFLHASFANVVGVCGNPHTTCKDRISTN
CHNSSSQVSITFCNLTTPARIYTQCRYQTTGSLKFYTVACNPRTPRDSPMYPVVPVHLDRIF*

>Rn-R17
MGLKLLESRLCLLLLLGLVLMLASCQPPTPSQWFEIQHIYNRAYPRCNDAMRHRNRFTRHCKDKNTFLHTSFASVVGVCGNRNIPCRNR
RYRNCHNSPYRVSITFCNLTTPARIYTQCRYQTTRSRKFYTVGCDPRTPRDSPMYPVVPVHLDRIF*

>Rn-RNase2
MGVKPLESRLCLLLLLGLVSMLASCQRPTPSQWFAIQHIYNSSYPQCNAAMLRVNSYTGRCKGINTFLHTSFASVVDVCGNPHITCKDG
RSTNCHNSSSQVSVTFCNLTTPARIYTQCRYQTTGSVKFYRVACNNRTSQESPMYPVVPVHLDEIF*

>Rn-ECP-like
MGLKLLESRLCLLLSLGLVLMLASCQPPTPSQWFEIQHIYNRAYPRCNDAMRHRNRFTGHCKDINTFLHTSFASVVGVCGNRNIPCGNR
TYRNCHNSRYRVSITFCNLTTPARIYTQCRYQTTRSRKFYTVGCDPRTPRDSPMYPVVPVHLDRIF*

>Rn-RNase16-like

MGLKLLESRLCLLLPLGLVLMLASCQRPTPSQRFAIQHIYNSAYPQCNAAMQRVNNYTGRCKDINTFLHTSFASVVGVCGNRNTTCNSN
RTRTNCHDSTYQVKVTICNLTNRAAVYPQCPYQTINSSKFYRVACDPRTPRDNRTYPVVPVHLDFIF*

>Rn-RNase4
MDIQRTQSLLLLLLLLTLLGLGLVQPSYGQDRMYQRFLRQHVDPEGTGGSDNYCNVMMQRRRMTSTQCKRFNTFIHEDIWNIRSICDTAN
IPCKNGNMNCHEGIVRVTDCRETGSSVPHNCRYRARASTRRVVIACEGTPEVPVHFDR*

>Rn-Ang1
MEMSLRPLLLVFVLGLVSTPSTLAQDDPRYTKFLTQHYDAKPKGRDARYCESMMRRRGLTSPCKEVNTFIHGNKGSIKAICGANGSPYG
ENLRISQSPFQITTCKHTGGSPRPPCRYRASAGFRHVVIACENGLPVHFDESFISL*

>Rn-Ang2
MSLRPLLLVFVLSLVATPSTLAQDDPRYTKFLTQHYDAKPKGRDARYCESMMKRRGLTSPCKEVNTFIHGNKGSIKAVCGANGSPYGEN
LRISQSPFQITTCKHTGGSPRPPCRYRASAGFRHVVIACENGLPVHFDESFISLYSAGPRRRPSSVLFLSPLTPEH*

>Rn-RNase6
MAVDLPRWLPLLLLLGLWEPMGLLCAQPKGLSKARWFEIQHIWASPQQCNAAMRGVNNYTRYCKQKNTFLHESFQNVAATCGLPNITCK
NGRKNCHESVKPVKMTDCSHTGQAYPNCRYSGDVQYKLFVVACEHPKKDDPPYQLVPVHLDKVV*

>Rn-RNase9
MKSLVIKCTWPLPLLLLLPLKLQGNYWDEKEYDLEPGLREFFREIASTGPTKPPTKERIIEMITVGERPLQDYDYCNSELRAKQIHDKG
RCYPERYIVGMSYEELSKACYGQRVQCKNGVKFCRRSMDLTDGVRCVLESGERMVDCMYTTIYMTGYPVVTCQWDKESQEFIPNYIHNM
SLPE*

>Rn-RNase10
MKVTLVHLLFMMLLLLLGLGVGLGLGLHMAAAILENQPLDEFWPSDSQDTAEATEEGQGTRTTEALVLDNKEMAVPVWSEDTVLSEDEV
GGSRMLRAKTLLQSKQGYLKFDLNIRDCNVMMAHKIKEHNQSCINDYTFIHEDPSTVGAVCNSPLVDCDLKGGKCHKSPRPFDLTLCKL
AKPGQVTPNCHYLTYITEKVIIITCNNTKQLEIK*

>Rn-RNase11
MAAFLLLLVLGLLLVEPSESKTKGAREQFSQEETRSAAKQTPEESTNSTLSDENISLGISRHVMSAPPRTSRRLSFVIPKRNTVRNGRD
CVNSPSVWRTDVEVNESCQLGNNFIHSSMDVSHGIPKATSGKCERTPNLRYCDSLGLECTMCKVLAGHQCPRYHEHRITSLKRILTVLT
SHSLMSWLVTGC*

>Rn-RNase12
MILMVIVFLLLLFWENELTEDVVLTSMEHLHVDYPQSAVPLRYCNYMILQRVIREPDYTCRKVHVFIHERPQKINRICTSSKKMTCPNY
SEIFCFQSDTKFRMTVCQLTGGSKYPACRYQISPTEGFVLVTCDDLGPVNFQGYVE*

>Rn-RNase13
MASDAASLLVLQLVLQPTLVTGITIQTAIKNFRILHVDYPMVNYPKGFHGYCNGLMAYVRGKLQDWYCPKIHYVVHAPFESIQKFCKYS
ESFCEDYNEYCTLTQNSFPITVCTLDHKQAPTSCSYNSTLTNQRLYLLCSRKHDAEPIGVIGLY*

Nake mole rat (*Heterocephalus glaber*)

>Hg-RNase1
MALEKSLVLFPLLVLVLLGLVCVQPSLGKESSAMKFRRQHMDSEGSPNTNANYCNEMMRRRNMTEGRCKPVNTFVHEPLADVQAVCFQK
NVACKNGQTNCYQSTSSMHITDCRLTSNSKYPTSYRSTSQMERSIIVACEGNPYVPVHFDASVESST*

>Hg-RNase4
MALQRTHSLLLLLLLLTLLGLGLVQPSYGQDRMYQRFLRQHVDPQETGGDDSYCHVMMQRRKMTSPKCKPFNSFIHEDIWNIRSICSTTN
IQCKNNQMNCHEGVMKVTDCRQTGNSKAPNCRYRARARTRRVVIACEGYPPVPVHFDA*

>Hg-RNase6A
MVRDVRFPLLLLLGLLVLPCPLWALPKKLTKAQWFEIQHVQPSPLRCDKAMSGVNNYTRHCKPTNTFLHDSFQNVSDSCTSPNVTCKNG
QKNCHQSASPVNLTICRLTRGKYPHCPYKDVPQIKFFIVACEPPRKNDPPYPLVPVHLDGIV*

>Hg-RNase6B
MVGDVRFPLLLLLGLLCPLWAVSQPFTRFQWFAIQHVSPNPIASCNVAMRPINQHLPRCKGTNTFLHDSLQNVINVCSLPNMRCQNGVN
NCHRSASPVNKTVCRLIRGSTKPNCRYTARAMVANFVVACSPPRAGDPPDPLVPVHLD*

>Hg-RNase6C
MMRDVRFPLLLLLALLCPLCSAPPPQGFTSFQWFSTQHVQPNQNISCNVAMTAINQHLPRCKPTNTFLHDSPQNVINVCTLQSVCCRNC
QNNCHRSAQPVGATVCRLTRGSTKPNCHYTASVILTDFFVACNPRQAGDPPNPLVPVHLD*

>Hg-RNase6D
MVRDVRFPLLLLLGLFVLLGPVWPALGGHTPLSWFENQHILKKNLSCDKLMDDINNYRPGCKASNTFLHVSLQSVIDVCALPTIPCKKI
KGNICHRSRDLVPTTRCVIKNKRDSCIYNEFSKLAHVTVACKRQFDSPLYPLLPFHLD*

>Hg-RNase7/8
MTPARPRCFSLLLLLLLLGLWVAEIPVSAKPRNMTSAQWFETQHVQPKPQACNPAMDRINKYTKHCKPLNSFLHASFSNVAATCQTPSIP
CKNGHKNCHKSPKLVSLTTCKHASGRYPNCRYKEKHLNASEYIVACDPPQSKDSGKFHLVPVHLEKVI*

>Hg-RNase9
MLFTKHPLPLLFLLLLQPMQLQGTFSNFYLLPNFTKEEFDNYIYELFGTGPTRPPSKRKIEKMVLVEEDWRPLHDPQYFTEETKYKNVH
YKMRCMNHHYFLQTSYELLQQTCYNLIALCSDGTNMCKMSNKTVEGVFCKLTEGTTIPECDYESTYMHGYVIITCRWNNETQEFIPHTI
NNMVPHT*

>Hg-RNase10
MKLTLVQIFVIMLLLLLGLGSGLGLGLQMAAAVLEDSDQPLNEFWSSDLQDKAEATEERKGTQTPETLVLSNKAIVQPGWPEETVFSED
EVGGNQGLRAEARFQSYRDYLRLDLTARECNAMMAPKVMQRNHSCIPEYTFIHEDPKIVKAVCNSPAVACELKGAKCHKSPRPFDLTYC
KLSKLSQVTPNCNYLTFIMEKVILITCKDMKLQLTAIQ*

>Hg-RNase12
MILMVIVFLLLLFWEKEPDEDLVVKSIEHLNVDYPKSRIPVRYCNSMIRQRVIREPNHTCKKEHVFIHERPPKINSICVSPRKMVCPNH
ATYFCFQSEAKFKMTLCQLVGGTIYPACRYHISTLEGYVLVTCDNLGPVNFQGYVE*

>Hg-RNase13
MAPAVAQLLFLQLVLGPAPVVNIKVHPEAQNFHNLHIDYPKVTFAEGFQGYCNGFMAYVRGRRQNWFCPKVHYVLHAPWKDIQKFCNYT
DYFCDVYNEYCTLTRDFFWLTTCTLDPKHPSTGCSYTTSVTRQRVYLVCSRRYKGSPIGIISLI*

Guinea pig (*Cavia porcellus*)

>Cp-RNase1A
MEKCLVLFPLLVLVLLGLGWVQPSLGAESSAMKFERQHVDSGGSSSSNANYCNEMMKKREMTKDRCKSLNTFVHEPLAEVQAVCSQRNV
SCKNGQTNCYQSYSSMHITECRLTSGSKFPNCSYRTSQAQKSIIVACEGKPYVPVHFDNSV*

>Cp-RNase1B
MEKCLVLFPLLVLVLLGLGWVQPSLGAESSAMKFQRQHMDPEGSPSNSSNYCNVMMIRRNMTQGRCKPVNTFVHESLADVQAVCFQKNV
LCKNGQTNCYQSYSRMRITDCRVTSSSKFPNCSYRMSQAQKSIIVACEGDPYVPVHFDASVEPST*

>Cp-RNase1C
MSLVLFPLLVLVLLGLKWLQLTQDKESSAMRFQRQHMDSDSTHISNPSYCNEMMQYRNMTRGRCKPVNTFVHEPLADVQAVCFQKNVLC
KNGQSNCYQSYSSMHITDCRLTSSSKFPDCSYRMSQAQKSIIVACEGNPYVPVHFDTSVEPST*

>Cp-RNase4A
MDLQRTHSLLLLLLLTLIGLGLVQPSYGQDRMYQRFLRQHVDPQVTGGNDYYCNLMMQRRRMTSPRCKPFNSFIHEDIWNIRSICSTSN
VECKNGLMNCHTGVVKVTDCRETGNSRAPNCRYRAMARTRQVVIACEGSPEVPVHFDA*

>Cp-RNase4B
MDLQRTHSLLLLLLLTLIGLGLVQPSYGQDRMYQRFLRQHVDPQVTGGNDYYCNLMMQRRRMTSPRCKPFNSFIHEDIWNIRSICSTSN
VECKNGLMNCHTGVVKVTDCRETGNSRAPNCRYRAMARTRQVVVACEGSPEVPVHFDA*

>Cp-RNase6A
MVRAVRFPLLLLLCLFGLLCPFWAVSQDLTPFQMFQHKHVQPDPRPCDPAMTAVNELEKNHKCRPTNTFLHNSLQNVIDVCTLPNQLCR
NGQNNCHQSIHPVKMTVCQLTKGQYPNCYYRTDSTLKNFTVACEPPQQEDPRQYPLVPVHFDAIVHNSQRFSSVLTGSCYYSLFCIFGL
LFFLLHLLF*

>Cp-RNase6B
MARNVTFPLLLLLGLFAILCPLWAMGPQPPAGFTPVSWFQKQHILEKPKSCQAAMNDINQFLNGLCKRSNTFLHVSLQNVKDVCALKAF
QCRDKSTTCHRSPAPVQMTVCKIMKDTNSCTYRESHTTEHFTVQCMHRFNDPKMELLPVHLE*

>Cp-RNase6C
MERDVRFPLLLLLGLFGLLYPLWALPKGLTKAQWFEIQHIQPIPLQCDGAMSDVNNYTKRCKPLNTFLHDSFQNVSDSCTLPNITCKNG
QHNCHQSASPVSLTNCKLTGGKYPNCRYKNASQFKFFIVACEPPQKNDTSYPLVPVHLDKIV*

>Cp-RNase7/8
MTQARARCCSLLLLLLLLGLWAAELPVSAKPKSMTSAQWFETQHVQPNPQACSSAMGHINTYTKSCKRLNDFLHTSLFNVAATCQTPSKT
CKNGGKNCHQSPKPVSLTTCKLASGKYPNCHYQEKHRNARYVVACERPQKKDSGKFRLVPVHLDEVI*

>Cp-RNase7/8ps
KAPARTELHSLLLFLLLGLWMAGVQTMRSPET*SQLSGLKLTSPLALKPAAQQ*WATSTSTQSTTNPSTPSCKHSSLVWLLPARPPS*P
AGRDIKSATGTCVPGQV*VYSWEVLRLQVEGKVFGCALLWGL*PSTLEG*SQHSLAPGHLGSII*

>Cp-RNase10
MKLTLVQIFFIMLLLLLGLGLGLGLGLHMAAAVLEDSDQPLNEFWSSDSQDKAEATEEGEGTPTPETLVLSNKAVVQPDWPEETILSED
EVGGDPALKAEVSFQSYRDYLRLDLTDRECNTLMANKVKQPNQSCVSEYTFIHEHLHTVKAVCNSPVVPCELKGAKCHKSPRAFDLTYC
KLSKPGQVTPKCNYITFIMKKVILITCKDMKLHLTAIQ*

>Cp-RNase11
METFSLLLLGLGFILAEASESIMGIIKEEFAEEKMQPETIKSGQEKETDGVLMNLTLFNKNDSLSLPKDAMPSLLLTFRSCCSIPKGNS
PVSDKECFGNKMTWSKFSEANETYELNNFIHSPAEVIHRVHKAYSCKRGKNCGIWVCQSPELENTVCQLSPGKQFPRCQYHSITSLKKI
LMVLAGHSLMSWLVSGSKL*

>Cp-RNase12
MTLMVLIFLLLLFWEREPAEEIVVTSIEHLHVDYPKSNPIRYCNSMVLQRVIREPNDTCKKKHVFIHERPQKLNSVCISHRKMVCPSQS
TIFCFQSETKFKMTLCQLIGGITYPACRYQVSALEGYVLVTCDNLGPVHFHGYIE*

>Cp-RNase13
MASAVAQRLLLLQLVLGPTLITDLKLSPAAQDFLKLHIDYPKVTYADGFRGYCNGFMAYVRGRRQHWFCPKIHYVLHSPWKDIWKFCNF
TDYYCDVYNQYCTLTQDSFPLTICSLAPEQPSTSCFYNTNTSRQRLYLLCSRKYKGHPIGIISLYQGM*

Rabbit (*Oryctolagus cuniculus*)

>Oc-RNase1
MALDRPLILLVLGLLMLGLAQSVLDNESPAKKFQRQHIDPKLPLSSTYCNKKMEQQDMTQGCKPLNTFVHEPLKKIQAVCFQEKVTCKD
GKTNCYRSTSKMHTTDCSLLDTSKYPDCKYQTVQKERYIILACEGNPFVPVHFDASVE*

>Oc-RNase2/3A
MLSARLCFLLLLGILGTATSFQAPPAGFTRAQWFEVQHINMTHSRCDNAMRVVNRYRGYCKGKNTFLHVTFVDAVNTCHNPNIPCTAGG
RQNCHRSSTQVPLTDCDLTRNSSNIRQCQYRRRRKTKFYVIACDPRSPRDPPSYPIVPVHLNGTV*

>Oc-RNase2/3B
MASKLDNFQLCLLLLLGILGTAVSFQAIPPNLTPAQWFAIQHIKMANSIVCGSAMRVVNSYTGHCKGKNTFLNITFSDAVGTCVHTPNM
RCLRSSRTNCHQSSRRVPLTDCILTRNAKNYRLCTYRKLTKNKSYVIACAHKSPNDSPVFPIVPVHLDGTV*

>Oc-RNase2/3C
MASKLDNFQLCLLLLLGILGTAVSFQAIPPNLTPAQWFAIQHIKMANSIVCGSAMRVVNSYTGHCKGKNTFLNITFSDAVGTCVHTPNM
RCLRSSRTNCHQSSRRVPLTDCILTRNAKNYRLCTYRKLTKNKSYVIACAHKSPNDSPVFPIVPVHLDGTV*

>Oc-RNase4
MMTLQRTHTLLLLLLLTLLGLAQPSFGQDRMYQRFLRQHVHPQETGGNDSYCNLMMQRRKMTSHHCKPFNTFIHEDIWNIRSICSTTSI
RCKNGKMNCHEGVVKVTDCKETGSSRAPNCRYRAAASTRRVVIACEGNPEVPVHFDR*

>Oc-RNase5
MVMGLGPLVLIFVLGLGVTPPTLAQDDSRYKHFLTQHYDAKPFGRNDRYCETMMKRRDLTSPCKDTNTFVHGNKGSIKDVCEDKNGKPY
GKNFRISKSSFQVTTCKHVGGSPWPPCRYRATSGSRNIVIACENGLPVHFDESVFQQKAH*

>Oc-RNase6
MVLDLQGCLPLFLLLLQLLGPMCSLCAWPKYLTKARWFEIQHIQPNLLQCNRAMRGVNNYTQHCKPFNTFLHDSFQDVAAVCDFPNVTC
RNGRHNCHQSPKPINMTNCRLTAGKYPDCSYSDATQYKFFIVACDPPQKSDPPYHLVPVHLDEIV*

>Oc-RNase7/8A
MAPAKTEFRSLLLLLLLGLWVAEYPVTAKPKDMTSAQWFETQHVQPTPQPCNSAMSRINQYTKQCKNLNTFLHESFTSVIPTCDTPNVA
CKNGRNNCHQSPGPVSMTNCQHVSGSYPDCKYKQTEKYASFIVACDPPQTKDDSSYPLLPVHFDKVV*

>Oc-RNase7/8B
MTRAQVGFCSLLLLLLLGLWVAERPVTAKPKNMTSAQWFETQHVQPTPQPCNSAMSRINQYTKHCKSLNTFLHESFTSVIPTCNTPNVA
CKNGRNNCHKSPQPVSLSTCAHVSGSYPGCKYKEKHLKKAYIVACDPPQKGDSWHSKLLPVHLDKVF*

>Oc-RNase9
MKILISKHPLPLVLLLLQLLQPLQFQDNERRYDLPPDKADEFEDYLEEFSSTGPTRPPTKKKFIRLSLIGTERTLSDSFFCTDTVRLKN
VHNKSRCVTEHNFIVMPYDDVKNLCYTRYVECKNGIKKCHRSRLPIEGVYCKLRRGTTLPDCDYDSTYKMGFVLITCRWQNSIKKLVPV
HVNDILVLQD*

>Oc-RNase10
MKLTLAQIFFMLLLLLLGLGMGLGLGLRMAAAVLEDSSQSLEEFWSGDFQDRAEAIEKAKSARTTETPVLSNNEVEQSGWQEDSILGED
EVAGNKVPRAGTPSRGNKEYLRFDLLYRECNTLMAEKVKESNRSCITQYIFIHEEPKTVKAVCSSPAVACEFMKGKCHKSPRPFDLTFC
KLSKPGQLTPHCNYHTFVLEKHIFITCNDQKIQIASGQ*

>Oc-RNase11
METSLLLLSLGLVLTGASESTVEVTKEEFAAKKVQYALAKSGRETQTDKVLMNLTLLDKNTSPILSKDMMSSSLLTLRKLQYRIPKVNS
LSSDRECCNEVTIWRKASEANGSCRLSNDLTCGTMEGIRGVQKMPSCKCGENPGVSCCQRAEPEKTMCQLPTGRQFPRCRYHSVTSLKK
LLTVLTGHSLMSWLVSGSKL*

>Oc-RNase12
MVLMVIIFLMLLFWENELNENGVLFTLEHLHVDYPQDSAPVRYCNHMIQQRLIREPDHTCKLEHVFIHERPQKINSICMSPKRVACLNH
STIFCFQSETKFRVTVCELIEGTRYPACRYRISPTQAFIQVTCDDLGPVSFEGYVE*

>Oc-RNase13
MAAAVARLLFFQLFLGPALSLDINMQSDTENFRTLYIDYPKVKFIKSFLGYCNGMMAYVRGRIEHWYCPKIHYVIHAPWDAIQKSCKDS
KSFCENYNKYCTLTQDSFPVTVCSLGAKQPPTSCRYNSTLTNKYLYLLCSGRYDAEPIGIVGLY*

Cow (*Bos taurus*)

>Bt-RNase1_brain
MALKSLVLLSLLVLVLLLVQVQPSLGKESAAAKFRRQHMDSGSSSSSNPNYCNQMMKRRRMTHGRCKPVNTFVHESLDDVKAVCSQKNI
TCKNGHPNCYQSKSTMSITDCRETGSSKYPNCAYKTSQKQKYITVACEGNPYVPVHFDGAVLLPATPVPSLPPPHRLL*

>Bt-RNase1_seminal
MALKSLVVLPLLVLVLLLVRVQPSLGKESAAAKFERQHMDSGNSPSSSSNYCNLMMCCRKMTQGKCKPVNTFVHESLADVKAVCSQKKV
TCKNGQTNCYQSKSTMRITDCRETGSSKYPNCAYKTTQVEKHIIVACGGKPSVPVHFDASV*

>Bt-RNase1_pancreatic
MALKSLVLLSLLVLVLLLVRVQPSLGKETAAAKFERQHMDSSTSAASSSNYCNQMMKSRNLTKDRCKPVNTFVHESLADVQAVCSQKNV
ACKNGQTNCYQSYSTMSITDCRETGSSKYPNCAYKTTQANKHIIVACEGNPYVPVHFDASV*

>Bt-RNase2/3A
MVPIQQDSRLRLILLLGLLGMVISLHAQPGTLTRAQWFEIQHINMAHHQCNAAMRVVNRYRMVCKGRNTFLHRTFAYVAGICNTPNVTC
SKPGRMNCHNSSVQVPITFCNLTRNAMNYTNCRYGRTRARKIFIVACEKRSPRDSPRYPVVPVHLDNII*

>Bt-RNase2/3B
MVPIQQDSRLRLILLLGLLGMVISLHAQPGTLTRALWFEIQHINMAHPQCNAAMRVVNRYRMVCKNKNTFLHRTFAYVAGICNTPNVTC
SKPGRMNCHNSSVQVPITFCNLTRPALNYTNCQYQQTRAWRIFIVACDNRSPRDSPRYPVVPVHLDNII*

>Bt-RNase4
MALQRTQAFLLLLLLTLLGLGLVQPSYGQDRMYQRFLRQHVDPDETGGNDSYCNLMMQRRKMTSHQCKRFNTFIHEDLWNIRSICSTTN
IQCKNGQMNCHEGVVRVTDCRETGSSRAPNCRYRAKASTRRVVIACEGNPEVPVHFDK*

>Bt-Ang1
MVMVLSPLFLVFILGLGLTPVAPAQDDYRYIHFLTQHYDAKPKGRNDEYCFNMMKNRRLTRPCKDRNTFIHGNKNDIKAICEDRNGQPY
RGDLRISKSEFQITICKHKGGSSRPPCRYGATEDSRVIVVGCENGLPVHFDESFITPRH*

>Bt-Ang2
MVMVLSPLFLVFMLDLGLTPQTLAQNDAYRGFLRKHYDPSPTGHDDRYCNTMMERRNMTRPCKDTNTFIHGNSDDIRAVCDDRNGEPYR
NGLRRSRSPFQVTTCRHRGGSPRPPCRYRAFRANRVIVIRCRDGFPIHLEENFIPPRP*

>BT-Ang3
MVMVLSPLFLVFMLGLGLTPLTLAEDDRRYRHFLIQHYDRSPKGRDNKYCETMMEKRHLTKPCKSINTFVHGNKNDIKDICKDKNGKPY
RGNLRISKSPFQVTICKHKGGSTRPPCHYKATKDYRVIVIGCEKGWPVHFDESFVPPHK

>Bt-RNase6
MGPHLLGRSSLLLLLLGMWWSVRPLCAVPKGLTKARWFEIQHIQPRLLQCNKAMSGVNNYTQHCKPENTFLHNVFQDVTAVCDMPNIIC
KNGRHNCHQSPKPVNLTQCNFIAGRYPDCRYHDDAQYKFFIVACDPPQKTDPPYHLVPVHLDKVV*

>Bt-RNase7/8A
MAPARAGFCPLLLLLLLGLWVAEDAVSARPGNMTPAQWFETQHVQPSPQGCNAAMRKINKFSKHCKDLNTFLHESFSSVATTCQTPNIT
CKNHENNCHQSQKPVSLTECKLTSRRYPDCKYKEKKQITSYIVACKPPKKGDSGKFKLVPVHLEKVL*

>Bt-RNase7/8B
MVPARAGFCPLLLLLLLGLWVAEDAVSARPGNMTPAQWFETQHVQPSPQGCSAAMHKINKFSKRCKDLKTFLHESFSSVTTSCQMPNIA
CKNGRNNFHQSQMPVSLIECKLTSEKYPDCRYNEEERNASYIVACEPPEKKYSKKFKLVPVHLDKVLNIFIFPWPWVLP*

>Bt-RNase9A
MGTLINVQPLFLLFLLLKPLQFVRINDSYLSDERREELEDYIDDLHATGPTKPPTKEAFKTHVIIDSEMPLTDEDYCNLEMKRKRVHNR
LYCAKEHFFLQASYEDIEEMCYNIFVKCTNGIRKCHRSRQIISGVHCVLTSGIMMPFCEYTSSFKEGWVFITCQWEDDIGEIIPVSVID
ILAI*

>Bt-RNase9B
MGTLINMLPLFLLFLLLKPLQFELIIDGSLTDKEFEEHLDGTSPTRAPTKELFKAHVIIDRKRPLHDPTYCSDEMKIKNIHRRLYCVKE
HFFLQASYEDIEKICHNTFMSCGEEVGTCYSSQKNIKAVHCVLTSGIKMPDCEYSSSYEKGWVFITCRWQNDIREIIPESVVGMLTSTR
KVPSPYRS*

>Bt-RNase10
MKLTLVQIFFMMLLLLLGLGVGLGVGLQMAAAVLEESDQLLDEFLSSDSQDKAEATKEGLASRSTETLLVSNKEVVQPEDTIISEDEVG
GDRMLRAEVLLHSNKDYLRSDVMDRECNALMALKVKSKDHTCIPQYIFIHEELDAVKAVCKSPAVACDLKGGKCHKSPRPFDLTFCKLS
KSGQVIPHCNYVTFILEKYILMSCSDMKVQITS*

>Bt-RNase11
MEIFFLLLLGLGVIFAGVSESIMEIIKEEFLEKEMKYDMAKSYQEKHTIEVLINLTVSYKNTSLSMSKDVSSSLLTFRRLHYSLPPKCN
PGNNKHYCNDMTVWRKVSEANGSFKLSNNFIHGSVEVVDGVPKAPSCKCGQTSGISCSETPELRTTTCQFTVGKQSPSCQHHGVTSLKK
ILVVLTSHSLMSWLVSVSNL*

>Bt-RNase12
MILMVIIFLVLLFWENELDEEAMAATLEQLHVDYPQSDIPIRYCNHMIIQRVIKEPNNTCKKGHFFIHERPRNINSVCSSPRRVTCQNH

APSLCFLSEIKFKMTVCKLIEGTRYPACNYHVFATEGFIVVTCDDMGPAKIQRYTE*

>Bt-RNase13
MAPLVAQLLFLQVVLGTALLENIKTQLAIKNFRTLHVDYPKVTYAQGFQGYCNGLMSYVRGRQESWYCPRIHYVLHAPWTVIWKFCKYS
ESFCENYNEYCTLTKDSIPLTICSLYPRQPPTSCRYNSTLTNQRLYLLCSEKYDGEPIDIIGLY*

>Bt-RNase14
MNLTWTLLLLLLLELTVFASGLPFSRRHIDNPRSWVPGGQHRYCDVMMRRRWLIHRGRCKQINTFIHEDLATIADFCTTPAVPCTSSGS
LLSCHNSSHDVSVTDCFAKAGTRPPYCHYQKKDSIRPICVGCKNGAPAHLDS*

>Bt-RNase15
MLLTWTLLLFLLMDQPSFSEAQDLLHHQTDRPRTKISANHKYYCDLMMKARGLATDQSCKKNKTFVHSVSPGTRGLCEGPAVTCRKMSE
VYNCHLIIFKVTQCNLYPEAVPPHCYYEGVTFQMDVRIVCVGKRPIHLDE*

Horse (*Equus caballus*)

>Ec-RNase1
MAQEKSLILFPLLVLVLLVLGCVQLSLGKESPAMKFERQHMDSGSTSSSNSTYCNQMMKRRNMTQGWCKPVNTFVHEPLADVQAICLQK
NITCKNGQSNCYQSSSSMHITDCRLTSGSKYPNCAYRTSQKERHIIVACEGNPYVPVHFDASVEVST*

>Ec-RNase2/3
MVPTQRDSRLCLLLLLGILGMVISYHATPAGLTRTQWFEIQHIKNMTHRRCNNEMLRVNNYTKRCKNINTFLHTTFAFVASVCNTPNVT
CPTTRYMNCHNSSVQVDITDCNLTSAPQPYKNCNYRQTSARKYFIVACNNSQPGDNATYSVVPVHLDWIS*

>Ec-RNase4
MALQRTLSLLLLLLLTMLGLVLIQPSYGQDRMYQRFLRQHVDPEVTVGSDAYCNLMMQRRKMTSYQCKRFNTFIHEDIWNIRSICSTTS
IQCKNGKMNCHEGVVKVTDCRETGSSRAPNCRYRAMTSTRRVVVACEGNPEVPVHFDR*

>Ec-RNase5
MAMSLCPLLLVFVLGLGLTPPSLAQDDSRYRQFLTKHYDANPRGRNDRYCESMMVRRHLTTPCKDTNTFIHGSKSSIKAICGNKNGNPY
GETLRISKTRFQVTTCKHAGGSPRPPCRYRATPGFRSIVIACENGLPVHFDESFFRP*

>Ec-RNase6
MVLSLLGRCPLFLLLLGLWVLVHPLCAWPKSLTQARWFEIQHIQPIPLQCNKAMNGVNNYTKHCKPENTFLHDSFQNVASVCNLRSIIC
KNGQKNCHQSSRPVHMTQCSLTSGTYPNCRYRDAAPYKFFIVACEPPQQGDPPYHLVPVHLDSIV*

>Ec-RNase7/8
MAAARAGFCPLLLLLLLGLWVVKVSVSAKPKHMTPAQWFETQHVQPKPQGCNTAMGNVNKYTKRCKDLNTFLHESFSSVATTCQTPSIA
CKNGHKNCHQSQKSVSLTMCDLTSGRYPDCRYKEKQLDAFFIVACDPPQKGDSGQFQLVPVHLDKVLQISSLPHSWVVP*

>Ec-RNase9
MWILITTQLLPLLLLMLQPLQFEKLFKYSDLSDVDSEEFEDYLEELYSTGPTKAPTKKTFQNQVLADPDRPLSDPHYCNDEVRMKNVHN
RLYCKKEHFFLQATYEEVQKICRHLFVPCKNGVKKCHRSKKLIEGVYCSLTSGTMIVDCIYESFYKRGFALITCRWQNDIQEIIPDHVD
DLQELYS*

>Ec-RNase10
MKLTLVQIFFMMLLLLLGLGMGLGLGLQMAAAVLEDSDQSLNDFWSSDSQEKAETTKEGDGTRTTETLLLSNKGVVQPVWPEETILAED
EVGGNKMLRADALFQSDKDYLRLDLMNRECNSLMAHKVKKRNHTCIPEYTFIHEELDTVKAVCKNPVVACDLKGAKCHKSSRPFDLTFC
KLSKPGQVTPHCNYLTFIFEKFIIISCNDMKVKIT*

>Ec-RNase11
METFSLLLLGLGLVLAGASESIMKIIKEELSEEKMKYGMTKSDQGKQTVELLMDLTLLYRNTSFGMSKDIMSSSLLTFRRLHYSFLKGN
SPGNDKEYCNDMTVWRKVSEANESCKFSNNFIHGSIEVIHSTPKAPCCKCGQNAVISCSENAELETTMYQLTRGKQFPRCQYHSVTSLK
KILAVLTGHSLMSWLVSGSKL*

>Ec-RNase12
MILMVIIFLLLLFWENELDDEGVVSTLEHLHVDYPQSDIPVRYCNSMILQRSIKGPDNACRKEHVFIHERPRNINRICSSPNKMACQNN
STILCFQSKTKFKMTVCKLIEGTRYPACRYRVSPTEGFILVTCDDMGPVNLQRYVE*

Dog (*Canis familaris*)

>Cf-RNase1
MAQEKFLVLLPLVVLALLGLACVQPSLARESKAMKFQRQHMDSHPAAISASYCNLMMKRRNMTDGWCKPVNTFVHEPLADVQAVCSQKD
VLCKNGQSNCHQSRSQMNITDCRLKNGSKFPKCVYTTTQKEQYIVVACEGNPHVPVHFDACL*

>Cf-RNase4
MAPPRTRALLLLLLTLLGLGLVQPADGQDRMYQRFLRQHVDPEGKGGNDTYCNVMMQRRKMTTRQCKRFNTFVHEDIWNIRSICSTTNIQ
CKNGKMNCHEGVVKVTDCRETGSSRAPNCRYRASASTRHVVIACEGDPELPVHFDR*

>Cf-RNase6
MMLHLPGPWPLLLLLLLGSWGPVPPAAAPPPGVTAVRWFITEHVRAGPVRCSTEMPQINYRPNICKGQNTFLQESFQNVVATCQQPNRT

CKNGLGNCHKSAGRVNMTYCLLTGRRPQCTYRTTYQNQFYIVACNNSQPGYPPNLLLPVHLDDTVPLGPTHFG*

>Cf-RNase9
MWTLLTMQPLALLLLLMMQPLQFIILKNFGDFTDEKLEEFEDYLGDIYSPGPAKPPTKDTFLRRIIIDPGRPLTDSAYCTEEIKMKNVH
NNFSCVREHFFLQIAYEDVQKTCKNLHVPCKNGVKKCHRSKEVIEGVYCNLTRGAKMTDCEYDSFYRQGYVLITCRWQNNIQEIVPVYV
DDIMALDNDVKANIYHNAKK*

>Cf-RNase10
MKLTVVQILFMMLLLLLGLGMGLGLGLRMAAAVLEDSDQSLMELWSSDSEDTAEATKGEGSRTTENLVLSSKGVLPPGWPEDPILGEDE
VAGNKVGGEPLLPSNKAYLRSDLLARECNTLMAPKVKGHNRTCISQYTFIHEDLDTVQAVCNSPVVACQLKGGKCHRSSRPFDLTFCRL
SKPGQVTPHCHYVTFIFEKYIIISCNDMKVQVVSGQ*

>Cf-RNase12
MILMVIIFLLLLFWENELHEEREVPTLEHLHVDYPQSDIPVRYCNRMVLQRVIRGPDNTCKKEHVFIHERPREINRVCTSPKKRVCQNH
SSILCFQSVTKFKMTACQLIEGTRYPACRYHISPIMGFAVVTCDHMGPVTLQRYVE*

>Cf-RNase13
MAHAVARLLFLQLLLGPTLVVDIRVQIATKKFRMLHIEYPRVNYPEGFQGYCNGLMGYVRGKKQSWFCPQTHYMVHAPWREIQKFCRYS
DSFCENYNEYCTLTEDSFPVTICSLASQQPPTSCNYNDTLTNQRLYLLCSGKRDAEPIGIIGLY*

Giant panda (*Ailuropoda melanoleuca*)

>Am-RNase1
MAQEKFFILFPLLVLVLLALGCVQPSLGKESRAKKFQRQHMDSDTSIVGSSYCNQMMKRRNMTAGWCKPVNTFVHEPLPDVQAVCLQKN
VTCKNGQPDCHQSSSRMHITDCRLKRGSKYPKCEYQTEQKEKSIIVACGGNPYVPVHFDASV*

>Am-RNase4
MALHRTRSLLLLLLLTLLGLGLVQPSYGQDRMYQRFLRQHVDPEGTGGNDTYCNLMMQRRKMTTHQCKRFNTFVHEDIWSIRSICSTTN
IQCKNGKMNCHEGVVKVTDCRETGSSSAPNCRYRASASTRHVVIACEGDPQLPVHFDR*

>Am-RNase5
MVKGLGPLLLVFMLGLGLTPPARAQDDSRYKHFLTQHSDAKPRGRNDRYCESMMERRGLTTPCKDTNTFIHGNKGSIKAICGNKNGNPY
GEALRLSKSPFQVTTCRHVGGSPRPPCRYRATPGFRHIVVACEHGLPVHFDESFFRL*

>Am-RNase6A
MMLDLLGPFPLLLLLLGSWGPVHPLGASAQPPTRAQWFQIQHISGGPVQCNNAMRRVNNNNQRCKPQNTFLHTTFQNVAATCSLPTRPC
RNGQNNCHRSARRIGMTYCNLTGGRFPNCRYSTTPQNQFYTVACNPRQPGDPPYRLVPVHLD*

>Am-RNase6B
MMLDLLGPFPVLLMLLGSWEPVHPLCHCGQPSASVQTFILRHLRAGPLQCNDAISATVRTFQIKHLREHRVQCNREMRRVNILDQRCKD
QNTFLYDSLHNVAATCLLPSMRCRNRTQMNCHQSESPINMTYCNLTGGRFPNCRYSTALQYRFYVVACNPRLPRDPPYPLVPVHLDLTI
PVVSLHLG*

>Am-RNase6C
MMLDRLGPFPVLLLLLGSWAAVLPPGAWTQPPTRVQKFIIQHLSAGPVQCNIAMRAVNNLNQTCKDQNTFLHDNFPNVTVTCGWPNRNC
RNGQNNCHQSTYAVGKTHCSLTGGRFPNCSYRTTPQHGLYIVACDPRQRGYPPNCLFPVHLD*

>Am-RNase6D
MMLDLLGPFPVLLMLLGSWEPVHPLCHCGQPSASVQTFILRHLRAGPLQCNDAMRLVNLSNRTCMRQNTFLHDTFRNVADTCGWPNRIC
RNGQDKCHLSANRINMTHCYHTGGRYPNCSYRTIPRQGLYTVACDPRQPGFDANCLFPVELD*

>Am-RNase6E
MMLDLLGPIPVLLLLLGSWAPVHPLGDWAQPSSSVQTFILRHLRAGPVQCNTETPPVIHLEQCRKPENTFLHDSFHNVSDTWLLPNRTC
SRGQKNGQQGANLSDMTHCNLTGGRYPHCRYNTAPQNRFYIVAITTLSQATLPISCFLYT*

>Am-RNase7/8
MAAVSMGSCPLLLPLLLGLRVAEVPVRAKPSNMTSAQWFEIQHVQPKPQRCDAAMGKVNKYTKHCKPFNTFLHQSFSSVAATCQTPTVA
CKRGRRNCHQSKKPVSLSTCELTSRKYPGCKYKEKRQVASYIVACDPPEKGDSRKFKLVPVHVDKTL*

>Am-RNase9
MRAPRAARPLALLLLLLQPLQFKFLRMHLGKSEEETEEFEDYLEELLRPGPARPLTKDAFQQRTIIDPTRPLTDPEYCTAEMKMKNVHN
KFRCVREHFFLQVAYEELQKLCKNIFVPCKNGVKKCHRSRQLIEGVYCNLTRGVRMSDCEYESSYRQGYVLITCRWQNDIQEIIPAYVN
DIMTLDKHVKGGNIFYSELEWPF*

>Am-RNase10
MKLTLVQIFFMMLLLLLGLGLGLGLGLQMAAAILEDSDQSLSELWSSDSDDKTKATKGEGSRTAETLLLSNKGVVQPGWSEDTVLGEDE
VRGSKMLRDEPPLHSHKDYLRSDLMTRECNTLMAQKLKEHNRTCISQYTFIHEDVDTVKAVCNSPVVACELKGGKCHKSSRPFDLTLCR
LSKPGQVTPHCNYLTFIFERRIIISCPDMKVQIMSGQ*

>Am-RNase11
METFSLLLLSLGLVLAGDSESLMERIKEEFSGGEMQYDMGNSDQGKQTIEVLMNLTLLYKNTSLRMSKDILSSSLLTFRRLRYSFAKGN

SPGNDKEYRNDMVVWREVSEANGSRKLSYNFTHGSMGVIHGAPKASSCKCGQNLGTNCSRSPELDTTTCQLAVGEQFPRCQYHSDTSLK
KILAVLTGHSLMSWLVSGSKL

>Am-RNase12
MILMVIIFLMLLFWENELNEEGEVWTMERLHVDYPKSDIPVRYCNRMVLQRVIRGPDNTCKKEHVFIHERPRNINRVCTSSKKRICQNR
SSTLCFQSMTKFKMTVCQLVGGTRYPACRYRMSSMKGFVAFTCDDMGPVSLQGYVE

>Am-RNase13
MAPAVARVLFLQLVLGPTLVVDIKVQIAIKNFRMLHVDYPRVNYPEGFQGYCNGLMAYVRGRKQSWFCPQIHYTVHAPWGEIKRFCLYS
DSFCENYDEYCTVTEDSFPVTICSLASGQPPVSCHYNDTLTNQRLYLLCSGKHDAEPIGIIGLY*

Microbat (*Myotis lucifugus*)

>Ml-RNase1A
MAQEKSLILSPLLVLVLLVLGWVQPSLGKESRAMKFQRQHMDPDGPSNNSNYCNQMMRRRHMTEGRCKPVNTFVHEPLVDVQAVCLQGN
ITCKNGQPNCHKSSSSMKITDCRVTRGSKYPNCDYRTSQKERHIIVACEGNPYVPVHFDASVEASS*

>Ml-RNase1B
MAQEKSPILFPLLVLVLLVLGWVQPSLGKESRAMKFQRQHMDPDGPSNNSNYCNQMMRRRLMTEGRCKPVNTFVHEPLVDVQAVCLQRN
ITCKNGQPNCHKSSSSMKITDCRVTSGSKYPNCDYRTSQKERHIIVACEGNPYVPVHFDASVEASS*

>Ml-RNase1C
MAQEKSLILFPLLVLVLLVLGWVQPSLGKESRAMKFQRQHMDPDGLSNNSNYCNQMMRRRHMTEGRCKPVNTFVHEPLVDVQAICLQGN
ITCKNGKPNCHRSSSSMKITDCRVTSGSKYPNCDYRTSQKERHIIVACEGNPYVPVHFDASVEVSS*

>Ml-RNase1D
MAQEKSPILFPLLVLVLLVLGWVQPSLGKESRAMKFQRQHMDPDGPSNNSNYCNQMMRRRLMTEGRCKPVNTFVHEPLVDVQAVCLQGN
ITCKNGQPNCHKSSSSMKITDCRVTSGSKYPNCDYRTSQKKRHIIVACEGNPYVPVHFDASVEVST*

>Ml-RNase1E
MAQEKSLILFPLLVLVLLVLGWVQPSLGKESRAMKFQRQHMDPGGPSNNSNYCNQMMRRRNMTKGWCKRVNTFVHEPLVDVQAVCLQGN
ITCKNGEPNCHKSNSSMNITDCHETSGSKYPNCDYQTSHKERHIIVACEGNPYVPVHFDASVEVSIGDQSSEMPLESSVSPSSLLPLRE
MTQGRAAMQHKSVSLA*

>Ml-RNase1F
MAQEKSLILFPLLVLVLLVLGWVQPSLGKESWAMMFQRQHMDPDGPSSNSNYCNQMMRRQLMTERQCKPVNTFIHEPLVDVQAICLQGN
IICKNGKPNCHKSSSSMKITDCRVKSSSEYPFCDYETSHKERHIIVVCGGNPYVPVHFHASVEVST*

>Ml-RNase1G
MAQEKSLILFPLLVLVLLVLGWVQPSLVKESPAMKFQRRHMDPGGPSNNSNYCNQMMRRRNMTEGRCKPVNTFIHKPLADVQAICLQGN
TPCKHGQPNCHKSSSRMKITDCRETNGSQYPNCDYRNSHKERHIIVACAGNPYVPVHFDASVEVAT*

>Ml-RNase2/3A
MVPTQRDSQLCLLLLLGLMGMVISFHAPPRGLTWAQWFEVQHVNMTNTRCTIAMQAINRLRRPCKGQNTFIHTSLAAVVNLCYTANITC
RNGRDKNCYRSRVAVHLTYCNLTRPAHPYYQCQYQQVALLRNYSVACDNGHIPVHFDRII*

>Ml-RNase2/3B
MVPTQRDSQLCLLLLLGLMGMVISFHAPPGGLSWAQWFEVQHVNMTNAQCTIAMQPINRLNYVSFQRPCKGQNTFINTSLAAVVNLCSG
TNITCRNRVDKNCYRSRVAVNLTYCNLTGPARHYNQCQYQQVTLLRNYSVACDNGHIPVHFDRII*

>Ml-RNase4A
MALLKTLSSLLLLLLTLLGLGLVQPSYGQPMYKRFLRQHVDPTVTGGDVKYCNLMMQRRRMTQFRCKQFNTFIHEDIWNIRSICSTTNI
QCKNGKMNCHEGVMSVTDCRDTGSSPAPNCRYGARASTRRVVIACEGNPPVPVHFDG*

>Ml-RNase4B
MALLKTLSSLLLLLLTLLGLGLVQPSYGQPMYKRFLRQHVDPTVTGGDVKYCNLMMQRRRMTQFRCKQFNTFIHEDIWNIRSICSTTNI
QCKNGKMNCHEGVVSVTDCRDTGSSPAPNCRYGARASTRRVVIACEGNPPVPVHFDS*

>Ml-RNase4C
MALQKTLSLLLLLLLTLLGLGLVQPSYSETMYERFLRQHVDFPATGGTNLYCNTMMQRRGMTTPSCKQFNTFIHEDNSTIDTICITRNI
LCKNGMMNCHAGVVNVTDCRDTGRTRPPNCSYQGRASTRRVVIACNGNPPLPVHLDR*

>Ml-RNase4D
MALQKTLSLLLLSLLTLLGLGLVQPSYGQFMYQRFLRQHVDSTQRGVTSSYCNTMMERRGMTRPRCKQFNTFIHADIGAINNICRTPNI
RCKNGKENCHEGVVRVTGCRLTGSSWGNCRYQGRGSSRRVVIACEGNPQVPVHFDG*

>Ml-RNase4E
MALQKTLSLLLLSLLTLLGLGLVQPSYGETMHQRFLREHVDSNWTGGNNWYCNTMMQRRGMTRPRCKQLNTFIHADIGTIKNICRTPNI
RCKNGRMNCHGRHVRVTECSLTASSQRKCSYQGRGRSRRVVIACQGNPPVPVRFDK*

>Ml-RNase4F

MALQKTLSLLLLSLLTLLGLWLVQPSYGETRYEKFQRQHVDSTGPGGTDFYCINRMDQKGMTRPKCKDFNTFIHENIKTINNICNNPNI
LCKNRYDMNCHEGVVRVTDCRLTERSPHCRYQGRGRTRRVVIACDGYPLVPVHFDS*

>Ml-RNase4G
MALQKTLSLLLLSLLTLLGLWLVQPSYDQGNYEKFQRQHVDSRGPGGTDLYCNTMMQKRGMTLDGCKKFNTFIHEDIGTINNICNAPNI
RCKKGRMNCHAGVVRVTDCSLTGGHSPYDCRYGARASTRRVVIACEGNPLVPVHFDS*

>Ml-RNase4H
MALQKTLSLLLLLLLTLLGLGLVQPSYGQSTYQRFLRQHVDSRGPGGTNLYCNTMMQRRGMTRPTCKQFNTFIHENIRTINNICRTTNI
RCKNGRMNCHAGVVRVTDCRLTARFPQNCRYQGRGSSRRVVIACEGNPLVPVHLDS*

>Ml-RNase4I
MALQKTLSLLVLSLLTLLGLGLVQPSYGETRNEKFQRQHVDSIGPGGTNLYCNRRMDKQEMTKLKCKPFNTFIHENIGTINNICNNPNI
RCKNGMMNCHAGVVRVTDCKDTGSSSPPNCSYKATASTRRVVIACQGNPLVPVHIDR*

>Ml-RNase4J
MALQKTLSLLLLSLLMLLWLGLVQPSYGETMYQRFLREHVDSTGTGGNSSYCKKMMQEREMTRPRCKRFNTFIHEDIGTIKNICKTPQI
PCKNGKMNCHEGEVRVTDCRLTARFRGNCRYQGRGSSRRVVIACQGKLPVHFDK*

>Ml-RNase4K
MALQKTLSLLLLLLLTLLGLGFVQPSYGQFMYQPLQQQHVDSTQRGGTSLYRNTMMQRQGMTRLRCKQFNTFIHENIGAINNICRTPNI
QCRNGKENCHEGVVRVTDCRLTASFPVNCRYEGRGSSRHVVIACEGNPQVPVHFDG*

>Ml-RNase5A
MMIGLGPLLLVFILGPCLTPPTLAQDDYRYRHFLEQHYDLNPRGRNDAYCDTMMRRRGLTSPCKDTNTFIHGTSNNIKAVCEDENGKPY
GANFRISKSPFQVTTCKLRGGSNRPPCRYRATAGSRDIVIACEHGLPVHFDESFYRP*

>Ml-RNase5B
MMMGLGLLLLVFMLGPCLTPPTLAQDGRYKHFLNQHYDAKPSGRNDAYCDTMMRRRGLTSPCKDTNTFIHGTSNNIKAVCEDKNGVPYG
ANLRRSKSPFQVTTCKLRGGSNQPPCRYRATAGSRDIVIACERGLPVHFDESFYRP*

>Ml-RNase5C
MMIGLGPLLLVFMLGPCLTPPTLAQDDYRYRHFLDQHYDANPRGRNNRYCDTMMRRRGLTSPCKDTNTFIHGTSNNIKAVCGDENGMPY
KDNFRISKSPFQVTTCKLRGGSNQPPCRYRATPGYRDIVIACEHGLPVHFDQSFYQP*

>Ml-RNase5D
MMMGLGLLLLVFMLGPCLTPPTLAQDDRRYRHFLDQHYDANPHGRNNKYCDTMMRRRGLTSPCKDTNTFIHGIKKNIKAVCEDENGVPY
GTNLRRSKSPFQVTICKLRGGSNRPPCEYKATAGSRDIVIACERGLPVHFDESFYRP*

>Ml-RNase5E
MMTGLGPLLLVFMLVPCLTPPTLAQDVDRYKHFLNQHYDLNPRGRNDAYCDTMMRRRGLTSPCKAKNTFIHGTSNNIKDVCGDKNGEPY
KENFRRSKSPFQVTTCKHIRGSTRPPCRYRATPGYRSIVIACEHGLPVHFDESIYSP*

>Ml-RNase5F
MMTGLGPLLLVFMLGPCLTPPTLAQDVDRYKHFLEQHYDLNPRGRNDAYCDTMMRRRGLTSPCKDTNTFIHGTENNIKDICTDKNGEPY
KENFRRSKSPFQVTTCKLRGGSTRPPCRYRATAGSRNIAIACGPGLPVHFDESFF*

>Ml-RNase5G
MMMGLGPLLLVFMLVPCLTPPTLAQDDRRYKHFLNQHYDAKPSGRNDAYCDTMMRRRGLTSPCKAKNTFIHGNSNNIKDVCGDKNGVPY
GKNLRRSKSPFQVTACKHRGGSNRPPCRYRATAGSRSIVIACEHGLPVHFDESFF*

>Ml-RNase6
MVLDLLKCFPPLLLLLGLWGPVYPLYALPKNLTKAQWFEIQHIQPSPLQCNKAMHGVNNYTRHCKRQNTFLHDSFQNVAAACELPKIVC
KNGRNNCHQSSKPVNLTNCNLTAGKYPNCSYKDSAQYKFFIIACDPPQKRDPPYHLIPVHLDKLV*

>Ml-RNase7/8
MAPARARFCPLVLLLLLGLWVAQVPVSAKPKHMTSAQWFEIQHVQPSPQACKGAMSNINKHTNHCKRLNTFLHESFSSVAATCQNPTIA
CKNGQKNCHQSKGPVSLTTCELTSGKYPNCNYKENALHASYIIACNPPQKGDAEKVHLVPVHLDKVV*

>Ml-RNase9
MCTLLTSQSLPLLLLLLQPLPLRVLMLDKHGFSDEMDEKFKDYIDELYSTGPTRPPTKESFQKYVMLEPDYKLYYDHLCTHEILFRNIH
NRFYCKKEHFFLHIAYEELQKTCHTKYVACKNGIRKCHKTKEQIEGAHCALTEGTNMPGCRYETTYKKGYALITCRWQDDIGEIIPDYV
NSILEIPGK*

>Ml-RNase10
MKLTLVQIFFMMLLLLLGLGMGLGLGLRMAAAVLEDSDQSLNELFWSSDSQDKAGVTKEGEGTRTTETLLLSNKGVVQPGWPEETIVNE
DEVGGNKMLRAEALSQSNKDYPRLDLMARECNTMMAHKMKEHNHTCITQYTFIHEELDTVKAVCKGPVIACELQGRKCHKSSRPFDLTF
CRLSKPGQVTPHCNYLTFIFEKFIIISCSDMKVQVIPNR*

>Ml-RNase11
MEASLLLLCLGLILVGSSGNKMETIKEEFSEGEMQNDLAKSDQEKQTIEILMNLTLLYKNTSLGISKDITSSLSTFRRLHDSFSKGNNP

GNDKECYNDIVIWRKVSEANGSCKLSNNFILGSMEVICRAPKTPSCKRGQNLGISCFESPDLVITMGQLTTGKQFPRCQYHSVTSFKKI
LAVLTGHSLMSWLVSGSRL*

>MlRNase12
MILMVIIFLMIVFWENELYEETVMSTIEHLHVDYPQSNIAVRYCNHMILERIIKEPDNTCKKEHVFIHERPRQINRVCTSPKRTACQNH
SSISCFQSETKFKMTVCKLIEGIRYPACRYHISPTEGFIVVTCDDMGPVNFQKYVE*

>Ml-RNase13
MAPAVAWLLFLQLVQGPTLVMDISIEVAIQNFRTMHIDYPKVNYPEGFQGYCNGLMAYVRGRQQSWYCPKRHYVIHAPWTDIEKSCKYC
ESFCENYNEYCTLSEDSFPLTICSLETKQPPTSCRYNSTLNNQRLYLLCSQKYDAKPIGIIGLY*

African elephant (*Loxodonta africana*)

>La-RNase1
MTLEKSFVLLPLLALMLLVLGWAQPSLGKESPARKFQRQHMDSDSNPSNSSSYCNLMMRRRNMTKGYCKSVNTFVHNPLVDVQAICLQK
NITCKNGQPNCHQSNSSIPITDCRLTGSSKYPNCAYRTSNKMRHIIVACKGKPYVPVHFDGSVEVSSLGQSTHTLTTS*

>La-RNase4
MTLQRTGSLFLLLLLTLLGLGLVQPSYGRESKYQRFLRQHMDPEGSGGNDGYCNLLMQRRKMTTSWCKPFNTFIHEDIWNIRSICSTTN
IQCKNGSMNCHEGVVKVTDCKETGSSRAPNCRYRAKTSTRRVVIACEGNPEVPVHFDK*

>La-RNase6A
MALDLLGHFPHLLLLLGLWGPMHPLFAVPPNLTRAQWFQIQHLRPSPLPCNQAMRGVNNYTHHCKDLNTFLHDSFHNVAAVCNLANITC
RNRSTNCHRSPNRVNMTVCNLTAGRYPNCNYNRAAAYKFFIVACNTPQAGGPPVPVHLDGVI*

>La-RNase6B
MVLDLLGHFPHLLLLLGLWGPMHPLFAVPQNLTKAEWFEIQHLQPSPLLCNQGMRGVNNYIQHCKPVNTFLHDSFQNVTAVCNLTNITC
KNGLNNCHQSQNPVNITHCRLTAGKYPKCTYKNAALYKSFIVACDPPQTGDPPYPLVPVHLDKII*

>La-RNase6C
MVLDLLGRFPHLLLLLGLWGPMYPLFAVPPNLTKAQWFQIQHLLPSPSQCNQAMRGVNNYTQHCKDVNTFLHDSFQNVAAVCNLTNIKC
RNGRNNCHRSQNPVNMTKCTLTSGNYSNCIYGNAALRKSFIVACDPRQRGDPPYPLLVPVHLD*

>La-RNase7/8
MTPARAGFWPLLLLLLGLWVANVPVSAKPKDKTPAQWFEIQHVQPHPRGCNAAMGSINKDKKHCKGLNTFLHDSFSSVATTCKNPTIAC
KNHRKNCHKSRGPVSLTICVHTSGKYPNCKYKEQSKKASYIVACDPPQKSDSGHFQLVPVHLDKVF*

>La-RNase9
MWQPSVVMYPLSVLLLLFPQAQFKTTHNPFIVSGNFEEDFEDYLTEFYGTGSTTEPTKEKFKRKMIIVPGRQIHISNEEYCSHAILFKN
IHNKLRCVKEHYFLQEPYEEIRKICYNSFIKCKNGIRKCNRSKKMMEGLYCKLRRGTTSPDCEYESFYKRGLAIITCKWQNSIGELVPV
SINDISMPYSY*

>La-RNase10
MKLTLVPIFFMLLLLLLGLGMGLGLGLHMAAAVLEDSDQPLNEFWASDSQDKAEADEEGEGTRTTETLVLSDQGLVQPGWQPEGTIFNE
DEVGDKMLRADVLSQSSKDYLRYDLLSRECNAMMAHKMKKHNQTCITQYTFLHEDEDTVKAVCNSPVIACELKGGKCHKSTRPFNLTHC
KLSKPGQVTPHCNYLTFIFEKYILITCNDTKPQLSPGQ*

>La-RNase11
METFSLLLLDLGLVLAGTSGSTMETIKEEFSEEDIEYDIAKACQEKQTIEVFMNSTLLDKNTSLSMSKDVMSSSLLTFRRLHYNFPKEN
SLGNGKQYCNAMVVWRIVSEANGSCKLRNNFTHGSTEMIHGIHKVPSCDYEPNFDMVQGNMVEPKCYKSPELETTMCQLTTGKQLSRCQ
YHSVTSLKKMLIVLTGHSLMSWLVSVSKL*

>La-RNase12
MVIIFLLLLFWENEMNVESGMPTLEQLHVDYPQSDVPVRYCNYMILQRVIKEPDNTCKKEHVFIHERPQKINAICTSVKKKACSNHSKL
FCFQSKIKFKMTACELIEGSRYPACRYHISPKEGFILVTCDEMGAVNFQGFVE*

>La-RNase13
MAPAVARLLFLQLVLGSALVLGINLWSAIKNFNHLFIDFPRVEYSSKFQGYCNGIMSYVRGRMQNWDCPKIHYLVHVPFKSIRKYCKHS
ENFCENYNHYCTLTKDSFPLTICQLSDKQPPTSCYYNTTLTNQKLYLLCSRKYDADPVDIIGLF*

Opossum (*Monodelphis domestica*)

>Md-RNase1
MALKGSHLMLLLLGLFLAELVHQSTGKESPSKKFQRQHMDPENSASDDTKYCDHMMKARNMTKGRCKPVNTFIHEPKEIVDAVCRETNI
TCKNGQSNCYQSSQPMSLTHCRQTGASKYPNCQYRGSDITKQIIVACEGNPYVPVHFDAYV*

>Md-RNase4
MAMQKIGSLFLLLALLGLWLVQPSSGQNRRYQQFLRQHVDPSSEGGDSTYCNQMMHRRRMTEPRCKPVNSFIHEDIWNINSICRTTDIR
CKNGQMNCHEGIMKTTDCRVTGGSTSPNCRYRAMTRTRHVVIACEDLLPVHFDR*

>Md-RNase5

MVMFLKAMDPFLLLFILGLWLVPPSLTTQANAREKHFLIQHYDSKPKGRDDKYCTAIMRQRGLTQPCKDMNTFIHGDYPSIKAVCGDKA
GNPYEGGRLRISKSRFQVTNCEHRGGSTKPPCKYRATSDFRYIIIACENNLPVHLDQTIIAK*

>Md-RNase7/8
MASVLASPCPLLLLFLGLWEVPVSTKPENLTHAQWFQLQHVQPSPLKCNRAMGRINYYKQHCKRLNTFLHDSFPNVATVCESPSMACKN
GQENCHQSPGTISMTQCDLTSGKYPNCKYSNTALDRAFIVACDPPEAGDPLGYQLVPVHLEQTI*

>Md-RNase12
MEAKSLLFSSAPFPVGERTRRNVNGIFPLLVLLLLTFLLMLGLSKSLDEDLQEKSFEEEHIDYPKSNRLFRYCNSMMLLKKIRGPNDTC
KRKHVFIHERLDNIVSMCNNFTTITTCKYPSRMNCHHSQRKLQLTDCKLTEGRRFPGCKYQSLPKFNSILFNCDEIGPVYLYKIVEDS*

>Md-RNase13
MVLVRILVLQLLLVPALTFLSFTSKEAENFRAVNIDFPKVNFSKGFQGYCNGLMSYVRGRQKLLKNCPNVHYVLHAPWEKVKISCHKTD
SFCEDFNEYCSLSQDVFPITTCSRVKETPPTTCLYNETTSTQRVYLLCSRKYNAKPIHIVGLF*

>Md-RNase16
MIPERSLLLSLLTLMVLGLSHHSLAESQEDKFKRQHLDSGTKGNPGKKYCDQMMAKRGMTKRKCKPVNTFLHESYQNIENICHEPNVPC
ANKNMHNCHKSNHPMKITECHLSGGSKPGKCRYRMNNVKKNVTVACVGKTLKPVHLDPPK*

>Md-RNase17
MALDRAALLFPLLAMVVVGLVSSESAAEKFQRQHVDSKSLAFNDNTYCNHMMKIRGMTKGNCKVFNTFILESIWKIRAICWNKKTACKQ
KFFNCHMSREPLKVTECQIKGNPKDSPCKYQTINDKKCVTVACNGWPPLPVHFDSSKDDICQRLPTT*

>Md-RNase18
MTRREENFWQQHYNDQRSDEAIQRQMRLISMVKERCKPKNTIIHDSPDKIRDICTNPNSTNVRCRNGSDNCYEGPNPFSVTVCEDTGSI
YSVYPNCQYNCHKEEPVKVTVACENGKPVHFDAS*

>Md-RNase19
MIHTLKGTFPLLQLTLWVTMFLVTAELPSNFTPAQWFNKQHVQHPKTKASNDDIYCNNEMRQINNYTHRCKSFNTFLDYMLEDIINACF
TPNITCKNLQKNCHKSTLKVPITNCVLTSDHYPNCRYHGISKMAFFVIACNPPLLADHSKSKLLPVHLDSSTEGPGPILLSS*

>Md-RNase20
MAQNVMGNSQFLLLRLSLMVLLVYSQPPPGFNWAQWFNEQHVQYPKSNAPNDNQYCNAEMYRVNNHTNVCKAFNSFLHNQTQNIINVCL
NPNTPCKNSRTNCHNSTFRNDITECTLTRGQFPNCQYRGRAVMKYFVIACNSIVPRGPLLPVHLDNTF*

>Md-RNase21
MAQNVMGNSQLLLLGLSLLVLLVYSQTRPGWERFQRFKNQHVRYPKHLASNNDQYCMMEMKWVNDYNNACKGFNSFLHNQTQNIINVCF
HPNIFCRNNETNCHRSIFRNSITECRSAGGQYPDCYYNGTAVRKYFVVACIPISPSGRLLPVHLDSTFWNPGQVFLLPPP*

>Md-RNase22
MALETIHLPQSLLLLLLLLLSLLLPPGVCDIYGKETRIQRFWRFHVDNPKSLIPGGNTKYCTVMIRRRQISERGKCKRENTFIHAMAGA
ILDVCKAQSVKACKVGRKNCHTSDHSFKQTLCVLQGFGYYPNCAYVSFPKFKKISLACTGLPRVPVYVDP*

>Md-RNase23
MVYMALKMAFSLMILLMAQPIAGTIEYVEVTEEQHMGFGISWDQNPARNFQIKHITDGYFTNCTVMMNRVNRKGSCKSINTFIHSHKNE
VNKVCKEKGTFIDCKQNRNYKCCFISKIKFNLTICEEKESRKSCEYHALKKEAHVVLGCNNNNQPVHFQGYFCETKPYRVYMEKRNLND
HLPSTYMSGAQEE*

>Md-RNase24
MYAGMWTFFLALFLDLANFSYTNDFWRRHVDYPRSTPKEEDNYCNWMMERRGMVWGNKCVPGNTFIHEKNSTIENMCNRTPRPCKNHRN
QFCHTSVRSFSVSDCFLEGGSQPPYCKYWVLVQSRKISVFCQYELPFHLED*

>Md-RNase25
MVHAGMKTLFLLLLLDLTNFSQAFHFWNYHVDYPKTQVSGTPDQYCNVIMQRRGMITRNHCFQINAFIHETNSTLQNICKTSPKTCGGP
SWLLCHESSKPLKLTYCSTQSYARPPNCGYKAQAFSEKILVVCVRGSPIYLFQT*

>Md-RNase26
MSQAVMWTVFLLIFLDLIHFSQTQDFWTHHVDFPKTHVFDNPSQYCDLMIMNKGLVNNSDCKPFNSFIHESNKTIFNICADSYGSFQDP
NELNCYESTYPLRITDCFARCISNCYPRCSYVEIFHLAKIRVACVNGRPQRIIRQIYCPPFWIRYWVPYGYYTYP*

>Md-RNase28
MACAVIWIFFLVSFMHLANVSQSQDFWTRHVDYPRTNISRIPSKYCNGIMKQRGLEIHNSCVYFTTFIHEMNQTILNICANSNVHHQCP
NGLTCHISPYPIMVTDCFMKLDANFPDCRYRGKIYFTRIKVICVNGRPKYVFSQAYLPPF*

>Md-RNase29
MWILFLVSFMHLANVSQSQSFWTHHVDYPRTNISGSPSKYCKMMIRQRELKMHDSCLYFHTFIHETNQTLLNICANSNVHHECLNRLTC
HISHYPLMVTDCFKIAHPNVPDCRYRGKIYFTRIKVICVKGRPEYLIHFPDLKE*

>Md-RNase30
MSSAVKWTVFLLIILNLTHFYQAKDFWTLHVDYPKTKASGKPPEYCNNIMRQRGLINNTYLRPFHTFIHESNSTILNICANSTNPHYAS
FAPNSYESQNPLHLTECCIKTCSPNFVCTYKEVIRMSKIRLACLNGHPDHLITYINYSPTKILINLLFGGPRR*

>Md-RNase32
MDCAVMWILFLVSFMHLANVSQSQDFWKQHVDYPRTEVSGGPSKYCNAMVKQRGLEMQNSCLYSNIFIHETNHSIVNICENTNHCFPSF
KGLPCHRSLYPVLVTHCFIRLHSVFPHCRYRGEIYFARIKIICMNGKPGFII*

Platypusm (*Ornithorhynchus anatinus*)

>Oa-RNase4
MFWQGTYSLLLLALLALLGPALLPVSGQKTMYQRFLRQHVDPEGENGGDATAYCNRMMQARKMTAQRCKAFNTFIHEPIYKINSICSTD
SISCKNGRMNCHEGVVRVTDCREKGGSRPGNCRYRAQVSTRRVVIACEGDPQVPVHFDR*

>OaRNase13
MAPAPPRTSTLFPLFFILALDPVPAPALLPSLSSKARTFRLMHIDFPRSEFAPGFRGYCNGLMAYVRAQRESWQCPEKHFVLHAPAATV
RAICAHTDSFCEDFGEFCTRSHKPLPVTTCARTPGLPPSVCRYNATVHVQSHRVWLLCSSKFEGFPMDVIGVS*

>Oa-RNase33
MAATTKTPGHMLPQVLPLALLLLATCTFGQFAVFLQRHVDQPKSQVPGGPRQYCNVLMQRRRLNTGNHCKPLNTFIHENQGALVALCRT
PAGPCRNPKKKNCHRSPQRLNVTDCKVIPGSQPPNCRYHSLAQSRNIVVACVGGQPVHLDG*

>Oa-RNase34
MVATTMTPGRMLPQVLPLALLLLATCTFGQFAVFLQRHVDQPKSQVPGGSRQYCNVLMQRRRLNTGNRCKPLNTFIHENQGALVALCRT
PARRCRNPRMHNCHRSPQRLRVTDCRAIPGGQPPGCRYRSLARSRNIVVACVGGQPVHLDG*

>Oa-RNase35
MVLILMGTTHPLPLVALFLLLHLTLPSPPLIATFDDFYKCHIVEEGANIQDLDKFCTDKMMESKCSKYKKVNTFISEGVKTVIDICKEK
RDYCRNPKWKNCHNSSQELELINCCPNTFKDGTFLYQGFMKRRKISVACVCHQPVHFDAQFPYDSLCNSNYSYLPEAPPT*

Other miscellaneous sequences

The large flying fox (megabat, *Pteropus vampyrus*)

>Pv-RNase1
MAQAKSLILFSLLLLVLLALGWVEPFRERESQAKKFIRQHMDPDSYPNIGSSYCNQMMRRRDMTKGRCKPVNTFVHEPLVDVQAVCFQE
KVTCKDGQSDCYQSSSSMSITDCRLTNGSKYPNCAYRTSQKKRHIIVACNGNPYVPVHFDASVEVST*

>Pv-RNase4
MALQRILSLLLLLLLTLLGLGLLQPSYGQDHMYQRFLRQHVDSKVTNRNESYCNLLMQRRKMTSRYCKYFNTFIHEDIWKIINICSTTN
IQCRTGEMNCHESGV**MPQTAG**QVPRPPSADIGP*LVLDRLSLPVTVIRRCLCTLPD

>Pv-RNase5
MVMGLGPLLLVFMMGLGLTLPTLAQNNYRYELFLTQHCDANPSGRDKKYCESMMAKRGMTTPCKDRNTFIHASKKEIKAVCEDKNGTPY
NGGLRKSKTPFQVTICKHTGGSPRPPCYYKATSGSRDIVIACEKGWPVHFDESFYRP*

>Pv-RNase6
MMLDLLERLFLLLLLLGLWGPMCPLYALPRNLTKARWFKIQHIQPLYLQCDTAMSGVNTYTQVCKPQNTFLHDSFQNVAIACMSPNIFC
KNGRKNCHQSTKRVYMTNCNLTGGTYPACRYKEANQTKFFIIACDPPQKGDPPYQLVPVHLDKVI*

Tarsier (*Tarsius syrichta*)

>TarsierR2/3A
MVPKLLDSRLCLLLLLGLMGMEGSFHVKPSQFTWAQWFTIQHINMTHTRCDDAMRVVNGYLRRCKNRNTFLRTTFDDTAGVCGTPNITC
PSNNTMKNCHQSPDQVNIVDCNLTKSSKNITNCLYAQSSAQKYYVIACANRTNQEPSKYPLIPVHLDRIF*

>TarsierR2/3B
MIPKQLDSRFCLLLLLGLMKMIGSLCAAPGNLTRAQWFTIQHINMTHPKCDDAMRVVNGYTGRCKNQNTFLNTTFPNVVNVCLTPAIKC
ITSKSQNCHKSSVKVNLTYCNLTTPGTVQTCKYAQTEAEMFYIVACDNRSALDPPIYPVVPVHLDATVMLLTQDLVPTLNSQASQSRSL
LRHVFDTVWSFGHSLLQRLFPSST*

>TarsierR2/3C
MAPKLLSSRFCLLLLLGLMGIVGSSHAKPSHVTWAQWFNIQHVNMTHSRCDDAMQVVNGYEKRCKNLNTFLHTNFSSVVSVCNTQNITC
NNGRMNCHESTEQVPLTICNITRHSTNYRNCTYRQTQAQKRYIIACNNSAPQDAEYPAMVPVHLDRLT*

>TarsierR2/3D
MIPKQLDSRFCLLLLLGLMEVIGSLCTPAGNLTQAQWFTIQHINMTHPQCDDVMQVVNSYTRRCKNRNTFLNSIFPNVVDVCLTPAIKC
ITSQSQNCHNSSVKVNLTYCNLTTPRTVQTCRYAQTEAEMFYIVACDNRSALDPHIYRGSSSPGCNSNAPDPGLGANSQQPSFTI*

>TarsierR7/8A
MAPVRAGFCPLLLLLLLGLWVAMISVSAKPGHVTDAGWFKTQHVQNKRRSCKAAMENINKHTKHCKDRNTFLQTTFSKVAATCRTPSIAC
KDGQKNCHKSRGAVSMTLCEVTSGKYPNCKYKENHLKKRYIVACNPPQKGDSKKFKLVPVHLDNIL*

>TarsierR7/8B

MVPVRALSCPLLLFLLLGLWVAKIPINVKPTNTTSAQWFEIQHVQPSPQACNSAMGKINKDKKNCKNLNTFLHESFSSVATTCQTPSIA
CKNGRKNCHKSQEPMSLTQCEYTSGRYPDCKYKGKQLDAFFIVACEPPQEGDKKYSLVPVHLDDVV*

Bushbaby (*Otolemur garnettii*)

>Og-RNase2/3A
MVPKLLNSRFCLLLPLVLMAMVGSFHAKPPQFTWAQWFEIQHINMTNPQCTIAMRVINRYYPQCKRRNTFLHTTFAAVAGVCGTPNTPC
VSNSSRTNCHNSSVPVAITYCNLTSRPTPVANCRYTNTAAQSLYVVACNNRDPQRDSLLYPVVPVHLDAI*

>Og-RNase2/3B
MIPKLLDSQLCLLLLLGLVMMVGSFDAPPGNFTPTLWFQIQHVNMTNPQCTIAMRSVNHYKPQCKPRNTFLHTTYPDVVKVCGTPNIPC
PTNPTRKNCHNSSVPVSLTYCNLTSRPTPVANCRYSQTSAQMFYVVACDNRDPRDSPLYPVVPVHLDAIMAIILDQHDVPSLAHQVPPS
RRLCVSTFATLLLFSYNLF*

>Og-RNase2/3C
MVPKLLDSQLCLILLLGFVAMVGSFDAPPGNFTPTQWFRIQHVNMTNPRCTIAMRSVNHYKPQCKPRNTFLNTTYPDVVKVCGTPNIPC
PTNPTRKNCHKSSVPVRLTDCHLTSRPTPVKDCKYSDKPEEKFYVVACNNRDPRDSPLYPVVPVHLDAIVSPTLDQHDVPSLAHQVPPS
RRLCVSTFATLLLFSYNLF*

>Og-RNase7/8
MAPARTGFCPLLLLLLLLLGLWVAKVPVSAKPRHMTSSQWFEIQHVQPHPRACNSAMGNINKHTKHCKDRNTFLHESFSRVATTCQTPK
IACKNGHKNCHQSRGPVSLTLCERTSGKYPNCRYKEKHLNKSYIVACDSPQKRDSQPFPLVPVHLDKVL*

Tree shrew (*Tupaia belangeri*)

>Tb-RNase7/8
MAPVRLGFCHLLLLLLLLGLWVAEIPVSAKPKHVTSSQWFKIQHVQPSPEACNSAMGKINKDTKRCKNLNTFLHKSFSDVAATCQNPVKT
CKNKRKNCHKSRGPVSLTLCEHTSGKYPNCRYKEKHMKASYTVACDPPQKGDTRKFRLVPVHLEPL*
