# Supplementary material for: The Expansion and Functional Diversification of the Mammalian Ribonuclease A Superfamily Epitomizes the Efficiency of Multigene Families at Generating Biological Novelty
Source: Genome Biol Evol. 2013 Oct 25;5(11):2124–40. doi: 10.1093/gbe/evt161 (PMC3845642; doi:10.1093/gbe/evt161)

Supplementary Table 1. List of mammals included in this study

Binomial name	Common name	Order	Assembly	Release date	Coverage
Homo sapiens	Human	Primates	GRCh37/hg19	Feb. 2009	> 10 x
Pan troglodytes	Chimpanzee	Primates	CGSC2.1.3/panTro3	Oct. 2010	6 x
Gorilla gorilla	Gorilla	Primates	gorGor3.1	May 2011	2.1x WGS, 35x Solexa
Pongo pygmaeus	Orangutan	Primates	WUGSC2.0.2/ponAbe2	Jul. 2007	6 x
Nomascus leucogenys	Gibbon	Primates	GGSC1.0/nomLeu1	Jan. 2010	5.6 x
Macaca mulatta	Rhesus macaque	Primates	MGSC1.0/rheMac2	Jan. 2006	6 x
Callithrix jacchus	Marmoset	Primates	WUGSC3.2/calJac3	Mar. 2009	6 x
Mus musculus	Mouse	Rodents	NCBI37/mm9	Jul. 2007	> 10 x
Rattus norvegicus	Rat	Rodents	RGSC5.0/rn5	Mar. 2012	7 x
Heterocephalus glaber	Naked mole rat	Rodents	BGI1.0/hetGla1	Jul.2011	>20x Illumina
Cavia porcellus	Guinea pig	Rodents	Broad/cavPor3	Feb. 2008	6.79 x
Oryctolagus cuniculus	European rabbit	Lagomorpha	Broad/oryCun2	Apr. 2009	7.48 x
Bos taurus	Cow	Cetartiodactyla	Baylor4.6.1/bosTau7	Oct. 2007	7.1 x
Equus caballus	Horse	Perissodactyla	Broad/ecuCap2	Sep. 2007	6.79 x
Canis familiaris	Dog	Carnivora	Broad3.1/canFam3	May 2005	7.6 x
Ailuropoda melanoleuca	Giant panda	Carnivora	BGI1.0/ailMel1	Dec 2009	6.8 x
Myotis lucifugus	Little brown bat	Chiroptera	Broad/myoLuc2	Jul. 2010	7 x
Loxodonta africana	African elephant	Proboscidea	Broad/loxAfr3	Jul. 2009	7 x
Monodelphis domestica	Opossum	Didelphimorphia	Broad/monDom5	Oct. 2006	7.33 x
Ornithorhynchus anatinus	Platypus	Monotremata	WUGSC5.0.1/ornAna1	Mar. 2007	6 x

Supplementary Table 2. Chromosomal or scaffold coordinates for the RNase genes identified in the 20 mammals. Genes are ordered by their coordinates (A) and by their names (B). "NA" means the gene is not available in the genome sequence but the sequence entry exists in NCBI as a result of an independent submission.

A. Ordered by gene coordinates

Species	Gene	Chromosome	Strand	Start	End	Accession No.
Human (Hs)	RNase10	14	+	20978631	20979278	NM_001012975
	RNase9	14	-	21024614	21025228	NM_001001673
	RNase11	14	-	21052037	21052633	NM_145250
	RNase12	14	-	21058442	21058882	NM_001024822
	RNase5 (ANG)	14	+	21161724	21162167	NM_001145
	RNase4	14	+	21167531	21167974	NM_002937
	RNase6	14	+	21249859	21250308	NM_005615
	RNase1	14	-	21269757	21270227	NM_002933
	RNase14psA	14	-	21342562	21342990	This study
	RNase3 (ECP)	14	+	21359846	21360325	NM_002935
	RNase2ps (ECRP)	14	+	21387800	21388280	NR_033909
	RNase14psB	14	-	21403128	21403556	This study
	RNase2 (EDN)	14	+	21423931	21424413	NM_002934
	RNase15ps	14	-	21457103	21457513	This study
	RNase13	14	-	21501980	21502447	NM_001012264
	RNase7	14	+	21511152	21511619	NM_032572
	RNase8	14	+	21526052	21526513	NM_138331
Chimpanzee (Pt)	RNase10	14	+	19446047	19446697	XM_528692
	RNase9	14	-	19491347	19491964	NM_001009131
	RNase11	14	-	19519751	19520350	XM_001141127
	RNase12	14	-	19526133	19526576	This study
	RNase5	14	+	19641942	19642382	NM_001009159
	RNase4	14	+	19647737	19648180	NM_001009107
	RNase6	14	+	19726966	19727418	NM_001009132
	RNase1	14	-	19747465	19747935	NM_001009108
	RNase3	NA	NA	NA	NA	AF294027
	RNase14ps	14	-	19817779	19818209	This study
	RNase2	14	+	19840808	19841293	NM_001009133
	RNase15ps	14	-	19885418	19885828	This study
	RNase13	14	-	19930722	19931192	XM_001148573
	RNase7	14	+	19941187	19941657	DQ033127
	RNase8	14	+	19956632	19956976	NM_001009156
Gorilla (Gg)	RNase10	14	+	1429124	1429774	This study
	RNase9	14	-	1481839	1482456	AY263970
	RNase11	14	-	1509764	1510363	This study
	RNase12	14	-	1516173	1516616	This study
	RNase5	14	+	1622389	1622832	AF441662
	RNase4	14	+	1628185	1628628	AF078118
	RNase6	14	+	1708611	1709060	AF037088
	RNase1	14	-	1728937	1729407	AF449629
	RNase14psA	14	-	1805251	1805679	This study
	RNase3	14	+	1828348	1828827	U24097
	RNase2ps	14	+	1840021	1840575	This study
	RNase14psB	14	-	1865394	1865822	This study
	RNase2	14	+	1886741	1887223	U24100.
	RNase15ps	14	-	1921282	1921692	This study
	RNase13	14	-	1966849	1967319	This study

	RNase7	14	+	1976025	1976492	This study
	RNase8ps	14	+	1990978	1991416	JQ353683
Orangutan (Pp)	RNase10	14	+	19816078	19816728	XM_002824514
	RNase9	14	-	19858935	19859549	XM_002824540
	RNase11	14	-	19888839	19889438	XM_002824520
	RNase12	14	-	19895425	19895868	XM_002824521
	RNase5	14	+	20019031	20019474	AF441663
	RNase4	14	+	20024834	20025277	NM_001134203
	RNase6	14	+	20105624	20106076	AF037082
	RNase1	14	-	20125309	20125778	NM_001133338
	RNase14psA	14	-	20192960	20193385	This study
	RNase3	14	+	20210197	20210679	XM_002824527
	RNase14psB	14	-	20231371	20231799	This study
	RNase2	14	+	20253714	20254199	U24104
	RNase14psC	14	-	20261280	20261672	This study
	RNase2ps	14	+	20282367	20282847	This study
	RNase15ps	14	-	20327240	20327650	This study
	RNase13	14	-	20378013	20378483	XM_002824533
	RNase7	14	+	20387243	20387713	XM_002824534
	RNase8	14	+	20402279	20402743	EF100699
Gibbon (Nl)	RNase10	GL397514	+	998919	999569	XM_003280896
	RNase9	GL397514	-	1052213	1052827	XM_003280913
	RNase11	GL397514	-	1067193	1067783	XM_003280900
	RNase12	GL397514	-	1073523	1073962	XM_003280914
	RNase4	GL397514	+	1179741	1180184	XM_003280901
	RNase6	GL397607	+	70736	71185	XM_003282022
	RNase1	GL397607	-	90730	91200	XM_003282027
	RNase2	GL397280	+	155130	155612	AF479628
	RNase15ps	GL397280	-	213587	213999	This study
	RNase13	GL397280	-	260093	260563	XM_003260669
	RNase7	GL397280	+	268879	269346	XM_003260658
	RNase8ps	GL397280	+	283836	284300	XM_003260938
Rhesus macaque (Mmu)	RNase10	7	+	83214182	83214829	XM_001090353
	RNase9B	7	-	83271387	83272001	NM_001171839
	RNase9A	7	-	83277301	83277915	NM_001032844
	RNase11	7	-	83304804	83305403	XM_001091056
	RNase12	7	-	83311166	83311609	XM_001091180
	RNase5	7	+	83431110	83431550	AF441667
	RNase4	7	+	83436957	83437400	XM_001091656
	RNase6	7	+	83506081	83506533	XM_001092250
	RNase1	7	-	83531706	83532164	NM_001044738
	RNase3B	7	+	83597363	83597845	XM_002804975
	RNase14psA	7	-	83584267	83584696	This study
	RNase3A	7	+	83610301	83610783	XM_002804976
	RNase14psB	7	-	83628321	83628747	This study
	RNase2	7	+	83650329	83650811	XM_001094839
	RNase15ps	7	-	83701171	83701580	This study
	RNase13	7	-	83745761	83746231	XM_001094063
	RNase7	7	+	83755298	83755768	XM_001094178
	RNase8	NA	NA	NA	NA	AF473859
Marmoset (Cj)	RNase10	10	+	44888672	44889322	XM_002753710

	RNase9	10	-	44915772	44916350	This study
	RNase12	10	-	44934072	44934515	XM_002753729
	RNase5	10	+	45014561	45015001	XM_002753737
	RNase4	10	+	45020519	45020962	XM_002753711
	RNase6	10	+	45063219	45063671	XM_002753714
	RNase1	10	-	45071412	45071882	XM_002753742
	RNase2/3	10	+	45111201	45111677	AF479634
	RNase15ps	10	-	45153156	45153573	This study
	RNase13ps	10	-	45193387	45193742	This study
	RNase7	10	+	45203411	45203881	XM_002753764
	RNase8	10	+	45219089	45219556	XM_002753765
Mouse (Mm)	Ear1	14	-	44396607	44397074	NM_007894
	Ear-ps6	14	-	44431387	44431853	AY665813
	Angps2	14	+	44442582	44442982	NG_001343
	Ear-ps9	14	-	44452533	44452998	AY665816
	Ear-ps8	14	-	44479345	44479811	AY665815
	Angps3	14	+	44490657	44491094	AY665822
	Ear10	14	-	44500563	44501033	NM_053112
	Ang5	14	+	44540146	44540583	AY665820
	Ear-ps7	14	-	44546736	44547182	AY665814
	Ear-ps10	14	+	44569379	44569845	AY665817
	Ang6	14	-	44579491	44579934	AY665821
	Ear-ps2	14	+	44624652	44625122	NG_001503
	Angps1	14	-	44634643	44635081	NG_001342
	Ear-ps14	14	+	44652227	44652693	This study
	Ear2	14	+	44680552	44681022	NM_007895
	RNase10	14	+	51628953	51629579	AY226990
	RNase9	14	-	51658640	51659194	NM_183032
	RNase11	14	-	51669192	51669770	AY665823
	RNase12	14	-	51676458	51676895	AY665824
	Ang1	14	+	51721079	51721516	NM_007447
	RNase4	14	+	51724496	51724942	NM_021472
	RNase6	14	+	51749828	51750289	NM_030098
	RNase1	14	-	51765121	51765570	NM_011271
	Ear5	14	+	51782139	51782606	NM_019398
	Ear-ps1	14	-	51787639	51788100	NG_001492
	Ang2	14	-	51815161	51815598	NM_007449
	Ear14	14	+	51823370	51823831	AY665809
	Ear11	14	-	51875114	51875581	NM_053113
	Ear-ps3	14	+	52373182	52373632	AY665810
	Ang4	14	-	52383730	52384164	NM_177544
	Ear-ps4	14	-	52412977	52413428	AY665811
	Ear-ps5	14	+	52458663	52459139	AY665812
	Ear6	14	+	52473673	52474140	NM_053111
	RNase13	14	-	52541894	52542355	AY665825
Rat (Rn)						
	RNase10	15	+	31766010	31766618	NM_001012467
	RNase9	15	-	31798066	3179877	NM_001008561
	RNase11	15	-	31807084	31807656	NM_001012476
	RNase12	15	-	31813998	31814432	NM_001012209
	Ang1	15	+	31859205	31859642	NM_001006992
	RNase4	15	+	31859205	31859642	NM_020082
	RNase6	15	+	31891330	31891791	NM_206815
	RNase1-like1	15	-	31874776	31875225	NM_001013232

	RNase1	15	-	31897387	31897842	NM_001029904
	Ang2	15	-	31932544	31933047	NM_001012359
	RNase1-like2	15	+	31958343	31958798	NM_001025116
	RNase2	15	-	31984127	31984594	NM_001007015
	RNase16-like	15	-	32022338	32022808	XM_002728191
	R17	15	-	32034173	32034640	NM_001012232
	ECP-like	15	-	32046895	32047362	XM_002728192
	RNaseps	15	-	32068182	32068643	NG_004824
	Ear3-like	15	+	32073262	32073717	XM_002728193
	RNase13	15	-	32136026	32136487	NM_001012231
Naked mole rat (Hg)	RNase10	JH168595	+	308751	309401	EHB04622
	RNase9	JH168595	-	357124	357681	EHB04623
	RNase11ps	JH168595	-	372000	372589	This study
	RNase12	JH168595	-	380574	381011	EHB04624
	RNase4	JH168595	+	484733	485176	EHB04626
	RNase6A	JH168595	+	501046	501501	EHB04627
	RNase1	JH168595	-	505382	505852	This study
	RNase1psC	JH167839	+	43107	43563	This study
	RNase1psA	JH167839	-	127732	128205	This study
	RNase1psB	JH167839	-	129291	129764	This study
	RNase1psD	JH170059	-	1451450	1451919	This study
	RNase6D	JH167839	-	46362	46805	This study
	RNase6psC	JH167839	-	82766	83221	This study
	RNase6C	JH167839	+	108085	108534	EHB02899
	RNase6psB	JH170059	+	1488278	1488722	This study
	RNase6B	JH170059	+	1473967	1474410	EHB08179
	RNase6psA	JH170059	+	1442976	1443445	This study
	RNase13	JH170059	+	1365438	1365899	EHB08173
	RNase7/8	JH170059	-	1353532	1354005	EHB08172
	RNase7/8ps	JH170059	-	1335502	1335960	This study
Guinea pig (Cp)	RNase6B	13	+	21969554	21970009	This study
	RNase10	13	-	21542554	21543204	XM_003474345
	RNase11	13	+	21483738	21484331	XM_003474534
	RNase12	13	+	21471443	21471877	XM_003474533
	RNase4A	13	-	21233130	21233573	XM_003474532
	RNase1B	13	-	21153166	21153630	P00679
	RNase6psB	13	+	21149785	21150252	This study
	RNase1A	13	+	21103169	21103621	P00678
	RNase4B	13	+	21082192	21082635	XM_003474529
	RNase6psA	13	+	21042019	21042467	This study
	RNase1ps	13	-	21029347	21029820	This study
	RNase6A	13	+	21025263	21025826	XM_003474341
	RNase6psC	13	+	21006081	21006529	This study
	RNase6C	13	+	21005508	21005963	XM_003474527
	RNase1C	13	-	20972800	20973258	This study
	RNase13	13	+	20876598	20877071	XM_003474524
	RNase7/8	13	-	20866745	20867215	XM_003474523
	RNase7/8ps	13	-	20839375	20839836	This study
	RNase15ps	13	-	20382997	20383393	This study
Rabbit (Oc)	RNase10	17	+	40967360	40968010	This study
	RNase9	17	-	40994813	40995379	XM_002717871
	RNase11	17	-	41018269	41018865	XM_002718037
	RNase12	17	-	41024476	41024913	XM_002718038

	RNase2/3C	17	-	40703838	40704320	This study
	RNase5	17	+	41115613	41116062	XM_002717878
	RNase4	17	+	41123853	41124293	XM_002718039
	RNase5ps	17	+	41159071	41159565	This study
	RNase6	17	+	41176437	41176901	XM_002718040
	RNase1	17	-	41182928	41183371	XM_002718041
	RNase2/3A	17	+	41208900	41209364	This study
	RNase2/3B	17	+	41241555	41242037	This study
	RNase13	17	-	41339104	41339565	This study
	RNase7/8B	17	+	41350754	41351224	XM_002717883
	RNase7/8A	17	+	41368773	41369243	XM_002718045
Cow (Bt)	RNase10	10	-	25912713	25913348	XM_002690744
	RNase9B	10	+	25874098	25874658	Cho et al. (2006)
	RNase9A	10	+	25858107	25858655	Cho et al. (2006)
	RNase9ps	10	+	25846171	25846712	Cho et al. (2006)
	RNase11	10	+	25827997	25828593	XM_002690659
	RNase12	10	+	25822386	25822823	XM_001249856
	Ang2	10	-	25717965	25718408	NM_001099396
	Ang1	10	-	25713181	25713627	NM_001078144
	RNase4	10	-	25708682	25709125	BC102072
	RNase6	10	-	25686849	25687313	NM_174594
	RNase1_pancreatic	10	+	25672997	25673449	NM_181810
	RNase7/8B	10	-	25636123	25636629	XM_002690656
	Ang3	10	-	25605141	25605587	XM_002690654
	RNase4ps	10	-	25600646	25601144	XM_002690653
	RNase1_brain	10	+	25573344	25573847	NM_173891
	RNase1_seminal	10	+	25541009	25541461	X51337
	RNase14	10	+	25517439	25517864	XM_002690740
	RNase2/3A	10	-	25501430	25501906	Cho et al. (2006)
	RNase2/3B	NA	NA	NA	NA	NM_001105511
	RNase13	10	+	25400842	25401303	NM_001105426
	RNase7/8A	10	-	25388779	25389249	XM_002690738
	RNase15	10	-	24872725	24873144	XM_002690731
	RNase7/8ps	AAFC02015629	-	12251	12717	This study
Horse (Ec)	RNase10	1	+	157443285	157443926	NM_001163987
	RNase9	1	-	157494101	157494658	This study
	RNase11	1	-	157531357	157531956	XM_001505138
	RNase12	1	-	157535799	157536236	XM_001505139
	RNase5	1	+	157659668	157660108	NM_001081899
	RNase4	1	+	157664736	157665179	This study
	RNase6	1	+	157694597	157695061	This study
	RNase1	1	-	157713625	157714095	XM_001505141
	RNase4ps	1	-	157767278	157767719	This study
	RNase14ps	1	-	157782258	157782693	This study
	RNase2/3ps	1	+	157793046	157793519	This study
	RNase2/3	1	+	157827427	157827906	This study
	RNase7/8	1	+	157908452	157908958	XM_001502729
	RNase7/8ps	1	+	157921235	157921738	This study
	RNase15ps	1	+	158347613	158348030	This study
Dog (Cf)	RNase10	15	+	17876192	17876788	XM_539677
	RNase9	15	-	17876192	17876788	Cho et al. (2006)
	RNase11ps	15	-	17898082	17898755	This study
	RNase12	15	-	17904089	17904526	Cho et al. (2006)

	RNase5ps	15	+	17993080	17993540	Cho et al. (2006)
	RNase4	15	+	17997441	17997878	XM_848675
	RNase6ps	15	+	18008391	18008876	Cho et al. (2006)
	RNase6	15	+	18025082	18025570	Cho et al. (2006)
	RNase1	15	-	18037515	18037970	XM_532618
	RNase13	15	-	18123258	18123719	Cho et al. (2006)
Giant panda (Am)	RNase10	GL194214.1	-	103682	104329	XM_002929507
	RNase9	GL194214.1	+	69592	70197	This study
	RNase11	GL194214.1	+	48991	49587	XM_002929506
	RNase12	GL194214.1	+	44648	45082	XM_002929505
	RNase5	GL195202.1	+	54415	54855	This study
	RNase4	GL195202.1	+	59572	60015	XM_002930969
	RNase6A	GL195779.1	+	8716	9171	XM_002931239
	RNase6B	GL195728.1	+	8598	9158	This study
	RNase6C	GL197533.1	+	71	526	This study
	RNase6D	GL196462.1	+	1531	1986	This study
	RNase6E	GL196141.1	-	7482	7938	This study
	RNase1	GL193688.1	+	538057	538512	XM_002927826
	RNase14ps	GL193688.1	+	491437	491860	This study
	RNase13	GL193688.1	+	442362	442823	XM_002927824
	RNase7/8	GL193688.1	-	433142	433612	XM_002927831
	RNase7/8ps	GL193688.1	-	418991	419465	This study
Little brown bat (Ml)	RNase10	GL429805	-	1644401	1645054	This study
	RNase9	GL429805	+	1610275	1610838	This study
	RNase11	GL429805	+	1588237	1588830	This study
	RNase12	GL429805	+	1582888	1583325	This study
	RNase6psC	GL429805	-	1358265	1358728	This study
	RNase1A	GL429805	+	1352336	1352803	This study
	RNase5psA	GL429805	-	1323158	1323590	This study
	RNase4K	GL429805	-	1314738	1315175	This study
	RNase5B	GL429805	-	1285197	1285634	This study
	RNase5D	GL429805	+	1270717	1271157	This study
	RNase6psE	GL429805	+	1236887	1237361	This study
	RNase5psF	GL429805	+	1226211	1226624	This study
	RNase5psE	GL429805	-	1177671	1178086	This study
	RNase4C	GL429805	-	1169723	1170163	This study
	RNase4G	GL429805	-	1133735	1134175	This study
	RNase1E	GL429805	+	1094896	1095480	This study
	RNase5psB	GL429805	-	1057383	1057799	This study
	RNase4E	GL429805	-	1051668	1052105	This study
	RNase6psF	GL429805	-	1046545	1046995	This study
	RNase4F	GL429805	-	1001617	1002054	This study
	RNase6psG	GL429805	-	996090	996563	This study
	RNase5psD	GL429805	-	958155	958569	This study
	RNase4D	GL429805	-	952411	952848	This study
	RNase1B	GL429805	+	938436	938903	This study
	RNase5A	GL429805	-	908381	908821	This study
	RNase4A	GL429805	-	899646	900086	This study
	RNase6	GL429805	-	876189	876653	This study
	RNase1F	GL429805	+	857061	857528	HM228936
	RNase14ps	GL429805	+	845546	846012	This study
	RNase2/3A	GL429805	-	832374	832823	This study
	RNase13	GL429805	+	750513	750974	This study
	RNase7/8	GL429805	-	740305	740775	This study

	RNase7/8ps	GL429805	-	728734	729189	This study
	RNase15ps	GL429805	-	298278	298692	This study
	RNase4J	GL430627	+	4829	5260	This study
	RNase5G	GL430627	+	75124	75558	This study
	RNase5psI	GL430627	+	93544	93942	This study
	RNase1C	GL430627	-	116927	117394	This study
	RNase5psG	GL430627	+	142699	143131	This study
	RNase4psC	GL430627	+	152598	153034	This study
	RNase5F	GL430627	+	173262	173696	This study
	RNase4psA	GL430627	+	187338	187778	This study
	RNase2/3B	GL431682	-	3371	3835	This study
	RNase4B	GL431800	-	116	556	This study
	RNase5C	GL431800	-	11138	11578	This study
	RNase1D	GL431800	+	39022	39489	This study
	RNase5psC	GL431489	+	33188	33600	This study
	RNase4H	GL431489	+	38775	39212	This study
	RNase6psD	GL431489	+	43881	44355	This study
	RNase6psB	GL431836	+	6740	7203	This study
	RNase1G	GL431836	-	19453	19920	This study
	RNase4I	GL429805	+	1231785	1232225	This study
	RNase4psB	GL429831	-	1983149	1983587	This study
	RNase5E	GL432341	-	3524	3964	This study
	RNase5psH	AAPE02062487	+	14123	14536	This study
	RNase6psA	GL431561	+	15667	16131	This study
Elephant (La)	RNase10	118	-	1824896	1825546	XM_003421612
	RNase9	118	+	1782351	1782920	This study
	RNase11	118	+	1753496	1754119	XM_003421643
	RNase12	118	+	1747574	1748002	XM_003421611
	RNase5ps	118	-	1518558	1518999	This study
	RNase4	118	-	1510934	1511377	XM_003421642
	RNase6C	118	-	1490472	1490930	XM_003421606
	RNase1psB	118	+	1461079	1461586	This study
	RNase6B	118	-	1434081	1434545	XM_003421603
	RNase1psA	118	+	1410362	1410874	This study
	RNase6A	118	-	1380295	1380750	XM_003421602
	RNase1	118	+	1350854	1351357	XM_003421601
	RNase14ps	118	+	1286497	1286917	This study
	RNase2/3ps	118	-	1260407	1260870	This study
	RNase13	118	+	1180847	1181308	XM_003421638
	RNase7/8	118	-	1155027	1155494	This study
	RNase7/8ps	118	-	1137433	1137883	This study
Opossum (Md)						
	RNase12	1	-	168827765	168828298	Cho et al. (2006)
	RNase23	1	+	168850836	168851411	Cho et al. (2006)
	RNase21	1	-	168865043	168865552	Cho et al. (2006)
	RNase5	1	+	169065283	169065738	XM_001379291
	RNase4	1	+	169072452	169072883	XM_001368691
	RNase19	1	+	169130185	169130700	Cho et al. (2006)
	RNase20	1	+	169156512	169156985	Cho et al. (2006)
	RNase17	1	+	169180723	169181193	Cho et al. (2006)
	RNase1	1	-	169189243	169189695	XM_001368722
	RNase22	1	-	169244440	169244919	Cho et al. (2006)
	RNase25	1	-	169267262	169267693	Cho et al. (2006)
	RNase30	1	-	169296786	169297274	Cho et al. (2006)

	RNase26	1	-	169319078	169319572	Cho et al. (2006)
	RNase32	1	-	169341963	169342388	Cho et al. (2006)
	RNase28	1	-	169348555	169349004	Cho et al. (2006)
	RNase29	1	-	169353642	169354073	Cho et al. (2006)
	RNase16	1	-	169498851	169499300	Cho et al. (2006)
	RNase24	1	-	169539657	169540079	Cho et al. (2006)
	RNase13ps	1	-	169598435	169598899	Cho et al. (2006)
	RNase7/8	1	+	169623910	169624371	XM_001379385
	RNase18	1	+	169665957	169666328	Cho et al. (2006)
	RNase27ps	1	+	169673664	169674105	Cho et al. (2006)
	RNase13	1	+	243417179	243417637	Cho et al. (2006)
Platypus (Oa)	RNase4	Ultra560	+	16953	17399	XM_001505292
	RNase13	Contig31936	-	2629	3117	XM_001515543
	RNase33	Contig23782	+	8449	8898	XM_001516892
	RNase34	Contig5065	-	4784	5236	XM_001521507
	RNase35	4	+	22016214	22016723	This study

B. Ordered by gene names

Species	Gene	Chromosome	Strand	Start	End	Accession No.
Human (Hs)	RNase1	14	-	21269757	21270227	NM_002933
	RNase2 (EDN)	14	+	21423931	21424413	NM_002934
	RNase2ps (ECRP)	14	+	21387800	21388280	NR_033909
	RNase3 (ECP)	14	+	21359846	21360325	NM_002935
	RNase4	14	+	21167531	21167974	NM_002937
	RNase5 (ANG)	14	+	21161724	21162167	NM_001145
	RNase6	14	+	21249859	21250308	NM_005615
	RNase7	14	+	21511152	21511619	NM_032572
	RNase8	14	+	21526052	21526513	NM_138331
	RNase9	14	-	21024614	21025228	NM_001001673
	RNase10	14	+	20978631	20979278	NM_001012975
	RNase11	14	-	21052037	21052633	NM_145250
	RNase12	14	-	21058442	21058882	NM_001024822
	RNase13	14	-	21501980	21502447	NM_001012264
	RNase14psA	14	-	21342562	21342990	This study
	RNase14psB	14	-	21403128	21403556	This study
	RNase15ps	14	-	21457103	21457513	This study
Chimpanzee (Pt)	RNase1	14	-	19747465	19747935	NM_001009108
	RNase2	14	+	19840808	19841293	NM_001009133
	RNase3	NA	NA	NA	NA	AF294027
	RNase4	14	+	19647737	19648180	NM_001009107
	RNase5	14	+	19641942	19642382	NM_001009159
	RNase6	14	+	19726966	19727418	NM_001009132
	RNase7	14	+	19941187	19941657	DQ033127
	RNase8	14	+	19956632	19956976	NM_001009156
	RNase9	14	-	19491347	19491964	NM_001009131
	RNase10	14	+	19446047	19446697	XM_528692
	RNase11	14	-	19519751	19520350	XM_001141127
	RNase12	14	-	19526133	19526576	This study
	RNase13	14	-	19930722	19931192	XM_001148573
	RNase14ps	14	-	19817779	19818209	This study
	RNase15ps	14	-	19885418	19885828	This study
Gorilla (Gg)	RNase1	14	-	1728937	1729407	AF449629
	RNase2	14	+	1886741	1887223	U24100.
	RNase2ps	14	+	1840021	1840575	This study
	RNase3	14	+	1828348	1828827	U24097
	RNase4	14	+	1628185	1628628	AF078118
	RNase5	14	+	1622389	1622832	AF441662
	RNase6	14	+	1708611	1709060	AF037088
	RNase7	14	+	1976025	1976492	This study
	RNase8ps	14	+	1990978	1991416	JQ353683
	RNase9	14	-	1481839	1482456	AY263970
	RNase10	14	+	1429124	1429774	This study
	RNase11	14	-	1509764	1510363	This study
	RNase12	14	-	1516173	1516616	This study
	RNase13	14	-	1966849	1967319	This study
	RNase14psA	14	-	1805251	1805679	This study
	RNase14psB	14	-	1865394	1865822	This study
	RNase15ps	14	-	1921282	1921692	This study
Orangutan (Pp)	RNase1	14	-	20125309	20125778	NM_001133338

	RNase2	14	+	20253714	20254199	U24104
	RNase2ps	14	+	20282367	20282847	This study
	RNase3	14	+	20210197	20210679	XM_002824527
	RNase4	14	+	20024834	20025277	NM_001134203
	RNase5	14	+	20019031	20019474	AF441663
	RNase6	14	+	20105624	20106076	AF037082
	RNase7	14	+	20387243	20387713	XM_002824534
	RNase8	14	+	20402279	20402743	EF100699
	RNase9	14	-	19858935	19859549	XM_002824540
	RNase10	14	+	19816078	19816728	XM_002824514
	RNase11	14	-	19888839	19889438	XM_002824520
	RNase12	14	-	19895425	19895868	XM_002824521
	RNase13	14	-	20378013	20378483	XM_002824533
	RNase14psA	14	-	20231371	20231799	This study
	RNase14psB	14	-	20192960	20193385	This study
	RNase14psC	14	-	20261280	20261672	This study
	RNase15ps	14	-	20327240	20327650	This study
Gibbon (Nl)	RNase1	GL397607	-	90730	91200	XM_003282027
	RNase2	GL397280	+	155130	155612	AF479628
	RNase4	GL397514	+	1179741	1180184	XM_003280901
	RNase6	GL397607	+	70736	71185	XM_003282022
	RNase7	GL397280	+	268879	269346	XM_003260658
	RNase8ps	GL397280	+	268858	284300	XM_003260938
	RNase9	GL397514	-	1052213	1052827	XM_003280913
	RNase10	GL397514	+	998919	999569	XM_003280896
	RNase11	GL397514	-	1067193	1067783	XM_003280900
	RNase12	GL397514	-	1073523	1073962	XM_003280914
	RNase13	GL397280	-	260093	260563	XM_003260669
	RNase15ps	GL397280	-	213587	213999	This study
Rhesus macaque (Mmu)	RNase1	7	-	83531706	83532164	NM_001044738
	RNase2	7	+	83650329	83650811	XM_001094839
	RNase3A	7	+	83610301	83610783	XM_002804976
	RNase3B	7	+	83597363	83597845	XM_002804975
	RNase4	7	+	83436957	83437400	XM_001091656
	RNase5	7	+	83431110	83431550	AF441667
	RNase6	7	+	83506081	83506533	XM_001092250
	RNase7	7	+	83755298	83755768	XM_001094178
	RNase8	NA	NA	NA	NA	AF473859
	RNase9A	7	-	83277301	83277915	NM_001032844
	RNase9B	7	-	83271387	83272001	NM_001171839
	RNase10	7	+	83214182	83214829	XM_001090353
	RNase11	7	-	83304804	83305403	XM_001091056
	RNase12	7	-	83311166	83311609	XM_001091180
	RNase13	7	-	83745761	83746231	XM_001094063
	RNase14psA	7	-	83628321	83628747	This study
	RNase14psB	7	-	83584267	83584696	This study
	RNase16ps	7	-	83701171	83701580	This study
Marmoset (Cj)	RNase1	10	-	45071412	45071882	XM_002753742
	RNase2/3	10	+	45111201	45111677	AF479634
	RNase4	10	+	45020519	45020962	XM_002753711
	RNase5	10	+	45014561	45015001	XM_002753737
	RNase6	10	+	45063219	45063671	XM_002753714

	RNase7	10	+	45203411	45203881	XM_002753764
	RNase8	10	+	45219089	45219556	XM_002753765
	RNase9	10	-	44915772	44916350	This study
	RNase10	10	+	44888672	44889322	XM_002753710
	RNase12	10	-	44934072	44934515	XM_002753729
	RNase13ps	10	-	45193387	45193742	This study
	RNase15ps	10	-	45153156	45153573	This study
Mouse (Mm)	RNase1	14	-	51765121	51765570	NM_011271
	Ear1	14	-	44396607	44397074	NM_007894
	Ear2	14	+	44680552	44681022	NM_007895
	Ear5	14	+	51782139	51782606	NM_019398
	Ear6	14	+	52473673	52474140	NM_053111
	Ear10	14	-	44500563	44501033	NM_053112
	Ear11	14	-	51875114	51875581	NM_053113
	Ear14	14	+	51823370	51823831	AY665809
	Ear-ps1	14	-	51787639	51788100	NG_001492
	Ear-ps2	14	+	44624652	44625122	NG_001503
	Ear-ps3	14	+	52373182	52373632	AY665810
	Ear-ps4	14	-	52412977	52413428	AY665811
	Ear-ps5	14	+	52458663	52459139	AY665812
	Ear-ps6	14	-	44431387	44431853	AY665813
	Ear-ps7	14	-	44546736	44547182	AY665814
	Ear-ps8	14	-	44479345	44479811	AY665815
	Ear-ps9	14	-	44452533	44452998	AY665816
	Ear-ps10	14	+	44569379	44569845	AY665817
	Ear-ps14	14	+	44652227	44652693	This study
	RNase4	14	+	51724496	51724942	NM_021472
	Ang1	14	+	51721079	51721516	NM_007447
	Ang2	14	-	51815161	51815598	NM_007449
	Ang4	14	-	52383730	52384164	NM_177544
	Ang5	14	+	44540146	44540583	AY665820
	Ang6	14	-	44579491	44579934	AY665821
	Angps1	14	-	44634643	44635081	NG_001342
	Angps2	14	+	44442582	44442982	NG_001343
	Angps3	14	+	44490657	44491094	AY665822
	RNase6	14	+	51749828	51750289	NM_030098
	RNase9	14	-	51658640	51659194	NM_183032
	RNase10	14	+	51628953	51629579	AY226990
	RNase11	14	-	51669192	51669770	AY665823
	RNase12	14	-	51676458	51676895	AY665824
	RNase13	14	-	52541894	52542355	AY665825
Rat (Rn)	RNase1	15	-	31897387	31897842	NM_001029904
	RNase1-like1	15	-	31874776	31875225	NM_001013232
	RNase1-like2	15	+	31958343	31958798	NM_001025116
	Ear3-like	15	+	32073262	32073717	XM_002728193
	R17	15	-	32034173	32034640	NM_001012232
	RNase2	15	-	31984127	31984594	NM_001007015
	ECP-like	15	-	32046895	32047362	XM_002728192
	RNase16-like	15	-	32022338	32022808	XM_002728191
	RNaseps	15	-	32068182	32068643	NG_004824
	RNase4	15	+	31859205	31859642	NM_020082
	Ang1	15	+	31859205	31859642	NM_001006992
	Ang2	15	-	31932544	31933047	NM_001012359
	RNase6	15	+	31891330	31891791	NM_206815

	RNase9	15	-	31798066	3179877	NM_001008561
	RNase10	15	+	31766010	31766618	NM_001012467
	RNase11	15	-	31807084	31807656	NM_001012476
	RNase12	15	-	31813998	31814432	NM_001012209
	RNase13	15	-	32136026	32136487	NM_001012231
Naked mole rat (Hg)	RNase1	JH168595	-	505382	505852	This study
	RNase1psA	JH167839	-	127732	128205	This study
	RNase1psB	JH167839	-	129291	129764	This study
	RNase1psC	JH167839	+	43107	43563	This study
	RNase1psD	JH170059	-	1451450	1451919	This study
	RNase4	JH168595	+	484733	485176	EHB04626
	RNase6A	JH168595	+	501046	501501	EHB04627
	RNase6B	JH170059	+	1473967	1474410	EHB08179
	RNase6C	JH167839	+	108085	108534	EHB02899
	RNase6D	JH167839	-	46362	46805	This study
	RNase6psA	JH170059	+	1442976	1443445	This study
	RNase6psB	JH170059	+	1488278	1488722	This study
	RNase6psC	JH167839	-	82766	83221	This study
	RNase7/8	JH170059	-	1353532	1354005	EHB08172
	RNase7/8ps	JH170059	-	1335502	1335960	This study
	RNase9	JH168595	-	357124	357681	EHB04623
	RNase10	JH168595	+	308751	309401	EHB04622
	RNase11ps	JH168595	-	372000	372589	This study
	RNase12	JH168595	-	380574	381011	EHB04624
	RNase13	JH170059	+	1365438	1365899	EHB08173
Guinea pig (Cp)	RNase1A	13	+	21103169	21103621	P00678
	RNase1B	13	-	21153166	21153630	P00679
	RNase1C	13	-	20972800	20973258	This study
	RNase1ps	13	-	21029347	21029820	This study
	RNase4A	13	-	21233130	21233573	XM_003474532
	RNase4B	13	+	21082192	21082635	XM_003474529
	RNase6A	13	+	21025263	21025826	XM_003474341
	RNase6B	13	+	21969554	21970009	This study
	RNase6C	13	+	21005508	21005963	XM_003474527
	RNase6psA	13	+	21042019	21042467	This study
	RNase6psB	13	+	21149785	21150252	This study
	RNase6psC	13	+	21006081	21006529	This study
	RNase7/8	13	-	20866745	20867215	XM_003474523
	RNase7/8ps	13	-	20839375	20839836	This study
	RNase10	13	-	21542554	21543204	XM_003474345
	RNase11	13	+	21483738	21484331	XM_003474534
	RNase12	13	+	21471443	21471877	XM_003474533
	RNase13	13	+	20876598	20877071	XM_003474524
	RNase15ps	13	-	20382997	20383393	This study
Rabbit (Oc)	RNase1	17	-	41182928	41183371	XM_002718041
	RNase2/3A	17	+	41208900	41209364	This study
	RNase2/3B	17	+	41241555	41242037	This study
	RNase2/3C	17	-	40703838	40704320	This study
	RNase4	17	+	41123853	41124293	XM_002718039
	RNase5	17	+	41115613	41116062	XM_002717878
	RNase5ps	17	+	41159071	41159565	This study
	RNase6	17	+	41176437	41176901	XM_002718040
	RNase7/8A	17	+	41368773	41369243	XM_002718045

	RNase7/8B	17	+	41350754	41351224	XM_002717883
	RNase9	17	-	40994813	40995379	XM_002717871
	RNase10	17	+	40967360	40968010	This study
	RNase11	17	-	41018269	41018865	XM_002718037
	RNase12	17	-	41024476	41024913	XM_002718038
	RNase13	17	-	41339104	41339565	This study
Cow (Bt)	RNase1_brain	10	+	25573344	25573847	NM_173891
	RNase1_seminal	10	+	25541009	25541461	X51337
	RNase1_pancreatic	10	+	25672997	25673449	NM_181810
	RNase2/3A	10	-	25501430	25501906	Cho et al. (2006)
	RNase2/3B	NA	NA	NA	NA	NM_001105511
	RNase4	10	-	25708682	25709125	BC102072
	RNase4ps	10	-	25600646	25601144	XM_002690653
	Ang1	10	-	25713181	25713627	NM_001078144
	Ang2	10	-	25717965	25718408	NM_001099396
	Ang3	10	-	25605141	25605587	XM_002690654
	RNase6	10	-	25686849	25687313	NM_174594
	RNase7/8A	10	-	25388779	25389249	XM_002690738
	RNase7/8B	10	-	25636123	25636629	XM_002690656
	RNase7/8ps	AAFC02015629	-	12251	12717	This study
	RNase9A	10	+	25858107	25858655	Cho et al. (2006)
	RNase9B	10	+	25874098	25874658	Cho et al. (2006)
	RNase9ps	10	+	25846171	25846712	Cho et al. (2006)
	RNase10	10	-	25912713	25913348	XM_002690744
	RNase11	10	+	25827997	25828593	XM_002690659
	RNase12	10	+	25822386	25822823	XM_001249856
	RNase13	10	+	25400842	25401303	NM_001105426
	RNase14	10	+	25517439	25517864	XM_002690740
	RNase15	10	-	24872725	24873144	XM_002690731
Horse (Ec)	RNase1	1	-	157713625	157714095	XM_001505141
	RNase2/3	1	+	157827427	157827906	This study
	RNase2/3ps	1	+	157793046	157793519	This study
	RNase4	1	+	157664736	157665179	This study
	RNase4ps	1	-	157767278	157767719	This study
	RNase5	1	+	157659668	157660108	NM_001081899
	RNase6	1	+	157694597	157695061	This study
	RNase7/8	1	+	157908452	157908958	XM_001502729
	RNase7/8ps	1	+	157921235	157921738	This study
	RNase9	1	-	157494101	157494658	This study
	RNase10	1	+	157443285	157443926	NM_001163987
	RNase11	1	-	157531357	157531956	XM_001505138
	RNase12	1	-	157535799	157536236	XM_001505139
	RNase14ps	1	-	157782258	157782693	This study
	RNase15ps	1	+	158347613	158348030	This study
Dog (Cf)	RNase1	15	-	18037515	18037970	XM_532618
	RNase4	15	+	17997441	17997878	XM_848675
	RNase5ps	15	+	17993080	17993540	Cho et al. (2006)
	RNase9	15	-	17876192	17876788	Cho et al. (2006)
	RNase10	15	+	17876192	17876788	XM_539677
	RNase11ps	15	-	17898082	17898755	This study
	RNase12	15	-	17904089	17904526	Cho et al. (2006)
	RNase13	15	-	18123258	18123719	Cho et al. (2006)
	RNase6	15	+	18025082	18025570	Cho et al. (2006)

	RNase6ps	15	+	18025082	18025570	Cho et al. (2006)
Giant panda (Am)	RNase1	GL193688.1	+	538057	538512	XM_002927826
	RNase4	GL195202.1	+	59572	60015	XM_002930969
	RNase5	GL195202.1	+	54415	54855	This study
	RNase6A	GL195779.1	+	8716	9171	XM_002931239
	RNase6B	GL195728.1	+	8598	9158	This study
	RNase6C	GL197533.1	+	71	526	This study
	RNase6D	GL196462.1	+	1531	1986	This study
	RNase6E	GL196141.1	-	7482	7938	This study
	RNase7/8	GL193688.1	-	433142	433612	XM_002927831
	RNase7/8ps	GL193688.1	-	418991	419465	This study
	RNase9	GL194214.1	+	69592	70197	This study
	RNase10	GL194214.1	-	103682	104329	XM_002929507
	RNase11	GL194214.1	+	48991	49587	XM_002929506
	RNase12	GL194214.1	+	44648	45082	XM_002929505
	RNase13	GL193688.1	+	442362	442823	XM_002927824
	RNase14ps	GL193688.1	+	491437	491860	This study
Little brown bat (Ml)	RNase1A	GL429805	+	1352336	1352803	This study
	RNase1B	GL429805	+	938436	938903	This study
	RNase1C	GL430627	-	116927	117394	This study
	RNase1D	GL431800	+	39022	39489	This study
	RNase1E	GL429805	+	1094896	1095480	This study
	RNase1F	GL429805	+	857061	857528	HM228936
	RNase1G	GL431836	-	19453	19920	This study
	RNase2/3A	GL429805	-	832374	832823	This study
	RNase2/3B	GL431682	-	3371	3835	This study
	RNase4A	GL429805	-	899646	900086	This study
	RNase4B	GL431800	-	116	556	This study
	RNase4C	GL429805	-	1169723	1170163	This study
	RNase4D	GL429805	-	952411	952848	This study
	RNase4E	GL429805	-	1051668	1052105	This study
	RNase4F	GL429805	-	1001617	1002054	This study
	RNase4G	GL429805	-	1133735	1134175	This study
	RNase4H	GL431489	+	38775	39212	This study
	RNase4I	GL429805	+	1231785	1232225	This study
	RNase4J	GL430627	+	4829	5260	This study
	RNase4K	GL429805	-	1314738	1315175	This study
	RNase4psA	GL430627	+	187338	187778	This study
	RNase4psB	GL429831	-	1983149	1983587	This study
	RNase4psC	GL430627	+	152598	153034	This study
	RNase5A	GL429805	-	908381	908821	This study
	RNase5B	GL429805	-	1285197	1285634	This study
	RNase5C	GL431800	-	11138	11578	This study
	RNase5D	GL429805	+	1270717	1271157	This study
	RNase5E	GL432341	-	3524	3964	This study
	RNase5F	GL430627	+	173262	173696	This study
	RNase5G	GL430627	+	75124	75558	This study
	RNase5psA	GL429805	-	1323158	1323590	This study
	RNase5psB	GL429805	-	1057383	1057799	This study
	RNase5psC	GL431489	+	33188	33600	This study
	RNase5psD	GL429805	-	958155	958569	This study
	RNase5psE	GL429805	-	1177671	1178086	This study
	RNase5psF	GL429805	+	1226211	1226624	This study
	RNase5psG	GL430627	+	142699	143131	This study

	RNase5psH	AAPE02062487	+	14123	14536	This study
	RNase5psI	GL430627	+	93544	93942	This study
	RNase6	GL429805	-	876189	876653	This study
	RNase6psA	GL431561	+	15667	16131	This study
	RNase6psB	GL431836	+	6740	7203	This study
	RNase6psC	GL429805	-	1358265	1358728	This study
	RNase6psD	GL431489	+	43881	44355	This study
	RNase6psE	GL429805	+	1236887	1237361	This study
	RNase6psF	GL429805	-	1046545	1046995	This study
	RNase6psG	GL429805	-	996090	996563	This study
	RNase7/8	GL429805	-	740305	740775	This study
	RNase7/8ps	GL429805	-	728734	729189	This study
	RNase9	GL429805	+	1610275	1610838	This study
	RNase10	GL429805	-	1644401	1645054	This study
	RNase11	GL429805	+	1588237	1588830	This study
	RNase12	GL429805	+	1582888	1583325	This study
	RNase13	GL429805	+	750513	750974	This study
	RNase14ps	GL429805	+	845546	846012	This study
	RNase15ps	GL429805	-	298278	298692	This study
Elephant (La)	RNase1	118	+	1350854	1351357	XM_003421601
	RNase1psA	118	+	1410362	1410874	This study
	RNase1psB	118	+	1461079	1461586	This study
	RNase2/3ps	118	-	1260407	1260870	This study
	RNase4	118	-	1510934	1511377	XM_003421642
	RNase5ps	118	-	1518558	1518999	This study
	RNase6A	118	-	1380295	1380750	XM_003421602
	RNase6B	118	-	1434081	1434545	XM_003421603
	RNase6C	118	-	1490472	1490930	XM_003421606
	RNase7/8	118	-	1155027	1155494	This study
	RNase7/8ps	118	-	1137433	1137883	This study
	RNase9	118	+	1782351	1782920	This study
	RNase10	118	-	1824896	1825546	XM_003421612
	RNase11	118	+	1753496	1754119	XM_003421643
	RNase12	118	+	1747574	1748002	XM_003421611
	RNase13	118	+	1180847	1181308	XM_003421638
	RNase14ps	118	+	1286497	1286917	This study
Opossum (Md)	RNase1	1	-	169189243	169189695	XM_001368722
	RNase4	1	+	169072452	169072883	XM_001368691
	RNase5	1	+	169065283	169065738	XM_001379291
	RNase7/8	1	+	169623910	169624371	XM_001379385
	RNase12	1	-	168827765	168828298	Cho et al. (2006)
	RNase13	1	+	243417179	243417637	Cho et al. (2006)
	RNase13ps	1	-	169598435	169598899	Cho et al. (2006)
	RNase16	1	-	169498851	169499300	Cho et al. (2006)
	RNase17	1	+	169180723	169181193	Cho et al. (2006)
	RNase18	1	+	169665957	169666328	Cho et al. (2006)
	RNase19	1	+	169130185	169130700	Cho et al. (2006)
	RNase20	1	+	169156512	169156985	Cho et al. (2006)
	RNase21	1	-	168865043	168865552	Cho et al. (2006)
	RNase22	1	-	169244440	169244919	Cho et al. (2006)
	RNase23	1	+	168850836	168851411	Cho et al. (2006)
	RNase24	1	-	169539657	169540079	Cho et al. (2006)
	RNase25	1	-	169267262	169267693	Cho et al. (2006)
	RNase26	1	-	169319078	169319572	Cho et al. (2006)

	RNase27ps	1	+	169673664	169674105	Cho et al. (2006)
	RNase28	1	-	169348555	169349004	Cho et al. (2006)
	RNase29	1	-	169353642	169354073	Cho et al. (2006)
	RNase30	1	-	169296786	169297274	Cho et al. (2006)
	RNase32	1	-	169341963	169342388	Cho et al. (2006)
Platypus (Oa)	RNase4	Ultra560	+	16953	17399	XM_001505292
	RNase13	Contig31936	-	2629	3117	XM_001515543
	RNase33	Contig23782	+	8449	8898	XM_001516892
	RNase34	Contig5065	-	4784	5236	XM_001521507
	RNase35	4	+	22016214	22016723	This study

Supplementary Table 3. The average d_S, d_N, and d_N/d_S values and their standard deviations of all possible ortholog pairs for 11 RNase gene lineages. RNase14 and RNase15 are not included here because they are present in only one species, the cow.

Gene lineages	Ave. d_S (s.d.)	Ave. d_N (s.d.)	Ave. d_N/d_S (s.d.)	Number of the eutherian species that it is present in
RNase1	0.272 (0.101)	0.157 (0.067)	0.587 (0.174)	18 (all)
RNase2/3	0.363 (0.159)	0.257 (0.115)	0.684 (0.179)	13
RNase4	0.262 (0.111)	0.085 (0.033)	0.363 (0.158)	18 (all)
RNase5	0.203 (0.085)	0.154 (0.052)	0.840 (0.312)	13
RNase6	0.296 (0.115)	0.213 (0.103)	0.701 (0.181)	18 (all)
RNase7/8	0.214 (0.086)	0.156 (0.066)	0.726 (0.183)	15
RNase9	0.334 (0.134)	0.309 (0.124)	0.914 (0.169)	17
RNase10	0.284 (0.110)	0.150 (0.056)	0.546 (0.122)	18 (all)
RNase11	0.242 (0.103)	0.205 (0.098)	0.846 (0.193)	15
RNase12	0.265 (0.108)	0.148 (0.054)	0.578 (0.163)	18 (all)
RNase13	0.292 (0.112)	0.180 (0.067)	0.643 (0.188)	16

Supplementary Table 4. The isoelectric points (*pI*) and the numbers of charged amino acids of the little brown bat RNase4 proteins and the human RNase2, RNase3, and RNase4 proteins. Only mature part, excluding the signal sequence, was used for calculating *pI* values and amino acid counts.

**Gene name**	***pI***	**Arg (R)**	**Lys (K)**	**R+K**	**Asp (D)**	**Glu (E)**	**D+E**
Hs-RNase2	9.20	8	4	12	4	1	5
Hs-RNase3	10.47	18	1	19	6	0	6
Hs-RNase4	9.18	13	5	18	6	5	11
Ml-RNase4A	9.35	12	5	17	6	3	9
Ml-RNase4B	9.35	12	5	17	6	3	9
Ml-RNase4C	8.93	12	2	14	6	3	9
Ml-RNase4D	9.92	15	3	18	3	4	7
Ml-RNase4E	10.82	17	5	22	3	3	6
Ml-RNase4F	9.01	13	6	19	8	5	13
Ml-RNase4G	8.84	10	6	16	8	3	11
Ml-RNase4H	10.75	17	2	19	3	2	5
Ml-RNase4I	9.30	9	8	15	5	4	9
Ml-RNase4J	9.73	14	8	22	4	7	11
Ml-RNase4K	9.21	12	2	14	3	5	8

Supplementary Figure Legends

Supplementary Figure 1. Pairwise synonymous (d_S) and nonsynonymous (d_N) nucleotide distances between orthologous RNase1 (A), RNase2/3 (B), RNase4 (C), RNase5 (D), RNase6 (E), RNase7/8 (F), RNase9 (G), RNase10 (H), RNase11 (I), RNase12 (J), RNase13 (K), and alpha-amylase (L) genes. The alpha-amylase gene is included to show an extreme level of d_N saturation during the evolution of this highly conserved gene.

Supplementary Figure 2. Phylogenetic trees of functional genes and pseudogenes of the five non-canonical RNase gene lineages, RNase14 and RNase15. For all trees, the Neighbor-Joining method with Kimura's two-parameter, the complete-deletion option, and 1,000 bootstrap replications were used. Trees for RNase9 (A), RNase10 (B), RNase11 (C), RNase12 (D), RNase13 (E), and RNases14 and 15 (F) were made with 500, 603, 536, 423, 352, and 294 nucleotide sites, respectively. Pseudogenes are distinguished with gray color. The scale bar number indicates the number of nucleotide substitutions per site.

Supplementary Figure 3. Phylogenetic trees of RNase2/3 (A) and RNase7/8 (B) genes in primates. Arrows in (A) and (B) indicate the time of duplication of RNase2 and RNase3 (A) and that of RNase7 and RNase8 (B), respectively. Mne: southern pig-tailed macaque (*Macaca nemestrina*), Mfa: crab-eating macaque (*Macaca fascicularis*), Cae: African green monkey (*Chlorocebus aethiops*), Soe: cotton-top tamarin (*Saguinus oedipus*), Sla: red-chested mustached tamarin (*Saguinus labiatus*), Atr: owl monkey (*Aotus trivirgatus*), Sse: common squirrel monkey (*Saimiri sciureus*), Ts: tarsier (*Tarsius syrichta*), Og: bushbaby (*Otolemur garnettii*), Tb: tree

shrew (*Tupaia belangeri*). The scale bar number indicates the number of nucleotide substitutions per site.

Supplementary Figure 4. Phylogenetic trees of the RNase6 genes and pseudogenes of the littler brown bat (Ml) and the flying fox (Pv, *Pteropus vampyrus*). The human (Hs) genes were used to root the tree. Pseudogenes are labeled gray. The scale bar number indicates the number of nucleotide substitutions per site.

Supplementary Figure 5. The protein sequence alignment of the human RNase2/EDN, RNase3/ECP, and RNase4 along with the little brown bat RNase4s. Arginines (R) are labeled with dark blue and lysines (K) are in light blue. The N-terminal signal sequences are underlined. Dashes represent alignment gaps and asterisks (*) symbolize stops codons.

Supplementary Figure 6. Phylogenetic trees of the RNase1 proteins (A) and RNase4 proteins (B) of the littler brown bat (*Myotis lucifugus*, Ml) and *Myotis davidii* (Md). For the tree in (A), three *M. davidii* RNase1 proteins identified by Zhang *et al.* and nine *M. lucifugus* RNase1 proteins identified by Xu *et al.* are included. For tree in (B), seven functional (full-length) *M. davidii* RNase4 proteins identified by Zhang *et al.* are included. Both trees were mid-point rooted. The scale bar number indicates the number of amino acid substitutions per site.

Supplementary Figure 1

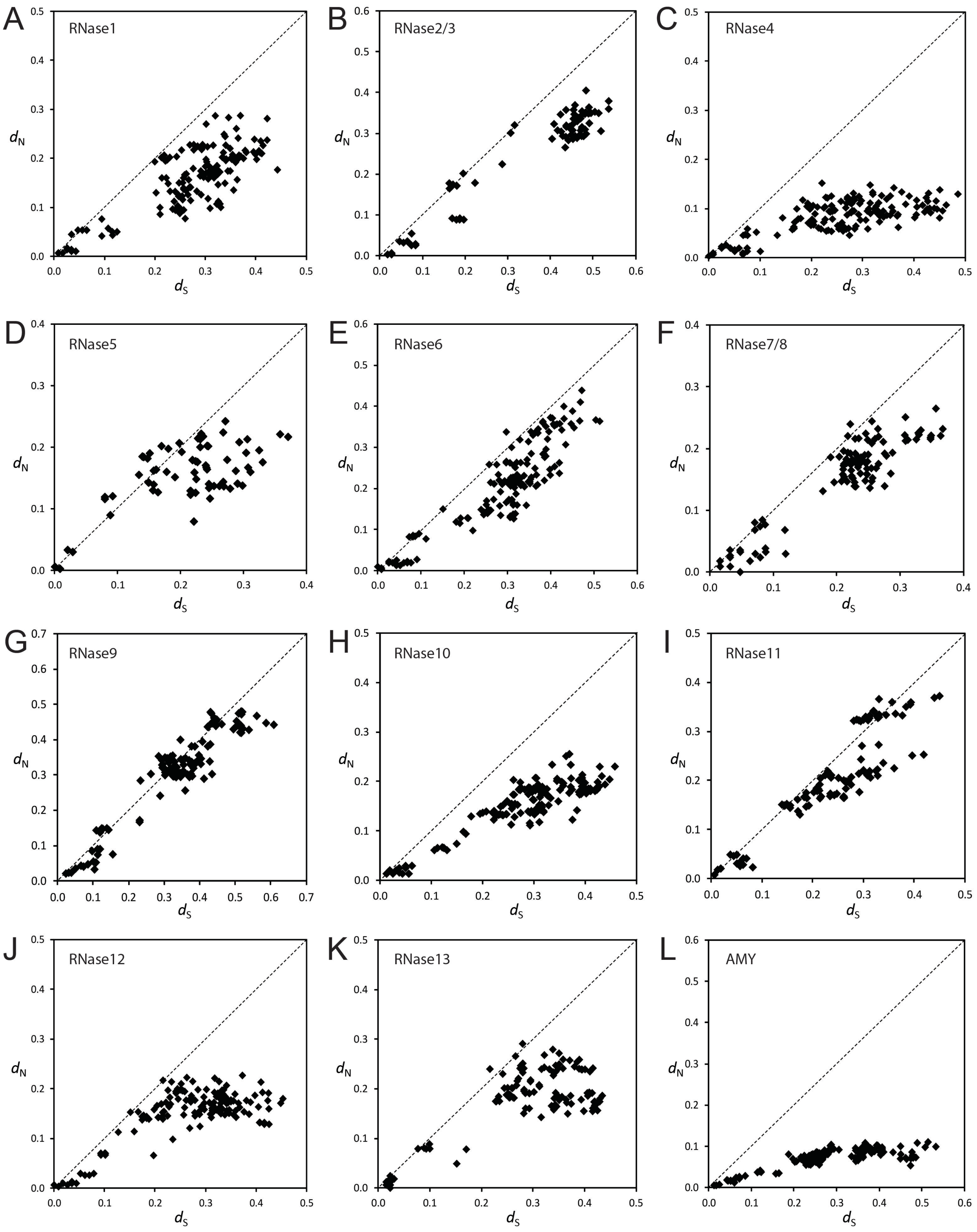

Supplementary Figure 2

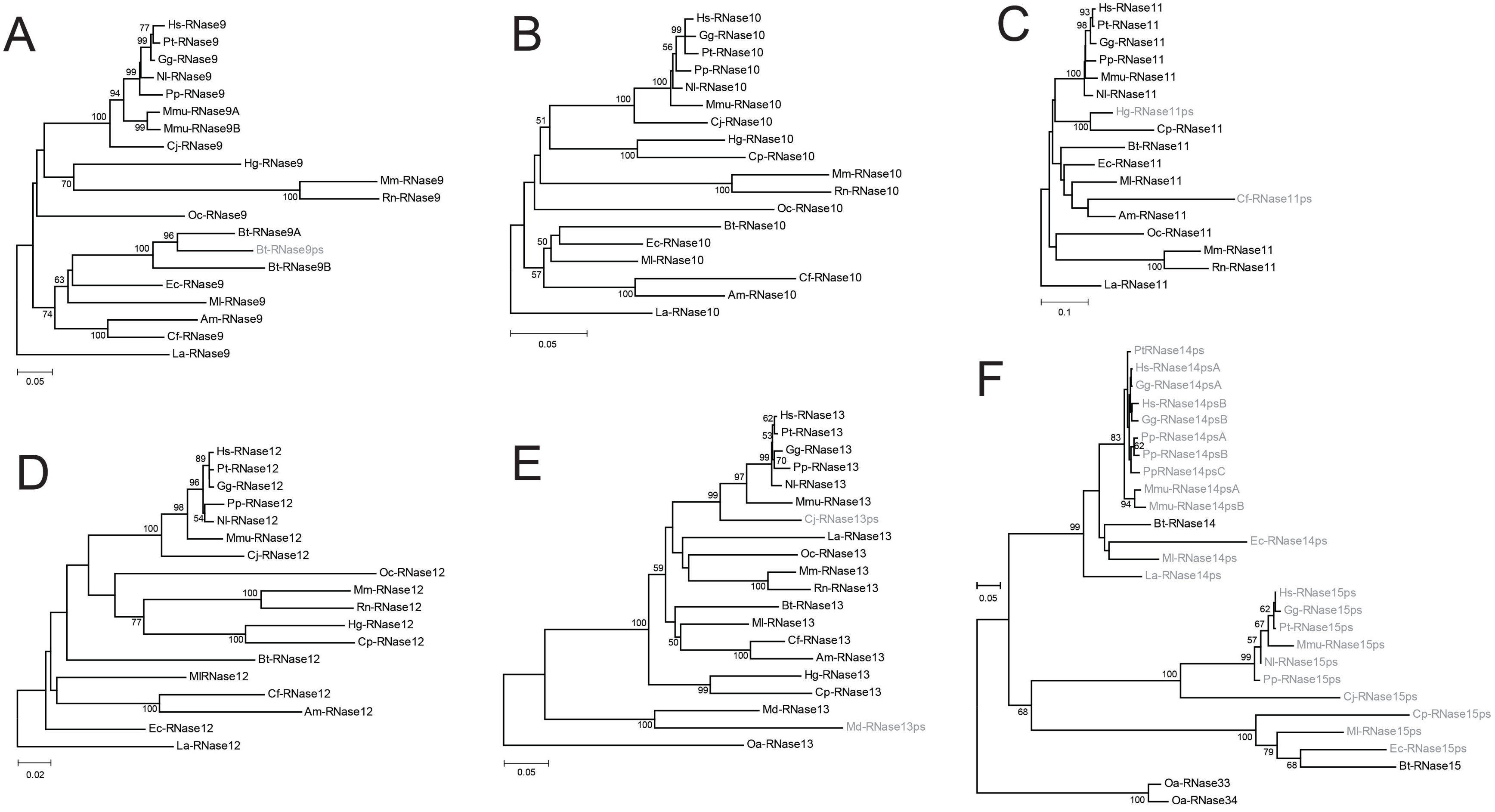

Supplementary Figure 3

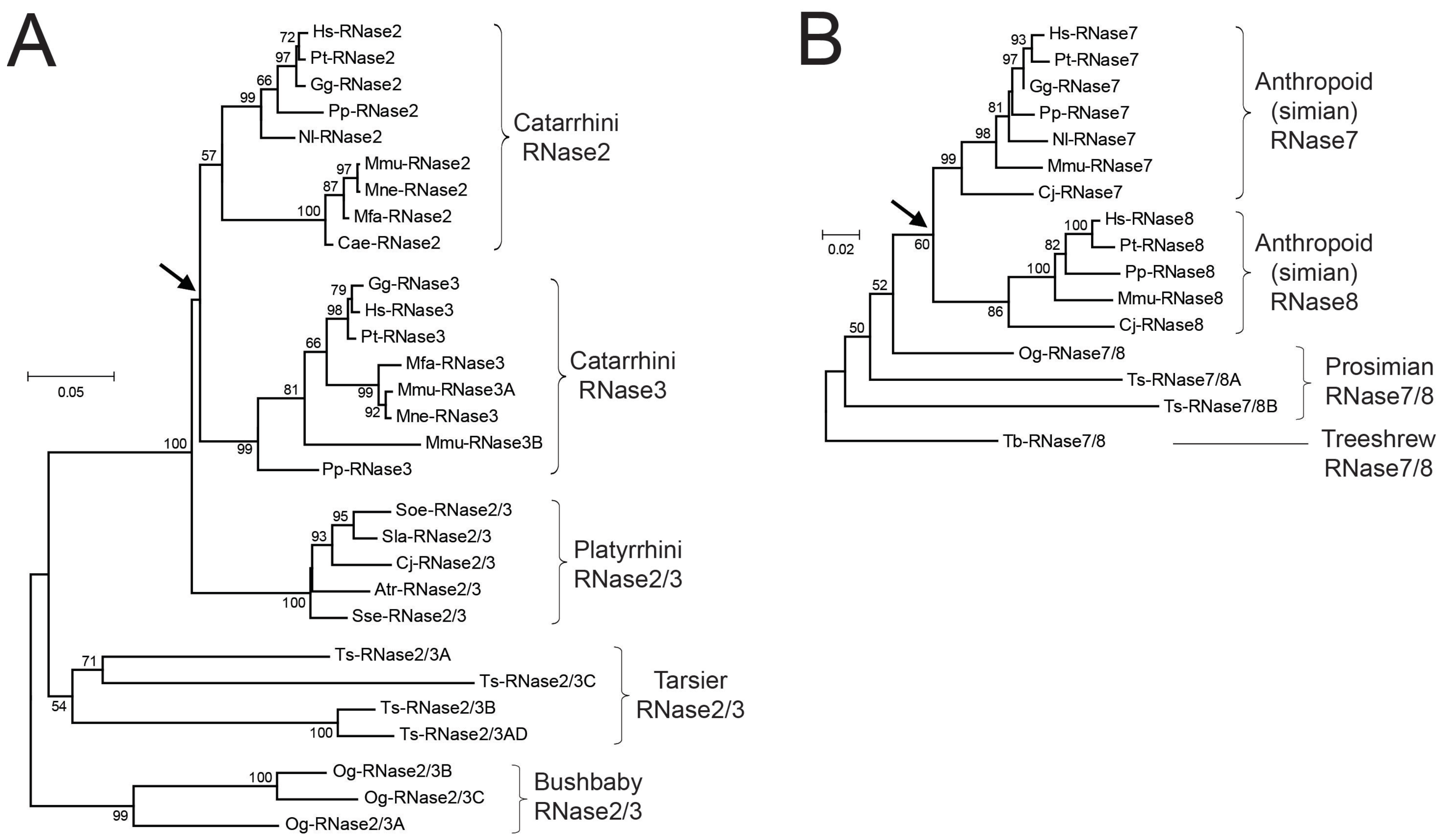

Supplementary Figure 4

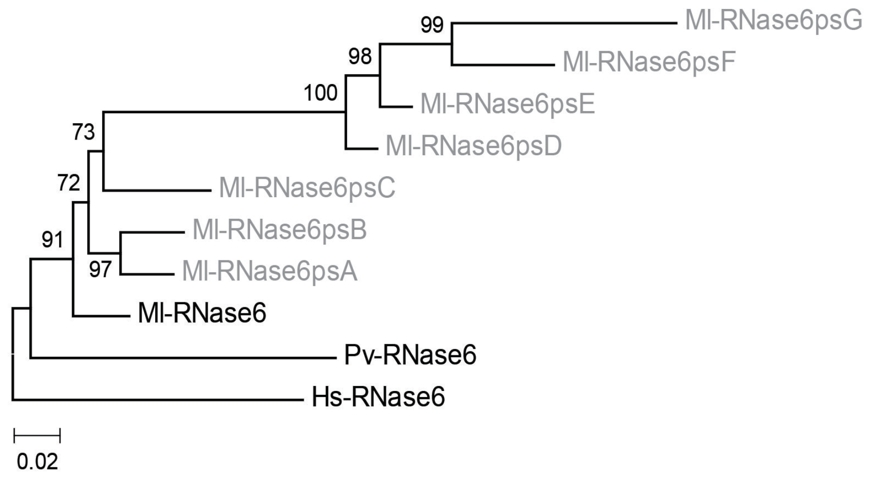

Supplementary Figure 5.

```
Hs-RNase2   MVPKLFTSQICLLLLLGLLAVEGSLHVKPPQFTWAQWFETQHINMTSQQ-----CTNAMQVINNYQRRCKNQNTFLLTT
Hs-RNase3   MVPKLFTSQICLLLLLGLMGVEGSLHARPPQFTRAQWFAIQHISLNPPR-----CTIAMRAINNYRWRCKNQNTFLRTT

Hs-RNase4   -MALQRTHSLLLLLLLTLLGL-GLVQPSYGQDGMYQRFLRQHVHPEETGGSDRYCNLMMQRRKMTLYHCKRFNTFIHED
Ml-RNase4A  -MALLKTLSSLLLLLLTLLGL-GLVQPSYGQP-MYKRFLRQHVDPTVTGGDVKYCNLMMQRRRMTQFRCKQFNTFIHED
Ml-RNase4B  -MALLKTLSSLLLLLLTLLGL-GLVQPSYGQP-MYKRFLRQHVDPTVTGGDVKYCNLMMQRRRMTQFRCKQFNTFIHED
Ml-RNase4C  -MALQKTLSLLLLLLLTLLGL-GLVQPSYSET-MYERFLRQHVDFPATGGTNLYCNTMMQRRGMTTPSCKQFNTFIHED
Ml-RNase4D  -MALQKTLSLLLLSLLTLLGL-GLVQPSYGQF-MYQRFLRQHVDSTQRGVTSSYCNTMMERRGMTRPRCKQFNTFIHAD
Ml-RNase4E  -MALQKTLSLLLLSLLTLLGL-GLVQPSYGET-MHQRFLREHVDSNWTGGNNWYCNTMMQRRGMTRPRCKQLNTFIHAD
Ml-RNase4F  -MALQKTLSLLLLSLLTLLGL-WLVQPSYGET-RYEKFQRQHVDSTGPGGTDFYCINRMDQKGMTRPKCKDFNTFIHEN
Ml-RNase4G  -MALQKTLSLLLLSLLTLLGL-WLVQPSYDQG-NYEKFQRQHVDSRGPGGTDLYCNTMMQKRGMTLDGCKKFNTFIHED
Ml-RNase4H  -MALQKTLSLLLLLLLTLLGL-GLVQPSYGQS-TYQRFLRQHVDSRGPGGTNLYCNTMMQRRGMTRPTCKQFNTFIHEN
Ml-RNase4I  -MALQKTLSLLVLSLLTLLGL-GLVQPSYGET-RNEKFQRQHVDSIGPGGTNLYCNRRMDKQEMTKLKCKPFNTFIHEN
Ml-RNase4J  -MALQKTLSLLLLSLLMLLWL-GLVQPSYGET-MYQRFLREHVDSTGTGGNSSYCKKMMQEREMTRPRCKRFNTFIHED
Ml-RNase4K  -MALQKTLSLLLLLLLTLLGL-GFVQPSYGQF-MYQPLQQQHVDSTQRGGTSLYRNTMMQRQGMTRLRCKQFNTFIHEN


Hs-RNase2   FANVVNVCGNPNMTCPSNKTRKNCHHSGSQVPLIHCNLTTPSPQNISNCRYAQTPANMFYIVACDNRDQRRDPPQYPVVPVHLDRII*
Hs-RNase3   FANVVNVCGNQSIRCPHNRTLNNCHRSRFRVPLLHCDLINPGAQNISNCTYADRPGRRFYVVACDNRDP-RDSPRYPVVPVHLDTTI*

Hs-RNase4   IWNIRSICSTTNIQC-KNG-KMNCHEGVVKVT--DCRDT--GSSRAPNCRYRAIASTRRVVIACEGN---------PQVPVHFDG*--
Ml-RNase4A  IWNIRSICSTTNIQC-KNG-KMNCHEGVMSVT--DCRDT--GSSPAPNCRYGARASTRRVVIACEGN---------PPVPVHFDG*--
Ml-RNase4B  IWNIRSICSTTNIQC-KNG-KMNCHEGVVSVT--DCRDT--GSSPAPNCRYGARASTRRVVIACEGN---------PPVPVHFDS*--
Ml-RNase4C  NSTIDTICITRNILC-KNG-MMNCHAGVVNVT--DCRDT--GRTRPPNCSYQGRASTRRVVIACNGN---------PPLPVHLDR*--
Ml-RNase4D  IGAINNICRTPNIRC-KNG-KENCHEGVVRVT--GCRLT---GSSWGNCRYQGRGSSRRVVIACEGN---------PQVPVHFDG*--
Ml-RNase4E  IGTIKNICRTPNIRC-KNG-RMNCHGRHVRVT--ECSLT---ASSQRKCSYQGRGRSRRVVIACQGN---------PPVPVRFDK*--
Ml-RNase4F  IKTINNICNNPNILC-KNRYDMNCHEGVVRVT--DCRLT----ERSPHCRYQGRGRTRRVVIACDGY---------PLVPVHFDS*--
Ml-RNase4G  IGTINNICNAPNIRC-KKG-RMNCHAGVVRVT--DCSLT--GGHSPYDCRYGARASTRRVVIACEGN---------PLVPVHFDS*--
Ml-RNase4H  IRTINNICRTTNIRC-KNG-RMNCHAGVVRVT--DCRLT---ARFPQNCRYQGRGSSRRVVIACEGN---------PLVPVHLDS*--
Ml-RNase4I  IGTINNICNNPNIRC-KNG-MMNCHAGVVRVT--DCKDT--GSSSPPNCSYKATASTRRVVIACQGN---------PLVPVHIDR*--
Ml-RNase4J  IGTIKNICKTPQIPC-KNG-KMNCHEGEVRVT--DCRLT---ARFRGNCRYQGRGSSRRVVIACQGK-----------LPVHFDK*--
Ml-RNase4K  IGAINNICRTPNIQC-RNG-KENCHEGVVRVT--DCRLT---ASFPVNCRYEGRGSSRHVVIACEGN---------PQVPVHFDG*--
```

Supplementary Figure 6.

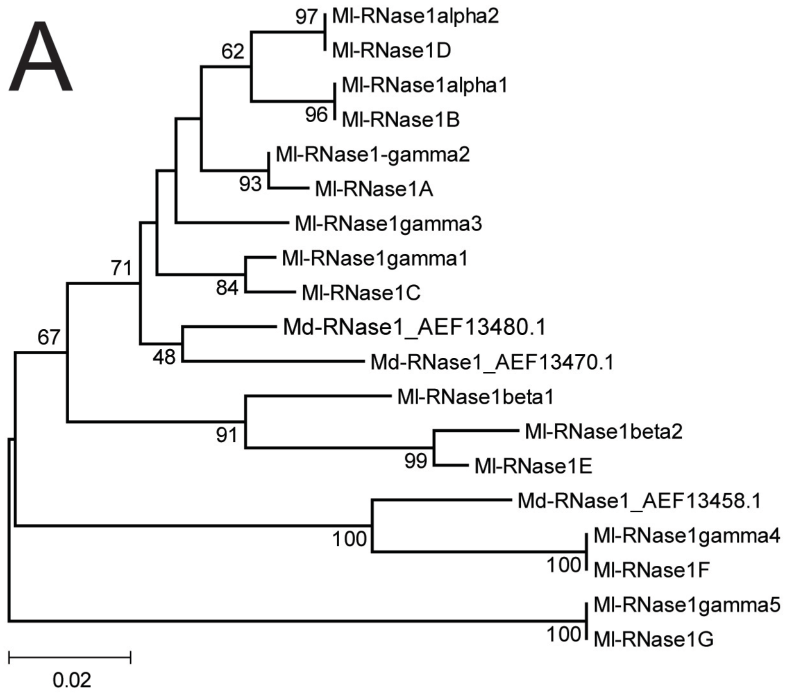

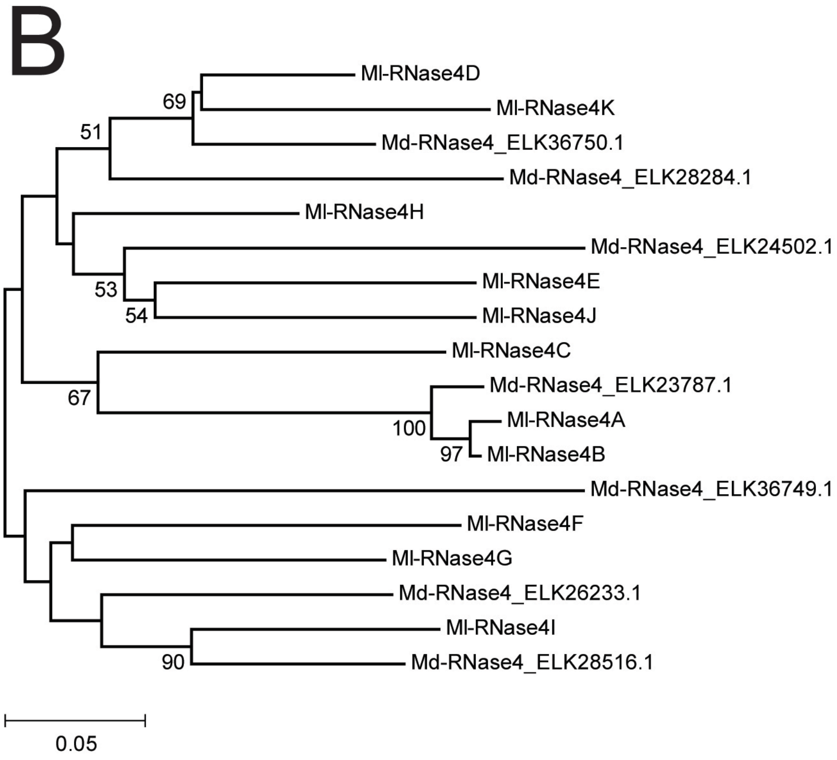

Supplement: Supplementary Data [file supp_evt161_Supplementary_Tables_and_Figures.pdf]
